# Peroxide-Mediated Release of Organophosphates from Boron-Containing Phosphotriesters: A New Class of Organophosphate Prodrugs

**DOI:** 10.1021/acs.orglett.3c02036

**Published:** 2023-07-18

**Authors:** Brittany M. Klootwyk, Amy E. Ryan, Arbil Lopez, Mitchell J. R. McCloskey, Chasity P. Janosko, Alexander Deiters, Paul E. Floreancig

**Affiliations:** Department of Chemistry, University of Pittsburgh, Pittsburgh, Pennsylvania 15260, United States

## Abstract

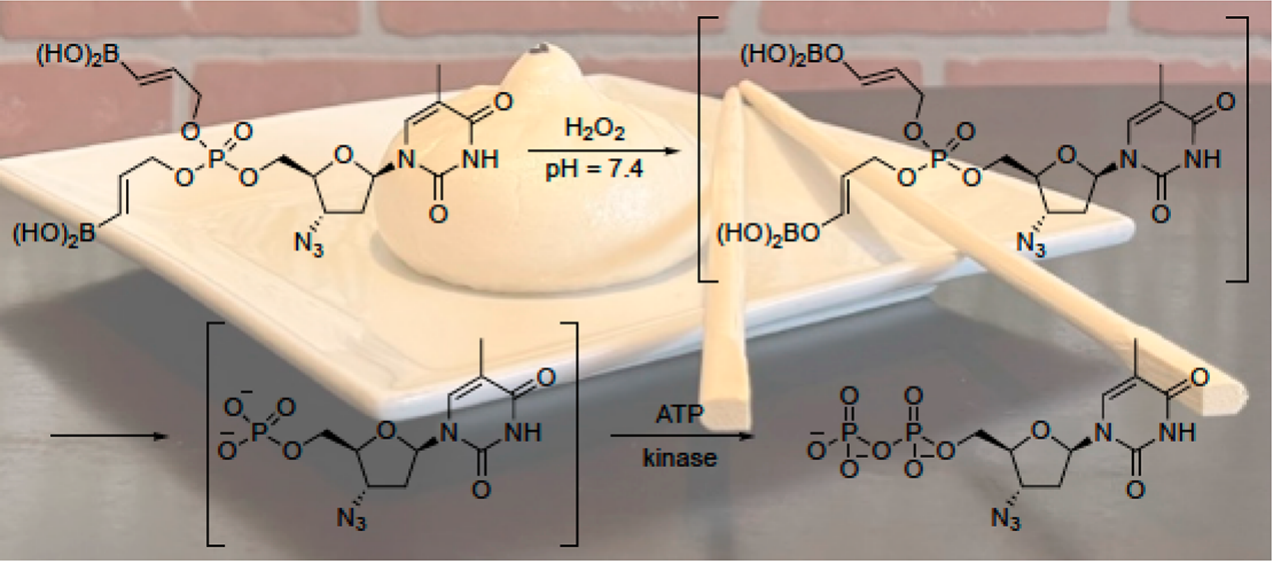

Phosphate mono- and diesters can be liberated efficiently from boryl allyloxy (BAO) and related phosphotriesters by H_2_O_2_. This protocol was applied to the release of a phosphorylated serine derivative and the nucleotide analogue AZT monophosphate. Nucleotide release in the presence of ATP and a kinase provides a diphosphate, demonstrating that this method can be applied to biological processes.

The phosphate group is an essential component of a wide range of biologically active molecules with disparate structures including nucleotide analogues (**1**)^1^ and secondary metabolites such as fostriecin (**2**) (Figure 1).^2^ Nucleoside/nucleotide anticancer and antiviral agents illustrate the challenges of employing phosphorylated molecules as therapeutic agents.^1^ Changes to nucleobases and/or the sugar unit can lead to inefficient nucleoside phosphorylation, which is necessary for disrupting nucleic acid chain elongation, thereby diminishing therapeutic efficacy. Nucleotide analogues subvert the initial phosphorylation problem since the phosphate group is already present at the 5′-site of the structure. However, the charged group substantially diminishes cell permeability and mitigates biological activity.^3^

**Figure 1:**
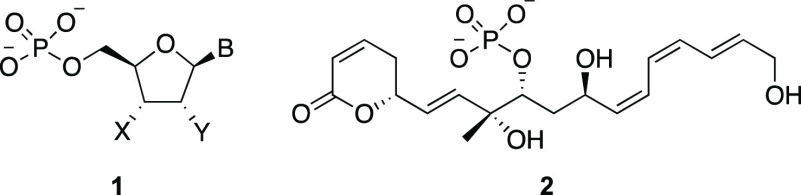
Representative biologically active phosphates.

Phosphate prodrugs address this problem (Figure 2), as seen in the clinical agents sofosbuvir (**3**) and remdesivir (**4**).^4^ These neutral agents enter cells and release nucleotide analogues through esterase-initiated cascades. Other phosphate prodrugs^5^ employ the esterase labile pivaloylmethyl and *S*-acyl-2-thioethyl groups, while cyclic phosphotriester groups are activated by acidic hydrolysis and cytochrome P450 oxidation.

**Figure 2:**
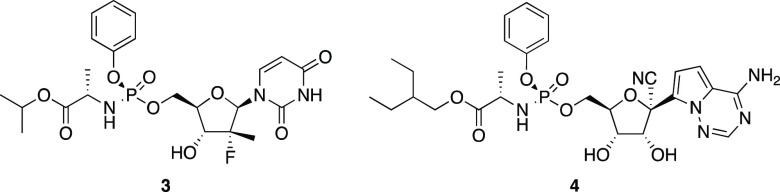
Nucleotide analogue prodrugs.

Applications of organophosphate prodrugs have been impressive, though site-selective release is challenging due to the ubiquitous presence of the releasing enzymes. Enhancing the targeted release capacity for phosphate-containing drugs offers the potential to increase their efficacy while minimizing off-target effects. Hydrogen peroxide is generated in numerous disease states, including cancer, viral infection, neurodegeneration, reperfusion injury, and arthritis.^6^ Elevated levels of H_2_O_2_ and its ability to initiate chemical reactions create the potential for its use in drug release, illustrated by the release of fluorophores^7^ and cytotoxins^8^ from boronates in cells and animals. Our prior studies in which boronate oxidation initiates fragmentation reactions that release polar groups through the generation of boron enolates from boryl allyloxy (BAO) groups^9^ or hemiacetal intermediates from α-boryl ethers^10^ led us to explore the potential of peroxide-mediated phosphate release. Herein we describe the development of a new phosphate group-release strategy based on the peroxide-mediated oxidation of borylated phosphoesters (Scheme 1). The process is rapid and efficient and can be applied to the release of a range of phosphate monoesters and diesters. Structural variants are explored to demonstrate the capacity for changing the byproduct without compromising the release efficiency. Biologically and medicinally relevant structures are liberated, and nucleotide release provides a viable substrate for subsequent kinase-mediated phosphorylation.

**Scheme 1:**
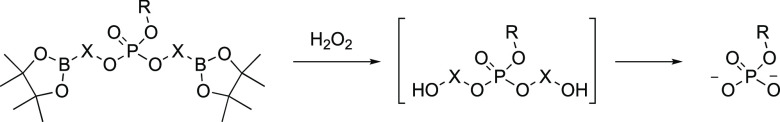
Design of Peroxide-Mediated Phosphate Releasing Agents

Proof-of-concept substrates for demonstrating phosphate release were prepared through classical phosphoramidite chemistry (Scheme 2). The substrate for phosphodiester release was prepared from **5** through sequential additions of *n*PrOH and allylic alcohol **6**(9) followed by oxidation with *t*BuOOH to yield **7**. The phosphomonoester-releasing substrate **8** was prepared by swapping the order of the nucleophile additions. These reactions are viable without intermediate phosphoramidite purification, though significantly higher throughput is achieved with purified phosphoramidites. The different yields for these reactions show that **6** is less nucleophilic than *n*PrOH and that the first step is more challenging than the second.

**Scheme 2:**
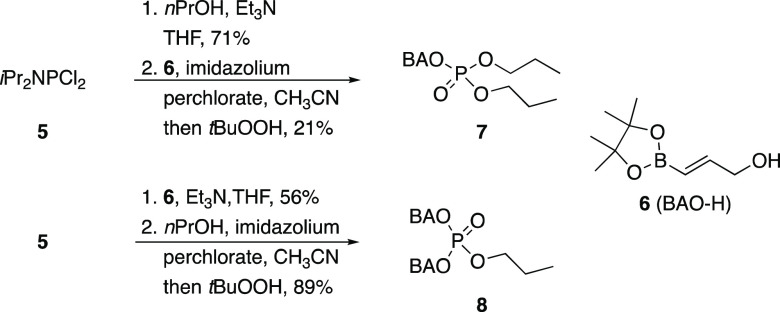
Synthesis of Oxidatively Labile Phosphates

^1^H NMR served as a convenient tool for monitoring phosphate release. Substrates were prepared as a solution in CD_3_CN and D_2_O in the presence of buffer (pH 7.4) followed by the addition of excess H_2_O_2_·urea (30 equiv) in D_2_O at 37 °C. The final concentration of the substrate was 2 mM with a CD_3_CN:D_2_O ratio of 7:3 (v/v). The resulting H_2_O_2_ concentration is higher than cellular levels,^11^ though prior related studies^10^ show that this concentration predicts cellular responses. Product concentrations were quantified against an internal standard. The products of these reactions were validated by comparison to independently prepared and characterized phosphates.^12^

Time courses for the oxidative cleavage reactions of **7** and **8** are shown in Figure 3. The cleavage of **7** proceeded rapidly and efficiently via the intermediate boron enolate, with 94% of the starting material being consumed within 18 min and phosphate **9** being produced in an equal amount. This experiment showed a pseudo-first-order rate constant of 2.3 × 10^–3^ L mol^–1^ sec^–1^ and a half-life of 301 s.^12^ The reaction with **8** was more complex since two cleavage events are required. Spectral overlap prohibited monitoring the formation of the phosphomonoester, though acrolein production is easily detected. The reaction showed a rapid consumption of **8**, with the concentration of the monocleavage product **10** increasing and then ultimately becoming negligible at 24 min. Subsequent NMR analysis confirmed that the product was phosphate **11**. Acrolein was formed in an 88% yield based on the two equiv that are expected from the cleavage of two BAO groups. The second BAO cleavage is significant, since it showed that phosphate dianion release is faster than boron enolate protonation. Additionally, the absence of a significant buildup of the monocleavage product indicates that the second cleavage is not substantially slower than the first. The pinacol esters partially hydrolyze to boronic acid intermediates prior to oxidation, although this does not impact cleavage efficiency. Phosphate release was not observed in the absence of peroxide or solely in the presence of urea.^12^

**Figure 3:**
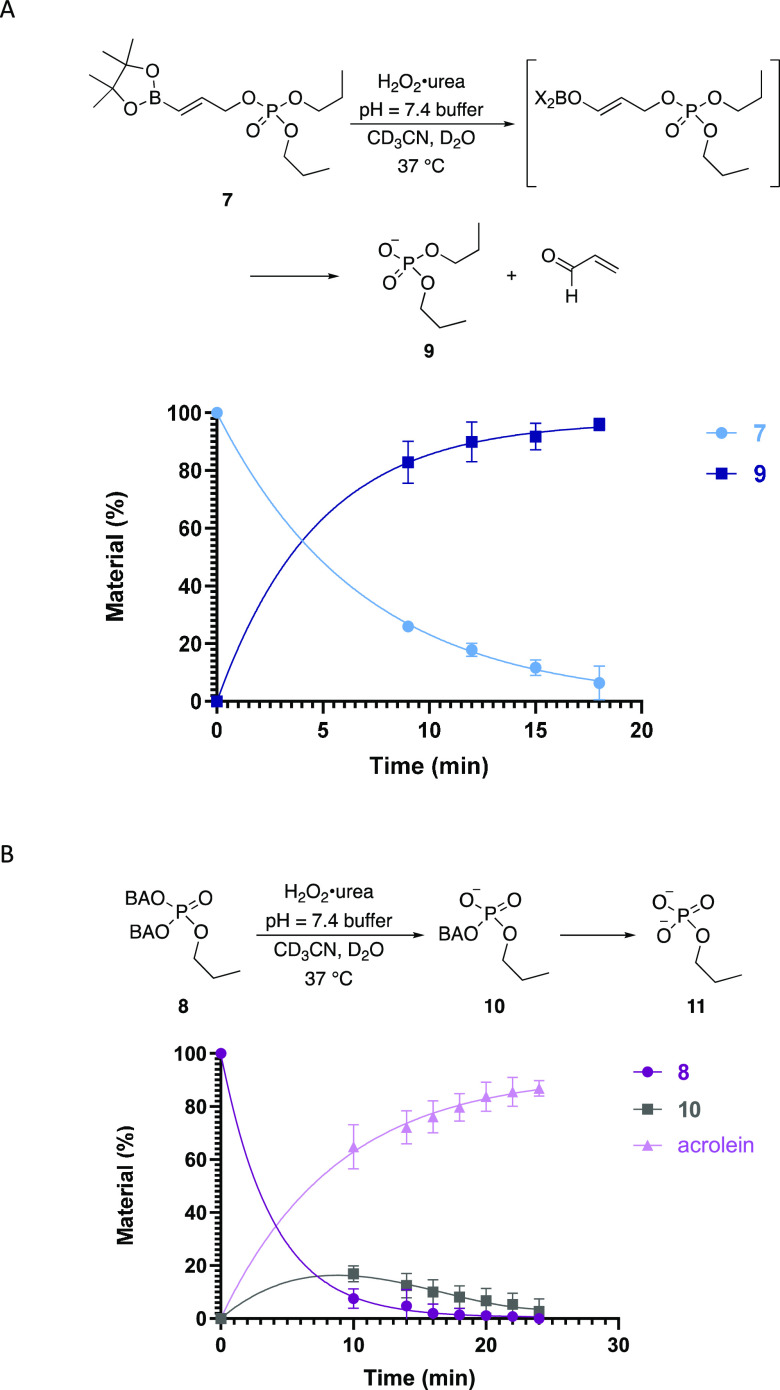
Time course of the oxidative phosphate release. (A) Cleavage to release a phosphodiester. (B) Cleavage to release a phosphomonoester via a phosphodiester intermediate. The reactions were run in triplicate, and error bars represent standard deviations from the mean.

Serveral substrates were synthesized and served to highlight the scope of the process (Table 1). The results are reported as a function of starting material consumption and product formation after 18 min for consistency in cleavage rate comparisons, unless otherwise noted.

**Table 1:**
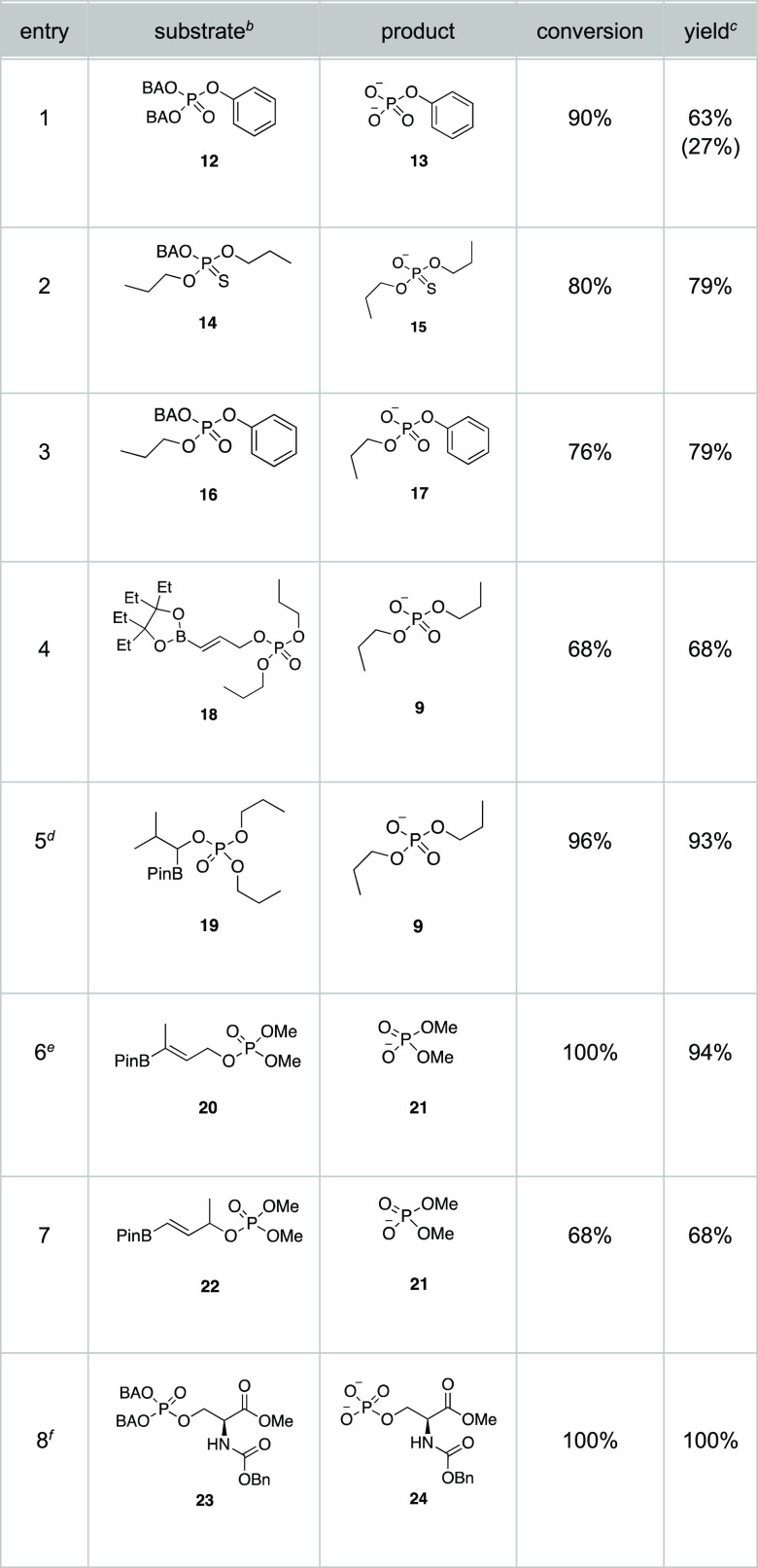
Oxidative Release of Phosphomonoesters and Phosphodiesters^a^

a Experiments were conducted at 37 °C with a substrate concentration of 2 mM and 30 equiv of H_2_O_2_·urea in CD_3_CN and D_2_O (7:3 v/v) at pH 7.4.

b See the Supporting Information for synthetic sequences and characterization data for the substrates and for starting material consumption and product formation plots.

c Calculated based on integration compared to the internal standard 1,2-dimethoxyethane. See text for times to and yields at complete conversion.

d Reaction was complete within 12 min.

e An alternate mechanism for product release was observed.

f Reaction was conducted at 0.2 mM in PBS buffer and analyzed by HPLC.

The phosphotriester **12** releases phenyl phosphate **13** somewhat more slowly than the breakdown of **8** (entry 1). While 90% of the starting material was consumed after 18 min, dicleavage product was formed in 63% yield, and 27% of the corresponding monocleavage product remained. This proceeded to 70% and 23% of the respective products after 25 min.^12^ We speculate that the rate arises from solubility issues, as turbidity was observed during the course of the reaction. The phosphorothioate **14** releases thiophosphate **15** in 79% yield after 18 min and 94% yield after 49 min, with turbidity again being observed as a potential source of the slowed release. Delivering thiophosphates, which are substantially more stable toward enzymatic cleavage than phosphates,^13^ is significant, regardless of the release rate. Phosphodiesters that contain an aryl and an alkyl group cleave selectively as shown in the conversion of **16** to **17**. Aryl phosphates can be cleaved enzymatically to release monoalkyl phosphates.^14^ Compound **18**, in which the BPin group is replaced by the recently reported and more easily handled EPin group,^15^ releases **9** extremely efficiently, albeit somewhat more slowly. Consumption with quantitative product release did not occur until 32 min. Boronate **18** is significantly more hydrolytically stable than **7**, which could be useful for applications in which the boronic acid analogue does not readily traverse a cell membrane. The diminished rate of release will be valuable when a slower phosphate production is therapeutically beneficial.

Acrolein is an inhalation toxin that can sequester glutathione,^16^ leading to concerns about its formation during prodrug cleavage, though the release of a glutathione scavenger could augment the potency of these agents in numerous applications.^17^ Although our studies with deliberate acrolein release through the cleavage of BAO-containing molecules showed no toxicity,^12^ the potential for a competitive biological response inspired us to prepare oxidatively cleavable boronate analogues that mitigate byproduct electrophilicity. The α-boryl phosphate **19** reacts with peroxide to form **9** within 12 min, indicating that this group reacts even faster than the BAO group. Boronates **20** and **22** (secBAO) are homologues of the original vinyl boronate and release methyl vinyl ketone and crotonaldehyde, respectively, in addition to **21**. These byproducts are significantly less electrophilic than acrolein.^18^ The phosphates were prepared by the addition of the alcohols to dimethylphosphoryl chloride, since the phosphoramidite protocol was unsuccessful. Both of these substrates release dimethyl phosphate in the presence of H_2_O_2_, though **20** showed an alternate, slower mechanism for release in the absence of peroxide, making structurally similar phosphates unsuitable for biological applications.^12^ This study shows that a range of options exist should byproduct release lead to a competitive biological response.

Serine derivative **23** served as a model for the conditional release of phosphoserine. The process was conducted with the initial concentration of **23** being 0.2 mM in biologically relevant PBS buffer, which is an order of magnitude more dilute than the previous studies to observe the capacity for oxidative cleavage at low substrate and oxidant concentrations. Serine phosphate **24** was formed in 89% yield after 60 min and quantitatively after 140 min as determined by HPLC, showing that phosphorylated amino acid units can be released under conditional control in peptides and proteins.^19^ Insterestingly, replacing the BAO groups of **8** with *p*-borylbenzyl groups^7,8^ resulted in oxidation but only monocleavage.^12^ Thus, the BAO group is substantially more effective for releasing dianionic phosphates.^20^

We explored the potential for nucleotide release through the preparation of AZT-derived phosphate **25** in which the boronic acid (HO-BAO group) proved desirable for isolation purposes (Scheme 3).^21^^1^H NMR studies showed that **25** reacts with H_2_O_2_ as expected to release AZT monophosphate (AZTMP) **26** in 91% yield after 25 min. Moreover, the negative control phosphate **27** proved to be completely inert under these conditions.^12^

**Scheme 3:**
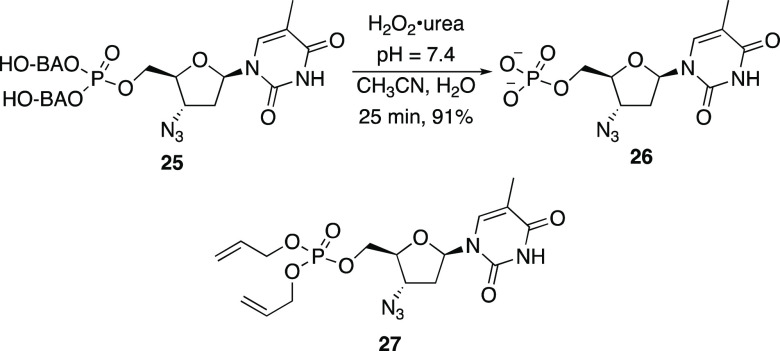
Nucleotide Analogue Release

Successful nucleotide analogue release led us to explore the viability of using peroxide-release to initiate the enzymatic phosphorylation of **26** in the presence of ATP to form AZT diphosphate **28** and ADP (Figure 4A). The F105Y mutant of thymidine monophosphate kinase (TMPK) was expressed and served to promote phosphorylation in this study.^22^ The phosphorylation assay (Figure 4B) employs the conversion of luciferin to oxyluciferin to provide an ATP-dependent luminescent signal in which ATP consumption through the phosphorylation of **26** can be determined by a luciferase assay.

**Figure 4:**
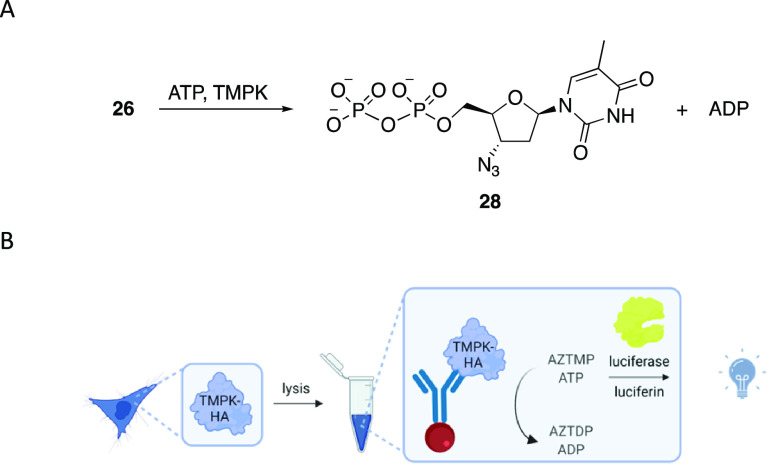
(A) ATP consumption through the phosphorylation of AZTMP to form AZT diphosphate. (B) Workflow of assessing H_2_O_2_-dependent phosphorylation of **26** by thymidine monophosphate kinase through quantification of ATP consumption using a luminescence assay.

Prior to conducting phosphorylation studies, we showed that ATP is stable to H_2_O_2_, **25**, and a mixture of **25** and H_2_O_2_ for 1 h. The assays were conducted by combining the substrate (2 μM), ATP (10 μM), and TMPK. Hydrogen peroxide (30 equiv) was added for the oxidative release studies of **25**. After 1 h CellTiter-Glo reagent was added, and luminescence was measured after 5 min. The results are shown in Figure 5. AZTMP showed a 20% reduction in luminescence, indicating complete conversion to **28**. The negative controls **27** and H_2_O_2_-free **25** failed to initiate ATP consumption. The combination of **25** and H_2_O_2_ showed a level of ATP consumption equivalent to that of the positive control **26**, confirming that oxidative cleavage provides a nucleotide analogue that can engage in enzymatic reactions.

**Figure 5:**
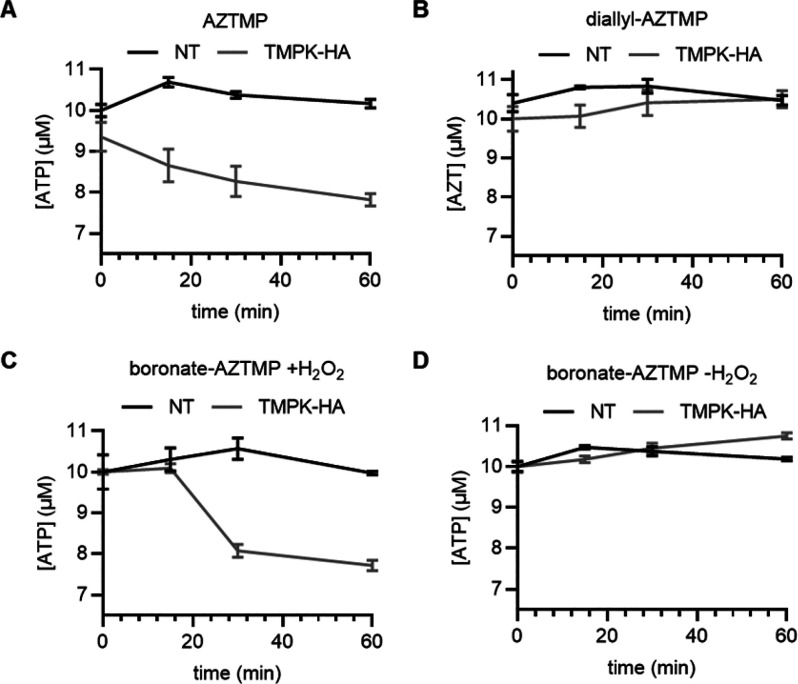
Oxidative release of **25** and subsequent phosphorylation of **26** to **28**, analyzed by a luciferase assay. Quantification of ATP concentration over 1 h in the absence (NT) or presence of immobilized TMPK-HA and 2 μM of (A) **26** (positive control), (B) **27** (negative control), (C) **25** activated with H_2_O_2_, and (D) **25** in the absence of H_2_O_2_. Error bars denote standard deviations from the mean of three replicates.

We have shown that a range of organoboronates can be incorporated into phosphotriesters to release phosphoesters upon exposure to H_2_O_2_. The reactions are rapid and efficient, even at low substrate and peroxide concentrations. Applying the protocol to AZT monophosphate release demonstrated that the released nucleotide is a suitable substrate for enzymatic phosphorylation, validating the compatibility of the process with biomolecular transformations. The elevated levels of H_2_O_2_ in numerous disease states indicate that this approach will be applicable to the development of site-selective phosphate-containing prodrugs.

## Data Availability

The data underlying this study are available in the published article and the Supporting Information.

## Supporting Information Available

Substrate preparation and characterization data, ^1^H NMR monitoring of oxidative cleavage reactions, HPLC traces, TMPK mutant expression, and enzymatic phosphorylation protocols (PDF)
FAIR data, including the primary NMR FID files, for compounds **6**–**9**, **11**–**27**, and **S1**–**S10** (ZIP)
