# Supplementary material for: Peroxide-Mediated Release of Organophosphates from Boron-Containing Phosphotriesters: A New Class of Organophosphate Prodrugs
Source: Org Lett. 2023 Jul 18;25(29):5530–5. doi: 10.1021/acs.orglett.3c02036 (PMC10391626; doi:10.1021/acs.orglett.3c02036)
Supplement: Supplementary file 1 — ol3c02036_si_001.pdf [file ol3c02036_si_001.pdf]

Supporting Information

Peroxide-Mediated Release of Organophosphates from Boron-Containing Phosphotriesters: A New Class of Organophosphate Prodrugs

Brittany M Klootwyk, Amy E. Ryan, Arbil Lopez, Mitchell J. R. McCloskey, Chasity P. Janosko, Alexander Deiters,* and Paul E. Floreancig*

Department of Chemistry
University of Pittsburgh
Pittsburgh, Pennsylvania 15260
florean@pitt.edu
deiters@pitt.edu

Table of Contents

General Experimental	S3
Experimental Procedures and Characterization	S4
Phosphate Release Studies and Product Characterization	S18
HPLC Studies on the Release of **23**	S39
Cytotoxicity Evaluation from Oxidatively Released Acrolein	S40
Kinase Studies	S42
^{1}H, ^{13}C, ^{11}B, and ^{31}P NMR Spectra	S47
References	S106

General Experimental

Proton (^{1}H) NMRs were recorded on Bruker Avance spectrometers at 300, 400, 500, and 600 MHz. Carbon (^{13}C) NMRs were recorded on Bruker Avance spectrometers at 100, 125, 151, and 176 MHz. Note that carbons that are attached to boron are broadened through quadrupolar relaxation[1] to the extent that they cannot be seen except in highly concentrated samples. The chemical shifts are reported in parts per million (ppm) on the delta (δ) scale. The solvent peak was used as a reference value, for ^{1}H NMR: $CDCl_3$ = 7.26 ppm, CD_3CN = 1.94 ppm, for ^{13}C NMR: $CDCl_3$ = 77.2 ppm, CD_3CN = 1.3 ppm and 118.3 ppm. Boron (^{11}B) and phosphorus (^{31}P) NMRs were recorded on Bruker Avance spectrometers at 128 or 160 MHz, and 162 or 202 MHz, respectively. The ^{11}B and ^{31}P chemical shifts were referenced to the IUPAC-approved unified scale.[2] The coupling data are reported as follows: s = singlet; d = doublet; t = triplet; q = quartet; quin = quintet; m = multiplet. High resolution mass spectra were collected on a ThermoFisher Q-Exactive Orbitrap instrument.

All distillations were performed under N_2 unless otherwise stated. Methylene chloride and acetonitrile were distilled from calcium hydride. Tetrahydrofuran was distilled over sodium/benzophenone. Methanol was distilled from ground calcium sulfate. Analytical TLC was performed on E. Merck pre-coated (25 mm) silica gel 60 F254 plates. Visualization was done under UV (254 nm) and by staining with anisaldehyde or $KMnO_4$ stain. Flash chromatography was done using SiliCycle SiliaFlash P60 40-63µm 60 Å silica gel. Reagent grade ethyl acetate, methanol, diethyl ether, acetonitrile, dichloromethane, and hexanes (commercial mixture) were purchased from Fisher Scientific and were used as-is for chromatography. All reactions were performed in flame-dried glassware under a positive pressure of Ar with magnetic stirring unless noted otherwise.

Boronate compounds should be rapidly purified by column chromatography to avoid decomposition. We found that using boric acid-treated silica gel helped to limit product degradation.[3]

Boric acid impregnated silica gel was generated following a previously reported literature procedure.[4] Silica gel (300 mL) was mixed with boric acid (28.0 g) and ethanol (550 mL) for two hours at room temperature. The silica gel was filtered and washed with ethanol three times (200 mL). The silica gel was dried overnight on a vacuum filtration setup and then dried in a 100 °C oven for 48 hours.

Yields of phosphates are reported based on ^{1}H NMR spectra against an internal standard unless otherwise noted. Separation conditions are reported to obtain material of sufficient purity for characterization and oxidative cleavage experiments.

Experimental Procedures and Characterization

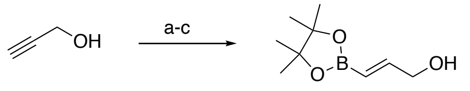

6

Reagents and conditions
a) TBSCl, imidazole, CH_2Cl_2, 96%. b) Pinacolbrane, $Cp_2Zr(H)Cl$, Et_3N, 70 °C, 57%. c) CSA, MeOH, CH_2Cl_2, 81%

Scheme S1. Synthesis of **6** (BAO–H).

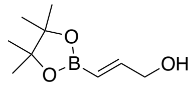

(*E*)-3-(4,4,5,5-Tetramethyl-1,3,2-dioxaborolan-2-yl)prop-2-en-1-ol (6)

Propargyl alcohol (4.11 g, 73.4 mmol) was added to a solution of imidazole (9.99 g, 147 mmol) in methylene chloride (147 mL). The reaction was cooled to 0 °C and *t*-butyldimethylsilyl chloride (13.3 g, 88.4 mmol) was added. After stirring for 1 h, the reaction was quenched with saturated ammonium chloride (aqueous, 200 mL), extracted twice with methylene chloride (150 mL), dried over sodium sulfate, and filtered. After concentrating the crude liquid, the alkyne was purified via column chromatography (10% EtOAc in hexanes) to give the desired pale-yellow liquid (11.9 g, 96%). Pinacol borane (9.44 g, 73.6 mmol) was added carefully (gas evolution) to a mixture of the silyl ether (11.9 g, 70.1 mmol), $Cp_2Zr(H)Cl$ (1.81 g, 7.01 mmol), and triethylamine (0.711 g, 7.01 mmol). The reaction was heated to 70 °C and stirred overnight. The reaction was diluted with 5% EtOAc in hexanes, filtered, and loaded directly onto a flash column for purification (5-10% EtOAc in hexanes gradient) to give the desired clear oil (11.9 g, 57%). A solution of the resulting vinyl boronate (11.9 g, 39.9 mmol) in 1:1 (v:v) CH_2Cl_2 and MeOH (266 mL) was cooled to 0 °C. Camphorsulfonic acid (0.93 g, 3.99 mmol) was added, and the reaction stirred for 1 h at rt. The mixture was concentrated *in vacuo* and purified by column chromatography (20-30% EtOAc in hexanes) to give the desired clear oil (5.90 g, 81%).

^{1}H NMR (500 MHz, $CDCl_3$) δ 6.74 (dt, *J* = 18.2, 4.0 Hz, 1H), 5.70 (app d, *J* = 18.1 Hz, 1H), 4.24 (dd, *J* = 3.9, 1.5 Hz, 2H), 1.27 (s, 12H)
^{13}C NMR (100 MHz, $CDCl_3$) δ 151.8, 83.5, 64.7, 24.9
^{11}B NMR (128 MHz, $CDCl_3$) δ 29.7
HRMS (ESI) $C_9H_{18}BO_3$ $[M+H]^+$ 185.1344, found 185.1343

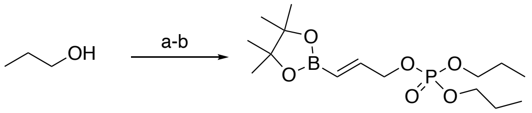

7

Reagents and conditions
a) $^{i}Pr_2NPCl_2$, Et_3N, THF, 0 °C. b) **6**, $ImHClO_4$, CH_3CN, then $^{t}BuOOH$.

Scheme S2. Synthesis of **7**.

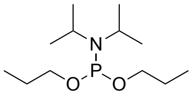

Dipropyl diisopropylphosphoramidite (S1)

A solution of $^{i}Pr_2NPCl_2$ (0.553 g, 2.74 mmol) in THF (7.4 mL) was cooled to –78 °C. A solution of n-PrOH (0.329 g, 5.47 mmol) and Et_3N (1.108 g, 10.95 mmol) in THF (7.4 mL) was added dropwise. The reaction was warmed to rt and was stirred for 4 h. The mixture was filtered and the salt was washed twice with THF (5.0 mL). The filtrate was concentrated, and the crude product was purified by flash chromatography (14% EtOAc in hexanes with 1% triethylamine) to yield the phosphoramidite (0.485 g, 71%) as a clear liquid.

^{1}H NMR (400 MHz, $CDCl_3$) δ 3.65 – 3.50 (m, 6H), 1.60 (td, *J* = 7.2, 7.2 Hz, 4H), 1.18 (d, *J* = 6.8 Hz, 12H) 0.94 (t, *J* = 7.4 Hz, 6H)
^{13}C NMR (100 MHz, $CDCl_3$) δ 65.2 (d, *J* = 16.9 Hz), 43.1 (d, *J* = 12.5 Hz), 24.9 (d, *J* = 6.8 Hz), 24.5 (d, *J* = 7.4 Hz), 10.4
^{31}P NMR (162 MHz, $CDCl_3$) δ 145.4
HRMS (ESI) *m/z* calcd. for $C_{12}H_{29}O_2NP$ $[M+H]^+$ 250.1930, found 250.1929

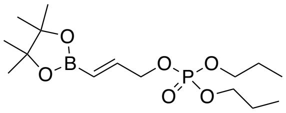

(*E*)-Dipropyl (3-(4,4,5,5-tetramethyl-1,3,2-dioxaborolan-2-yl)allyl) phosphate (7)

To imidazolium perchlorate (0.328 g, 1.95 mmol), alcohol **6** (0.299 g, 1.62 mmol), CH_3CN (4.5 mL) and powdered molecular sieves (4 Å, 0.300 g) was added a solution of the phosphoramidite **S1** (0.485 g, 1.95 mmol) in CH_3CN (4.5 mL). The reaction was stirred for 2 h. The mixture was cooled to 0 °C and *t*-butyl hydroperoxide (0.5 M in toluene, 9.7 mL, 4.9 mmol) was added. The reaction was stirred for 10 min and then quenched with Me_2S (0.302 g, 4.86 mmol). The mixture was concentrated, diluted with CH_2Cl_2 (10 mL), and was washed with brine (10 mL) twice. The organic layer was dried with $MgSO_4$, filtered, and concentrated *in vacuo*. ^{1}H NMR showed a yield of 65% relative to the internal standard 1,2-dimethoxyethane. For kinetics, the crude mixture was rigorously purified via column chromatography (25-35% EtOAc in hexanes). Residual solvent was removed on a vacuum line for 1 h at 90 °C to yield the pure phosphate (0.118 g, 21%) as a clear, colorless oil.
^{1}H NMR (400 MHz, CD_3CN) δ 6.54 (dt, *J* = 18.1, 4.4 Hz, 1H), 5.68 (dt, *J* = 18.1, 1.7 Hz, 1H), 4.55 (ddd, *J* = 8.3, 4.5, 1.8 Hz, 2H), 3.96 (td, *J* = 6.7, 6.7 Hz, 4H), 1.66 (sextet, *J* = 7.1 Hz, 4H), 1.24 (s, 12H), 0.93 (t, *J* = 7.4 Hz, 6H)
^{13}C NMR (100 MHz, CD_3CN) δ 147.4 (d, *J* = 7.3 Hz), 84.3, 70.0 (d, *J* = 5.9 Hz), 68.9 (d, *J* = 5.1
^{11}B NMR (128 MHz, CD_3CN) δ 29.6
^{31}P NMR (162 MHz, CD_3CN) δ –0.91
Hz), 25.1, 24.3 (d, *J* = 6.6 Hz), 10.3
HRMS (ESI) *m/z* calcd. for $C_{15}H_{31}O_6BP$ $[M+H]^+$ 349.1946, found 349.1952

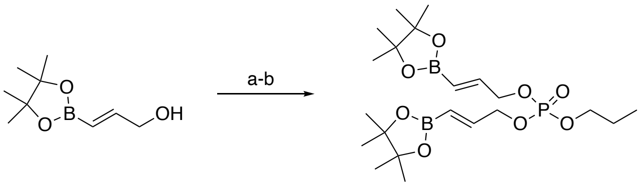

6 **8**

Reagents and conditions
a) $^{i}Pr_2NPCl_2$, Et_3N, THF, 0 °C. b) PrOH, $ImHClO_4$, CH_3CN, then ^{t}BuOOH.

Scheme S3. Synthesis of **8**.

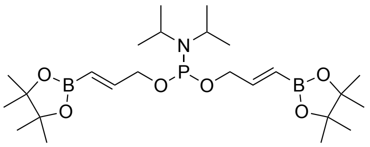

Bis((*E*)-3-(4,4,5,5-tetramethyl-1,3,2-dioxaborolan-2-yl)allyl) diisopropylphosphoramidite (S2)

A solution of $^{i}Pr_2NPCl_2$ (1.90 g, 9.39 mmol) in THF (25 mL) was cooled to –78 °C. A solution of alcohol **6** (3.46 g, 18.8 mmol) and Et_3N (3.80 g, 37.6 mmol) in THF (25 mL) was added dropwise. The reaction was warmed to rt and was stirred for 3 h. The mixture was filtered and the salt was washed twice with THF (25 mL). The filtrate was concentrated, and the crude product was purified

by flash chromatography (14% EtOAc in hexanes with 1% triethylamine) to yield the phosphoramidite (0.677 g, 55%) as a clear liquid.
^{1}H NMR (400 MHz, CD_3CN) δ 6.61 (dt, *J* = 18.0, 3.9 Hz, 2H), 5.69 (app d, *J* = 18.0 Hz, 2H), 4.25 (two overlapping dddd, 4H), 3.66 (m, 2H), 1.26 (s, 24H), 1.20 (d, *J* = 6.7 Hz, 16H)
^{13}C NMR (100 MHz, CD_3CN) δ 150.7 (d, *J* = 6.8 Hz), 83.7, 65.1 (d, *J* = 17.6 Hz), 43.4 (d, *J* = 12.3 Hz), 24.7, 24.5 (d, *J* = 7.3 Hz)
^{11}B NMR (128 MHz, CD_3CN) δ 29.8
^{31}P NMR (202 MHz, CD_3CN) δ 147.8
HRMS (ESI) *m/z* calcd. for $C_{24}H_{47}B_2NO_6P$ $[M+H]^+$: 498.3322, found 498.3311

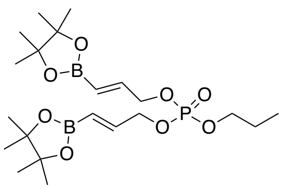

Propyl bis((*E*)-3-(4,4,5,5-tetramethyl-1,3,2-dioxaborolan-2-yl)allyl) phosphate (8)
To imidazolium perchlorate (0.320 g, 1.90 mmol), n-propanol (0.095 g, 1.6 mmol), CH_3CN (4.5 mL) and powdered molecular sieves (4 Å, 0.300 g) was added a solution of phosphoramidite **S2** (0.945 g, 1.90 mmol) in CH_3CN (4.5 mL). The reaction was stirred for 1 h. The mixture was cooled to 0 °C and *t*-butyl hydroperoxide (0.5 M in toluene, 9.5 mL, ~4.7 mmol) was added. The reaction was stirred for 10 min and then quenched with Me_2S (0.294 g, 4.74 mmol). The mixture was concentrated, diluted with CH_2Cl_2 (10 mL), and was washed with brine (10 mL) twice. The organic layer was dried with $MgSO_4$, filtered, and concentrated *in vacuo*. Residual solvent was removed on a vacuum line for 1 h at rt to yield the pure phosphate (0.666 g, 89%) as a colorless oil.
^{1}H NMR (500 MHz, CD_3CN) δ 6.54 (dt, *J* = 18.0, 4.2 Hz, 2H), 5.68 (dt, *J* = 18.1, 1.6 Hz, 2H), 4.57 (dd, *J* = 4.5, 1.8 Hz, 2H), 4.56 (dd, *J* = 4.5, 1.8 Hz, 2H), 3.98 (td, *J* = 6.7, 6.7 Hz, 2H), 1.66 (sextet, *J* = 7.0 Hz, 2H), 1.24 (s, 24H), 0.93 (t, *J* = 7.4 Hz, 3H)
^{13}C NMR (125 MHz, CD_3CN) δ 147.2 (d, *J* = 6.9 Hz), 84.4, 70.3 (d, *J* = 5.4 Hz), 69.1 (d, *J* = 5.3 Hz), 25.1, 24.3 (d, *J* = 7.3 Hz), 10.3
^{11}B NMR (160 MHz, CD_3CN) δ 29.7
^{31}P NMR (202 MHz, CD_3CN) δ –0.92
HRMS (ESI) *m/z* calcd. for $C_{21}H_{40}O_8B_2P$ $[M+H]^+$ 473.2641, found 473.2651

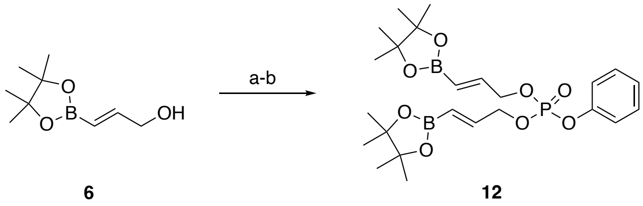

Reagents and conditions
a) $^{i}Pr_2NPCl_2$, Et_3N, THF, 0 °C. b) PhOH, 1H-tetrazole, CH_3CN, then $^{t}BuOOH$.

Scheme S4. Synthesis of **12**.

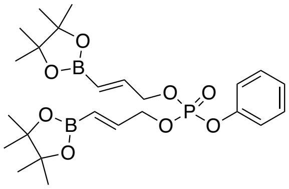

Phenyl bis((*E*)-3-(4,4,5,5-tetramethyl-1,3,2-dioxaborolan-2-yl)allyl) phosphate (12)
A solution of $^{i}Pr_2NPCl_2$ (0.83 g, 4.1 mmol), Et_3N (1.04 mL, 10.3 mmol), and THF (1.0 M) was cooled to –10 °C. A solution of **6** (1.51 g, 8.21 mmol) in THF (1.4 mL) was added to the flask dropwise. The reaction was warmed to rt and stirred for 2 h. The heterogeneous mixture was

filtered under N_2 and the salt was washed twice with THF (5.0 mL). The crude phosphoramidite was concentrated and used immediately in the next step.

To a solution of 1H-tetrazole (0.45 M in CH_3CN, 5.0 mL, 2.3 mmol) was added the phosphoramidite (1.02 g, 2.05 mmol). A solution of PhOH (97 mg 1.0 mmol,) in CH_3CN (5.6 mL) was added, and then the reaction was stirred for 90 min. The mixture was cooled to 0 °C and *t*-butyl hydroperoxide (5.0-6.0 M solution in decane, 0.56 mL, ~3.1 mmol) was added. The reaction was stirred for 10 min and then quenched with dimethyl sulfide (0.19 g, 3.1 mmol). The mixture was concentrated. ^{1}H NMR showed a yield of 41% relative to the internal standard 1,2-dimethoxyethane. For kinetics, the crude mixture was rigorously purified via column chromatography (20-40% EtOAc in hexanes). Residual solvent was removed on a vacuum line for 1 h at 60 °C to yield the desired phosphate as a clear oil (0.101 g, 19%).

^{1}H NMR (400 MHz, CD_3CN) δ 7.33 (m, 2H), 7.17 (m, 3H), 6.47 (dt, *J* = 18.0, 4.4 Hz, 2H), 5.62 (dt, *J* = 17.6 Hz, 2H), 4.65 (dd, *J* = 4.5, 1.2 Hz, 2H), 4.62 (dd, *J* = 4.6, 1.3 Hz, 2H), 1.18 (s, 24H)

^{13}C NMR (100 MHz, CD_3CN) δ 151.2 (d, *J* = 7.2 Hz), 146.2 (d, *J* = 6.8 Hz), 130.5, 125.9 (d, *J* = 1.5 Hz), 120.7 (d, *J* = 5.1 Hz), 84.0, 69.6 (d, *J* = 5.1 Hz), 30.5, 24.7

^{11}B NMR (128 MHz, CD_3CN) δ 29.7

^{31}P NMR (162 MHz, CD_3CN) δ –6.24

HRMS (ESI) *m/z* calcd. for $C_{24}H_{38}O_8B_2P$ $[M+H]^+$ 507.2485, found 507.2469

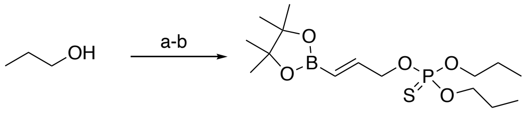

14

Reagents and conditions

a) $^iPr_2NPCl_2$, Et_3N, THF, 0 °C. b) **6**, 1H-tetrazole, CH_3CN, then xanthane hydride.

Scheme S5. Synthesis of **14**.

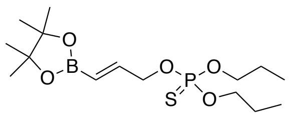

(*E*)-*O*,*O*-Dipropyl *O*-(3-(4,4,5,5-tetramethyl-1,3,2-dioxaborolan-2-yl)allyl) phosphorothioate (14)

A solution of $^iPr_2NPCl_2$ (0.50 g, 2.5 mmol), triethylamine (0.53 g, 5.3 mmol), and THF (2.5 mL) was cooled to –10 °C. A solution of n-propanol (0.31 g, 5.1 mmol) in THF (0.9 mL) was added to the flask dropwise. The reaction was warmed to rt and stirred for 2 h. The heterogeneous mixture was filtered under N_2 and the salt was washed twice with THF (3.0 mL). The crude phosphoramidite was concentrated and used immediately in the next step.

To a solution of 1H-tetrazole (0.45 M in CH_3CN, 1.6 mL, 0.70 mmol) was added the phosphoramidite (0.175 g, 0.702 mmol). A solution of **6** (0.065 g, 0.35 mmol) in CH_3CN (2 mL) was added and the reaction was stirred at rt for 1 h. Xanthane hydride (0.105 g, 0.702 mmol) was added and the reaction was stirred for 10 min. After concentration the crude mixture was dissolved in CH_2Cl_2 (3.0 mL), filtered, and concentrated to dryness. ^{1}H NMR showed a yield of 63% relative to the internal standard 1,2-dimethoxyethane. For kinetics, the crude mixture was rigorously purified via column chromatography (20% EtOAc in hexanes followed by 30% CH_2Cl_2 in hexanes) to yield the desired thiophosphate as a yellow oil (0.083 g, 63%).

^{1}H NMR (400 MHz, CD_3CN) δ 6.57 (dt, *J* = 18.0, 4.4 Hz, 1H), 5.71 (d, *J* = 18.0 Hz, 1H), 4.63 (dd, *J* = 10.1, 4.4 Hz, 2H), 4.03 (td, *J* = 7.2, 7.2 Hz, 4H), 1.70 (sextet, *J* = 7.0 Hz, 4H), 1.27 (s, 12H), 0.96 (t, *J* = 7.6 Hz, 6H)

^{13}C NMR (100 MHz, CD_3CN) δ 146.8 (d, *J* = 7.3 Hz), 83.9, 70.3 (d, *J* = 6.8 Hz), 69.1 (d, *J* = 4.9 Hz), 24.7, 23.7 (d, *J* = 7.5 Hz), 10.0
^{11}B NMR (128 MHz, CD_3CN) δ 29.7
^{31}P NMR (162 MHz, CD_3CN) δ 68.2
HRMS (ESI) *m/z* calcd. for $C_{15}H_{31}O_5BPS$ $[M+H]^+$ 365.1717, found 365.1715

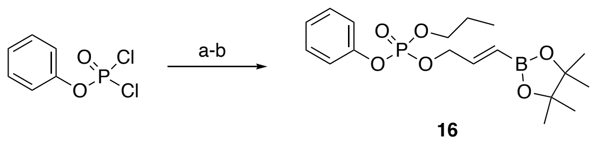

Reagents and conditions
a) ^{n}PrOH, Pyr., Et_2O. b) **6**, *N*-methylimidazole, THF.

Scheme S6. Synthesis of **16**.

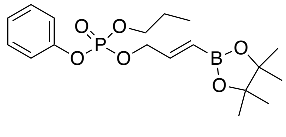

(*E*)-Phenyl propyl (3-(4,4,5,5-tetramethyl-1,3,2-dioxaborolan-2-yl)allyl) phosphate (16)

A solution of ^{n}PrOH (0.15 g, 2.3 mmol,) and pyridine (0.19 g, 2.3 mmol) was added dropwise to $PhOP(O)Cl_2$ (0.49 g, 2.3 mmol) in Et_2O (6.2 mL) at 0 °C. The reaction was stirred for 30 min then was warmed to rt and stirred for another 30 min. The mixture was filtered and the solid was triturated and washed with anhydrous Et_2O (3x). The filtrate was concentrated *in vacuo* and the crude, colorless liquid (83% NMR yield) was moved forward without further purification.

The chlorophosphate (0.480 g crude) in THF (9.5 mL) was dissolved in a solution of boronate **6** (0.314 g, 1.71 mmol) in THF (0.5 mL), then was cooled to –78 °C. *N*-methyl imidazole (0.70 g, 8.5 mmol) was added dropwise and the reaction was stirred at –78 °C for 10 min, then was warmed to rt and stirred overnight. The mixture was concentrated and diluted with H_2O (10 mL). The solution was extracted with CH_2Cl_2 (10 mL, 3x), dried with $MgSO_4$, filtered, and concentrated. The crude oil was purified via column chromatography (5-30% CH_3CN in CH_2Cl_2). Residual solvent was removed on a vacuum line for 1 h. at 60 °C to yield the desired phosphate as a clear oil (0.103 g, 15%).

^{1}H NMR (400 MHz, CD_3CN) δ 7.39 (t, *J* = 7.9 Hz, 2H), 7.22 (td, *J* = 8.6, 1.0 Hz, 3H), 6.53 (dt, *J* = 18.1, 4.2 Hz, 1H), 5.68 (dt, *J* = 18.1, 1.8 Hz, 1H), 4.68 (ddd, *J* = 8.6, 4.6, 0.7 Hz, 2H), 4.10 (td, *J* = 6.8, 6.8 Hz, 2H), 1.69 (m, 2H), 1.23 (s, 12H), 0.93 (t, *J* = 7.4 Hz, 3H)
^{13}C NMR (100 MHz, CD_3CN) δ 151.7 (d, *J* = 6.8 Hz), 146.8 (d, *J* = 6.7 Hz), 130.8, 126.1, 121.0 (d, *J* = 4.5 Hz), 84.3, 71.1 (d, *J* = 6.6 Hz), 69.7 (d, *J* = 5.8 Hz), 25.0, 24.3 (d, *J* = 7.3 Hz), 10.2
^{11}B NMR (128 MHz, CD_3CN) δ 29.6
^{31}P NMR (162 MHz, CD_3CN) δ –6.20
HRMS (ESI) *m/z* calcd. for $C_{18}H_{29}O_6BP$$[M+H]^+$ 383.1789, found 383.1800

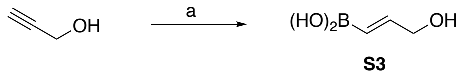

Reagents and conditions
a) Catecholborane, then H_2O.

Scheme S7. Synthesis of allyl alcohol boronic acid.

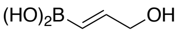

(*E*)-(3-Hydroxyprop-1-en-1-yl)boronic acid (S3)

Catecholborane (1.23 g, 10.3 mmol) was added dropwise at 0 °C to propargyl alcohol (0.28 g, 5.0 mmol) with vigorous stirring and venting. The solution was warmed to rt and stirred overnight.

The resulting solid was cooled to 0 °C and water was added in 1 mL increments until gas evolution ceased (5 mL). The mixture was stirred overnight, then the slurry was loaded directly onto a silica gel column and purified by flash chromatography (40% EtOAc in hexanes, then 5-15% MeOH in CH_2Cl_2). Concentration under vacuum yielded the semi-crude light brown solid (0.127 g, 25%) which was used in the next reaction without further purification. Characterization data match reported literature values.[5]

^{1}H NMR (400 MHz, CD_3OD) δ 6.57 (dt, J = 17.9, 4.1 Hz, 1H), 5.81 (dt, J = 17.8, 1.9 Hz, 1H), 4.12 (dd, J = 4.2, 1.9 Hz, 2H)

^{11}B NMR (128 MHz, CD_3OD) δ 27.6

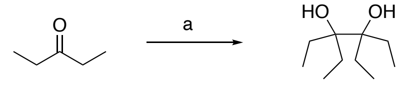

S4

Reagents and conditions
a) $TiCl_4$, THF, then Zn.

Scheme S8. Synthesis of Epin.

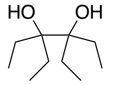

3,4-Biethylhexane-3,4-diol (S4)

A solution of 3-pentnone (2.14 g, 25.0 mmol) in THF (81 mL) in a three-neck flask was cooled to –60 °C. Titanium tetrachloride (7.13 g, 37.5 mmol) was added dropwise while nitrogen was flowing through one neck of the flask with an output in the other neck to remove the HCl vapor (*Caution*: *perform in a well-ventilated hood*). The reaction was stirred for 1 h, then zinc powder (6.54 g, 100 mmol,) was added in three portions. The mixture was warmed to rt and was then refluxed at 70 °C for 3 h. The mixture was cooled to 0 °C, then saturated potassium carbonate (50 mL) was added slowly and stirring was continued for 30 min. The crude slurry was filtered through a Celite plug and washed three times with EtOAc (10 mL). The filtrate was extracted three times with additional EtOAc (20 mL), dried with $MgSO_4$, filtered, and concentrated under reduced pressure. The crude mixture was purified by flash chromatography (10% EtOAc in hexanes with 0.5% triethylamine) to yield **S4** as a light-yellow oil (1.44 g, 33%). Characterization data match reported literature values.[6]

^{1}H NMR (400 MHz, $CDCl_3$) δ 1.61 (m, 8H), 0.94 (t, J = 7.5 Hz, 12H)

^{13}C NMR (100 MHz, $CDCl_3$) δ 78.8, 27.3, 9.0

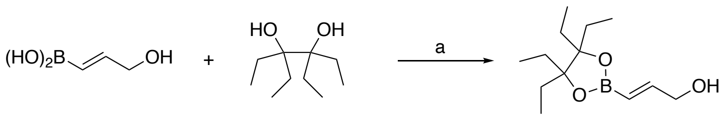

S3 **S_4** **S5**

Reagents and conditions
a) $MgSO_4$, CH_2Cl_2.

Scheme S9. Synthesis of Epin boronate.

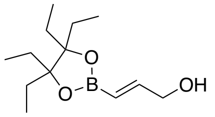

(*E*)-3-(4,4,5,5-Tetraethyl-1,3,2-dioxaborolan-2-yl)prop-2-en-1-ol (S5)

Diol **S4** (0.185 g, 1.10 mmol) and $MgSO_4$ (0.800 g) was added to a solution of boronic acid **S3** (0.325 g, 3.20 mmol) in CH_2Cl_2 (0.58 mL). The reaction was stirred at rt for 48 h. The mixture was loaded directly onto a silica gel column and purified by flash chromatography (10-20% EtOAc in hexanes) to yield **S5** as a light-yellow oil (0.187 g, 74%).

^{1}H NMR (500 MHz, $CDCl_3$) δ 6.73 (dt, *J* = 18.1, 4.2 Hz, 1H), 5.71 (dt, *J* = 18.1, 1.8 Hz, 1H), 4.22 (dd, *J* = 4.2, 1.9 Hz, 2H), 1.67 (m, 8H), 0.92 (t, *J* = 7.4 Hz, 12H)
^{13}C NMR (125 MHz, $CDCl_3$) δ 151.4, 88.4, 64.6, 26.4, 8.8
^{11}B NMR (160 MHz, $CDCl_3$) δ 29.4
HRMS (ESI) *m/z* calcd. for $C_{13}H_{26}BO_3$ $[M+H]^+$: 241.1970, found: 241.1964

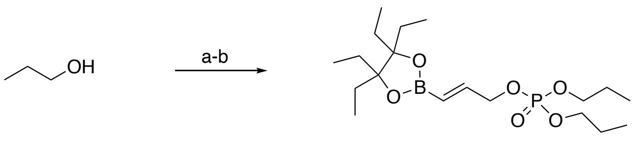

18

Reagents and conditions
a) $^{i}Pr_2NPCl_2$, Et_3N, THF, –78 °C. b) **S5**, $ImHClO_4$, CH_3CN, then $^{t}BuOOH$.

Scheme S10. Synthesis of **18**.

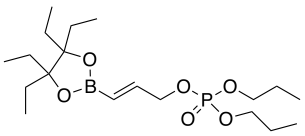

(*E*)-Dipropyl (3-(4,4,5,5-tetraethyl-1,3,2-dioxaborolan-2-yl)allyl) phosphate (18)

S1 (32 mg, 0.13 mmol) was dissolved in THF (0.5 mL) and stirred with 0.2 g 3Å MS for 5 min under argon. Imidazolium perchlorate (28 mg, 0.16 mmol) and **S5** (26 mg, 0.11 mmol) were dissolved in distilled CH_3CN (1.1 mL), and 0.4 g powdered 3Å MS was added. This solution was added dropwise to the phosphoramidite solution, and the mixture was stirred for 2 h. The mixture was cooled to 0 °C and $^{t}BuOOH$ in decane/toluene (0.5 M, 0.44 mL, 0.22 mmol) was added dropwise. After 20 minutes, the reaction was quenched with Me_2S (20 mg, 0.33 mmol). The solution was then filtered and concentrated. $^{t}BuOH$ was removed by azeotropic distillation with benzene, and the residual solvent was removed on a vacuum line. The crude material was purified by flash chromatography (15-20% EtOAc in hexanes) to yield **18** as a clear oil (49 mg, 100%).
^{1}H NMR (400 MHz, CD_3CN) δ 6.62 (dt, *J* = 17.9, 4.7 Hz, 1H), 5.77 (dt, *J* = 17.9, 1.7 Hz, 1H), 4.60 (m, 2H), 4.00 (q, *J* = 6.7 Hz, 4H), 1.68 (m, 12H), 0.95 (t, *J* = 7.4 Hz, 6H), 0.91 (t, *J* = 7.5 Hz, 12H)
^{13}C NMR (100 MHz, CD_3CN) δ 147.0 (d, *J* = 6.6 Hz), 89.0, 69.6 (d, *J* = 6.2 Hz), 68.6 (d, *J* = 5.5 Hz), 28.7, 23.9 (d, *J* = 6.6 Hz), 9.9, 8.7
^{11}B NMR (128 MHz, CD_3CN) δ 29.0
^{31}P NMR (162 MHz, CD_3CN) δ –0.75
HRMS (ESI) *m/z* calcd. for $C_{19}H_{39}BO_6P$ $[M+H]^+$ 405.2572, found 405.2570

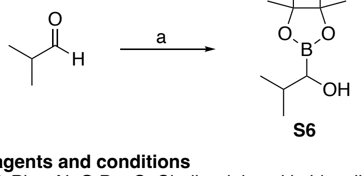

S6

Reagents and conditions
a) B_2Pin_2, NaOtBu, CuCl, dicyclohexyl imidazolium tetrafluoroborate, MeOH, PhMe.

Scheme S11. α-Boryl alcohol synthesis.

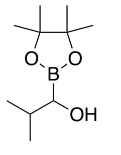

2-Methyl-1-(4,4,5,5-tetramethyl-1,3,2-dioxaborolan-2-yl)propan-1-ol (S6)

CuCl (0.138 g, 1.40 mmol), bis(pinacolato)diboron (1.95 g. 7.70 mmol,), 1,3-bis(cyclohexyl)-imidazolium tetrafluoroborate (0.244 g, 0.700 mmol), and sodium *t*-butoxide (0.135 g, 1.40 mmol) were dissolved in toluene (50 mL) and the mixture was stirred for 30 min. Isobutyraldehyde (0.51 g, 7.0 mmol) immediately followed by methanol (0.45 g, 14 mmol) were added to the reaction and stirring was continued for 90 min. The heterogenous mixture was filtered through a short silica plug pretreated with methylene chloride and washed with diethyl ether. The filtrate was concentrated and purified via column chromatography (5-10% Et_2O in CH_2Cl_2 then 20% EtOAc in hexanes) to give **S6** as a clear oil (0.994 g, 71%).

^{1}H NMR (400 MHz, $CDCl_3$) δ 3.37 (d, J = 4.6 Hz, 1H), 1.90 (m, 1H), 1.53 (br s, 1H), 1.28 (s, 6H), 1.26 (s, 6H), 0.97 (d, J = 8.0 Hz, 3H), 0.95 (d, J = 8.0 Hz, 3H)

^{13}C NMR (100 MHz, $CDCl_3$) δ 84.2, 32.4, 25.0, 24.9, 19.5, 18.6

^{11}B NMR (128 MHz, $CDCl_3$) δ 32.7

HRMS (ESI) *m/z* calcd. for $C_{20}H_{43}B_2O_6$ $[2M+H]^+$: 401.3240, found 401.3240

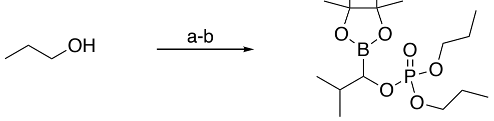

19

Reagents and conditions

a) ^{i}Pr$_2$NPCl$_2$, Et$_3$N, THF, 0 °C. b) **S6**, ImHClO$_4$, CH$_3$CN, then ^{t}BuOOH.

Scheme S12. Synthesis of **19**.

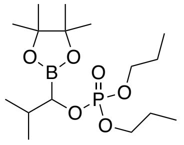

2-Methyl-1-(4,4,5,5-tetramethyl-1,3,2-dioxaborolan-2-yl)propyl dipropyl phosphate (19)

A solution of ^{i}Pr$_2$NPCl$_2$ (0.50 g, 2.5 mmol), triethylamine (0.53 g, 5.3 mmol), and THF (2.5 mL) was cooled to –10 °C. A solution of n-propanol (0.305 g, 5.07 mmol) in THF (0.9 mL) was added to the flask dropwise. The reaction was warmed to rt and stirred for 2 h. The heterogeneous mixture was filtered under N_2 and the salt was washed twice with THF (3.0 mL). The crude phosphoramidite was concentrated and used immediately in the next step to avoid unwanted decomposition.

Imidazolium perchlorate (0.131 g, 0.778 mmol) was dissolved in a solution of the crude phosphoramidite (0.194 g, 0.778 mmol) in CH_3CN (2.0 mL). A solution of CH_3CN (1.0 mL) and boronate **S6** (0.104 g, 0.519 mmol) was added and the reaction was stirred for 2 h. The mixture was cooled to –10 °C and *t*-butyl hydroperoxide (5.0-6.0 M solution in decane, 0.28 mL, 1.6 mmol) was added. The reaction was stirred for 10 min then quenched with Me_2S (0.13 g, 2.1 mmol). The mixture was diluted with ethyl acetate (10 mL) and washed with brine (5.0 mL) twice. The organic layer was dried with $MgSO_4$, filtered, and concentrated. ^{1}H NMR showed a yield of 30% relative to the internal standard toluene. The crude mixture was purified via column chromatography (1-5% MeOH in CH_2Cl_2) to yield the desired phosphate as a clear oil (0.032 g, 17%).

^{1}H NMR (500 MHz, CD_3CN) δ 4.00-3.90 (overlapping m, 4H), 3.85 (dd, J = 7.7, 5.9 Hz, 1H), 1.96 (m, overlaps solvent, 1H), 1.66 (m, 4H), 1.26 (s, 6H), 1.25 (s, 6H), 0.97-0.91 (overlapping m, 12H)

^{13}C NMR (125 MHz, CD_3CN) δ 85.2, 69.74 (d. J = 6.4 Hz), 69.71 (d, J = 6.4 Hz), 32.1 (d, J = 6.1 Hz), 25.1, 25.0, 24.43 (d, J = 6.4 Hz), 24.41 (d, J = 6.6 Hz), 19.5, 19.2, 10.4, 10.3

^{11}B NMR (160 MHz, CD_3CN) δ 31.0

^{31}**P NMR** (202 MHz, CD_3CN) δ –0.14
HRMS (ESI) *m/z* calcd. for $C_{16}H_{35}O_6BP$ $[M+H]^+$ 365.2259, found 365.2254

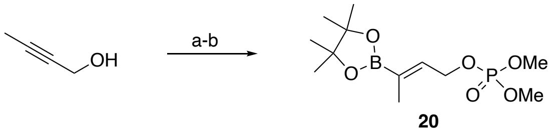

Reagents and conditions
a) B_2Pin_2, NaO^tBu, CuCl, PCy_3, PhMe, 44%. b) $(MeO)_2P(O)Cl$, Pyr., DMAP, CH_2Cl_2.

Scheme S13. Synthesis of **20**.

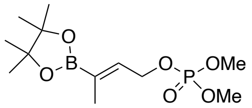

(*Z*)-Dimethyl (3-(4,4,5,5-tetramethyl-1,3,2-dioxaborolan-2-yl)but-2-en-1-yl) phosphate (20)

PCy_3 (0.240 g, 0.856 mmol) in toluene (0.07 mL) was added to a flask charged with CuCl (0.071 g, 0.71 mmol), sodium *t*-butoxide (0.103 g, 1.07 mmol), B_2Pin_2 (2.17 g, 8.56 mmol), butynol (0.50 g, 7.1 mmol,) and toluene (13 mL). The reaction was stirred overnight then was quenched with methanol (0.46 g, 14 mmol). The heterogenous mixture was filtered through a Celite pad that was pretreated with DCM. The crude mixture was concentrated and purified via column chromatography (15-35% *t*-butyl methyl ether in CH_2Cl_2) to give the desired product **S7** as a clear oil (0.628 g, 44%). Characterization data match reported literature values.[7]

S7

^{1}**H NMR** ($CDCl_3$, 500 MHz) δ 6.42 (td, *J* = 5.9, 1.5 Hz, 1H), 4.30 (dd, *J* = 5.9, 0.8 Hz, 2H), 1.70 (s, 3H), 1.26 (s, 12H)
^{13}**C NMR** ($CDCl_3$, 125 MHz) δ 144.0, 83.6, 60.0, 24.9, 14.3
^{11}**B NMR** ($CDCl_3$, 160 MHz) δ 30.3

Pyridine (0.12 g, 1.5 mmol) and DMAP (0.037 g, 0.30 mmol) were added to a solution of the allylic alcohol (0.20 g, 1.0 mmol,) in CH_2Cl_2 (2 mL). The mixture was cooled to 0 °C and $(MeO)_2P(O)Cl$ (0.43 g, 3.0 mmol) was added slowly. The reaction was warmed to rt and stirred overnight. After diluting with EtOAc (10 mL), the reaction was quenched with saturated ammonium chloride and the aqueous layer was extracted twice with EtOAc. The organic layer was washed with brine, dried with Na_2SO_4, filtered, and concentrated. The crude mixture was purified by column chromatography (20-40% EtOAc in hexanes) and the residual solvent was removed on a vacuum line for 1 hour at 60 °C to give the desired clear oil (0.061 g, 20%).

^{1}**H NMR** (400 MHz, CD_3CN) δ 6.26 (t, *J* = 6.0 Hz, 1H), 4.66 (dd, *J* = 6.2, 0.9 Hz, 1H), 4.64 (dd, *J* = 6.2, 0.9 Hz, 1H), 3.71 (s, 3H), 3.68 (s, 3H), 1.68 (s, 3H), 1.24 (s, 12H)
^{13}**C NMR** (125 MHz, CD_3CN) δ 139.1 (d, *J* = 6.3 Hz), 84.2, 64.4 (d, *J* = 5.5 Hz), 54.5 (d, *J* = 5.5 Hz), 24.7, 14.0
^{11}**B NMR** (160 MHz, CD_3CN) δ 29.9
^{31}**P NMR** (162 MHz, CD_3CN) δ 1.44
HRMS (ESI) *m/z* calcd. for $C_{12}H_{25}O_6BP$ $[M+H]^+$ 307.1476, found 307.1487

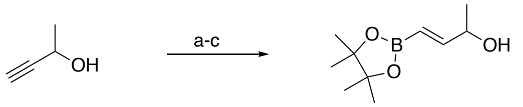

S8

Reagents and conditions
a) TBSCl, imidazole, DMAP, CH_2Cl_2, 0 °C to rt. b) HBPin, Et_3N, $Cp_2Zr(H)Cl$, 70 °C, 78% (two steps). c) CSA, MeOH, CH_2Cl_2, 0 °C to rt, 26%.

Scheme S14. Synthesis of **S8**.

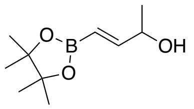

(*E*)-4-(4,4,5,5-Tetramethyl-1,3,2-dioxaborolan-2-yl)but-3-en-2-ol (S8)

But-3-yn-2-ol (0.84 mL, 12 mmol) was added to a solution of imidazole (2.04 g, 30.0 mmol) and DMAP (0.293 g, 2.40 mmol) in CH_2Cl_2 (120 mL). The mixture was cooled to 0 °C and TBSCl (2.71 g, 18.0 mmol) was added. After stirring for 1 h the reaction was quenched with saturated ammonium chloride (aqueous, 100 mL), extracted twice with methylene chloride (100 mL), dried over sodium sulfate, and filtered. After concentrating the crude liquid the alkyne was purified via column chromatography (0-20% EtOAc in hexanes) to give the desired pale-yellow liquid (2.471 g). Pinacol borane (1.61 g, 12.6 mmol) was added carefully (gas evolution) to a mixture of the silyl ether (2.471 g), $Cp_2Zr(H)Cl$ (0.309 g, 1.20 mmol), and triethylamine (0.12 g, 1.2 mmol). The reaction was heated to 70 °C and stirred overnight. The mixture was diluted with 5% EtOAc in hexanes, filtered, and loaded directly onto a column for purification by flash chromatography (5-10% EtOAc in hexanes) to give the desired clear oil (2.933 g, 78% over two steps). A solution of the boronate (2.932 g, 9.391 mmol) in 1:1 (by volume) methylene chloride and methanol (63 mL) was cooled to 0 °C. Camphorsulfonic acid (0.218 g, 0.939 mmol) was added, and the reaction stirred for 1 h at rt. The mixture was concentrated and purified by column chromatography (5-10% EtOAc in hexanes) to give the desired clear oil (0.486 g, 26%).

^{1}H NMR (500 MHz, $CDCl_3$) δ 6.62 (dd, J = 18.1, 5.0 Hz, 1H), 5.59 (dd, J = 18.1, 1.5 Hz), 4.31 (m, 1H), 1.88 (br s, 1H), 1.25 (two overlapping s, 15 H)

^{13}C NMR (125 MHz, $CDCl_3$) δ 156.4, 83.5, 69.7, 24.9, 22.8

^{11}B NMR (160 MHz, $CDCl_3$) δ 29.9

HRMS (ESI) *m/z* calcd. for $C_{10}H_{18}O_3B$ $[M{-}H]^-$ 197.1344, found 197.1348

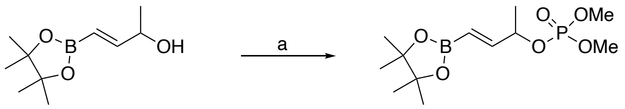

S8 **22**

Reagents and conditions
a) $(MeO)_2P(O)Cl$, Pyr., DMAP, CH_2Cl_2.

Scheme S15. Synthesis of **22**.

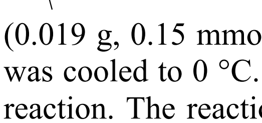

(*E*)-Dimethyl (4-(4,4,5,5-tetramethyl-1,3,2-dioxaborolan-2-yl)but-3-en-2-yl) phosphate (22)

Allylic alcohol **S8** (0.100 g, 0.505 mmol) was added to a solution of DMAP (0.019 g, 0.15 mmol) and pyridine (0.061 mL, 0.76 mmol) in CH_2Cl_2 (1.0 mL) and the mixture was cooled to 0 °C. Dimethyl chlorophosphate (0.220 g, 1.52 mmol) was added dropwise to the reaction. The reaction was warmed to rt and was stirred overnight. The reaction was quenched with saturated ammonium chloride (5.0 mL) and extracted twice with ethyl acetate (10 mL). The organic layer was washed with brine (10 mL), then dried with sodium sulfate, filtered, and

concentrated to give the desired product as a clear oil (49 mg, 29%) without the need for further purification.

^{1}H NMR (500 MHz, $CDCl_3$) δ 6.54 (dd, *J* = 18.2, 4.8 Hz, 1H), 5.66 (d, *J* = 18.0 Hz, 1H), 4.94 (m, 1H), 3.73 (m, 3H), 3.71 (m, 3H), 1.40 (d, *J* = 6.5 Hz, 3H), 1.24 (s, 12H)

^{13}C NMR (126 MHz, $CDCl_3$) δ 151.0 (d, *J* = 6.2 Hz), 83.4, 76.0 (d, *J* = 5.5 Hz), 54.2 (d, *J* = 6.4 Hz), 54.1 (d, *J* = 7.0 Hz), 21.5 (d, *J* = 3.1 Hz)

^{11}B NMR (160 MHz, $CDCl_3$) δ 29.7

^{31}P NMR (202 MHz, $CDCl_3$) δ 0.41

HRMS (ESI) *m/z* calcd. for $C_{12}H_{25}O_6BP$ $[M+H]^+$ 307.1476, found 307.1480

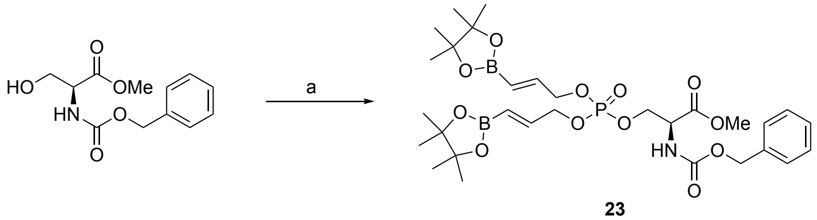

23

Reagents and conditions
a) **S2**, $ImHClO_4$, CH_3CN, then *t*BuOOH.

Scheme S16. Synthesis of **23**.

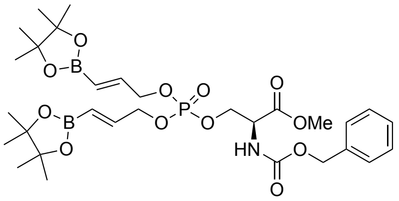

***N*-Cbz Serine methyl ester phosphate precursor (23)**

To imidazolium perchlorate (0.131 g, 0.776 mmol), Cbz-L-serine methyl ester (0.164 g, 0.647 mmol), CH_3CN (1.3 mL) and powdered molecular sieves (4 Å, 0.150 g) was added a solution of phosphoramidite **S2** (0.386 g, 0.776 mmol) in CH_3CN (1.3 mL). The reaction was stirred for 2 h. The mixture was cooled to 0 °C and *t*-butyl hydroperoxide (0.5 M in toluene, 3.88 mL, ~1.94 mmol) was added. The reaction was stirred for 10 min and then quenched with Me_2S (0.121 g, 1.94 mmol). The mixture was concentrated, diluted with CH_2Cl_2 (10 mL), and was washed with brine (10 mL) twice. The organic layer was dried with $MgSO_4$, filtered, and concentrated *in vacuo*. The crude mixture was purified via column chromatography (3-5% CH_3OH in CH_2Cl_2). The isolated product was dissolved in CH_2Cl_2 and quickly flushed through a short SiO_2 plug (prewashed with CH_2Cl_2) to remove post-column boric acid to yield the pure phosphate (0.175 g, 41%) as a colorless oil.

^{1}H NMR (400 MHz, CD_3CN) δ 7.44-7.33 (m, 5H), 6.54 (dt, *J* = 18.3, 4.4 Hz, 2H), 6.39 (br d, *J* = 8.2 Hz, 1H), 5.70 (app d, *J* = 18.0 Hz, 2H), 5.13 (s, 2H), 4.63-4.50 (m, 5H), 4.43-4.35 (m, 1H), 4.35-4.27 (m, 1H), 3.75 (s, 3H), 1.26 (s, 24H)

^{13}C NMR (100 MHz, CD_3CN) δ 170.1, 156.5, 146.4 (d, *J* = 6.7 Hz), 137.5, 129.1, 128.6, 128.4, 84.0, 69.1 (d, *J* = 5.1 Hz), 67.6 (d, *J* = 5.5 Hz), 67.0, 55.0, 52.9, 24.7

^{11}B NMR (128 MHz, CD_3CN) δ 29.7

^{31}P NMR (162 MHz, CD_3CN) δ –1.0

HRMS (ESI) *m/z* calcd. for $C_{30}H_{47}O_{12}NB_2P$ $[M+H]^+$ 666.3017, found 666.3026

$[\alpha]_D^{22}$ –6.3 (*c* 1.0, DMSO)

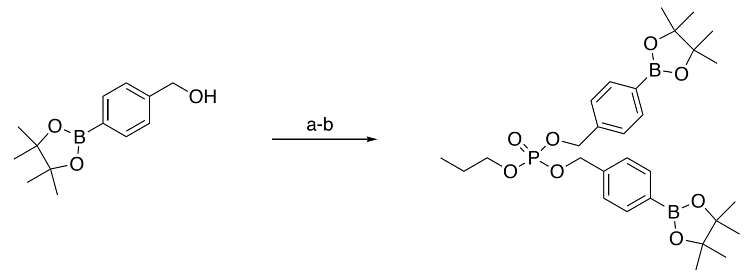

S10

Reagents and conditions

a) $^{i}Pr_2NPCl_2$, Et_3N, THF, 0 °C. b) $^{n}PrOH$, $ImHClO_4$, CH_3CN, then $^{t}BuOOH$.

Scheme S17. Synthesis of **S10**.

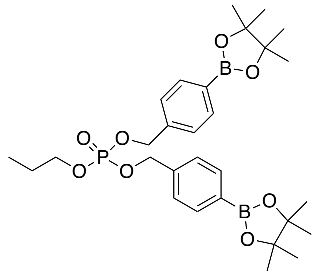

Propyl bis(4-(4,4,5,5-tetramethyl-1,3,2-dioxaborolan-2-yl)benzyl) phosphate (S10)

A solution of $^{i}Pr_2NPCl_2$ (0.41 mL, 2.0 mmol) in THF (3 mL) was cooled to –78 °C. A solution of borylated benzyl alcohol (0.91 g, 4.0 mmol) and triethylamine (0.42 g, 4.2 mmol) in THF (6.3 mL) was added dropwise, then the solution was warmed to rt and stirred overnight. The mixture was filtered and the salt was washed twice with THF (5.0 mL). The filtrate was concentrated in vacuo, and the crude product was purified by flash chromatography (15% EtOAc in hexanes with 1% triethylamine) to yield the intermediate phosphoramidite **S9** as a white solid (0.714 g, 60%).

S9

^{1}H NMR (400 MHz, $CDCl_3$) δ 7.70 (d, *J* = 8.0 Hz, 4H), 7.38 (d, *J* = 8.0 Hz, 4H), 4.76 (m, 4H), 3.72 (m, 2H), 1.35 (s, 24H), 1.22 (d, *J* = 6.9 Hz, 12H)

^{13}C NMR (100 MHz, $CDCl_3$) δ 143.6 (d, *J* = 7.4), 135.0, 126.9, 84.4, 65.6 (d, *J* = 18.2), 43.5 (d, *J* = 12.4), 24.8, 24.5 (d, *J* = 7.3)

^{11}B NMR (128 MHz, $CDCl_3$) δ 31.1

^{31}P NMR (162 MHz, $CDCl_3$) δ 148.1

HRMS (ESI) *m/z* calcd. for $C_{32}H_{51}B_2NO_6P$ $[M+H]^+$ 598.3635, found 598.3656

Phosphoramidite **S9** (0.51 g, 0.85 mmol) was dissolved in THF (3 mL) and stirred with 0.5 g 3Å molecular sieves for five min under argon. Imidazolium perchlorate (0.17 g, 1.0 mmol) and n-PrOH (43 mg, 0.71 mmol) were dissolved in distilled CH_3CN (7 mL), and 0.75 g powdered 3Å molecular sieves was added. This solution was added dropwise to phosphoramidite solution, and the reaction was stirred for 2 h. The mixture was cooled to 0 °C and $^{t}BuOOH$ in decane/toluene (0.5 M, 2.12 mL, 1.06 mmol) was added dropwise. After 20 min, the reaction was quenched with Me_2S (0.11 g, 1.8 mmol). The solution was filtered and concentrated. $^{t}BuOH$ was removed by azeotropic distillation with benzene, and the residual solvent was removed on a vacuum line for 3 h. The product was purified by flash chromatography (5% MeOH in CH_2Cl_2) to yield **S10** as a white solid (20 mg, 4.1%).

^{1}H NMR (400 MHz, $CDCl_3$) δ 7.73 (d, *J* = 7.9 Hz, 4H), 7.37 (d, *J* = 7.9 Hz, 4H), 5.06 (d, *J* = 8.4 Hz, 4H), 3.98 (dt, *J* = 7.0, 6.6 Hz, 2H), 1.65 (m, 2H), 1.35 (s, 24 H), 0.92 (t, *J* = 7.4 Hz, 3H)

^{13}C NMR (MHz, $CDCl_3$) δ 140.1 (d, *J* = 6.4 Hz), 135.2, 127.7, 84.51, 69.9 (d, *J* = 5.4 Hz), 69.2 (d, *J* = 5.6 Hz), 24.8, 23.9 (d, *J* = 6.9 Hz), 9.8

^{11}B NMR (128 MHz, $CDCl_3$) δ 30.7

^{31}P NMR (162 MHz, $CDCl_3$) δ –0.90

HRMS (ESI) *m/z* calcd. for $C_{29}H_{44}B_2O_8P$ $[M+H]^+$ 573.2954, found 573.2943

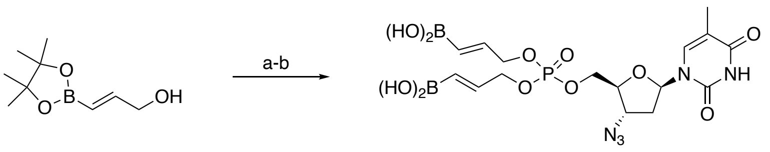

25

Reagents and conditions

a) $^{i}Pr_2NPCl_2$, Et_3N, THF. b) AZT, 1-H-tetrazole, CH_3CN, then $^{t}BuOOH$, then pH = 7.4 phosphate buffer.

Scheme S18. Synthesis of **25**.

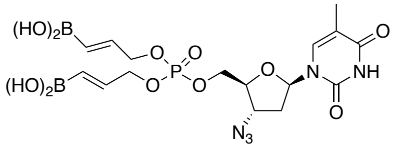

AZT Nucleotide precursor 25

A solution of $^{i}Pr_2NPCl_2$ (0.27 g, 1.3 mmol) in THF (3.5 mL) was cooled to –78 °C. A solution of boronate **6** (0.480 g, 2.61 mmol) and triethylamine (0.57 g, 5.6 mmol) in THF (7.5 mL) was added to the flask dropwise over 1 h. The reaction was slowly warmed to rt and stirred overnight. The heterogeneous mixture was filtered under N_2 and the salt was washed twice with THF (3.0 mL). The crude phosphoramidite was concentrated *in vacuo* then purified via column chromatography (14% EtOAc in cyclohexane with 1% triethylamine) to yield the desired product as a clear oil (0.315 g, 49%).

The phosphoramidite (0.315 g, 0.634 mmol) and azidothymidine (0.085 g, 0.32 mmol,) were dissolved in a solution of 1H-tetrazole (0.45 M in CH_3CN, 1.40 mL, 0.634 mmol) in CH_3CN (1.8 mL). The reaction was stirred at rt for 1 h. The mixture was then cooled to 0 °C and *t*-butyl hydroperoxide (0.5 M in toluene and decanes, 1.90 mL, 0.951 mmol) was added dropwise. After stirring for 10 min at rt, the reaction was quenched with Me_2S (0.059 g, 0.95 mmol). After five min, the stirring was stopped and the mixture was filtered through a cotton plug. The precipitate was washed with cooled CH_3CN then the resulting solution concentrated. ^1H NMR showed a yield of 58% relative to the internal standard 1,2-dimethoxyethane. The mixture was then stirred in a solution (2.0 mL) of 20% CH_3CN in phosphate buffer (pH = 7.4) overnight. For kinetics, the crude mixture was rigorously purified using two rounds of automated reverse phase column chromatography (10-30% CH_3CN in H_2O + 0.1% TFA over 11 minutes then 10-20-100% CH_3CN in H_2O + 0.1% TFA over 14 minutes) then lyophilized to remove water to yield a white solid (0.0065 g, 4%).

^{1}H NMR (500 MHz, D_2O) δ 7.51 (d, *J* = 1.1 Hz, 1H), 6.53 (app q, *J* = 5.0 Hz, 1H), 6.50 (app q, *J* = 4.8 Hz, 1H), 5.77 (dt, *J* = 3.2, 1.6 Hz, 1H), 5.73 (dt, *J* = 3.1, 1.7 Hz, 1H), 4.72 (m, overlapping with H_2O signal, 6H), 4.47 (m, 2H), 4.39 (m, 1H), 4.20 (m, 1H), 2.56 (m, 2H), 1.88 (d, *J* = 0.95 Hz, 3H)

^{13}C NMR (151 MHz, D_2O) δ 166.3, 142.9 (overlapping d, *J* = 5.6 Hz), 137.3, 85.3, 82.0 (d, *J* = 7.6 Hz), 69.8 (overlapping d, *J* = 5.8 Hz), 66.9, 59.4, 36.0, 11.7

^{11}B NMR (160 MHz, D_2O) δ 27.3

^{31}P NMR (202 MHz, D_2O) δ –0.71

HRMS (ESI) *m/z* calcd. for $C_{16}H_{25}O_{11}N_5B_2P$ $[M+H]^+$ 516.1469, found 516.1473

$[\alpha]_D^{25}$ 12.4 (*c* = 0.17, H_2O)

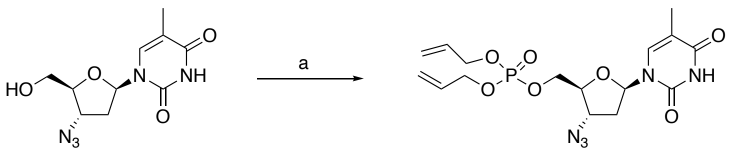

27

Reagents and conditions
a) PCl_3, Pyr., then allyl alcohol, then H_2O_2.

Scheme S19. Synthesis of negative control compound **27**.

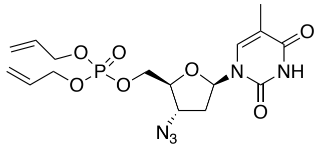

Diallyl (((2*S*,3*S*,5*R*)-3-azido-5-(5-methyl-2,4-dioxo-3,4-dihydropyrimidin-1(2*H*)-yl)tetrahydrofuran-2-yl)methyl) phosphate (27)

Phosphorus trichloride (0.455 g, 3.28 mmol) was added to a solution of azidothymidine (0.250 g, 0.936 mmol) in pyridine (50 mL). The reaction was stirred for 15 min, then allyl alcohol (0.47 g, 8.1 mmol) was added and stirring was continued for another 60 min. The reaction was diluted with methylene chloride (125 mL) and then hydrogen peroxide (30% aqueous solution in water, 0.33 mL, 3.3 mmol) was added. After 30 min, the reaction was quenched with aqueous sodium bicarbonate (250 mL), extracted twice with dichloromethane (250 mL), and dried with sodium sulfate. After filtering then concentrating, the crude mixture was purified via column chromatography (20-50% CH_3CN in CH_2Cl_2) to give the desired phosphate as a clear oil (0.043 g, 11%).

^{1}H NMR (500 MHz, CD_3CN) δ 9.14 (br s, 1H), 7.37 (d, *J* = 0.6 Hz, 1H), 6.14 (t, *J* = 6.6 Hz, 1H), 6.00 (dd, *J* = 10.7, 5.5 Hz, 1H), 5.95 (dd, *J* = 10.6, 5.5 Hz, 1H), 5.35 (app q, *J* = 1.5 Hz, 1H), 5.31 (app q, *J* = 1.5 Hz, 1H), 5.25 (app d, *J* = 10.5 Hz, 2H), 4.54 (m, 4H), 4.36 (q, *J* = 6.0 Hz, 1H), 4.23 (m, 2H), 4.00 (m, 1H), 2.37 (app td, *J* = 6.6, 1.0 Hz, 2H), 1.83 (d, *J* = 1.1 Hz, 3H)

^{13}C NMR (125 MHz, CD_3CN) δ 164.2, 150.9, 136.3, 133.1 (d, *J* = 6.6 Hz), 118.2 (d, *J* = 1.8 Hz), 111.2, 85.0, 82.5 (d, *J* = 7.3 Hz), 68.8 (d, *J* = 2.7 Hz), 68.7 (d, *J* = 2.2 Hz), 66.9 (d, *J* = 5.4 Hz), 60.6, 37.1, 12.1

^{31}P NMR (202 MHz, CD_3CN) δ –0.94

HRMS (ESI) *m/z* calcd. for $C_{16}H_{23}O_7N_5P$ $[M+H]^+$ 428.1330, found 428.1309

$[\alpha]_D^{25}$ 41.7 (*c* = 0.84, CH_3CN)

Phosphate Release Studies

General Procedure for Monitoring Monophosphate Release via ^{1}H NMR Spectroscopy.
The NMR spectrometer (600 MHz) was programmed to acquire a spectrum every 32 scans (2 minutes, 5 seconds) with a relaxation time of 7 seconds and locked to CD_3CN. Buffer solutions were prepared using D_2O according to Gomori[8] and the pH was monitored before each use. Each sample was prepared with toluene (5 μL, 0.0075 mmol) or 1,2-dimethoxyethane (5 μL, 0.007705 mmol) as the internal standard where both were prepared as a 1.5 M solution in CD_3CN. All experiments were run at 37 °C and the final concentration of substrate was 0.002 M and of hydrogen peroxide was 0.06 M in a mixture of CD_3CN and D_2O.

Representative procedure for Sample Preparation and Phosphate Release in the Presence of H_2O_2.
Monophosphate **7** (0.57 mg, 0.0016 mmol) was added to CD_3CN (500 μL) and toluene (internal standard, 5 μL) in an NMR tube. An initial ^{1}H NMR spectrum was taken to obtain baseline integration values relative to the internal standard. A solution of pH 7.4 buffer (50 μL, 0.1 M in D_2O) and D_2O (110 μL) was added to the NMR tube and a second 1H NMR spectrum was taken to observe hydrolysis of the boronate ester to the boronic acid without undesired release. A solution of H_2O_2•urea (1.4 M, 36 μL, 30.0 equiv) was added to the NMR tube and the reaction was mixed by inversion three times. Release of the phosphate was then monitored by ^{1}H NMR spectroscopy over time, using the integration values to calculate quantitative amounts of product formation and starting material consumption. This preparation is modeled after Hanna's[9] protocol.

Characterization Data for Released Products

Phosphates **13** (Acros Organics), **21** (Acros Organics), and **26** (Santa Cruz Biotechnology, Inc.) were purchased and dissolved in the same solvents that were used for the oxidative cleavage studies for collection of characterization data.

For characterization of **11** and **15**, compounds **8** and **14**, respectively, were subjected to ^{1}H NMR-scale oxidative cleavage conditions and characterized *in situ* after quenching excess hydrogen peroxide with sodium thiosulfate due to challenges with isolation of the products. Both have dimethoxyethane or toluene (internal standard) and acrolein-derived byproducts present in addition to the deuterated solvent mixture used during kinetic analysis.

The remainder of the phosphates were characterized by subjecting a small amount of caged compound to oxidative cleavage conditions in a flask reaction and isolating the desired products via reverse-phase column chromatography (RP-ISCO). This includes **9**, **17**, and **24**. Yields were not optimized since the objective was the formation of spectroscopic standards.

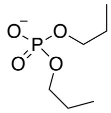

Dipropyl phosphate (9)

Product isolated as a white solid (0.0016 g, 1.5%) from oxidative cleavage of compound **7** (0.204 g, 0.586 mmol).

^{1}H NMR (500 MHz, CD_3CN, phosphate buffer in D_2O) δ 3.70 (q, *J* = 6.6 Hz, 4H), 1.55 (sextet, *J* = 7.1 Hz, 4H), 0.86 (t, *J* = 7.4 Hz, 6H)

^{13}C NMR (125 MHz, CD_3CN) δ 67.7 (d, *J* = 6.8 Hz), 29.3 (d, *J* = 7.3 Hz), 10.1

^{31}P NMR (202 MHz, CD_3CN) δ 1.47

HRMS (ESI) *m/z* calcd. for $C_6H_{16}O_4P$ $[M+H]^+$ 183.0781, found 183.0782

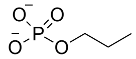

Monopropyl phosphate (11)

Product characterized under oxidative cleavage conditions and not isolated from solution (NMR Yield = 95%).

^{1}H NMR (400 MHz, CD_3CN, phosphate buffer in D_2O) δ 3.67 (app q, J = 6.6 Hz, 2H), 1.54 (sextet, *J* = 7.2 Hz, 2H), 0.86 (t, *J* = 7.7 Hz, 3H)

^{13}C NMR (176 MHz, CD_3CN, phosphate buffer in D_2O) δ 66.7 (d, *J* = 6.5 Hz), 23.6 (d, *J* = 7.3 Hz), 9.6

^{31}P NMR (162 MHz, CD_3CN:phosphate buffer in D_2O 60:40) δ 1.72

HRMS (ESI) *m/z* calcd. for $C_3H_8O_4P$ $[M-H]^-$ 139.0155, found 139.0161

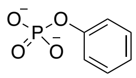

Phenyl phosphate (13)

Product purchased as a white solid.

^{1}H NMR (300 MHz, CD_3CN, phosphate buffer in D_2O) δ 7.26 (t, *J* = 7.8 Hz, 2H), 7.14 (dd, *J* = 7.6, 0.9 Hz, 2H), 7.00 (t, *J* = 7.3 Hz, 1H)

^{13}C NMR (75 MHz, D_2O) δ 153.8 (d, *J* = 6.2 Hz), 129.3, 122.5, 120.4 (d, *J* = 4.4 Hz)

^{31}P NMR (122 MHz, D_2O) δ –1.09

HRMS (ESI) *m/z* calcd. for $C_6H_8O_4P$ $[M+H]^+$ 175.0155, found 175.0160

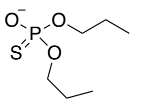

Dipropyl thiophosphate (15)
Product characterized under oxidative cleavage conditions and not isolated from solution (NMR Yield = 98%).
^{1}H NMR (400 MHz, CD_3CN, phosphate buffer in D_2O) δ 3.76 (m, 4H), 1.58 (sextet, *J* = 7.1 Hz, 4H), 0.88 (t, *J* = 7.2 Hz, 6H)
^{13}C NMR (151 MHz, phosphate buffer in D_2O) δ 67.3 (d, *J* = 6.2 Hz), 23.4 (d, *J* = 7.7 Hz), 9.7
^{31}P NMR (162 MHz, CD_3CN, phosphate buffer in D_2O) δ 55.41
HRMS (ESI) *m/z* calcd. for $C_6H_{14}O_3PS$ $[M\text{-}H]^-$ 197.0396, found 197.0401

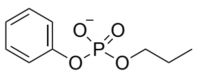

Phenyl propyl phosphate (17)
Product isolated as a white solid (0.018 g, 4%) from oxidative cleavage of compound **16** (0.895 g, 2.342 mmol).
^{1}H NMR (500 MHz, CD_3CN, phosphate buffer in D_2O) δ 7.30 (t, *J* = 7.9 Hz, 2H), 7.14 (d, *J* = 8.3 Hz, 2H), 7.08 (t, *J* = 7.7 Hz, 1H), 3.80 (q, *J* = 6.6 Hz, 2H), 1.54 (sextet, *J* = 7.1 Hz, 2H), 0.83 (t, *J* = 7.5 Hz, 3H
^{13}C NMR (101 MHz, CD_3CN) δ 162.2, 152.0 (d, *J* = 6.6 Hz), 129.7, 124.2, 120.2 (d, *J* = 4.2 Hz), 68.6 (d, *J* = 6.8 Hz), 23.4 (d, *J* = 7.3 Hz), 9.6
^{31}P NMR (202 MHz, CD_3CN) δ –3.99
HRMS (ESI) *m/z* calcd. for $C_9H_{14}O_4P$ $[M+H]^+$ 217.0624, found 217.0624

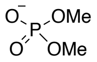

Dimethyl phosphate (21)
Product purchased as a colorless liquid.
^{1}H NMR (300 MHz, CD_3CN, phosphate buffer in D_2O) δ 3.48 (d, *J* = 10.6 Hz, 6H)
^{13}C NMR (76 MHz, D_2O) δ 53.2 (d, *J* = 6.0 Hz)
^{31}P NMR (122 MHz, CD_3CN) δ –1.11
HRMS (ESI) *m/z* calcd. for $C_2H_8O_4P$ $[M+H]^+$ 127.0155, found 127.0160

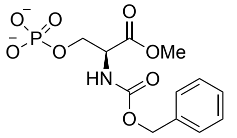

Cbz-Serine monophosphate (24)
Product isolated as a white solid (0.0019 g, 11%) from oxidative cleavage of compound **23** (0.032 g, 0.049 mmol).
^{1}H NMR (500 MHz, $CD_3CN:D_2O:(CD_3)_2SO$ 60:35:5) δ 7.37-7.30 (m, 5H), 5.08 (d, J = 25.9 Hz, 1H), 5.05 (d, *J* = 25.9 Hz, 1H), 4.44-4.40 (m, 1H), 4.23-4.19 (m, 1H), 4.12-4.09 (m, 1H), 3.69 (s, 3H)
^{13}C NMR (126 MHz, $CD_3CN:D_2O:(CD_3)_2SO$ 60:35:5) δ 171.4, 157.4, 137.2, 129.1, 128.7, 128.3, 67.2, 66.0 (d, *J* = 4.0 Hz), 55.1 (d, *J* = 7.3 Hz), 53.2
^{31}P NMR (202 MHz, $CD_3CN:D_2O:(CD_3)_2SO$ 60:35:5) δ –0.76
HRMS (ESI) *m/z* calcd. for $C_{12}H_{17}NO_8P$ $[M+H]^+$ 334.0686, found 334.0695
$[\alpha]_D^{25}$ –8.8 (*c* 1.7, H_2O)

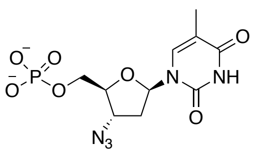

AZT monophosphate (26)
Product purchased as a white solid.
^{1}H NMR (400 MHz, CD_3CN, phosphate buffer in D_2O) δ 7.72 (s, 1H), 6.17 (t, *J* = 6.8 Hz, 1H), 4.45 (m, 1H), 2.46-2.28 (m, 3H), 2.10 (m, 2H), 1.85 (s, 3H), 1.11 (t, *J* = 7.0 Hz, 1H)
^{13}C NMR (101 MHz, $(CD_3)_2SO$) δ 163.9, 149.3, 135.3, 109.4, 82.6, 81.1 (d, *J* = 8.9 Hz), 62.4 (d, *J* = 4.3 Hz), 58.9 (d, *J* = 4.6 Hz), 55.1, 34.3, 9.7

^{31}P NMR (162 MHz, CD_3CN) δ 1.01
HRMS (ESI) *m/z* calcd. for $C_{10}H_{15}N_5O_7P$ $[M+H]^+$ 348.0704, found 348.0712
$[\alpha]_D^{25}$ 23.5 (c = 0.63, H_2O)

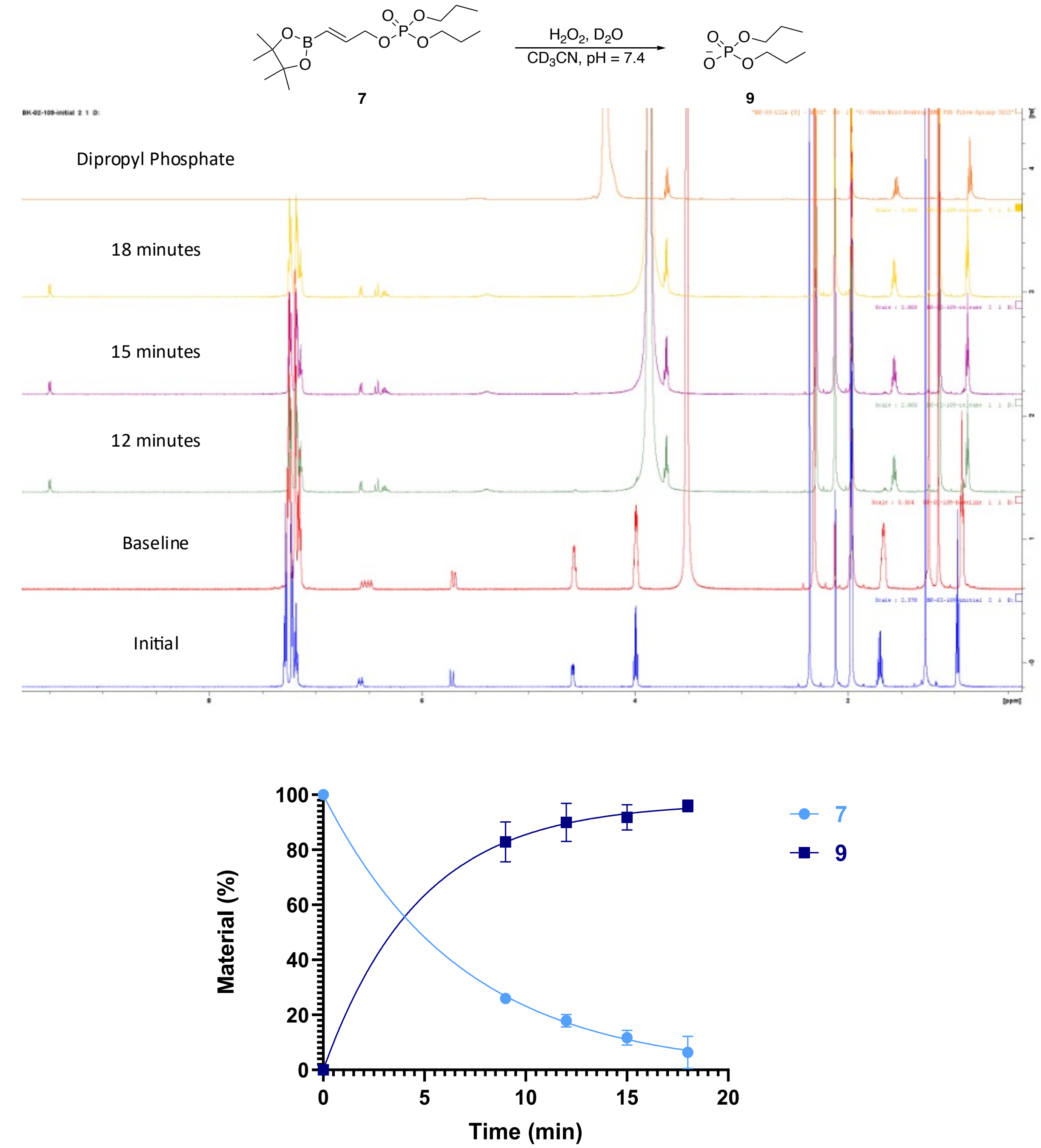

Figure S1. Oxidative cleavage of phosphate **7**.

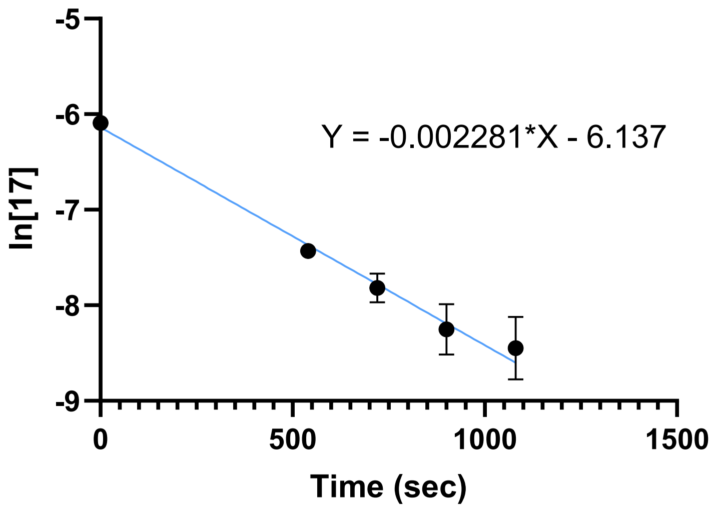

Figure S2. Rate constant determination for the oxidative cleavage of **7**.

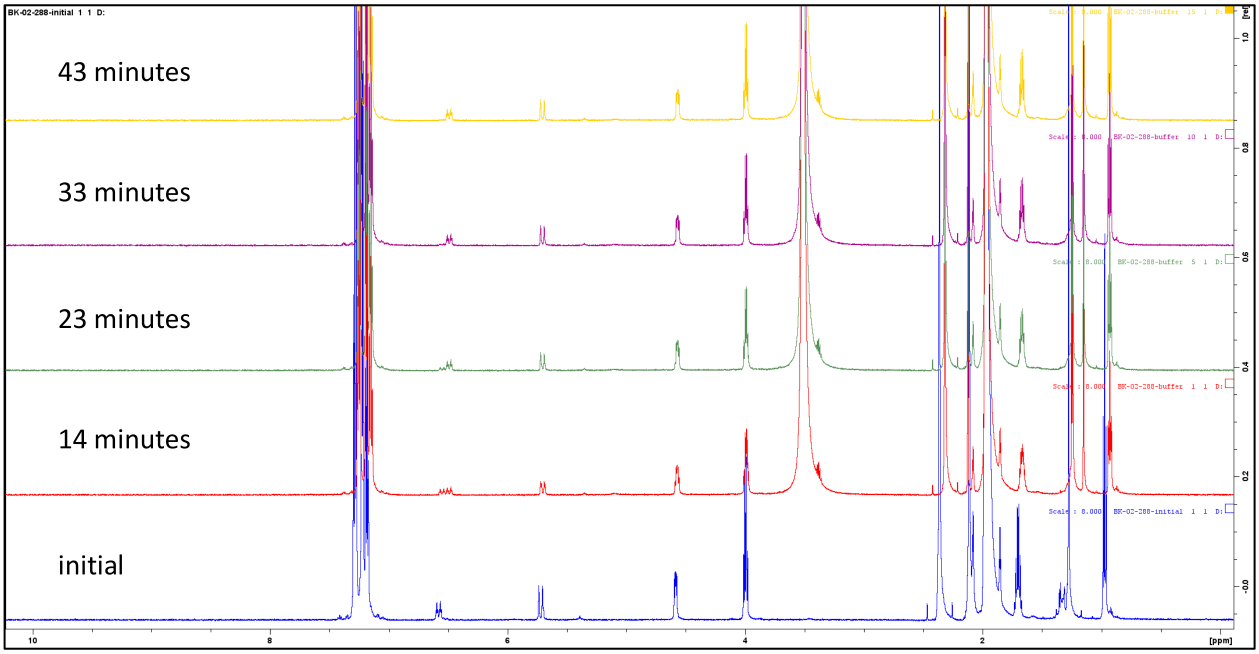

Figure S3. Phosphate **7** in aqueous buffer but in the absence of H_2O_2.

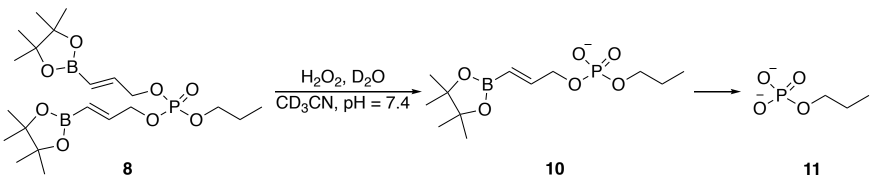

8 **10** **11**

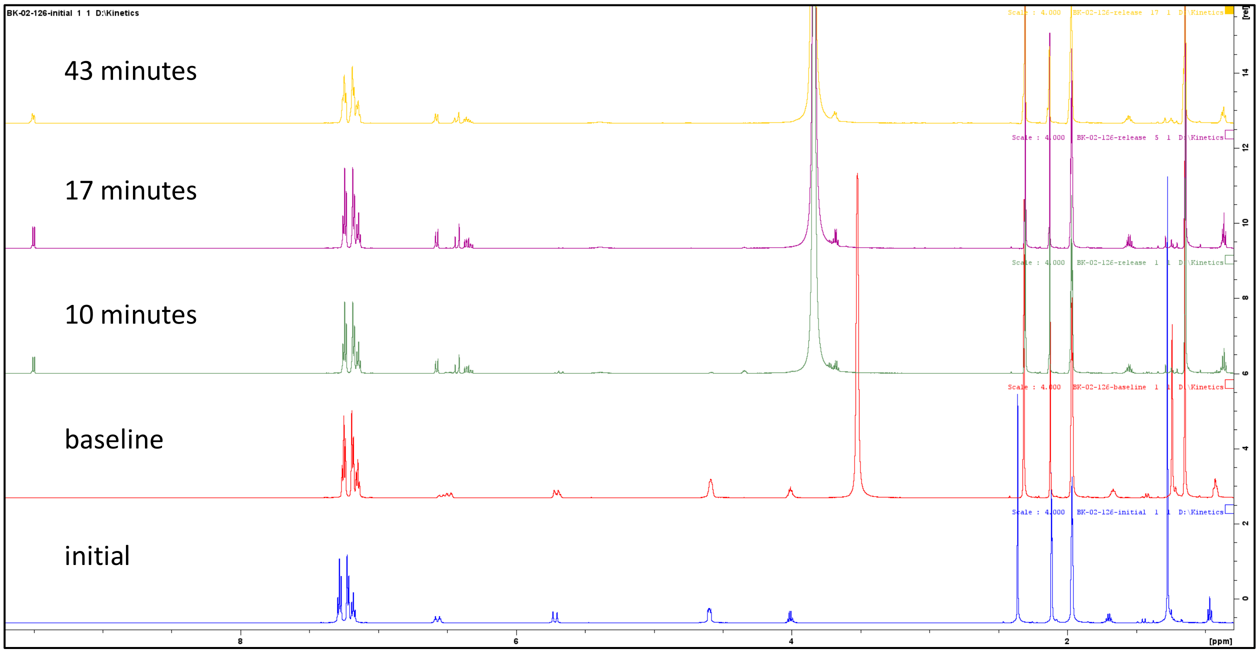

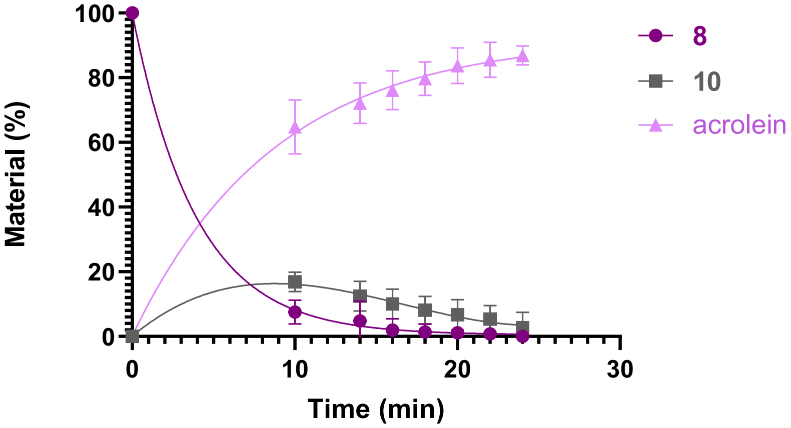

Figure S4. Oxidative cleavage of phosphate **8**.

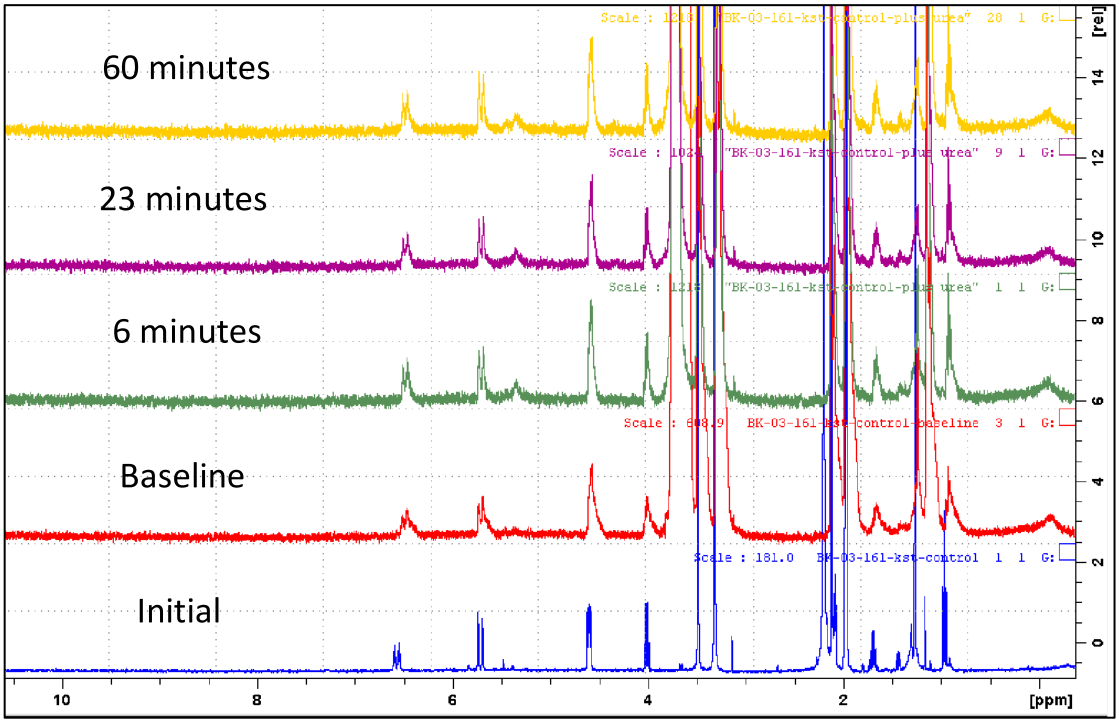

Figure S5. Treatment of **8** with urea but not H_2O_2.

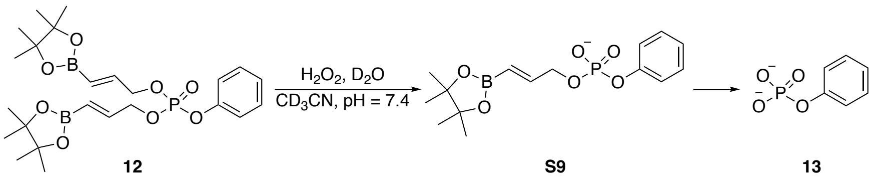

12 **S9** **13**

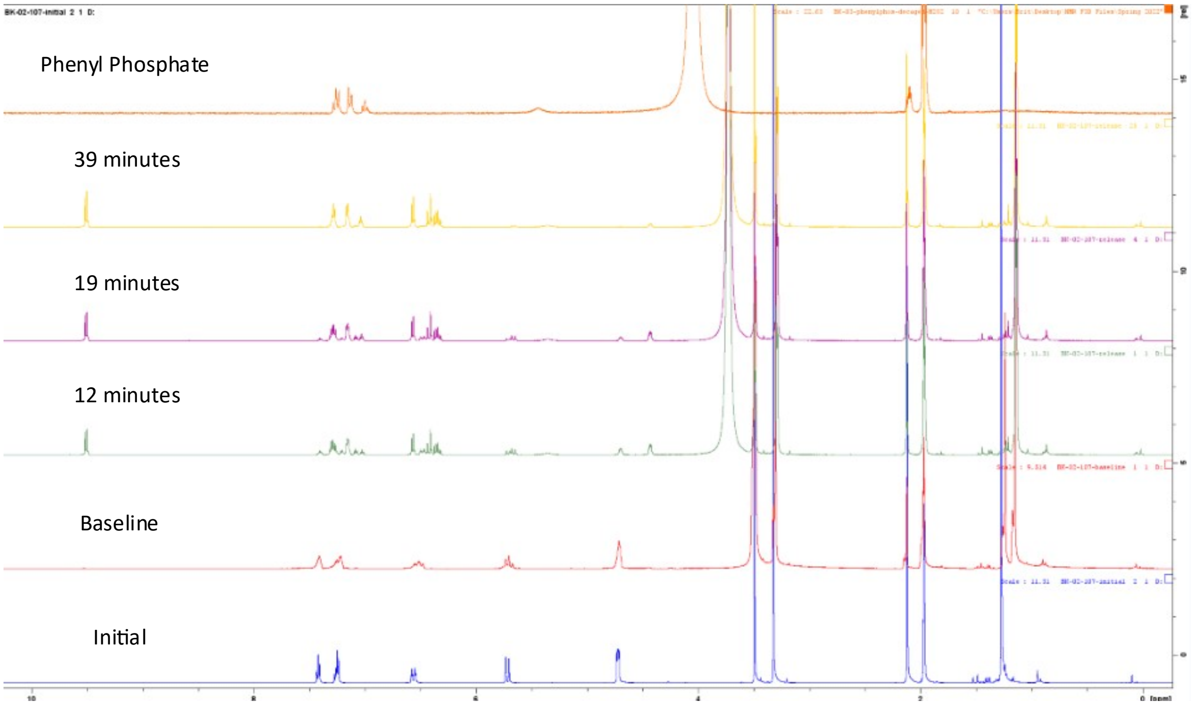

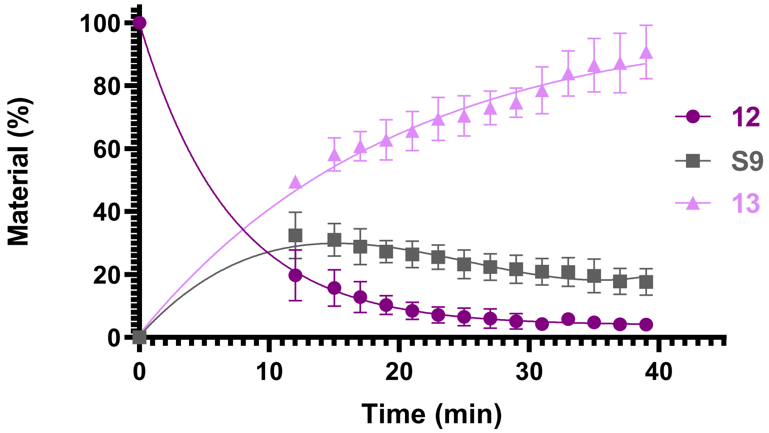

Figure S6. Oxidative cleavage of phenyl phosphate **12**.

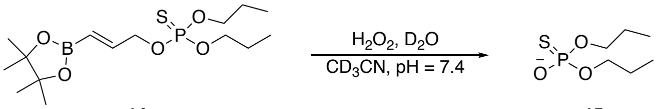

14 **15**

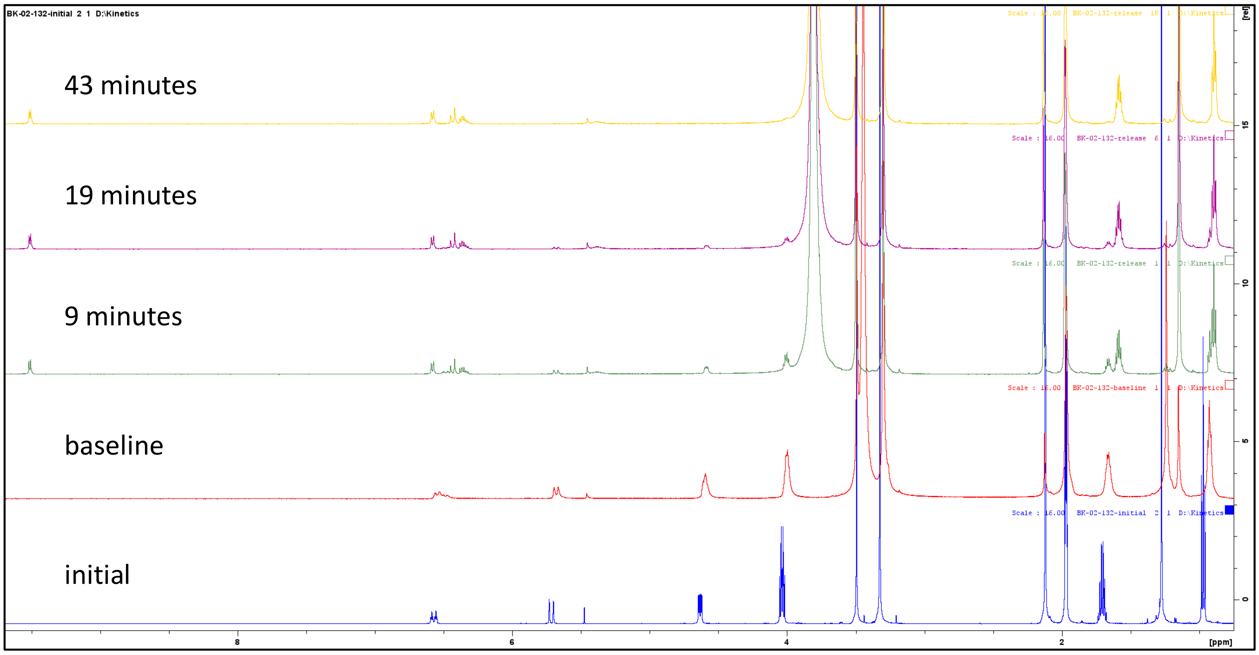

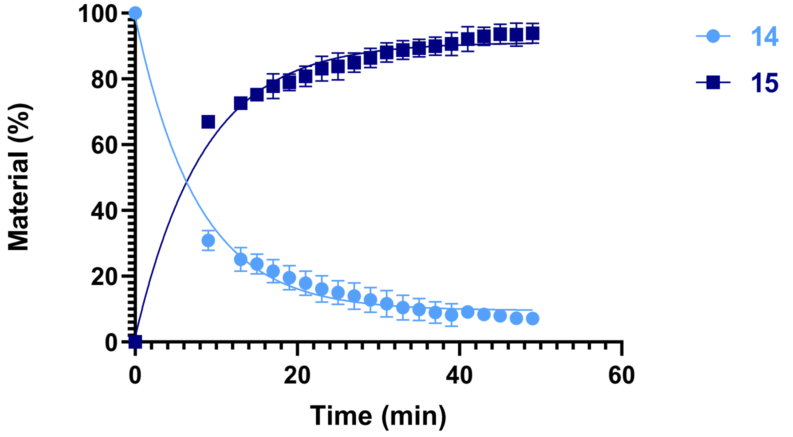

Figure S7. Oxidative cleavage of thiophosphate **14**.

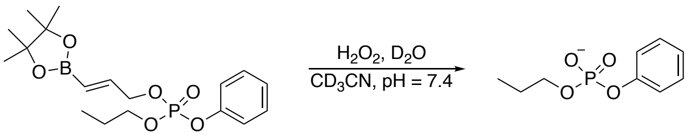

16 **17**

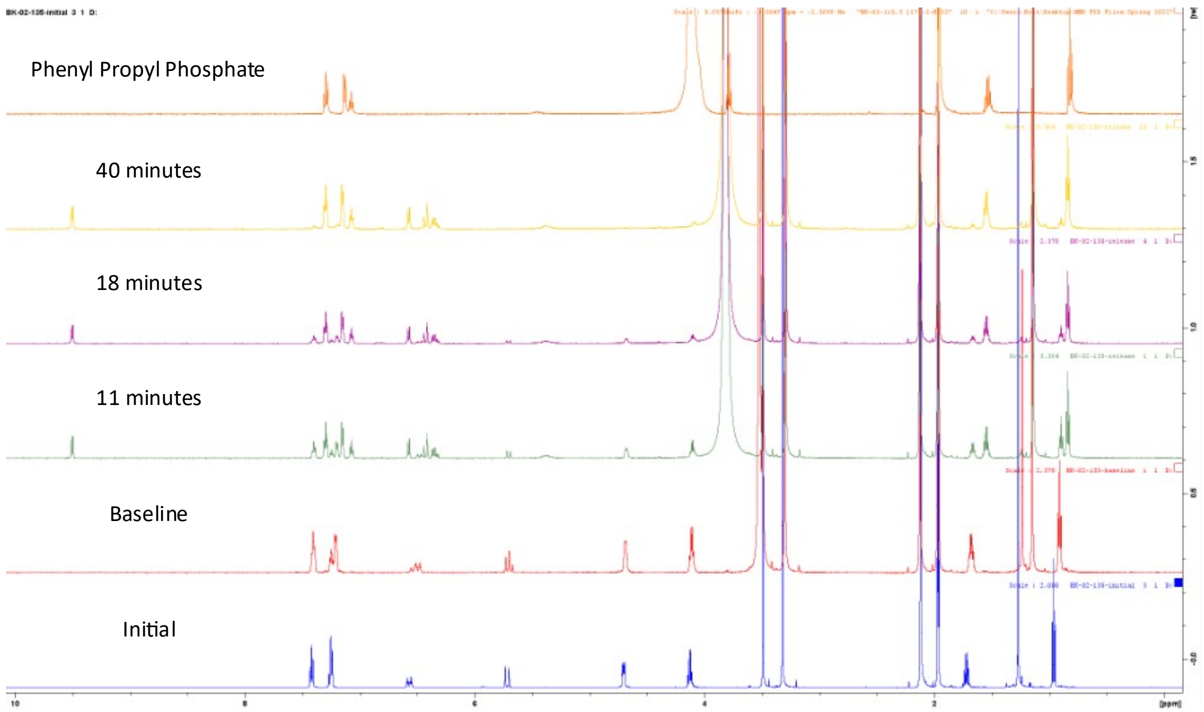

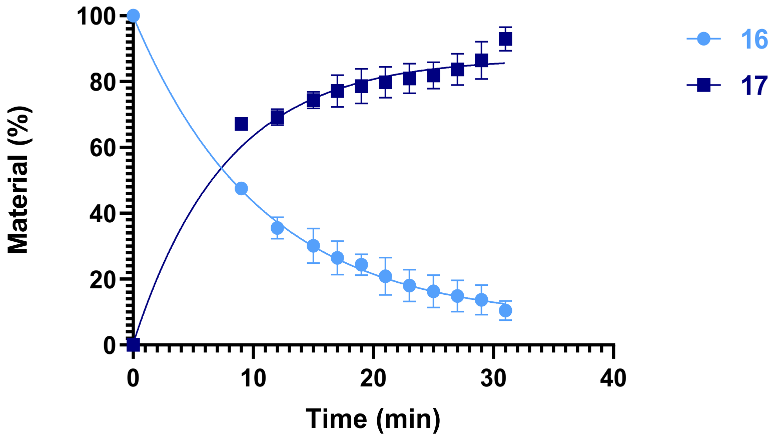

Figure S8. Oxidative cleavage of propyl phenyl phosphate **16**.

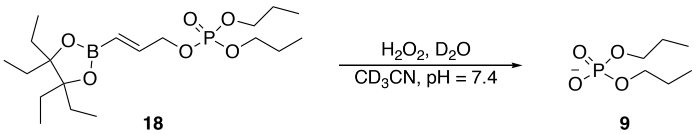

18

9

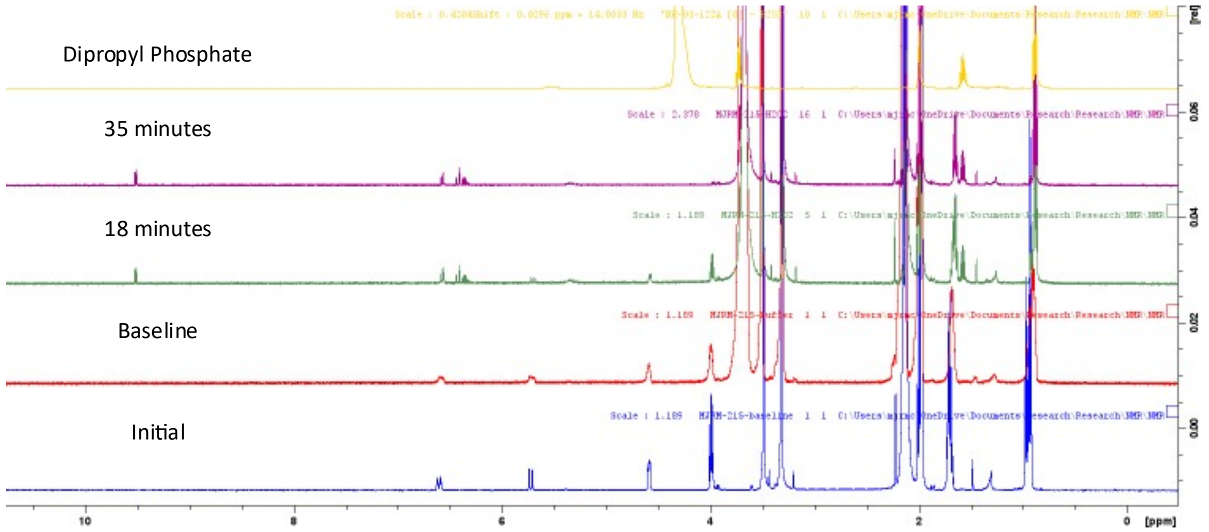

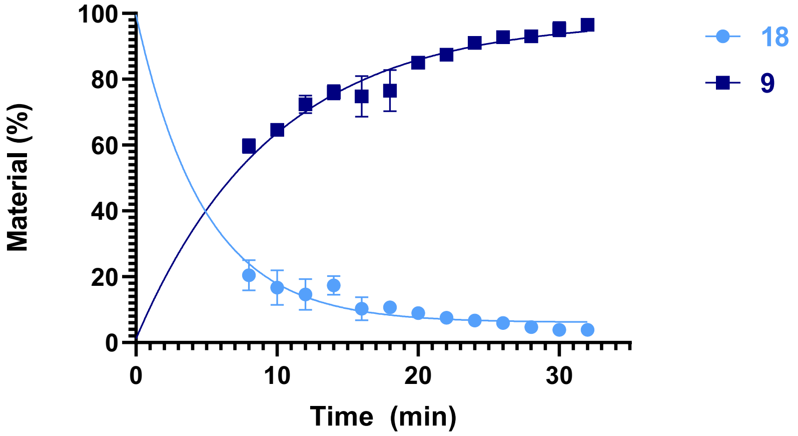

Figure S9. Oxidative cleavage of Epin-containing phosphate **18**.

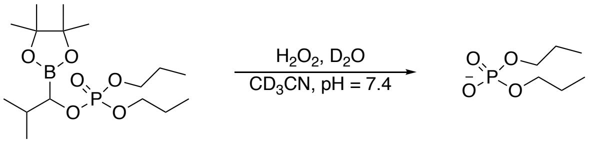

19 **9**

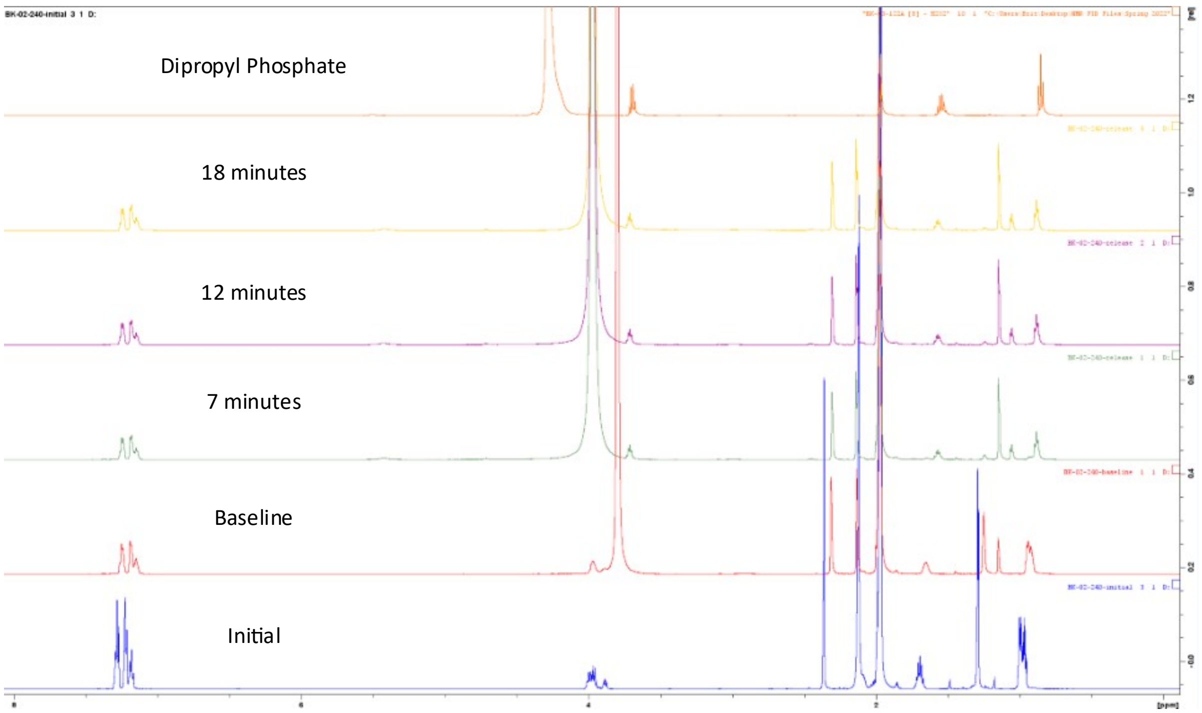

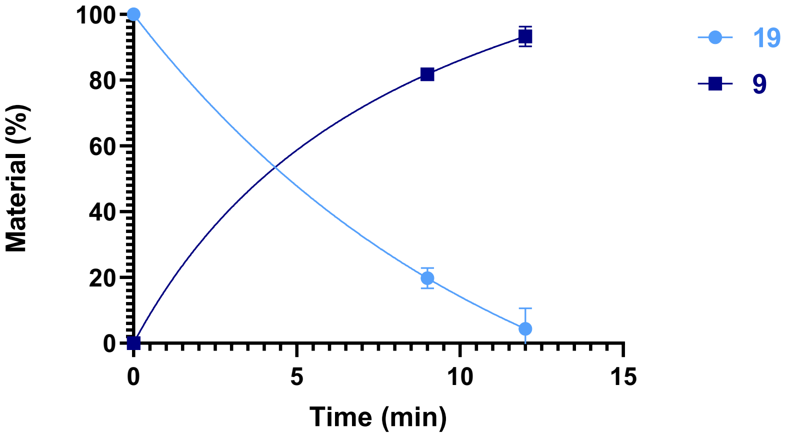

Figure S10. Oxidative cleavage of α-boryl phosphate **19**.

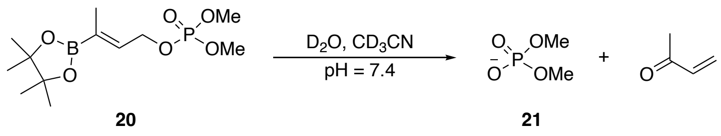

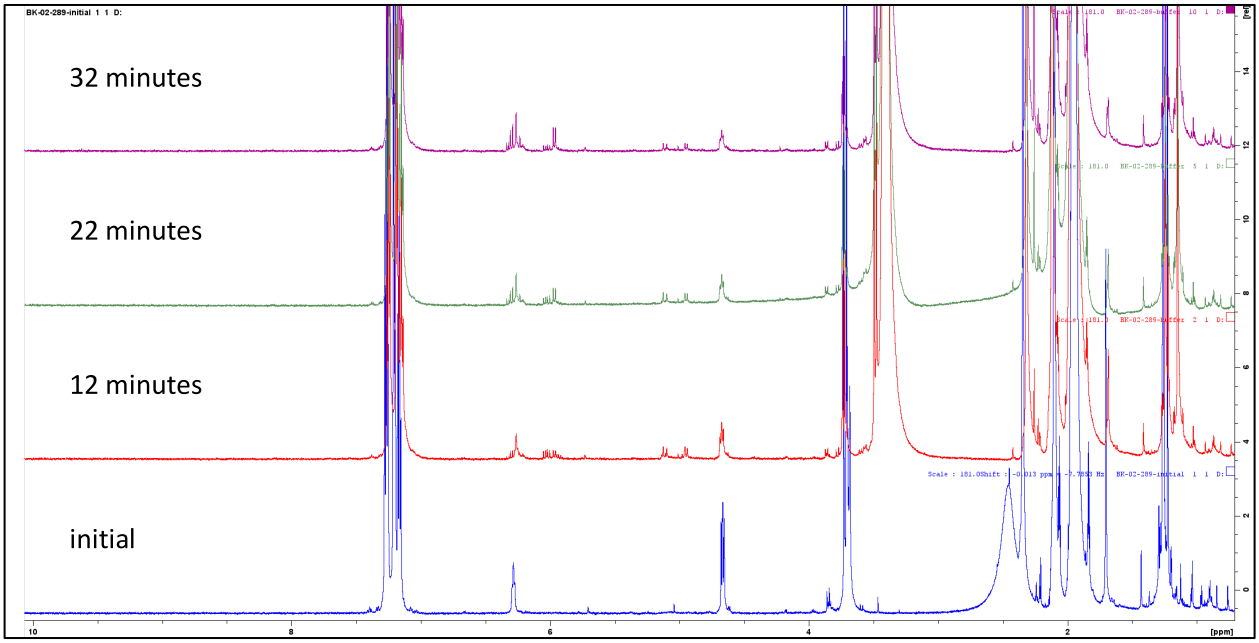

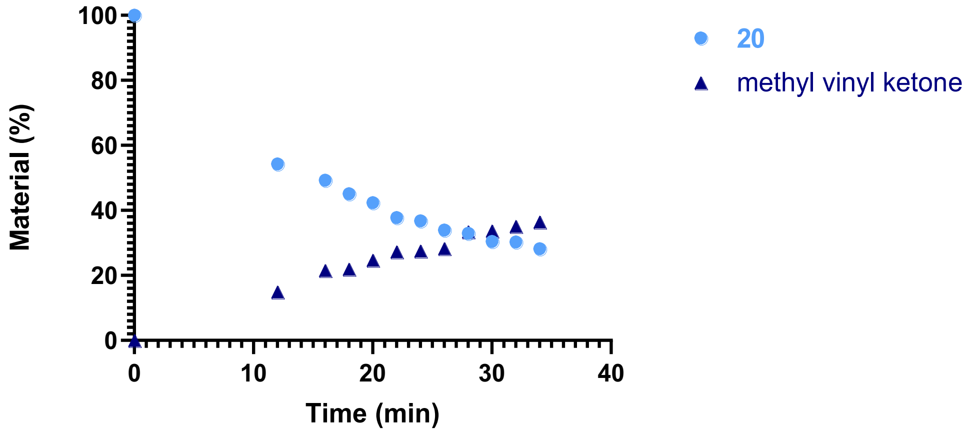

Figure S11. Aqueous instability of phosphate **20**.

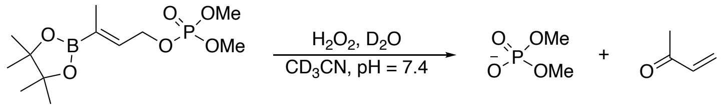

20 **21**

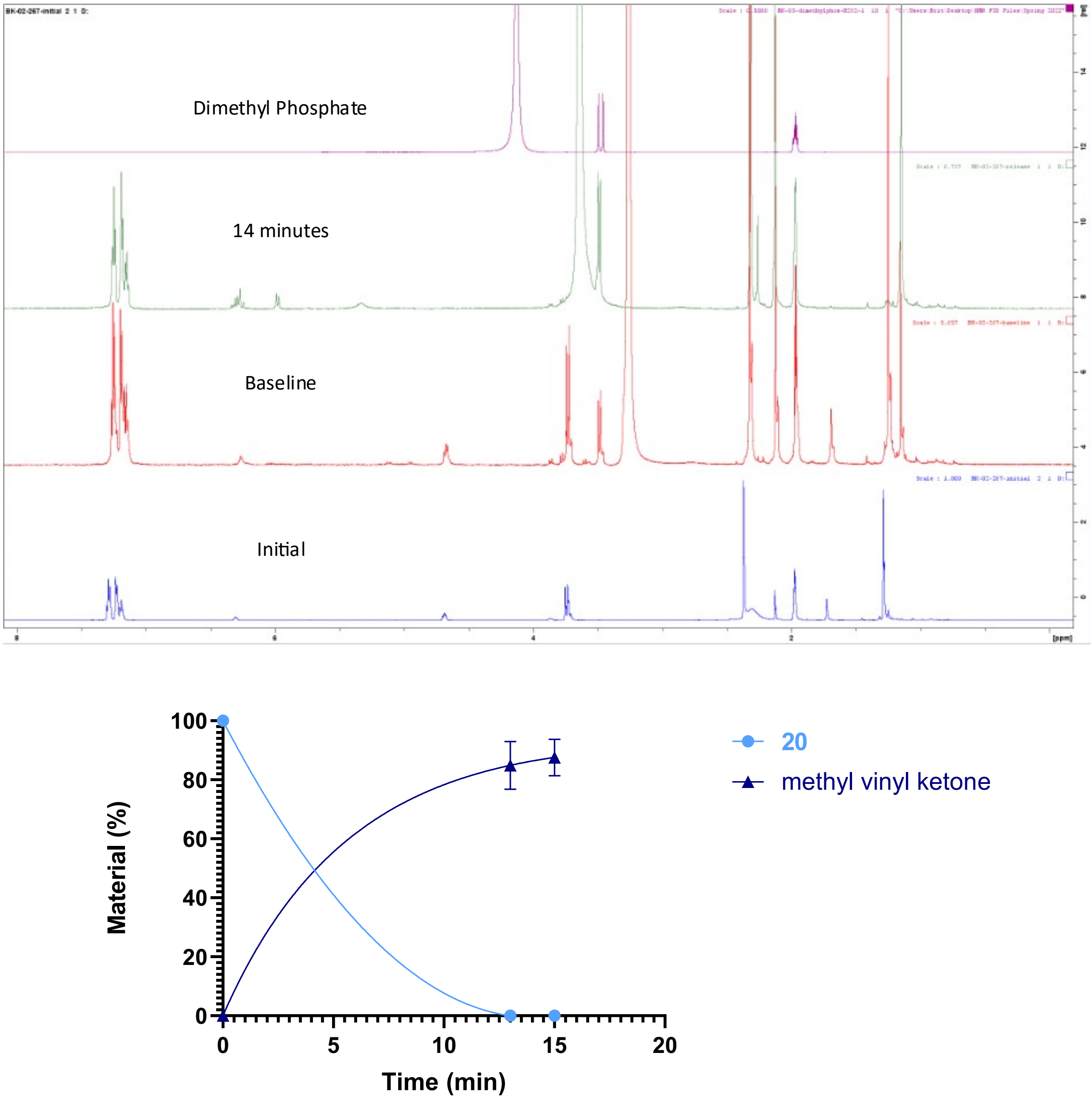

Figure S12. Oxidative cleavage of phosphate **20**.

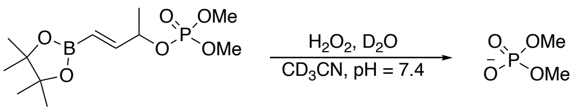

22 **21**

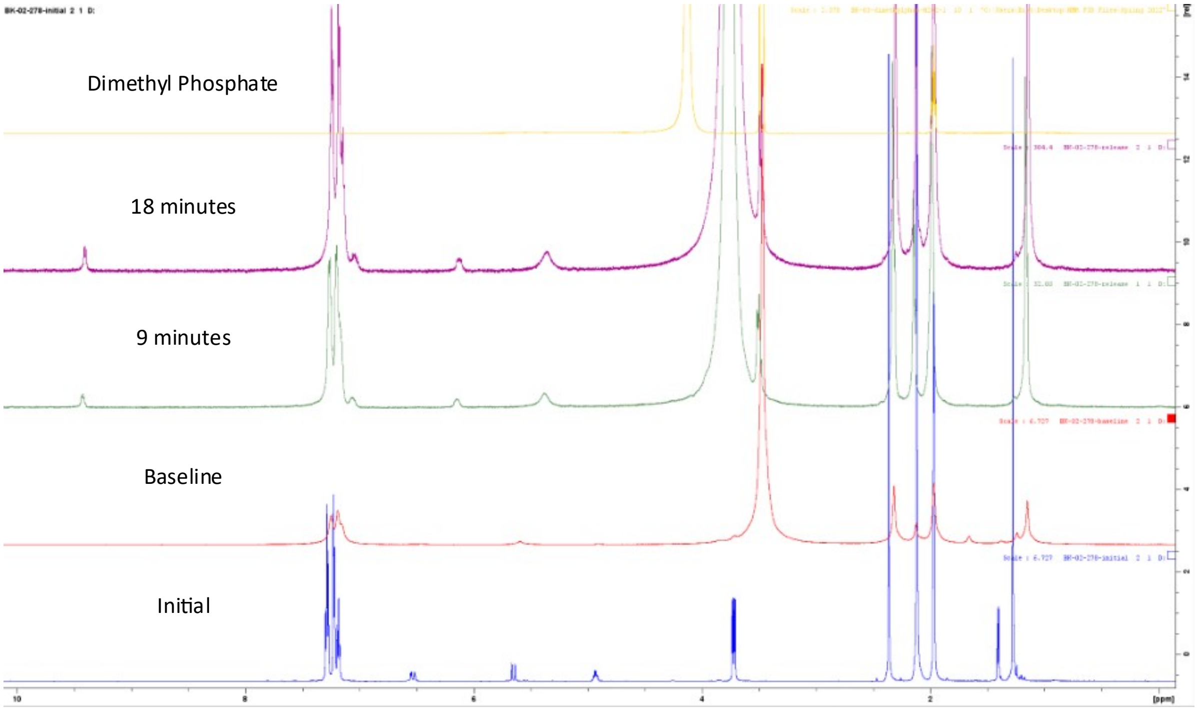

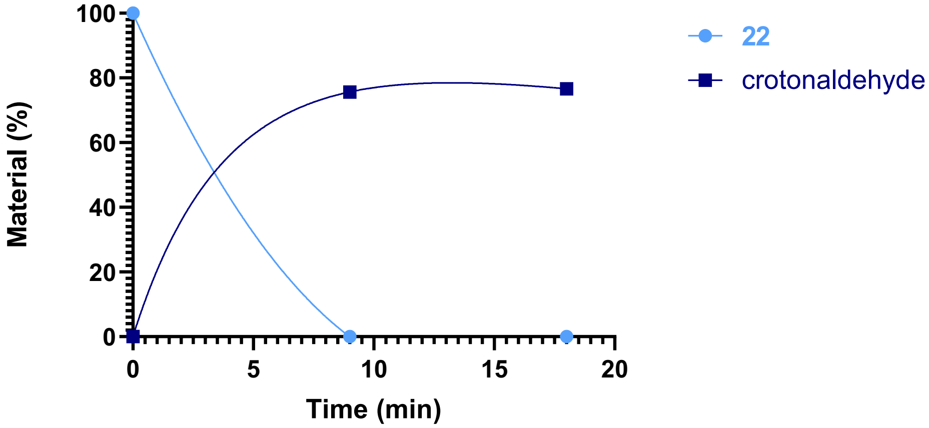

Figure S13. Oxidative cleavage of secondary phosphate **32**.

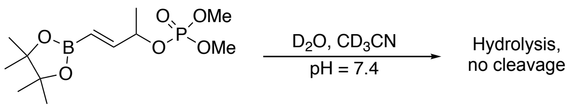

22

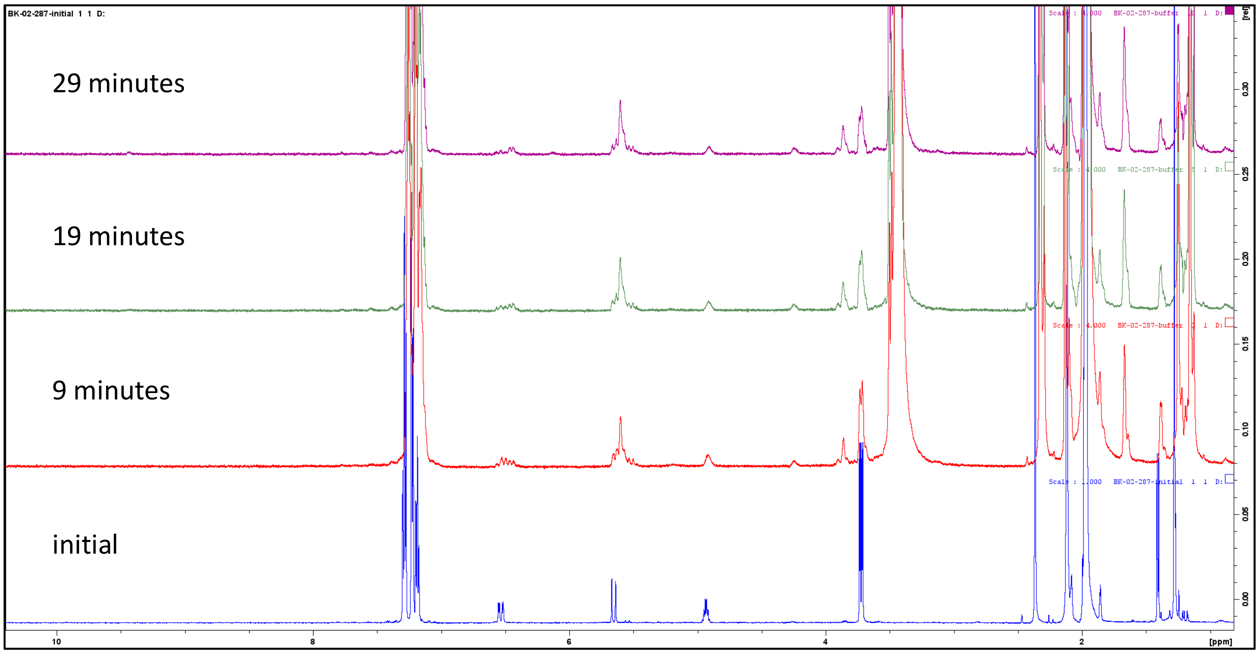

Figure S14. Compound **22** in aqueous buffer but in the absence of H_2O_2.

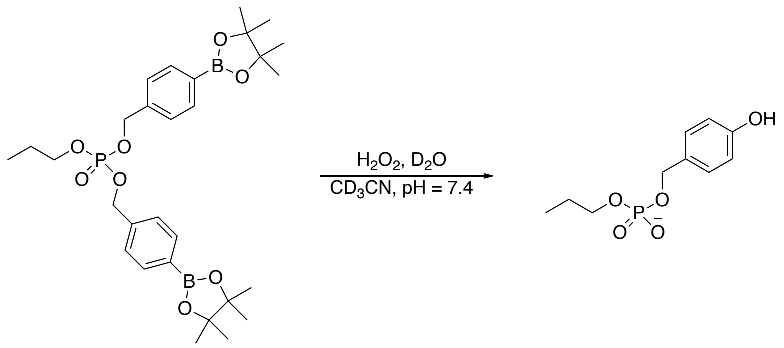

S10

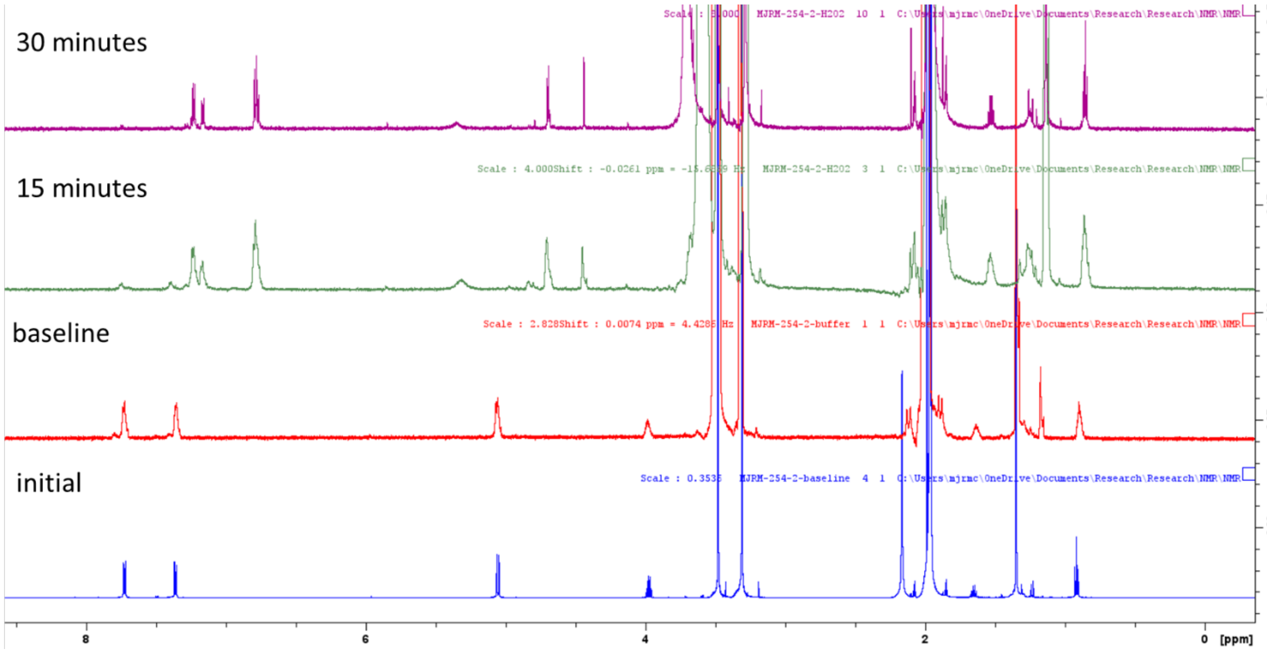

Figure S15. Partial cleavage of dibenzylic phosphate **S10**.

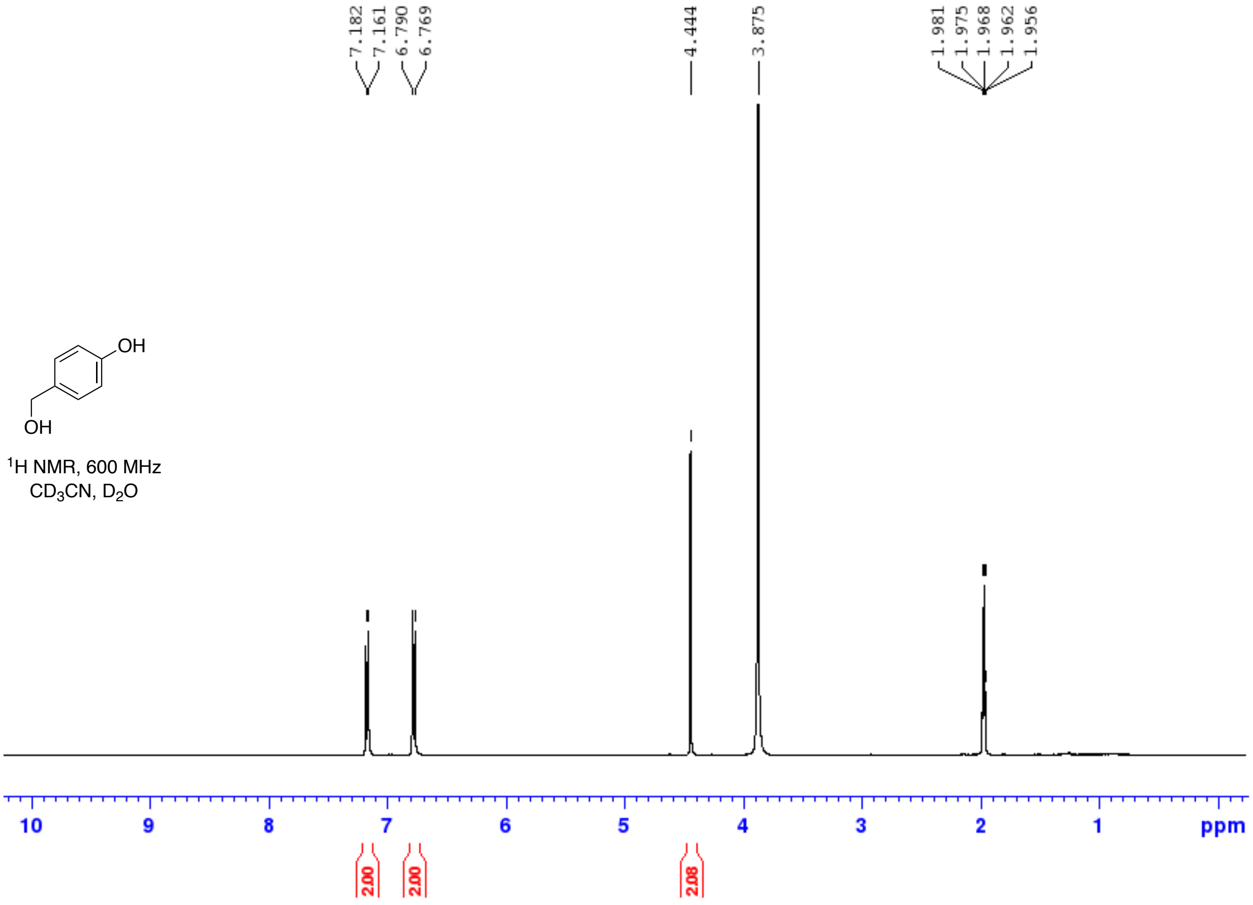

Figure S16. Confirmation of *para*-hydroxy benzyl alcohol formation.

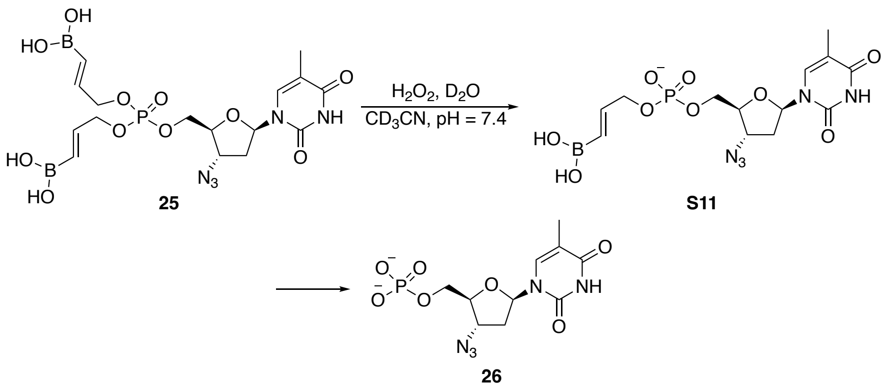

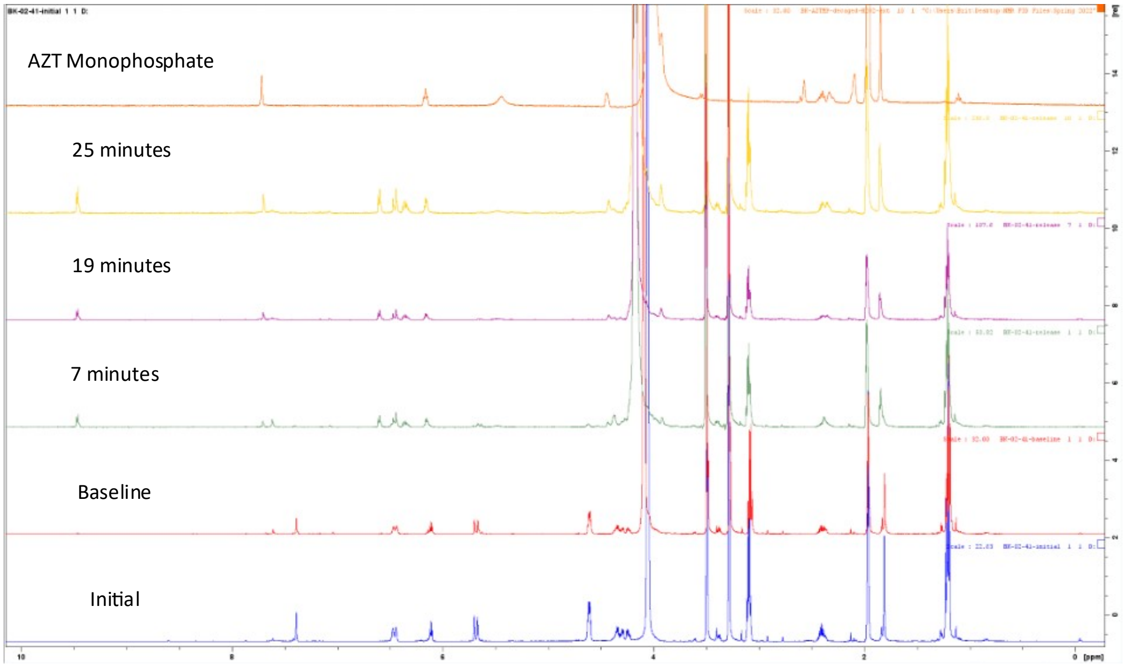

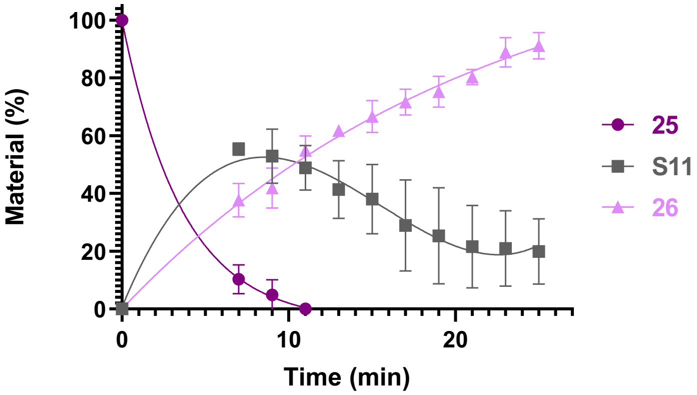

Figure S17. Oxidative cleavage of AZT-prodrug **25**.

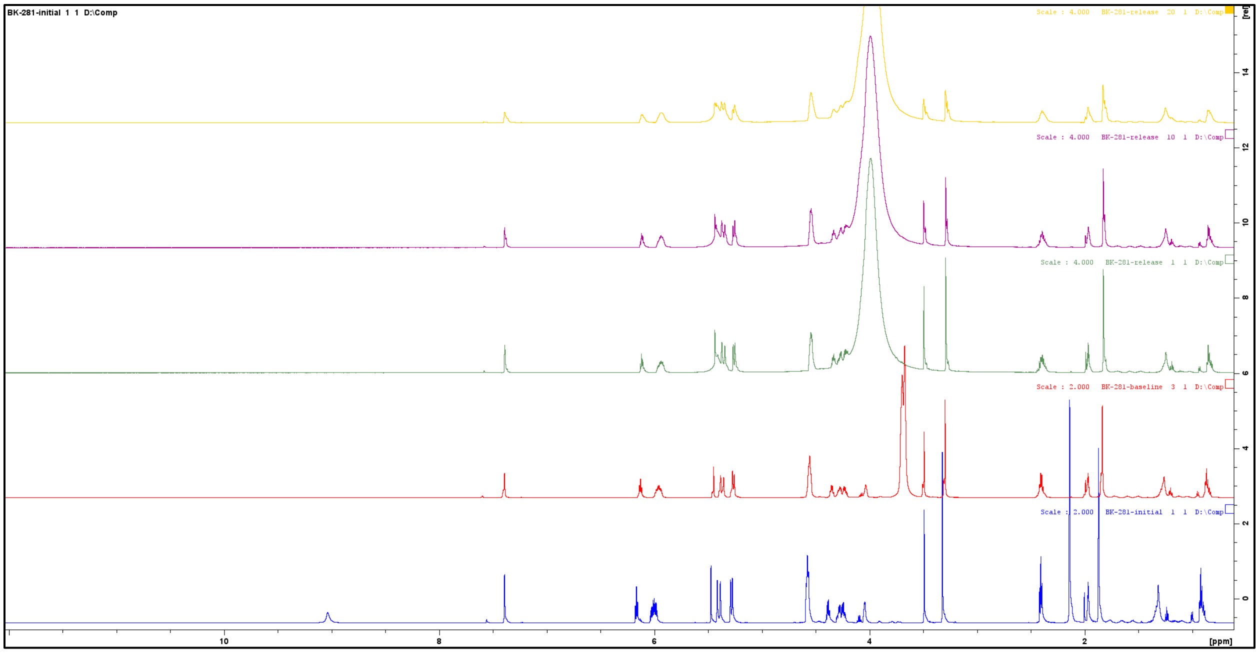

Figure S18. Exposure of negative control **27** to H_2O_2 at pH = 7.4. Blue: *t* = 0, CD_3CN. Red: *t* = 0, CD_3CN, D_2O, pH = 7.4 phosphate buffer. Green: *t* = 9 min, CD_3CN, D_2O, pH = 7.4 phosphate buffer, H_2O_2. Purple: *t* = 27 min, CD_3CN, D_2O, pH = 7.4 phosphate buffer, H_2O_2. Yellow: t = 47 min, CD_3CN, D_2O, pH = 7.4 phosphate buffer, H_2O_2.

HPLC-based study of the oxidative breakdown of 23.

Phosphate **23** (2 µL, 100 mM in DMSO) was diluted with PBS buffer (pH = 7.4, 990 µL) to prepare a solution with a final concentration of 200 µM. The solution was incubated at 37 °C for 10 minutes and then treated with aqueous H_2O_2 (8 µL, 100 mM in PBS buffer). The reaction was further incubated at 37 °C and 110 µL aliquots were removed at multiple time points and quenched with $Na_2S_2O_3$ (20 µL, 40 mM in H_2O). These aliquots were analyzed via HPLC at time points of 15, 30, 60, and 140 min, and the chromatograms were compared to those of pure materials. These materials were prepared in stock solutions (100 mM in PBS buffer, pH = 7.4) with a final concentration of 200 µM. The experiments employed a 250 x 4.6 mm Luna 5 mm C18 100 Å LC column with a gradient of 10-35% CH_3CN in H_2O over 40 min with a flow rate of 1 mL/min. A Hitachi Diode Array Detector (L-2455) at 220 nm, was used to monitor the compounds. Aliquots were studied by LCMS at various times during the experiment. Starting phosphate **23** and its corresponding boronic acid were completely consumed by the first time point while the monocleavage product mass was prominent, indicating that the monocleavage product and the starting material co-elute but the initial cleavage is quite rapid.

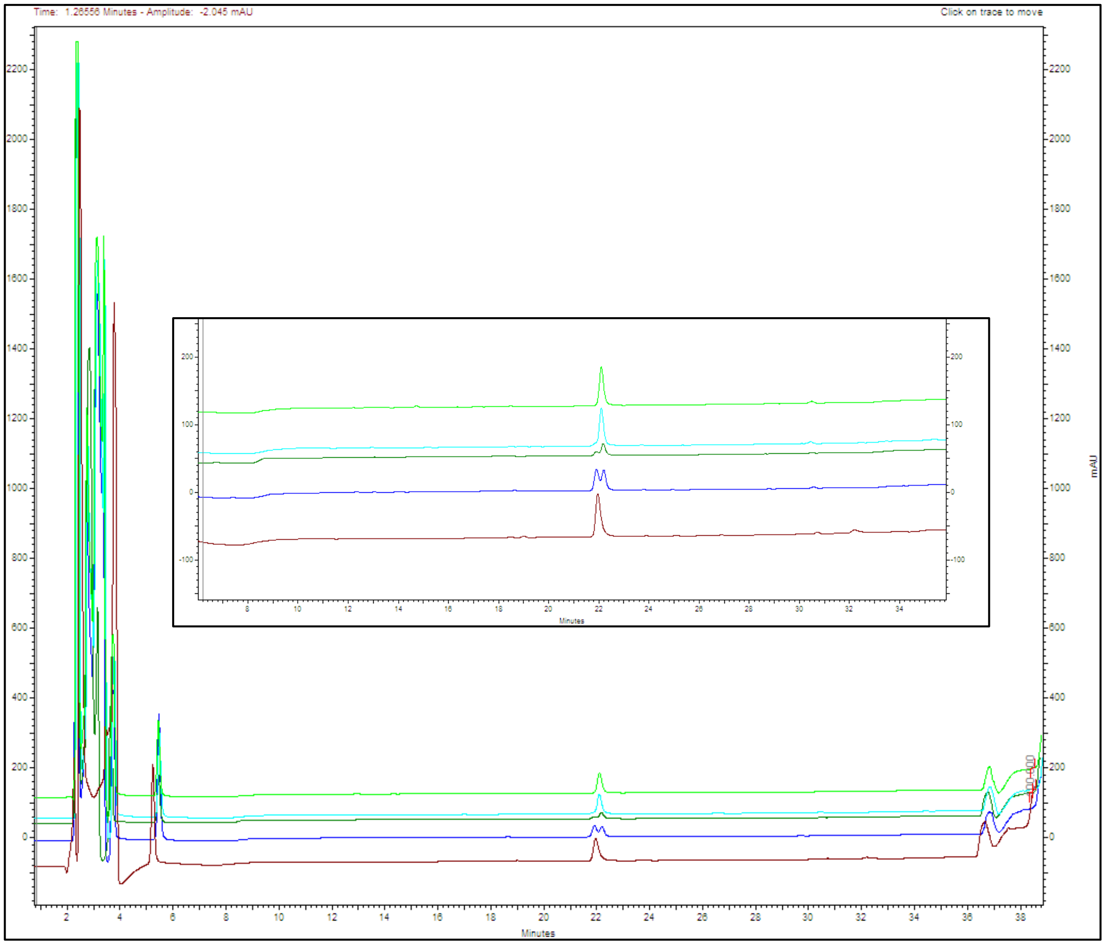

Figure S19. Chromatograms over time post peroxide addition of to **23**. Blue = 10 min, dark green = 30 min, turquoise = 60 min, lime green = 140 min. The inset is a zoomed in section of the chromatograms.

Evaluation of Acrolein Toxicity from BAO Cleavage

To evaluate the toxicity of the released byproduct acrolein, boronate-modified cyclohexanol **S11**, in which oxidative cleavage to yield acrolein was independently verified, was utilized as a control compound. The cytotoxicity of this compound and its subsequent byproducts was determined using a Sulforhodamine B (SRB) assay.[10] In this assay, SRB first binds to cell-surface proteins under acidic conditions. Cells are then washed several times to remove any unbound dye before adding a Tris base buffer, which extracts all bound SRB from the cell-surfaces. The amount of dye present in each well, or the absorbance at 510 nm, is used to represent cell density and to calculate the percent cell viability. Cytotoxicity was tested in an immortilized cell line derived from non-cancerous human tissue, HEK293T. HEK293T cells have low micromolar levels of endogenous peroxide,[11] and therefore can be utilized as a negative control for boronate release. Upon treatment with exogenous peroxide, acrolein is produced, but no increase in cellular toxicity was observed with up to 100 μM concentrations of the test substrate (Figure 2). Compound **S12**, an analog of the cytotoxin pederin, was used as an assay control. These results suggest no toxicity of the acrolein byproduct formed during boronate release.

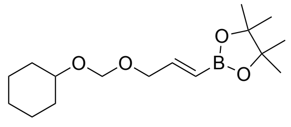

S11

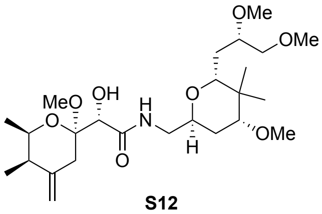

S12

HEK293T cells were grown and maintained at 37 ºC with 5% CO_2 in tissue culture-treated 10 cm plates (Corning 353003). Cells were grown in Dulbecco's Modified Eagle Medium (DMEM, HyClone sh3000303) supplemented with 10% fetal bovine serum (HyClone sh3039603) and tested every 3 months for mycoplasma contamination.

For each compound, a series of solutions was prepared (DMEM, 10% FBS, 10% DMSO) using serial dilutions to achieve 10x final concentrations (0.0001 – 1 mM). If appropriate, serial dilutions also included a 10x final concentration of hydrogen peroxide (1 μM). Cells were plated in white, clear-bottom, 96-well plates (Greiner 655098) at a cell density of 5,000 cells/well in a total volume of 90 μL per well. Immediately after plating, cells were treated in triplicate with the 10x compound solution to achieve the experimental concentration of each compound (0.00001 – 100 μM, 1% DMSO). For each experiment, DMSO-treated wells served as a "no treatment" control, while on a separate 96-well plate, a set of DMSO-treated wells was plated for use as a "no growth" control. The plates containing compound treated cells or the "no treatment" control wells were incubated for 48 hours, as to allow for the "no treatment" control cells to reach ~80% confluency. The plates containing the "no growth" control cells were incubated for only 3 hours to allow enough time for attachment of the cells to the plate, then the cells were fixed, by the slow addition of 100 μL ice-cold 30% trichloroacetic acid. After a 1 hour incubation, these plates were washed 3x with water, then allowed to air-dry. After the specified incubation time for the experimental plates, these cells were fixed, washed, and dried in the same manner. After all plates were completely dry, 100 μL of 0.04% SRB (20 mg SRB, 500 μL acetic acid, 49.5 mL water) was added to each well and allowed to incubate for 30 minutes. After this time, each well was quickly washed 3x with 100 μL of 2% acetic acid solution to remove any unbound dye, then again allowed to air-dry. To each well, 100 μL of Tris solution (10 mM, pH 10.5) was added and the plates were placed on the room-

temperature shaker for 30 minutes to solubilize bound dye. Absorbance was measured at 510 nm using a Tecan Infinite M1000 plate reader. The % cell viability was calculated using the following formula: $[A_{sample} - A_{no\ growth}] / [A_{no\ treatment} - A_{no\ growth}]$. Data was then plotted in Prism 8 using a non-linear regression curve fit to calculate IC_{50} values.

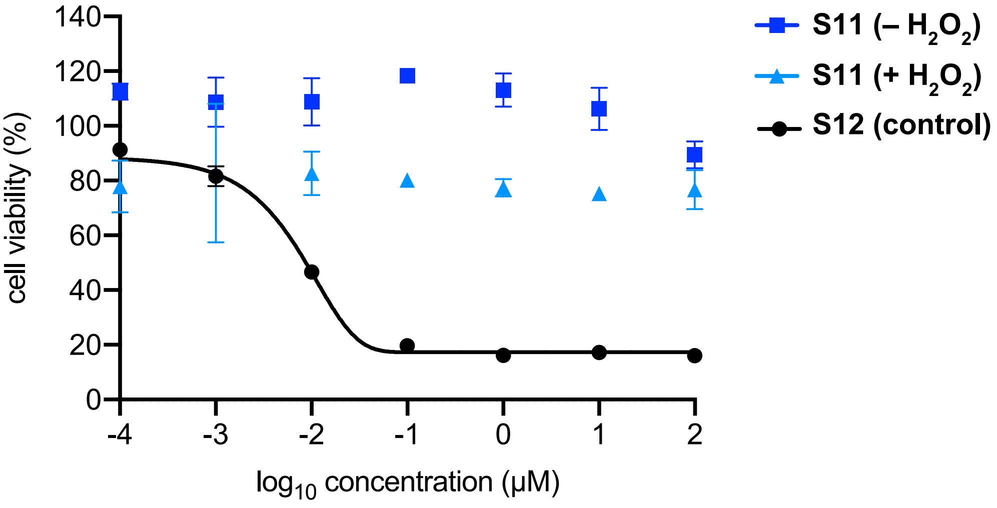

Kinase studies

Plasmid Construction: pTMPK-HA-P2A-mCherry.

Gibson assembly cloning was used to generate the pTMPK-P2A-mCherry plasmid. The TMPK gene was amplified from pcDNA3.1-DTYMK (Addgene 100546) using primers P1 and P2 (Table S1) and the backbone was amplified from pUb-A-EGFP-P2A-mCherry (*J. Am. Chem. Soc.* **2021**, *143*, 9222) using primers P3 and P4. Gene/plasmid amplification for both reactions was performed in a 50 µL reaction volume containing template DNA (10 ng), 10 µL of 5x NEB HF buffer (Thermo Scientific, F530L), 1 µL of dNTP mix (10 mM, each, Thermo Scientific, R0191), 1 µL of both forward and reverse primers (10 µM, each, Sigma Aldrich), and 0.5 µL of Phusion polymerase (2 U/µL, Thermo Scientific, F530L). The reactions included an initial denaturing step (95 °C, 3 minutes), followed by 30 cycles of denaturing (95 °C, 30 s), annealing (61.7 °C, 30 s), and elongation steps (72 °C, 3 minutes). The thermocycler program was completed with final elongation (72 °C, 10 minutes) and cooling steps (12 °C, 5 minutes), and the samples were stored at 4 °C until further use. Template DNA was digested by combining 44 µL of the PCR reaction mixture with 5 µL of 10x CutSmart buffer and 1 µL of DpnI (20 U/µL, NEB, R0176S) for 1 hour at 37 °C. The insert and backbone PCR products were gel extracted by adding 10 µL of 6x loading dye (NEB, B7024S) to the PCR mixture and running the full volume on a 0.8% (w/v) agarose gel in Tris-buffered EDTA (TBE, 10 mM Tris-HCl, 1 mM EDTA, pH = 8.0) buffer at 80 V for 30 minutes. The band corresponding to the correct length was cut from the gel and purified with the GeneJET Gel Extraction Kit (Thermo Scientific, K0692) according to the manufacturer's protocol. The insert and backbone fragments were ligated using a standard Gibson assembly method. To transform cells, a 50 µL aliquot of chemically competent Top10 cells was thawed on ice in a 1.7-mL microcentrifuge tube (Laboratory Products Sales, L211511) and combined with 5 µL of the Gibson assembly mixture. The cells were mixed by gently tapping the side of the tube, and the tube was incubated on ice for 30 minutes. The cells were heat shocked by incubating the tube in a hot water bath (42 °C, 30 s), followed immediately by incubation on ice for 2 minutes. Ice-cold SOC media (200 µL, VWR, 100219-988) was applied to the cell mixture, and the tube was incubated at 37 °C with shaking at 250 RPM for 40 minutes. The entire contents of the transformation mixture were plated on 10 mL of LB agar supplemented with 10 µL of a 100 mg/mL kanamycin stock and incubated overnight at 37 °C. Three colonies were inoculated into 5 mL of LB broth supplemented with 5 µL of a 100 mg/mL kanamycin stock and grown overnight with shaking (37 °C, 250 rpm), followed by DNA miniprep (Thermo Scientific, K0503). Plasmid sequence was confirmed by Genewiz Sanger sequencing using CMV forward, EGFP-N, and EGFP-C reverse universal primers (P5, P6, and P7).

The Gibson assembly procedure described above was used to generate pTMPK-HA-P2A-mCherry. The HA epitope sequence was built into primers P8 and P9, and the PCR amplification was completed as described above with an annealing temperature of 63.6 °C and an elongation time of 3.5 minutes. The Gibson assembly, transformation, miniprep, and sequencing were completed as described above.

Site-directed mutagenesis was performed to introduce the F105Y mutant to pTMPK-HA-P2A-mCherry using primers P10 and P11. The reaction was completed in a 50 µL reaction volume containing template DNA (10 ng), 10 µL of 5x NEB GC buffer (Thermo Scientific, F530L), 1 µL of dNTP mix (10 mM, each, Thermo Scientific, R0191), 1 µL of both forward and reverse primers (5 µM, each, Sigma Aldrich), 1.5 µL of DMSO, and 0.5 µL of Phusion polymerase (2 U/µL,

Thermo Scientific, F530L). The thermocycler reaction protocol was completed as described above using an annealing temperature of 64.7 °C and an elongation time of 3.5 minutes. The DpnI treatment was completed as described above. To assess the purity of the PCR product, 2 µL of the mixture was diluted in 8 µL of water and 2 µL of 6x loading dye and run on a 0.8% (w/v) agarose gel in Tris-buffered EDTA buffer at 80 V for 30 minutes. If a single band corresponding to the correct length was observed, the mixture was used immediately for transformation. If impurities were observed, the PCR product was purified by gel extraction, as described above. PCR product transformation, miniprep, and plasmid sequencing were completed as described above.

Table S1. Cloning and Mutagenesis Primers. List of primers used for plasmid construction (shown 5' to 3'). Base mutations are indicated by capitalization.

Primer	Sequence
P1	cagatctcgagctggaattctgcagatatcatggcggcccgg
P2	agctccgcttcccttccatagctcccccagcggcttctctg
P3	gagctatggaagggaagcggagctactaacttcagcctgctgaagc
P4	tgcagaattccagctcgagatctgagtccggtagcgctagcg
P5	cgcaaatgggcggtaggcgtg
P6	cgtcgccgtccagctcgacca
P7	gttcagggggaggtgtg
P8	tacccatacgatgttccagattacgctggaagcggagctactaacttcagcctgctgaagcaggc
P9	agcgtaatctggaacatcgtatgggtacttccatagctcccccagcggcttctctgtgg
P10	gtggccTACaccggtgccaaggagaatttttccctagattggtgtaaacagc
P11	accggtGTAggccacaccagaaaatgcgtatctgtccacgac

Western Blotting. For analysis of protein expression and pulldown HEK293T cells were seeded into a 24-well plate (100,000 cells/well, Greiner, 665160) in 500 µL of complete medium. When cells reached ~90% confluency, the media in each well was replaced with 450 µL of fresh antibiotic-free DMEM. The cells in each well were transfected by combining 800 ng of pTMPK-HA-P2A-mCherry with 4 µL of a 1 mg/mL stock of linear polythyleneimine (LPEI, Polysciences, 23966) and brought to 50 µL with OptiMEM transfection media (Thermo Scientific, 22600050) in a 1.7 mL microcentrifuge tube (Laboratory Products Sales, L211511). The transfection mixture was mixed by pipetting and incubated for 10 minutes at room temperature. The mixture was added dropwise to the media of each replicate well, and the cells were incubated for 48 hours at 37 °C.

Following the 48-hour incubation for protein expression, the cells were washed with 250 µL of ice-cold PBS to remove residual medium, placed on ice, and lysed with 100 µL of ice-cold GE Mammalian Protein Extraction Buffer (28941279) supplemented with 1 µL of 100x protease inhibitor cocktail (Thermo Scientific, 78429) with 250 RPM shaking for 30 minutes. Lysates were transferred to a 1.7 mL microcentrifuge tube. Cell debris was pelleted by centrifugation at 21000 g for 10 minutes at 4 °C and supernatant (54 µL) was transferred to a fresh microcentrifuge tube. An aliquot of lysate (12 µL) was transferred to a fresh tube and stored at -20 °C until further use.

Anti-HA antibody (0.1 µg, ProteinTech 50-173-5882) was diluted into 10 µL of fresh GE Mammalian Protein Extraction Buffer, added to each sample, and incubated for 1 hour at 4 °C. Protein A resin (10 µL, SCBT, sc-2001) was added to each sample and rocked for 2 hours at 4 °C, before being pelleted at 100 g for 5 minutes at 4 °C. After transferring a 12 µL aliquot of the supernatant to a fresh microcentrifuge tube (stored at -20 °C until further use), the remaining supernatant was discarded and the resin was washed three times with 300 µL of chilled TMPK assay buffer (10 mM Tris-HCl, 5 mM $MgCl_2$, 0.05% Tween-20, pH 7.6). The final wash was removed and discarded, and the beads were combined with 60 µL of water and 20 µL of 4x SDS-PAGE sample loading buffer (200 mM Tris-HCl, pH 6.5, 400 mM DTT, 8% (w/v) SDS, 6 mM bromophenol blue, 4 M glycerol). The lysate and flow through samples which had been collected previously were combined with 4 µL of 4x SDS-PAGE sample loading buffer and all three tubes were heated at 95 °C for 10 minutes.

Samples were separated by 10% (v/v) SDS-PAGE gel electrophoresis (60 V for 30 minutes, 150 V for 1 hour) with an ice pack placed inside the tank next to the gel cassette, followed by protein transfer to a 0.45 µm PDVF membrane (Millipore, IPVH00010) at 80 V for 90 minutes using ice-cold Transfer Buffer (25 mM Tris-HCl, 192 mM glycine, pH = 8.3). The membrane was blocked for 1 hour at room temperature with 7 mL of blocking buffer (5% milk in TBS with 0.1% (v/v) Tween 20, TBST) while rocking. Blots were probed with rabbit mAb anti-HA (1:2000 dilution, Cell Signaling Technology 3724S) and rabbit pAb anti-GAPDH (1:2000 dilution, ProteinTech 50-172-6351) primary antibodies in 7 mL of fresh blocking buffer overnight with rocking at 4 °C. After washing three times by incubating the blots with 10 mL of ice-cold TBST at room temperature with rocking, membranes were incubated for 1 hour at room temperature with goat anti-rabbit IgG HRP-linked secondary antibody (1:5000 dilution, Cell Signaling 7074S) in 7 mL of TBST. After washing another three times with 10 mL of ice-cold TBST at room temperature with rocking, blots were developed with SuperSignal West Pico Chemiluminescent Substrate (Thermo Scientific, 34580) by mixing 4 mL of the luminol/enhancer solution with 4 mL of the peroxide solution and incubating the membrane in the resulting solution for 5 minutes at room temperature with rocking. The blots were imaged on a BioRad ChemiDoc system with automated exposure times.

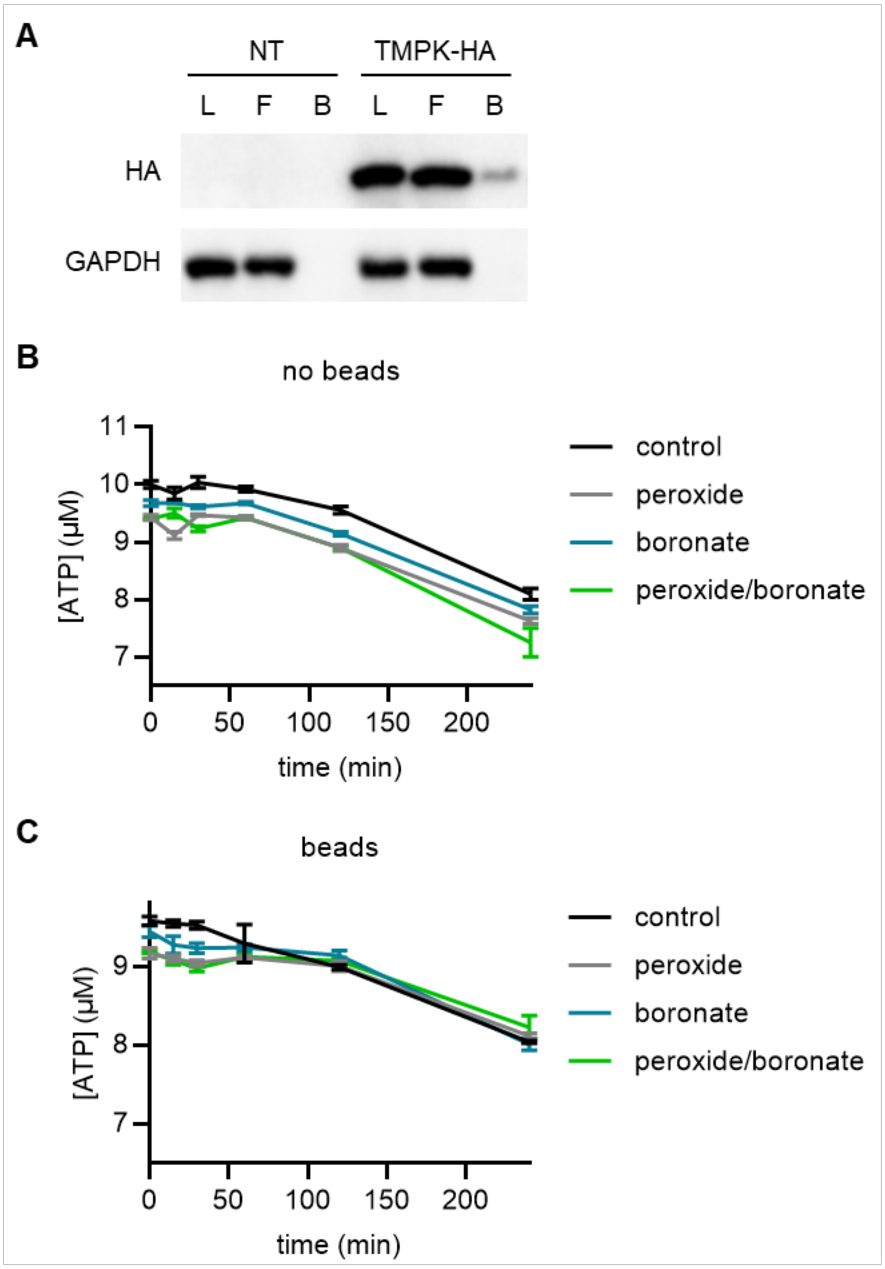

Figure S20. Timecourse analysis of ATP stability. A) Validation of TMPK-HA expression in HEK293T cells and pulldown from cell lysates. L = lysate; F = flow-through; B = bead eluants. Timecourse quantification of ATP concentration over the course of 4 hours in the absence or presence of H_2O_2 (60 μM), boronate-AZTMP (2 μM), or boronate AZTMP preincubated with H_2O_2. Assay completed in the absence (B) or presence (C) of Protein A agarose beads. Error bars denote standard deviation from three replicates.

TMPK Biochemical Assay. For biochemical evaluation of AZTMP phosphorylation by immobilized TMPK, TMPK-HA-P2A-EGFP was expressed in triplicate for each condition in a 24-well plate with the same transfection method used for western blot analysis. Following a 48 h incubation for protein expression, the cells were lysed as described in the Western Blot section.

The following procedure was duplicated for analysis of phosphate conversion in the absence of TMPK-HA through the use of non-transfected cells. Triplicate lysates were transferred and combined in a 1.7 mL microcentrifuge tubes (Laboratory Products Sales, L211511). Cell debris was pelleted by centrifugation at 21000 g for 10 minutes at 4 °C and supernatant (270 µL) was collected into a fresh tube. Anti-HA antibody (0.3 µg, ProteinTech 50-173-5882) was diluted into 30 µL of fresh GE Mammalian Protein Extraction Buffer, added to each sample, and incubated for 1 hour at 4 °C. Protein A resin (30 µL, SCBT, sc-2001) was added to each sample and rocked for 2 h at 4 °C, before being pelleted at 100 g for 5 minutes at 4 °C. The supernatant was discarded and the resin was washed four times with 550 µL of chilled TMPK assay buffer (10 mM Tris-HCl, 5 mM $MgCl_2$, 0.05% Tween-20, pH 7.6). Before spinning down the final wash step, the beads were separated into three 200 µL aliquots. The beads were pelleted (1000 g, 5 minutes, 4 °C) and the final wash was removed and discarded, leaving about 30 µL of liquid at the bottom of the tube. For the boronate-AZTMP assay, a master mix solution was made by combining 679 µL of TMPK assay buffer with 7 µL of a 200 µM boronate-AZTMP (**25**) stock solution and 7 µL of either water or a 6 mM H_2O_2 stock solution. Stock solutions for the AZTMP (**26**) and diallyl-AZTMP (**27**) positive and negative controls were generated by combining 679 µL of TMPK assay buffer with 7 µL of water and 7 µL of a 200 µM stock solution of either AZTMP (**26**) or diallyl-AZTMP (**27**). The master mix solutions were preincubated for one hour at 37 °C and completed by adding 7 µL of a 1 mM ATP stock solution to each tube. Beads were resuspended in 220 µL aliquots of the master mix solutions in triplicate and immediately spun down (1000 g, 1 minute) in order to take an aliquot for the 0 min timepoint. The beads were resuspended by gently tapping the side of the tube and the reactions were incubated at 37 °C for 2 hours. Aliquots were removed after 15, 30, 60, and 120 min by quickly pelleting the beads through centrifugation (1000 g, 1 minute) at 4 °C, and transferring 10 µL from each reaction to a fresh PCR tube (VWR, 20170-012). Following each timepoint, the beads were resuspended by gently tapping the side of the tube and the reactions were incubated at 37 °C until the next timepoint. After the reaction was complete and all samples were collected, each aliquot was diluted by adding 90 µL of fresh Millipore water, and 10 µL of each resulting solution was transferred to a white, clear bottom, 384-well plate (4500 cells/well, Greiner, 781182). The CellTiter-Glo reagent (Fisher Scientific, PR-G7570) was warmed to 37 °C and protected from light immediately before the start of the luminescence assay. An automatic 16-channel pipette (Thermo Scientific, 46300700) was used to quickly add 10 µL of the CellTiter-Glo reagent to each well. The plate was incubated at room temperature for five minutes, and luminescence was measured using a Tecan M1000 pro. Luminescence intensity values were normalized to the 0 min timepoint for each condition and multiplied by ten to calculate the concentration of ATP remaining. Data was averaged from three biological replicates and standard deviations were calculated.

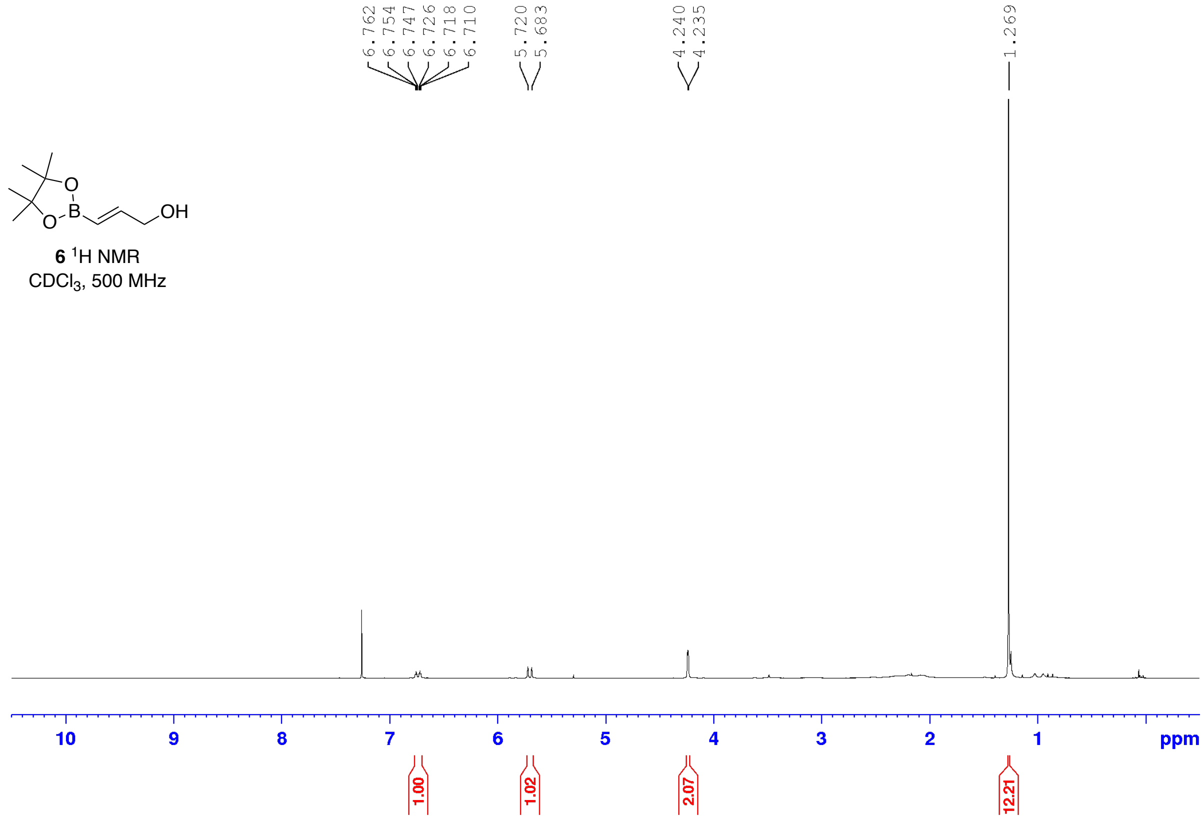

6 ^{1}H NMR
CDCl$_3$, 500 MHz
6.762
6.754
6.747
6.726
6.718
6.710
5.720
5.683
4.240
4.235
1.269
1.00
1.02
2.07
12.21
ppm

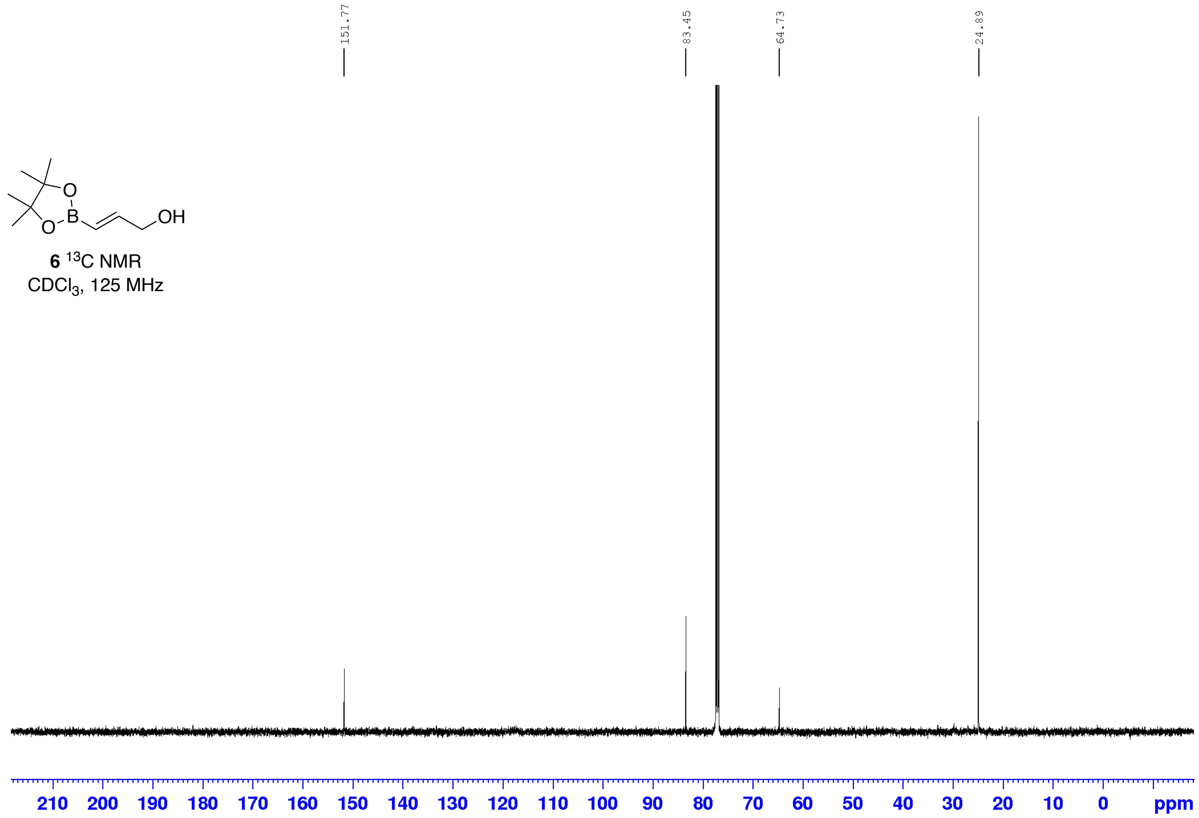

6 ^{13}C NMR
CDCl$_3$, 125 MHz
151.77
83.45
64.73
24.89
ppm

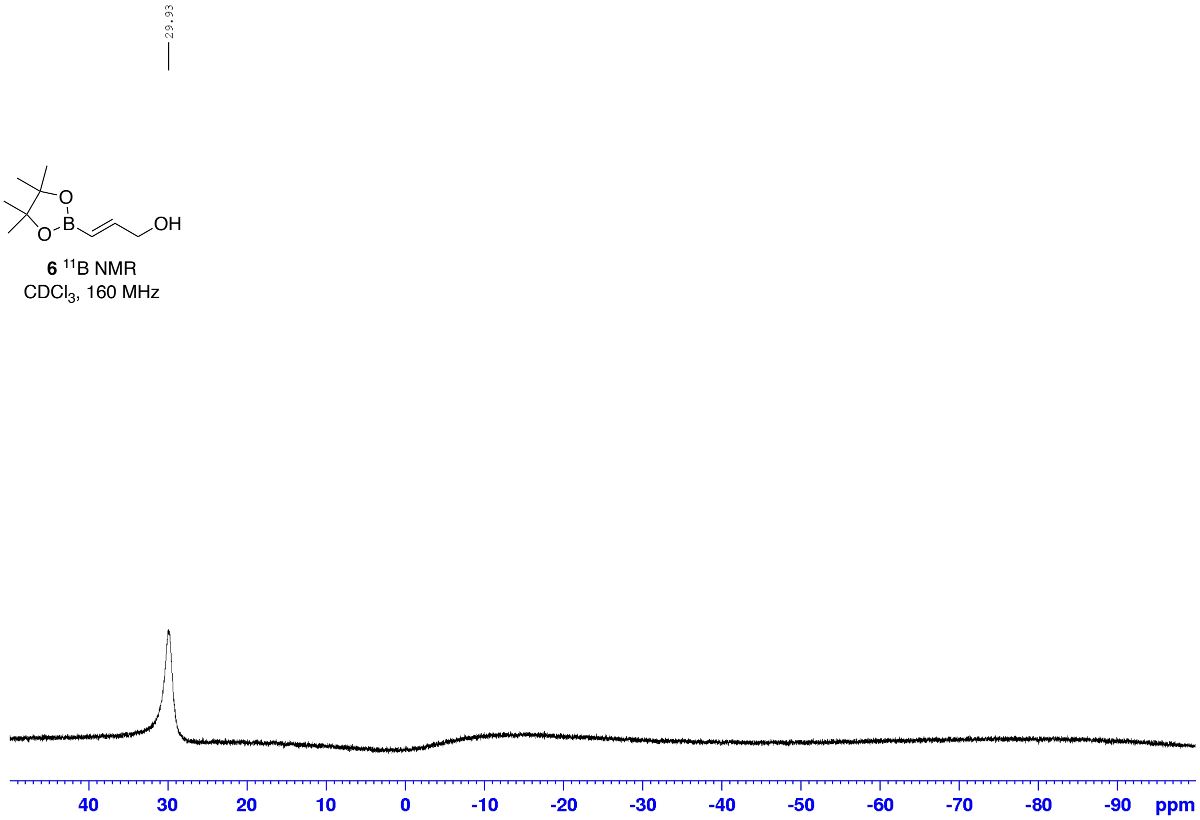
29.93
O
B
O
OH
6 ¹¹B NMR
CDCl₃, 160 MHz
40 30 20 10 0 -10 -20 -30 -40 -50 -60 -70 -80 -90 ppm

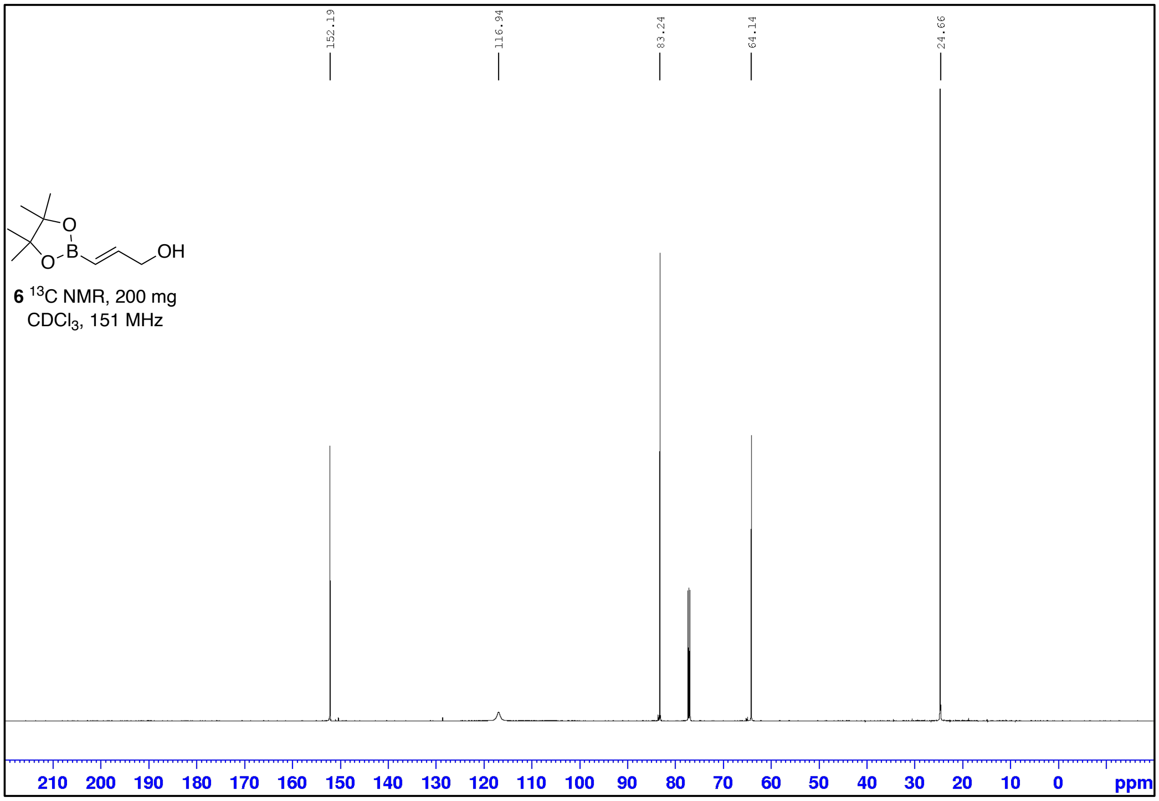
152.19
116.94
83.24
64.14
24.66
O
B
O
OH
6 ¹³C NMR, 200 mg
CDCl₃, 151 MHz
210 200 190 180 170 160 150 140 130 120 110 100 90 80 70 60 50 40 30 20 10 0 ppm

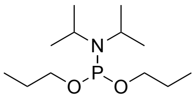

S1, ^{1}H NMR
400 MHz, CD_3CN

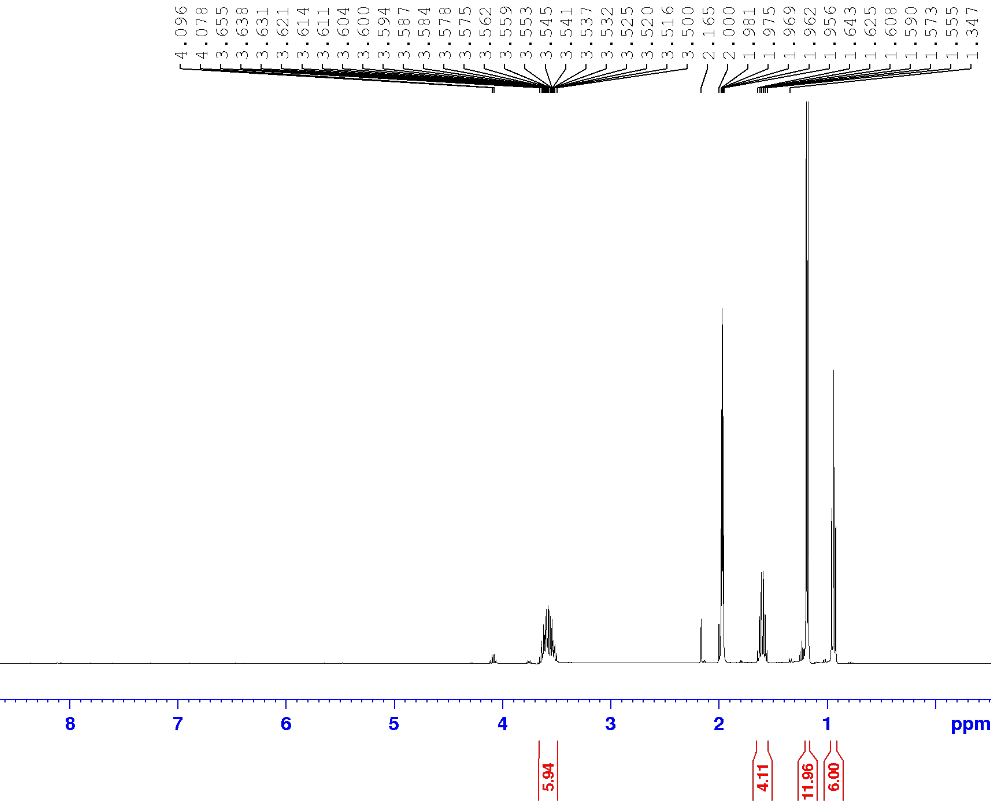

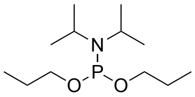

S1, ^{13}C NMR
100 MHz, CD_3CN

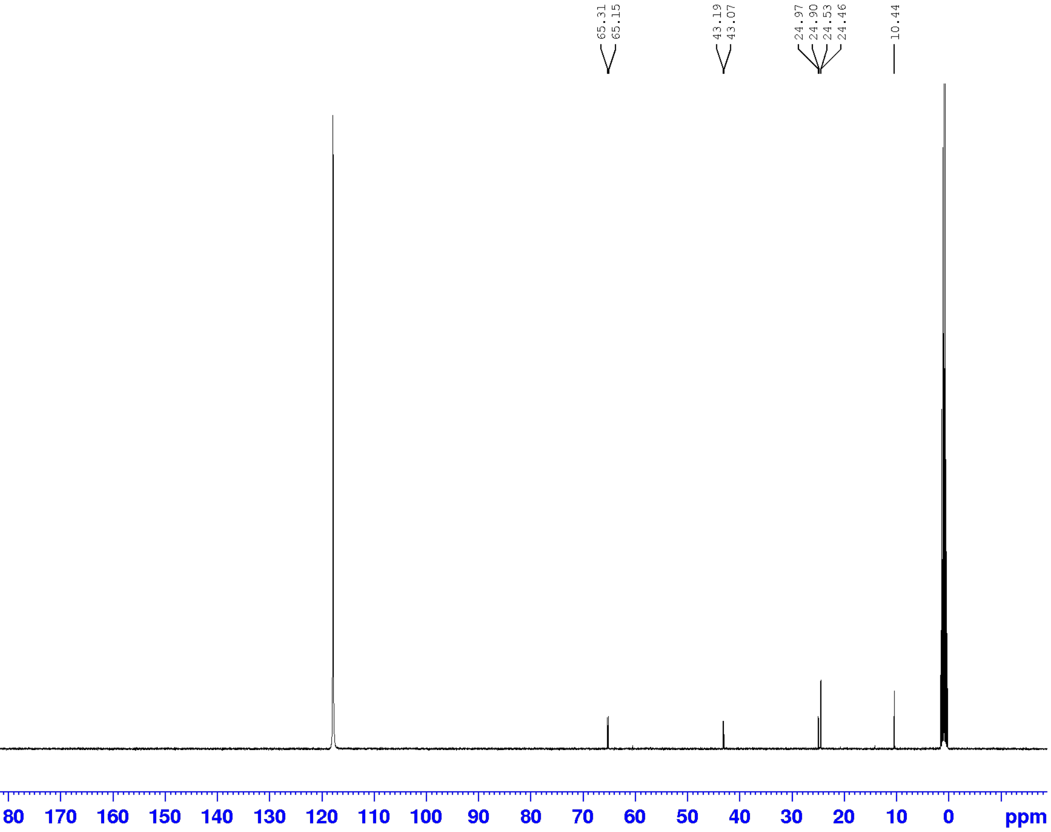

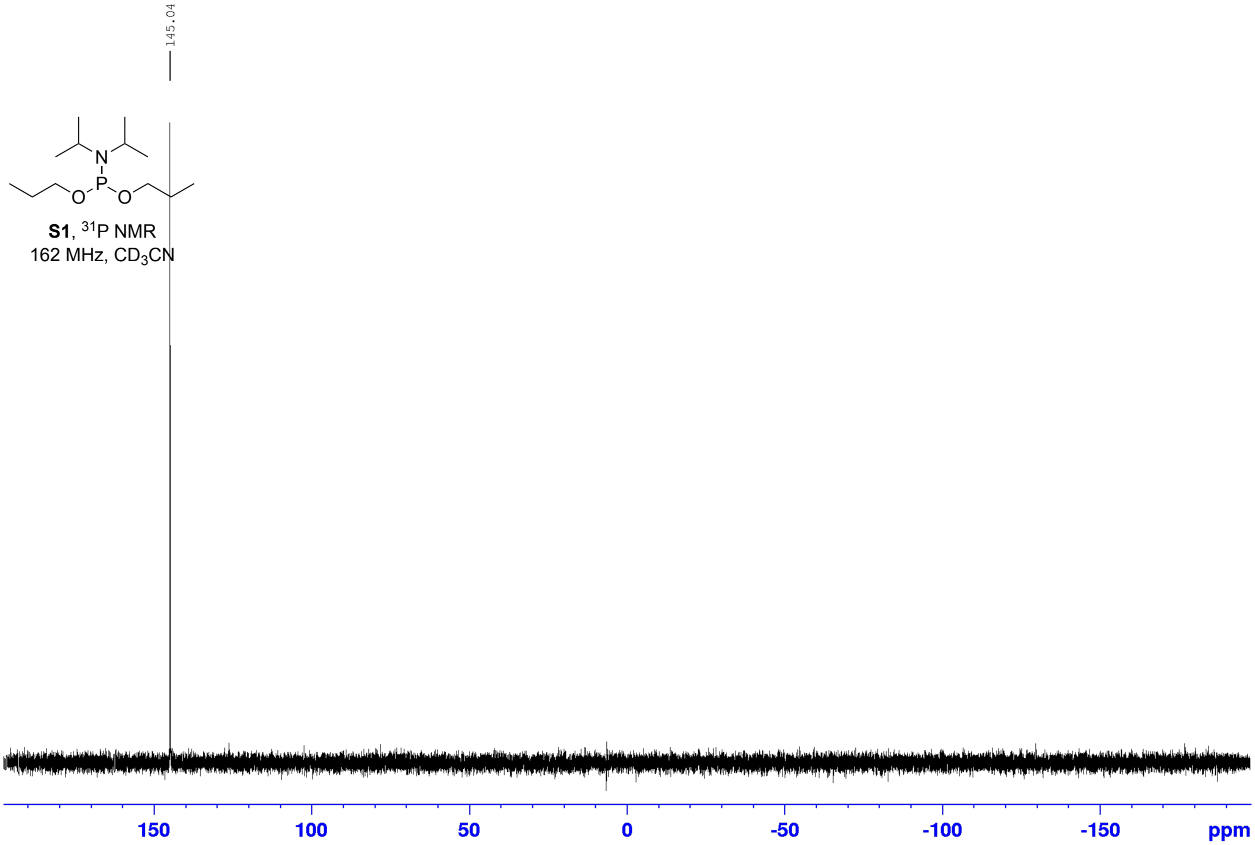
145.04
N
P
O
O
S1, 31P NMR
162 MHz, CD3CN
150
100
50
0
-50
-100
-150
ppm

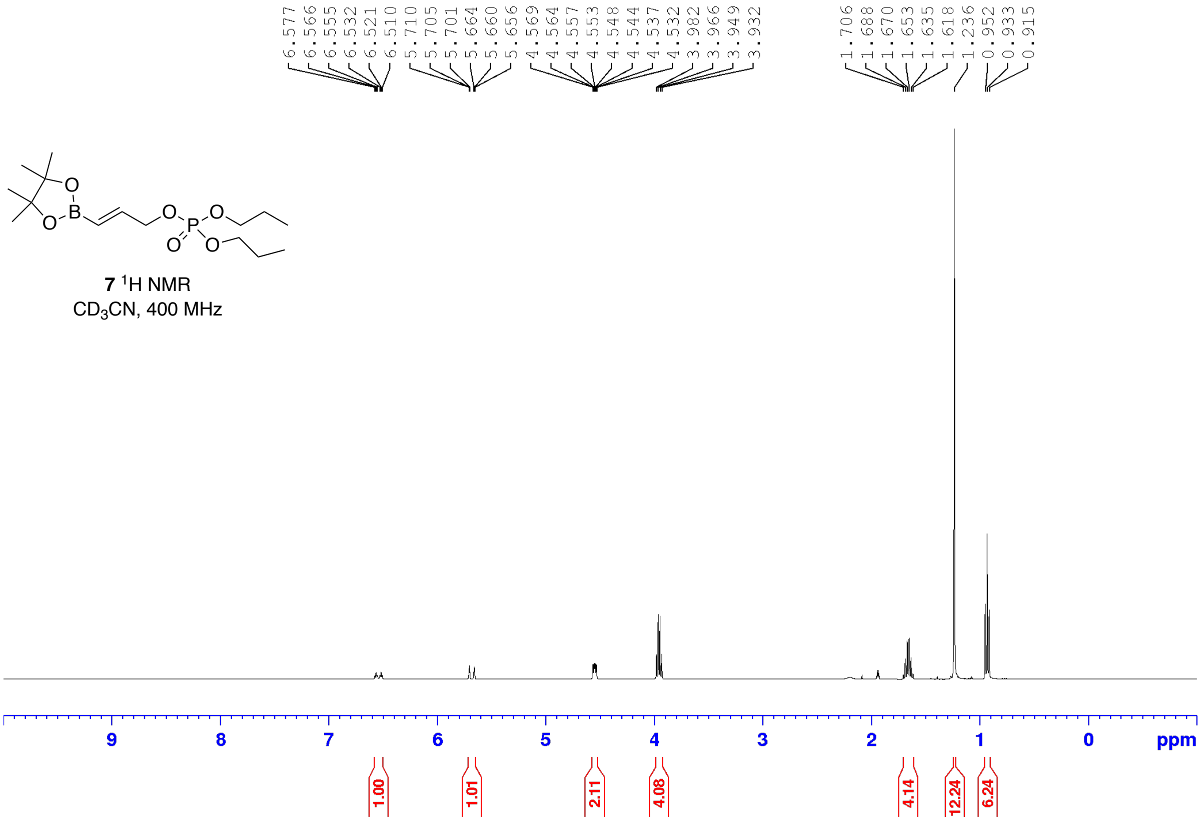

7 ^{1}H NMR
CD_3CN, 400 MHz

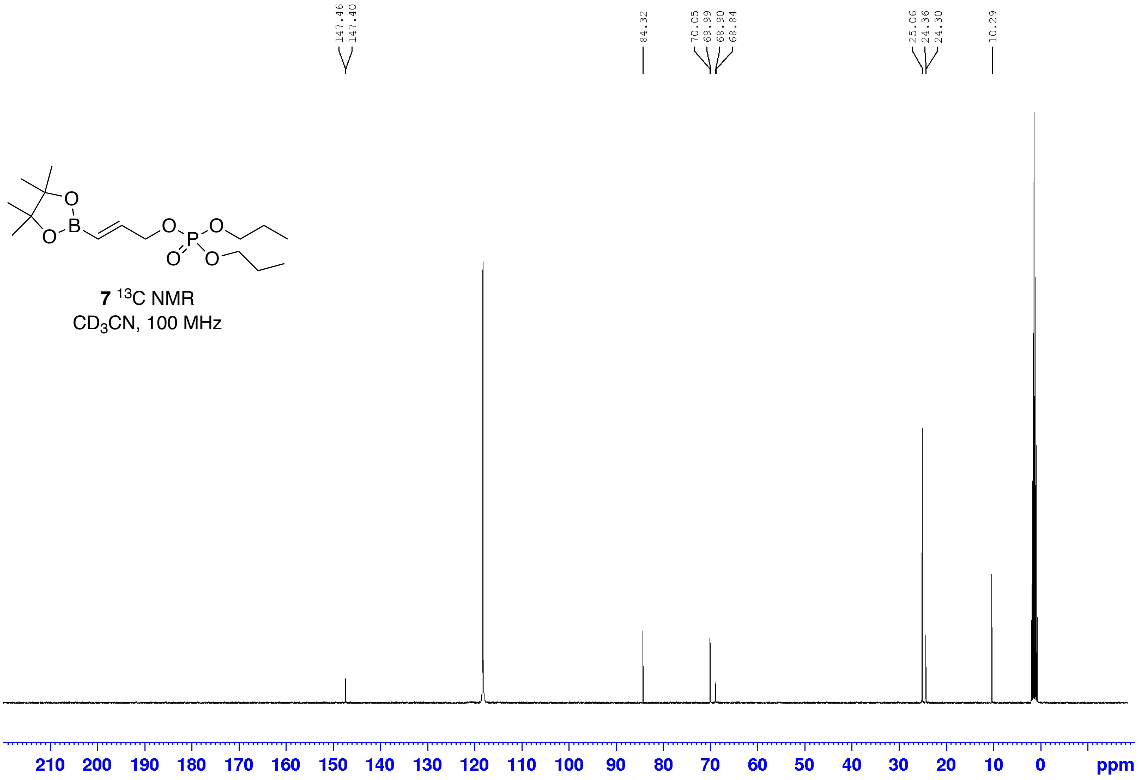

7 ^{13}C NMR
CD_3CN, 100 MHz

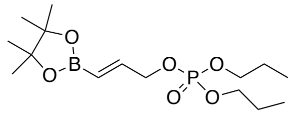

7 ^{31}P NMR
CD_3CN, 162 MHz

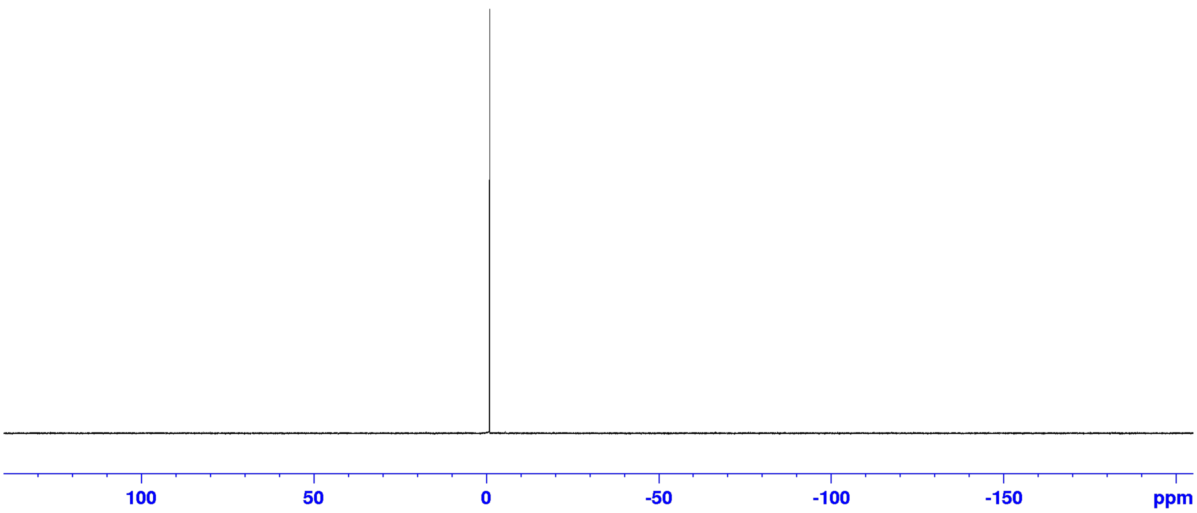

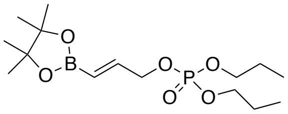

7 ^{11}B NMR
CD_3CN, 128 MHz

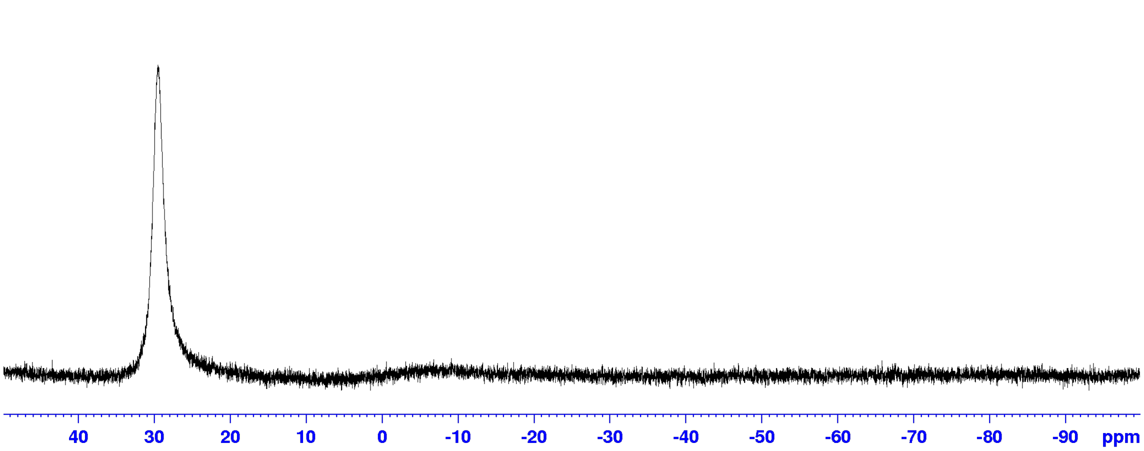

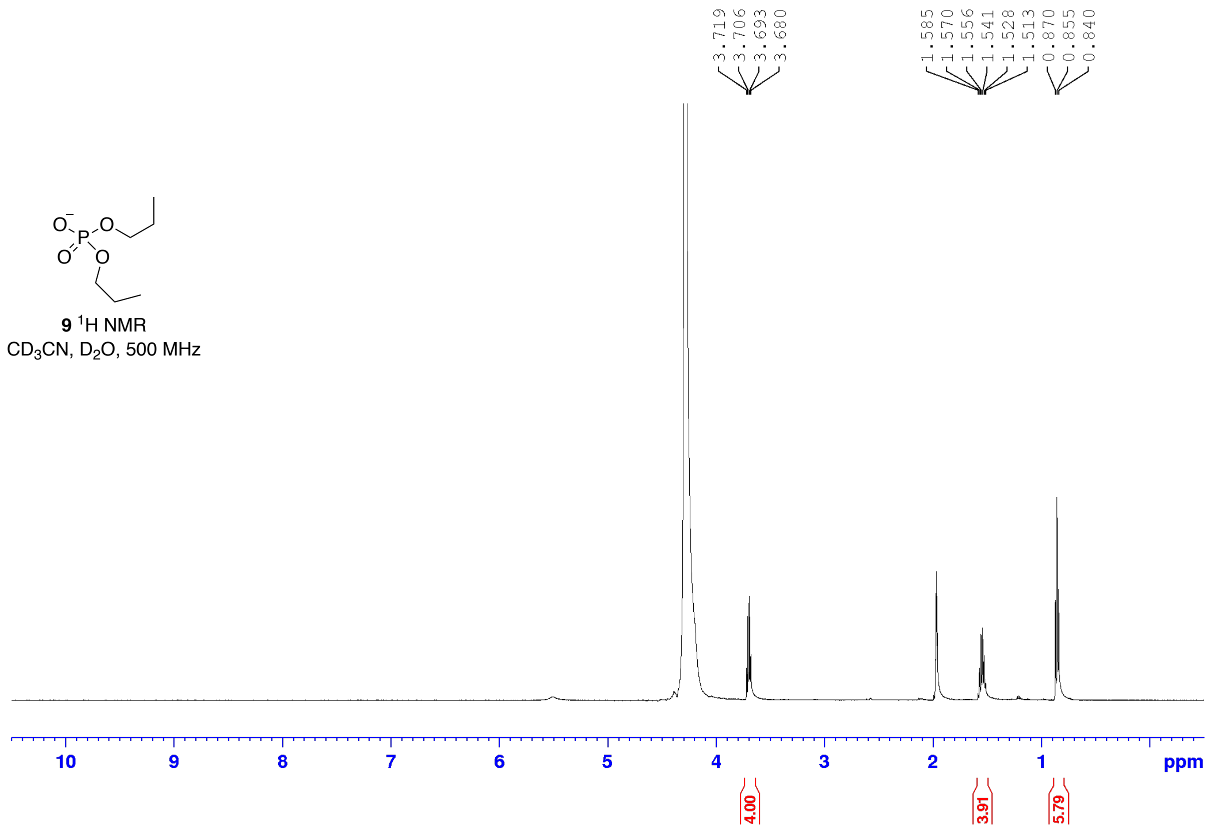
9 ¹H NMR
CD3CN, D2O, 500 MHz
3.719
3.706
3.693
3.680
1.585
1.570
1.556
1.541
1.528
1.513
0.870
0.855
0.840
10 9 8 7 6 5 4 3 2 1 ppm
4.00
3.91
5.79

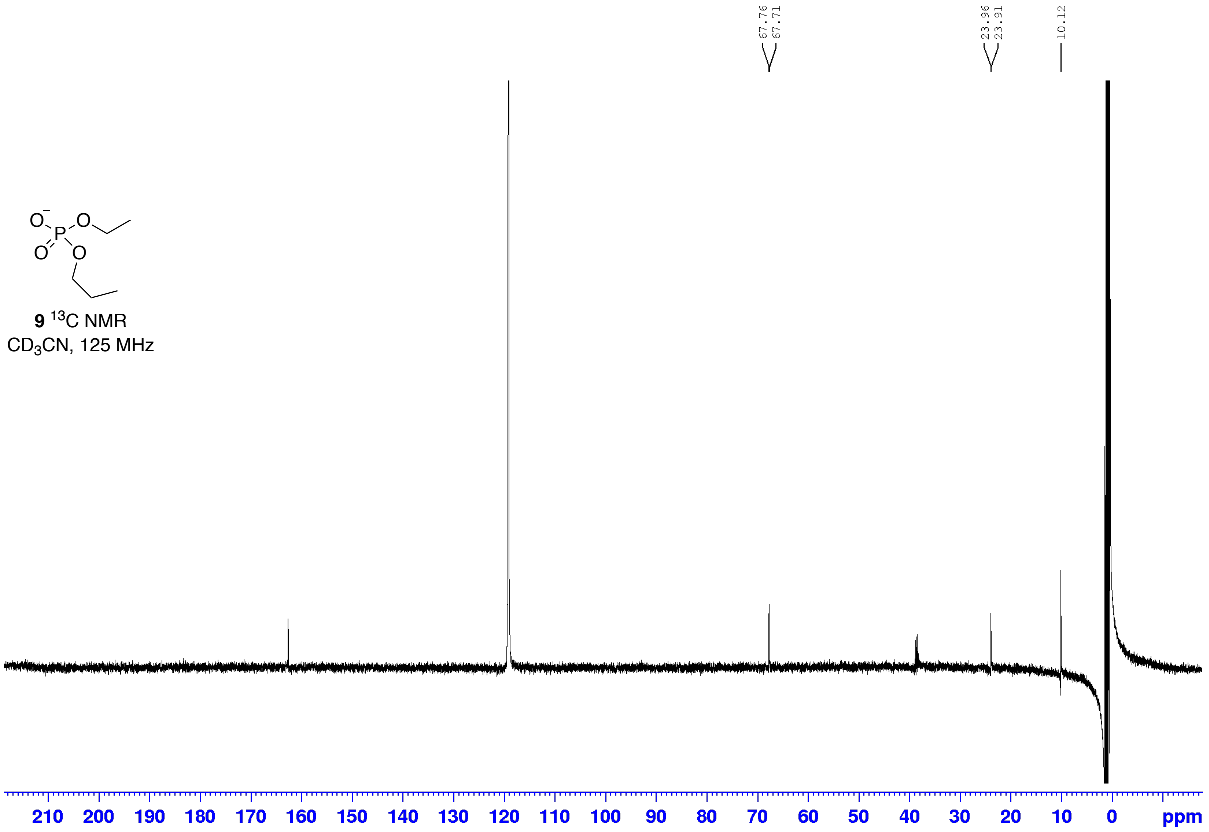
9 ¹³C NMR
CD3CN, 125 MHz
67.76
67.71
23.96
23.91
10.12
210 200 190 180 170 160 150 140 130 120 110 100 90 80 70 60 50 40 30 20 10 0 ppm

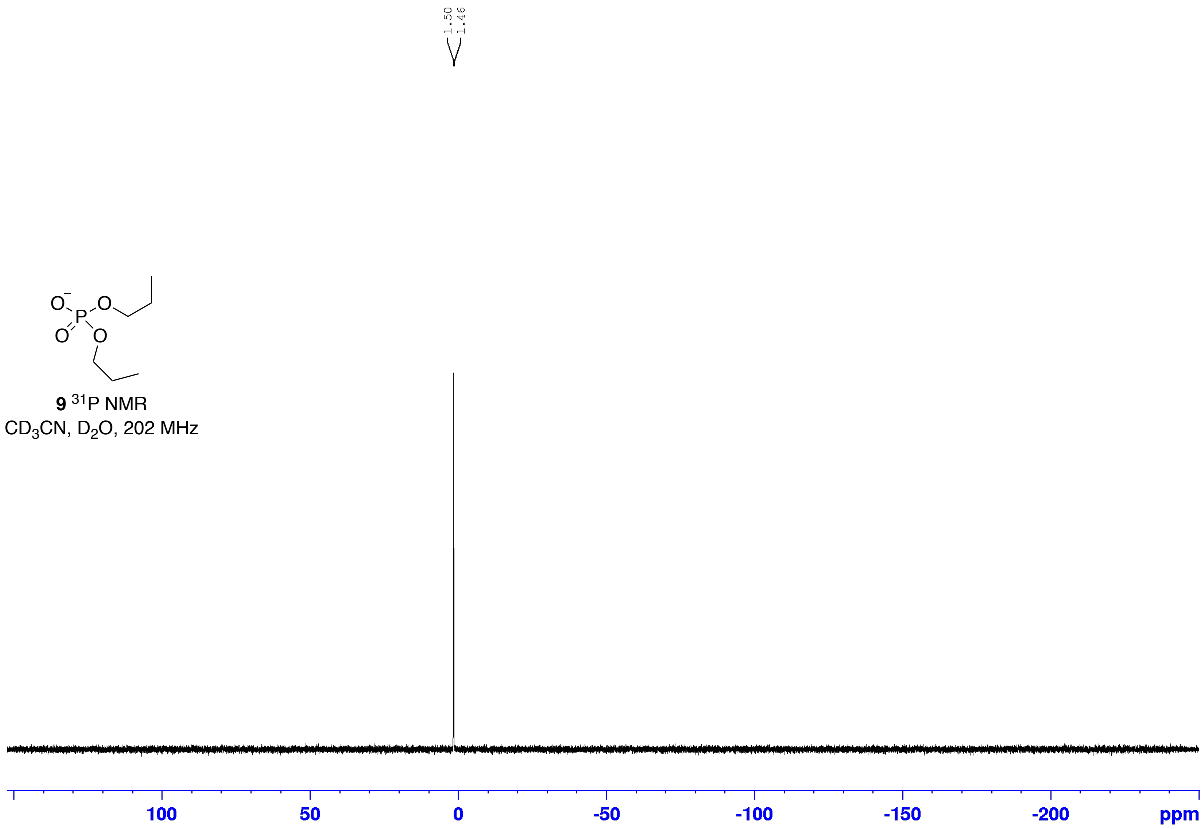
1.50
1.46
9 ^{31}P NMR
CD_3CN, D_2O, 202 MHz
100
50
0
-50
-100
-150
-200
ppm

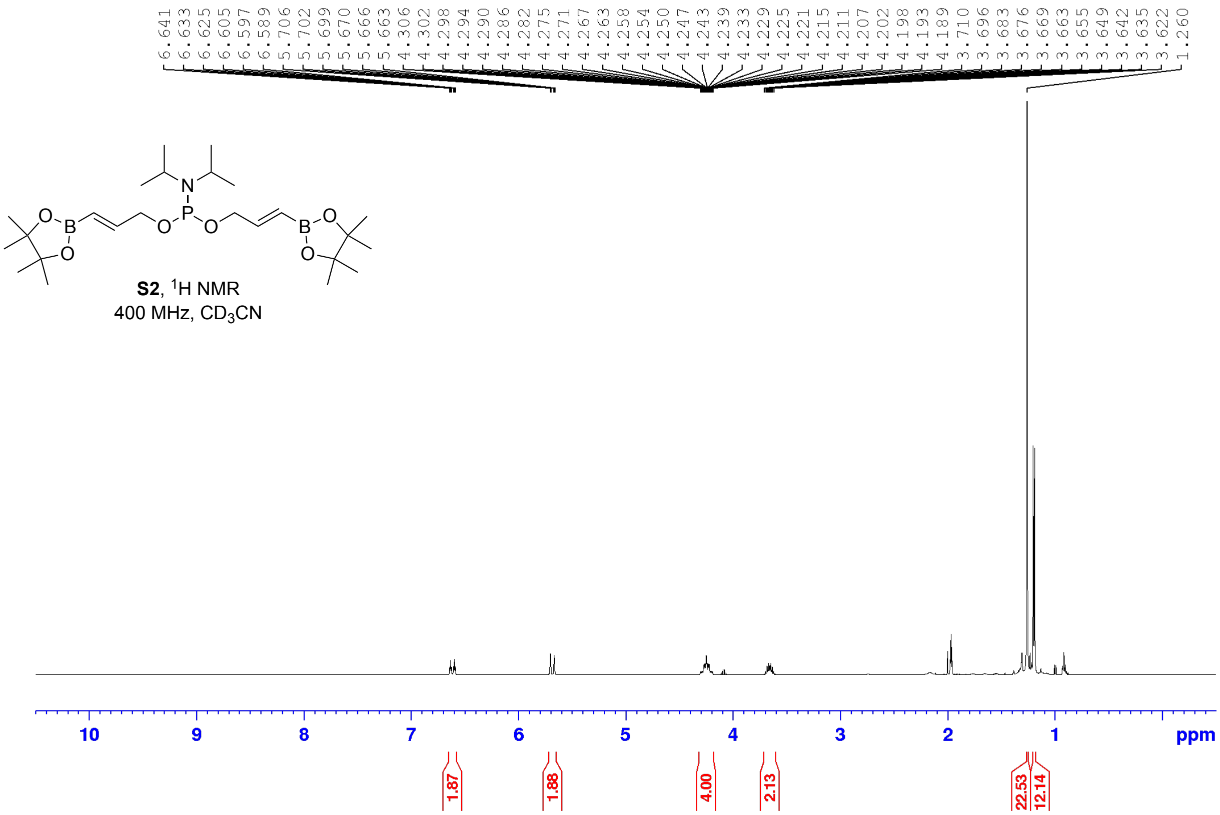
6.641 6.633 6.625 6.605 6.597 6.589 5.706 5.702 5.699 5.670 5.666 5.663 4.306 4.302 4.298 4.294 4.290 4.286 4.282 4.275 4.271 4.267 4.263 4.258 4.254 4.250 4.247 4.243 4.239 4.233 4.229 4.225 4.221 4.215 4.211 4.207 4.202 4.198 4.193 4.189 3.710 3.696 3.683 3.676 3.669 3.663 3.655 3.649 3.642 3.635 3.622 1.260
S2, 1H NMR
400 MHz, CD3CN
10 9 8 7 6 5 4 3 2 1 ppm
1.87 1.88 4.00 2.13 22.53 12.14

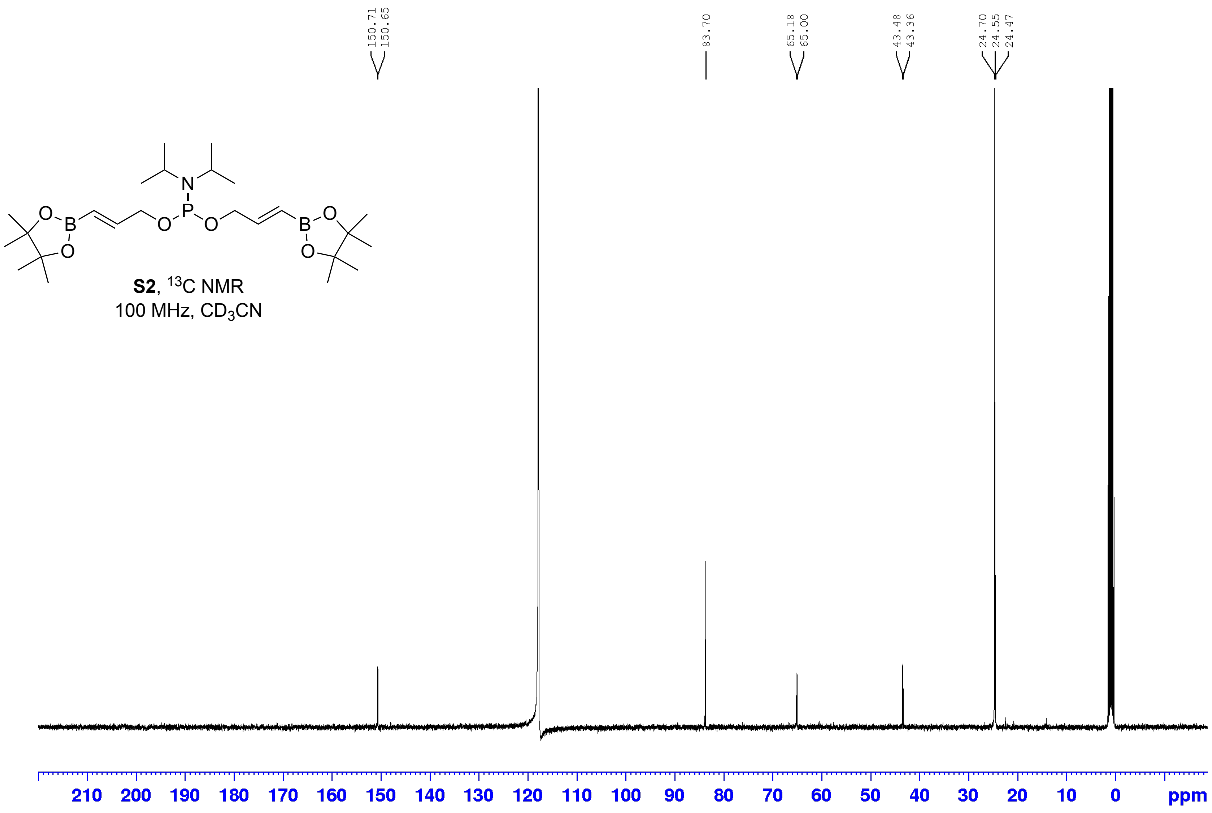
150.71 150.65 83.70 65.18 65.00 43.48 43.36 24.70 24.55 24.47
S2, 13C NMR
100 MHz, CD3CN
210 200 190 180 170 160 150 140 130 120 110 100 90 80 70 60 50 40 30 20 10 0 ppm

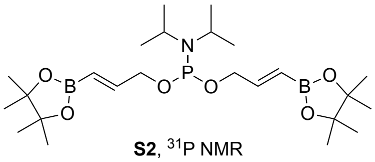

S2, ^{31}P NMR
202 MHz, CD_3CN

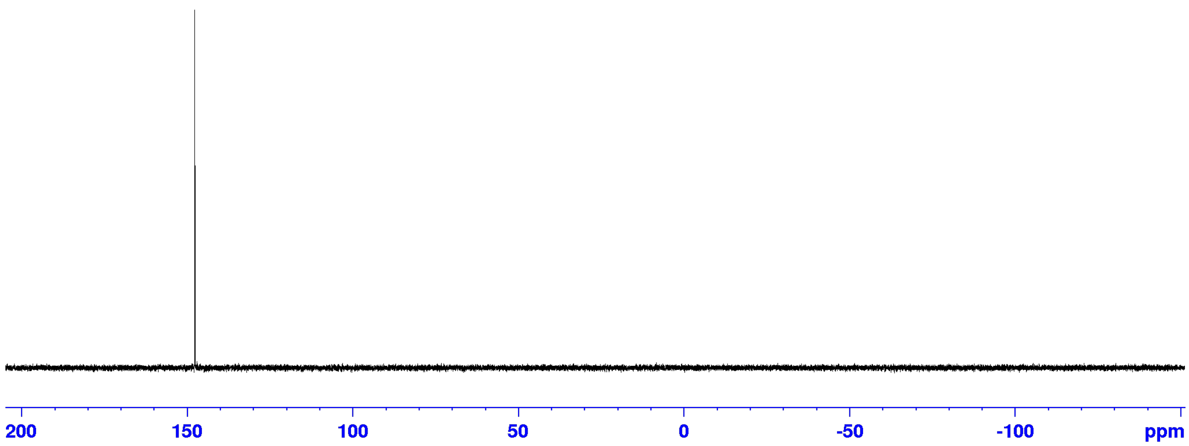

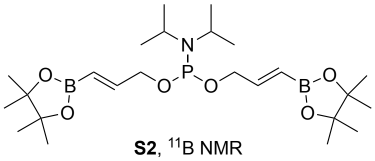

S2, ^{11}B NMR
128 MHz, CD_3CN

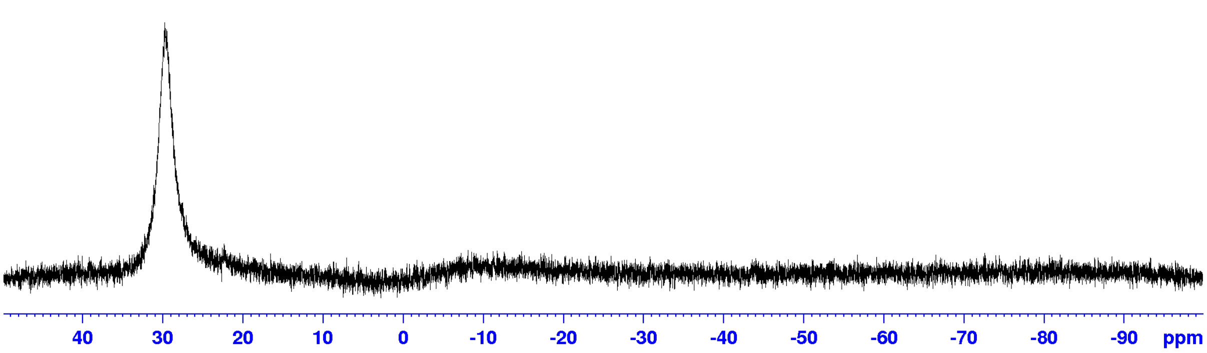

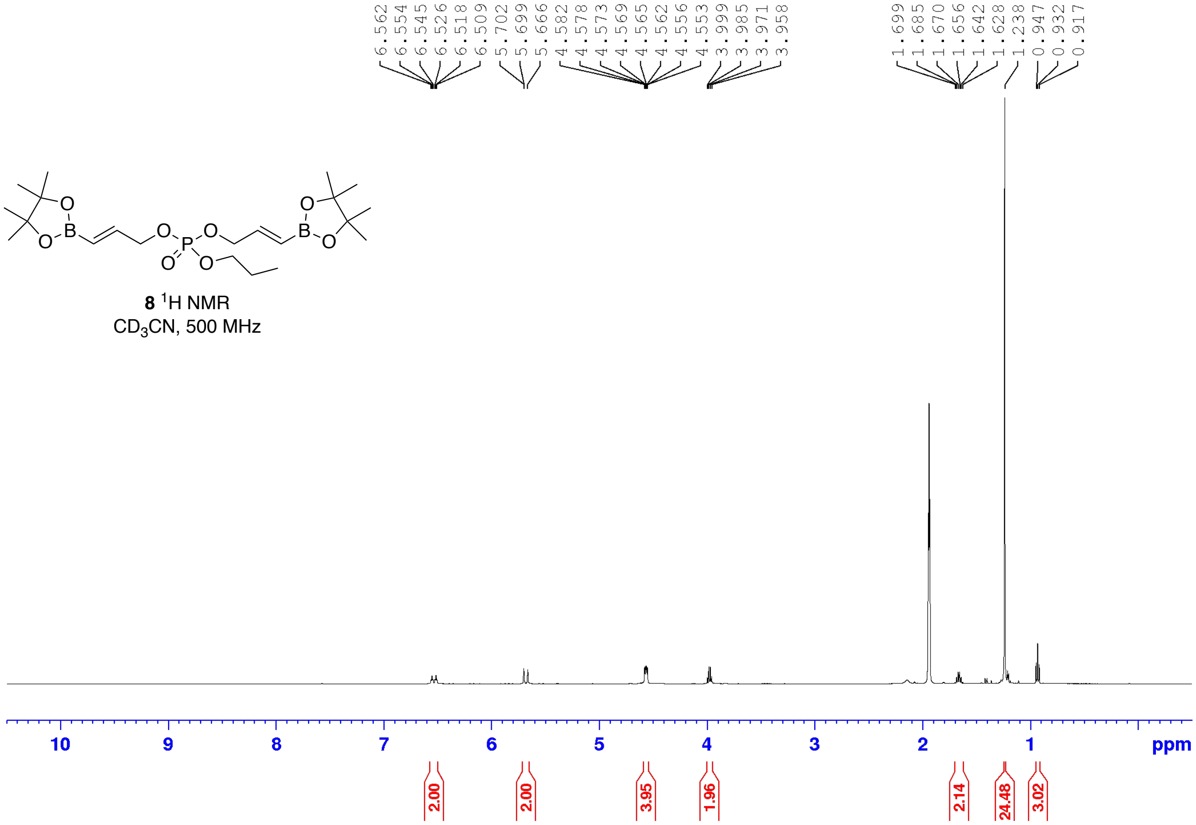

8 ^{1}H NMR
CD_3CN, 500 MHz

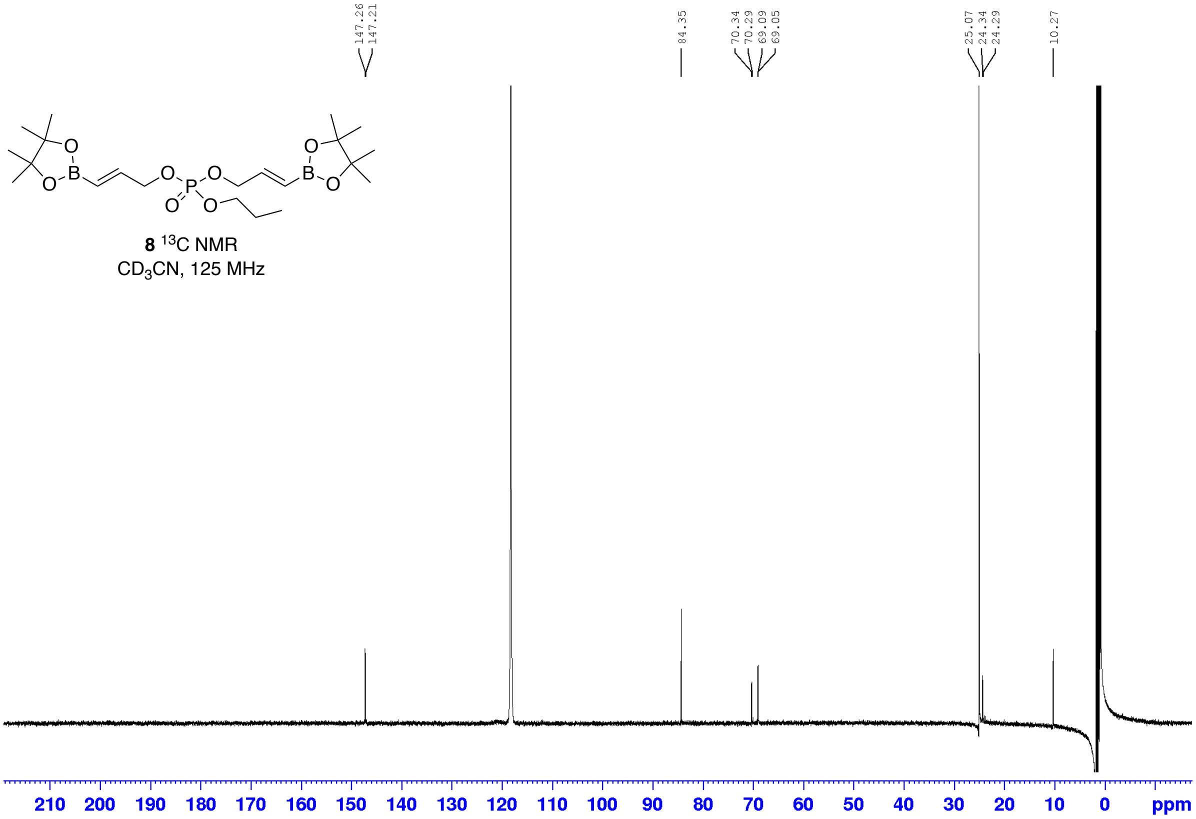

8 ^{13}C NMR
CD_3CN, 125 MHz

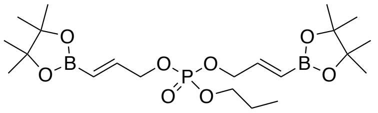

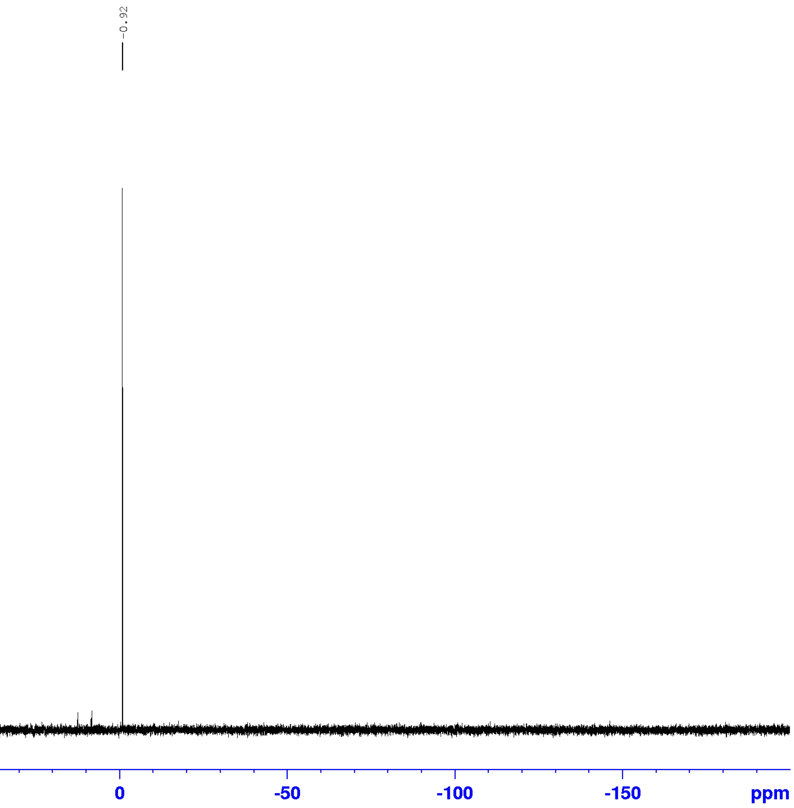

8 ^{31}P NMR
CD_3CN, 202 MHz

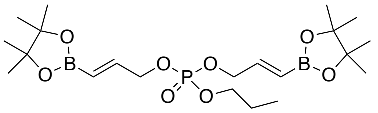

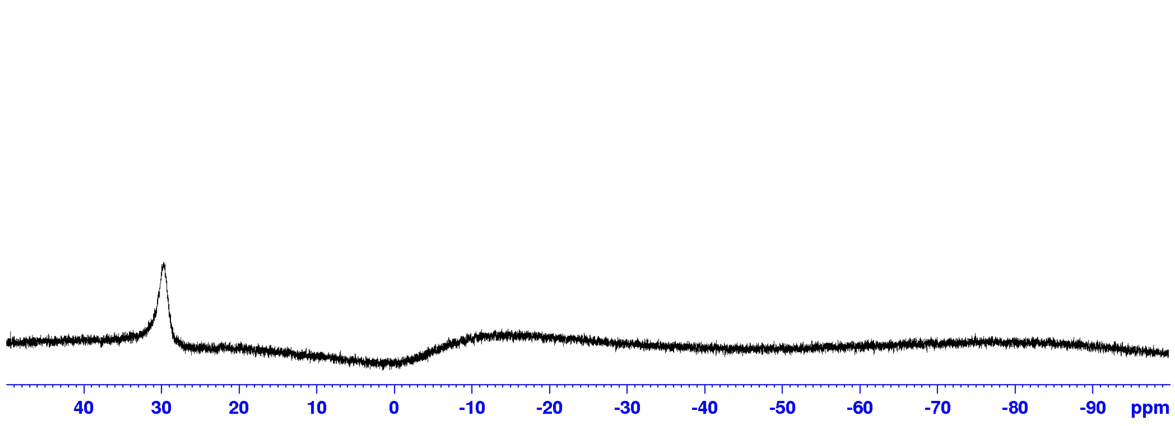

8 ^{11}B NMR
CD_3CN, 160 MHz

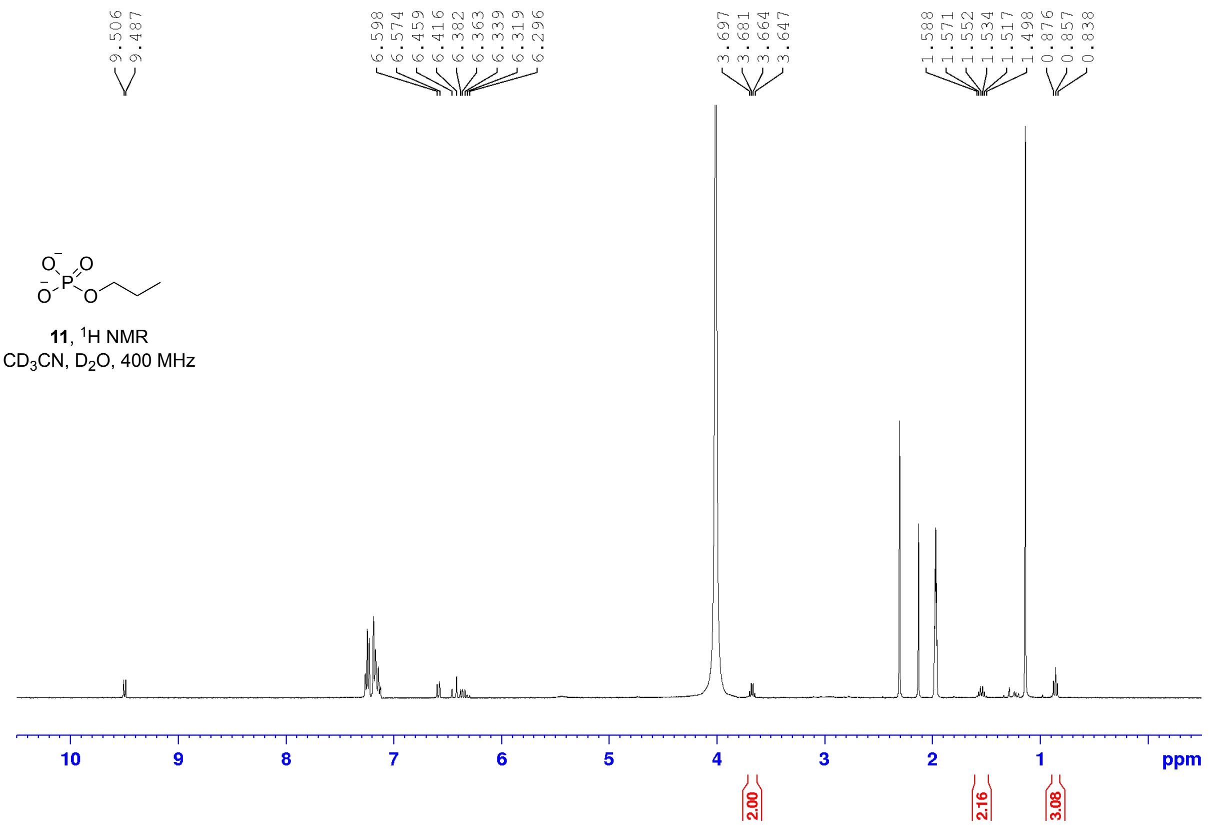
9.506
9.487
6.598
6.574
6.459
6.416
6.382
6.363
6.339
6.319
6.296
3.697
3.681
3.664
3.647
1.588
1.571
1.552
1.534
1.517
1.498
0.876
0.857
0.838
11, 1H NMR
CD3CN, D2O, 400 MHz
10 9 8 7 6 5 4 3 2 1 ppm
2.00
2.16
3.08

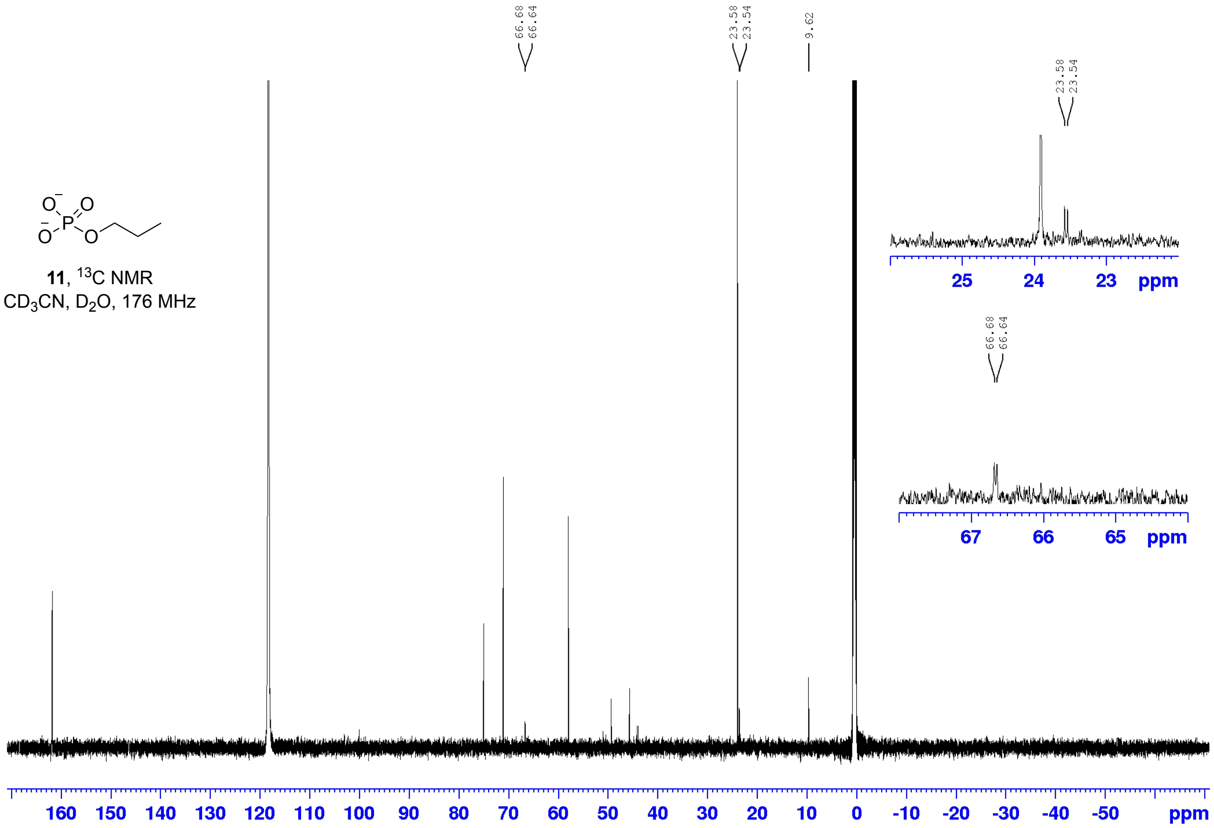
66.68
66.64
23.58
23.54
9.62
11, 13C NMR
CD3CN, D2O, 176 MHz
25 24 23 ppm
67 66 65 ppm
160 150 140 130 120 110 100 90 80 70 60 50 40 30 20 10 0 -10 -20 -30 -40 -50 ppm

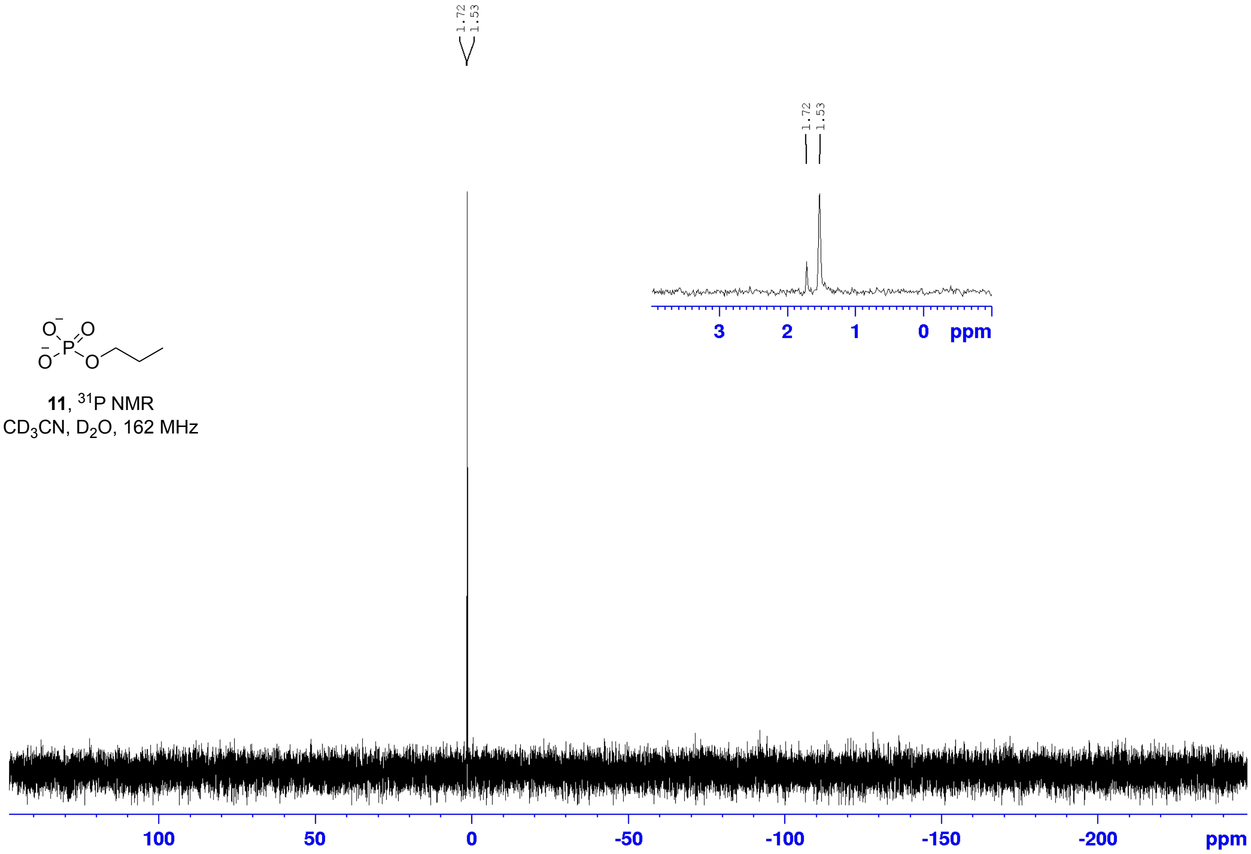

11, ^{31}P NMR
CD_3CN, D_2O, 162 MHz

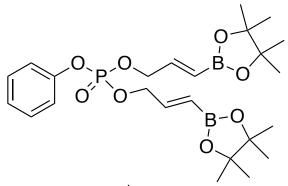

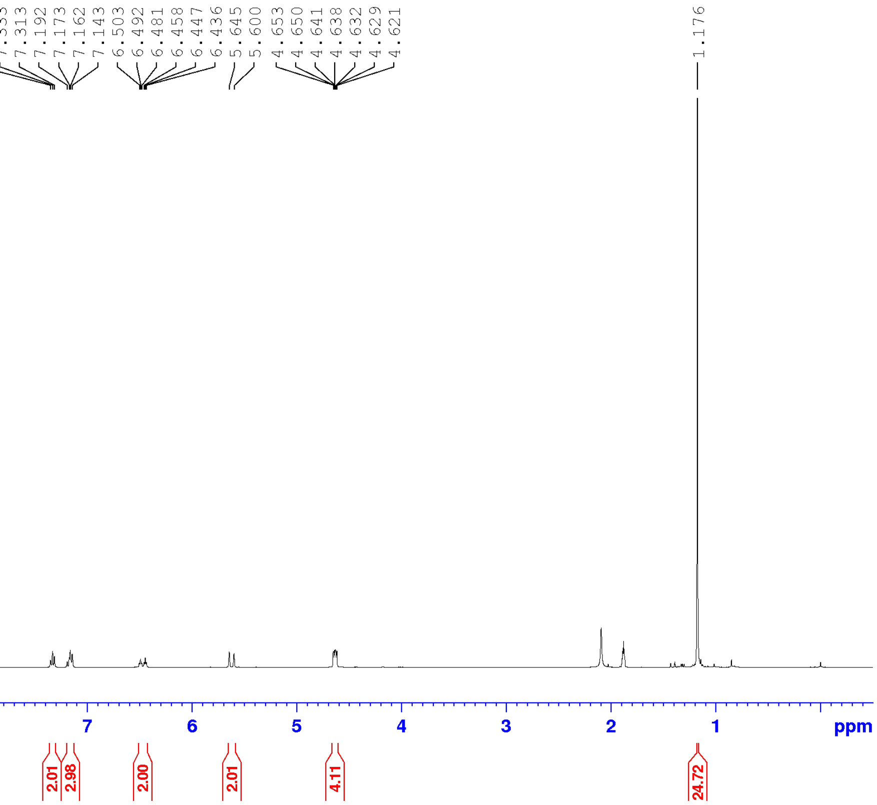

12 ^{1}H NMR
CD_3CN, 400 MHz

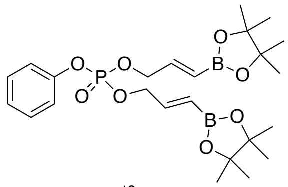

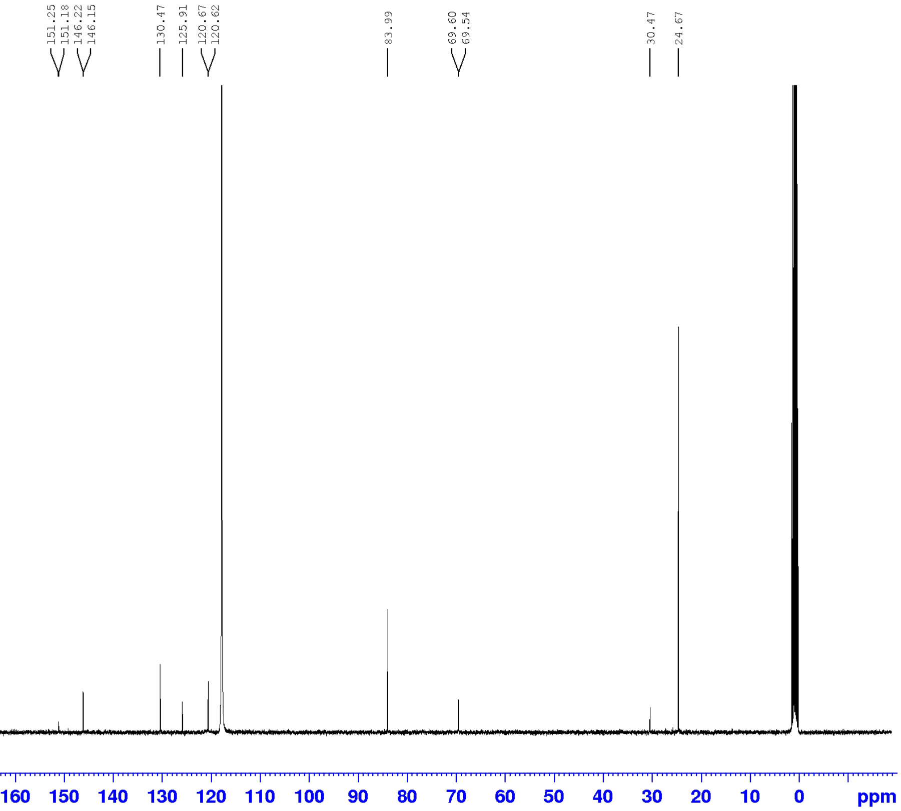

12 ^{13}C NMR
CD_3CN, 100 MHz

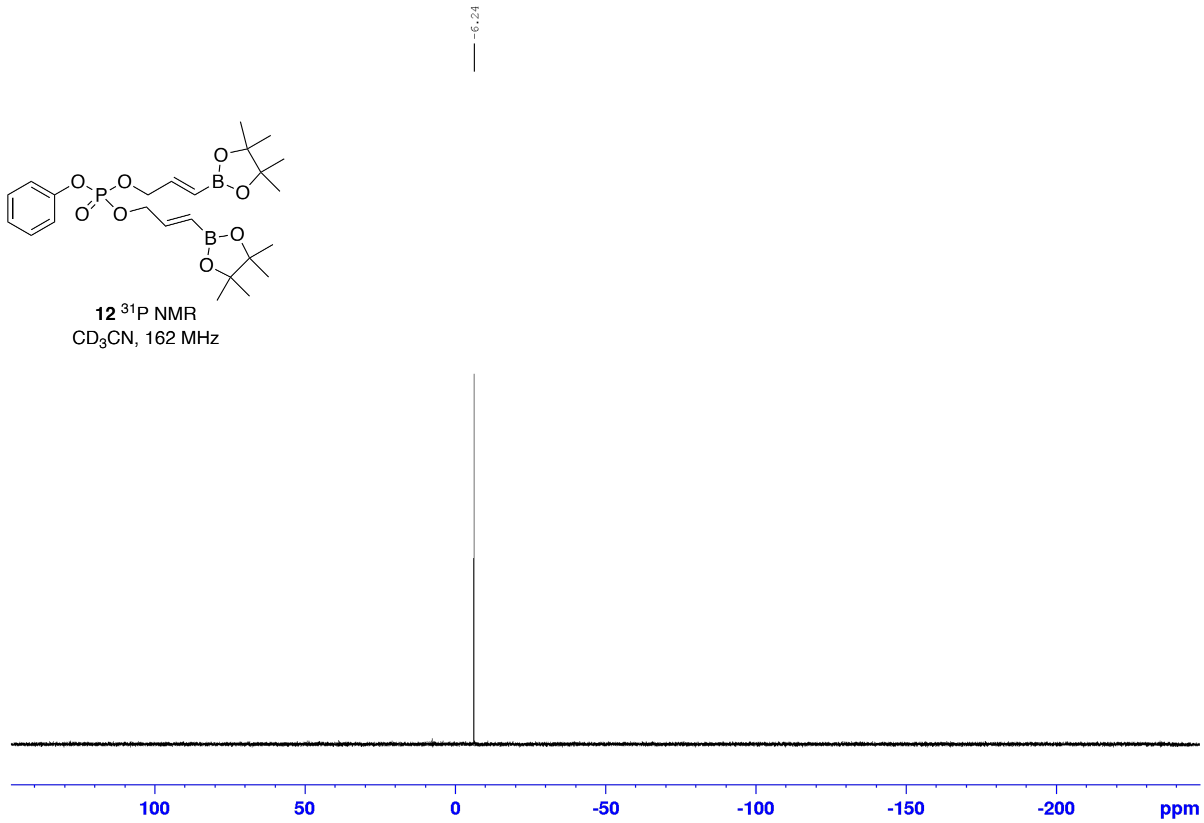
-6.24
12 ^{31}P NMR
CD_3CN, 162 MHz
100
50
0
-50
-100
-150
-200
ppm

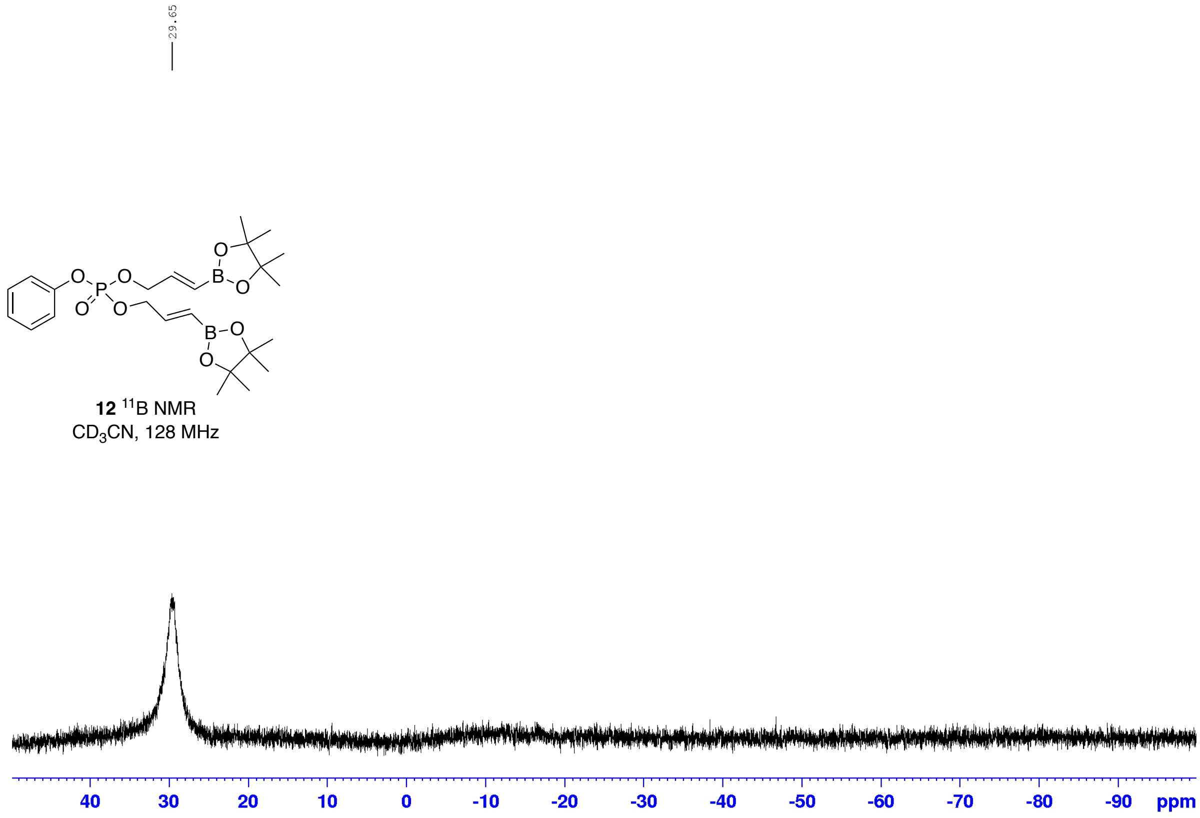
29.65
12 ^{11}B NMR
CD_3CN, 128 MHz
40
30
20
10
0
-10
-20
-30
-40
-50
-60
-70
-80
-90
ppm

13 ^{1}H NMR
CD_3CN, D_2O, 300 MHz

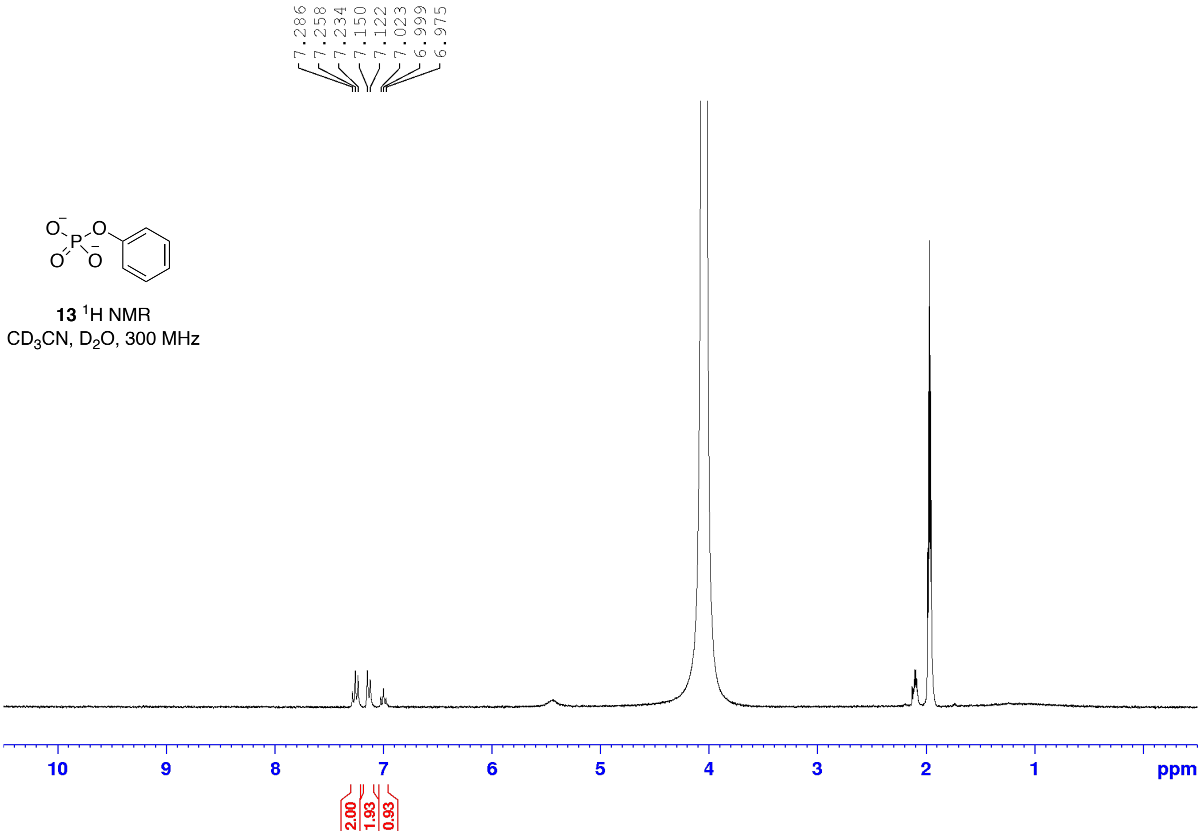

13 ^{13}C NMR
CD_3CN, D_2O, 75 MHz

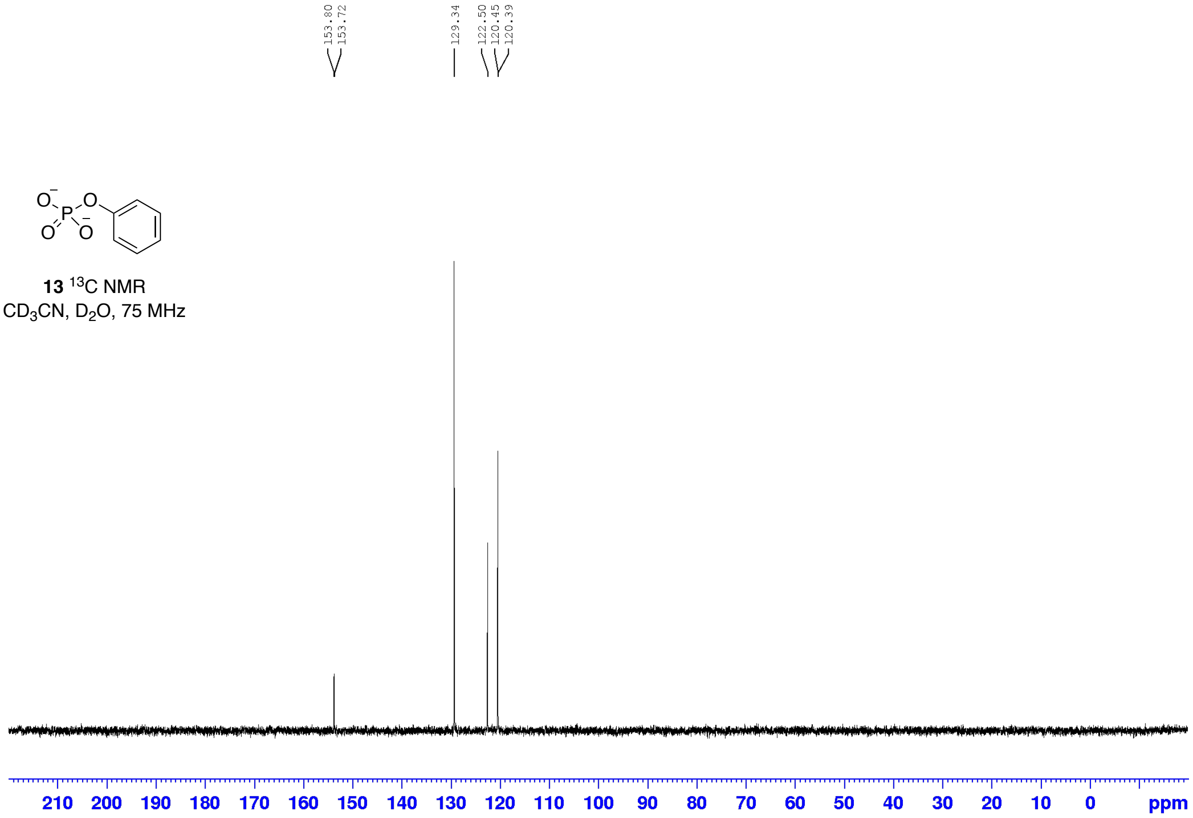

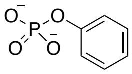

13 ^{31}P NMR
CD_3CN, D_2O, 122 MHz

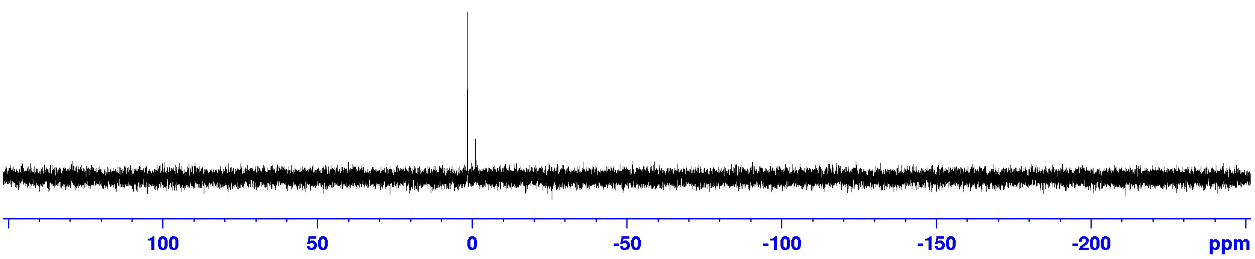

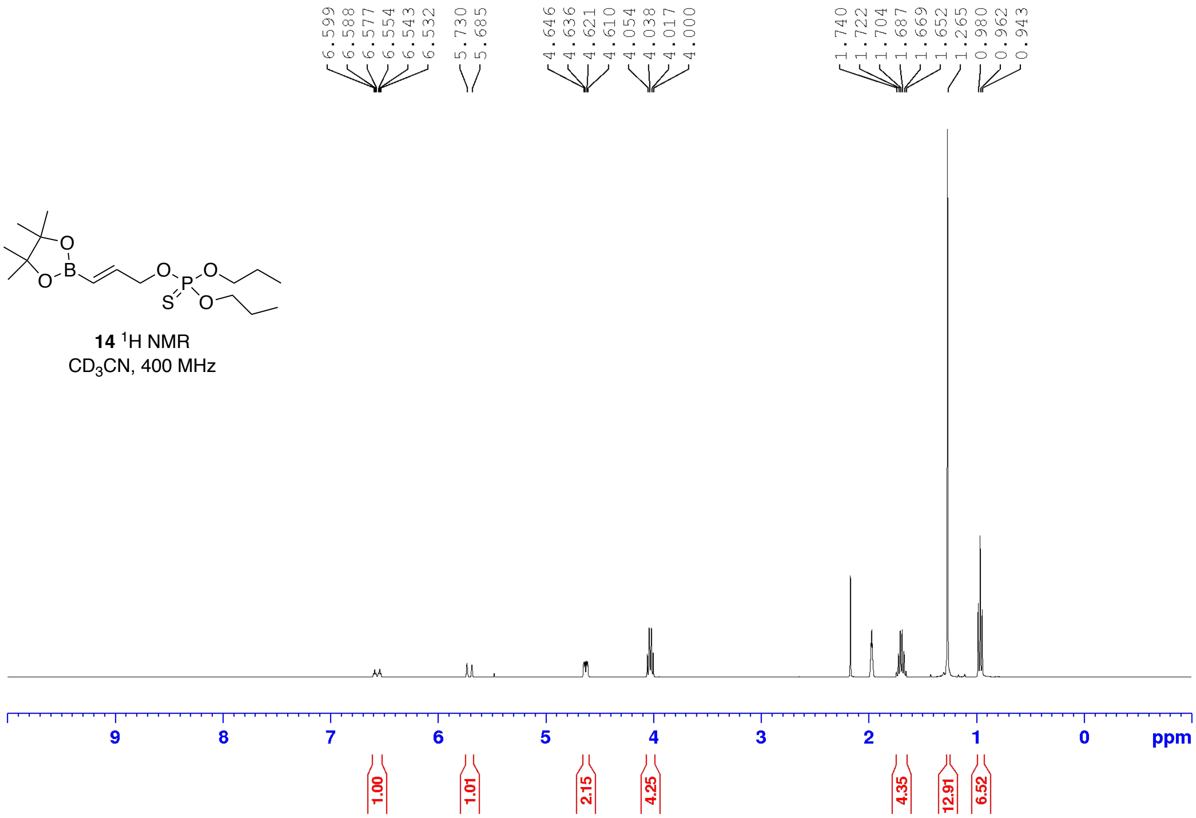

14 ^{1}H NMR
CD_3CN, 400 MHz

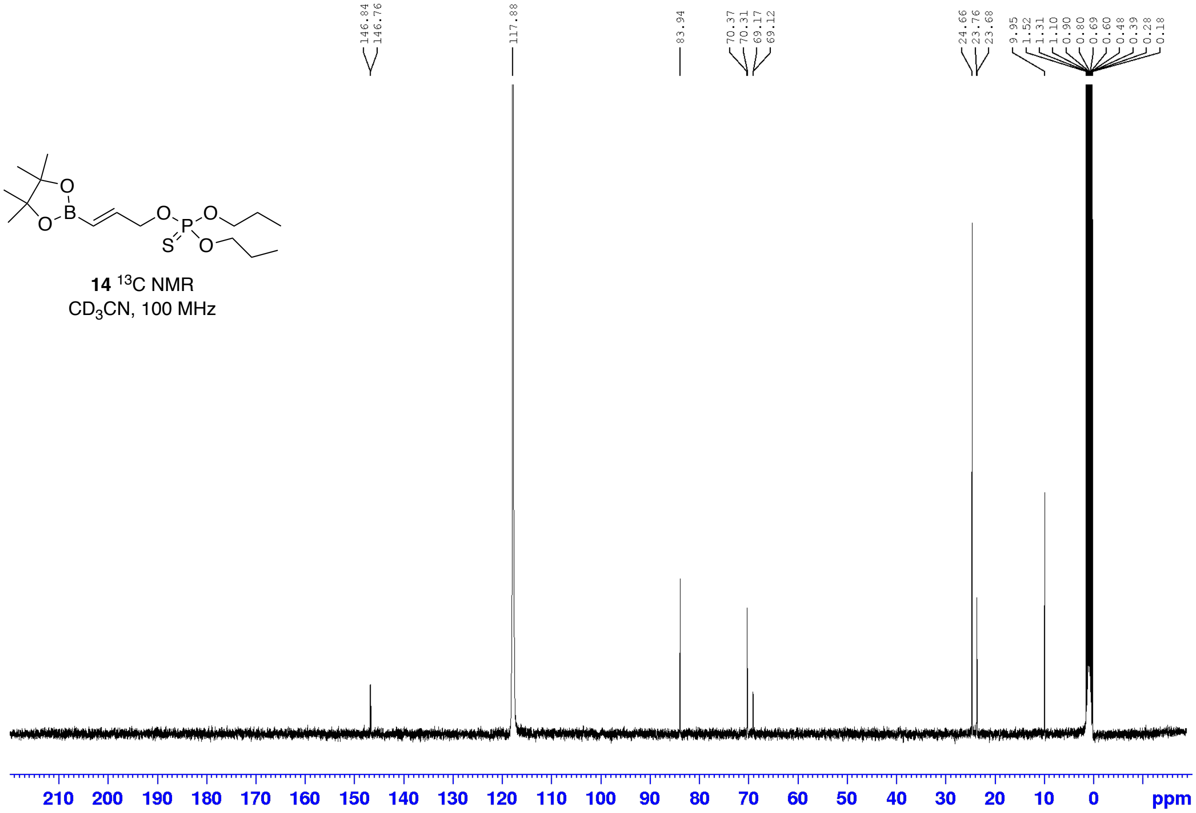

14 ^{13}C NMR
CD_3CN, 100 MHz

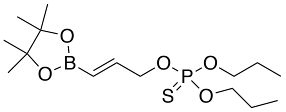

14 ^{31}P NMR
CD_3CN, 162 MHz

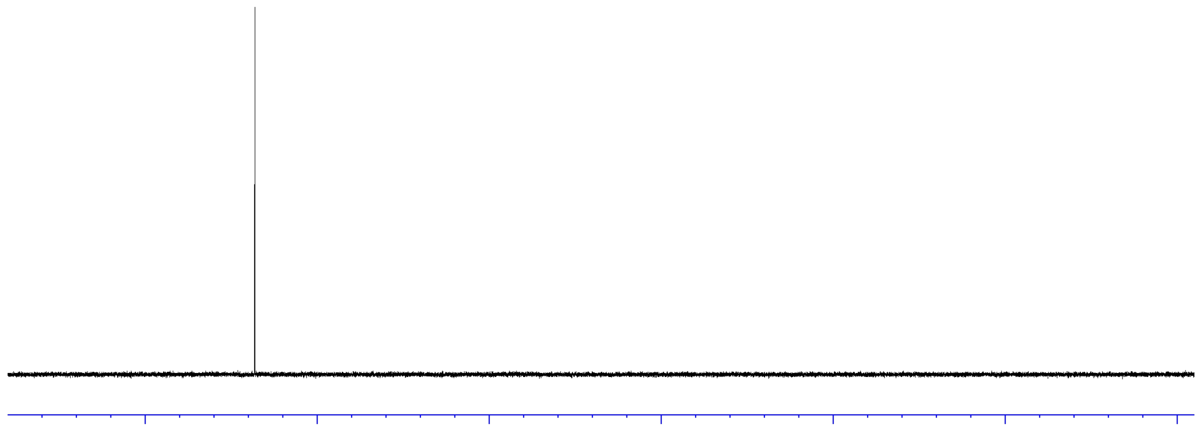

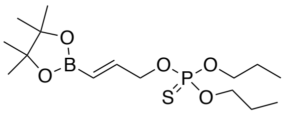

14 ^{11}B NMR
CD_3CN, 128 MHz

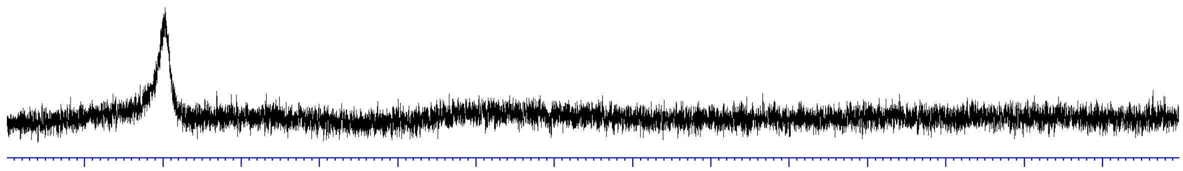

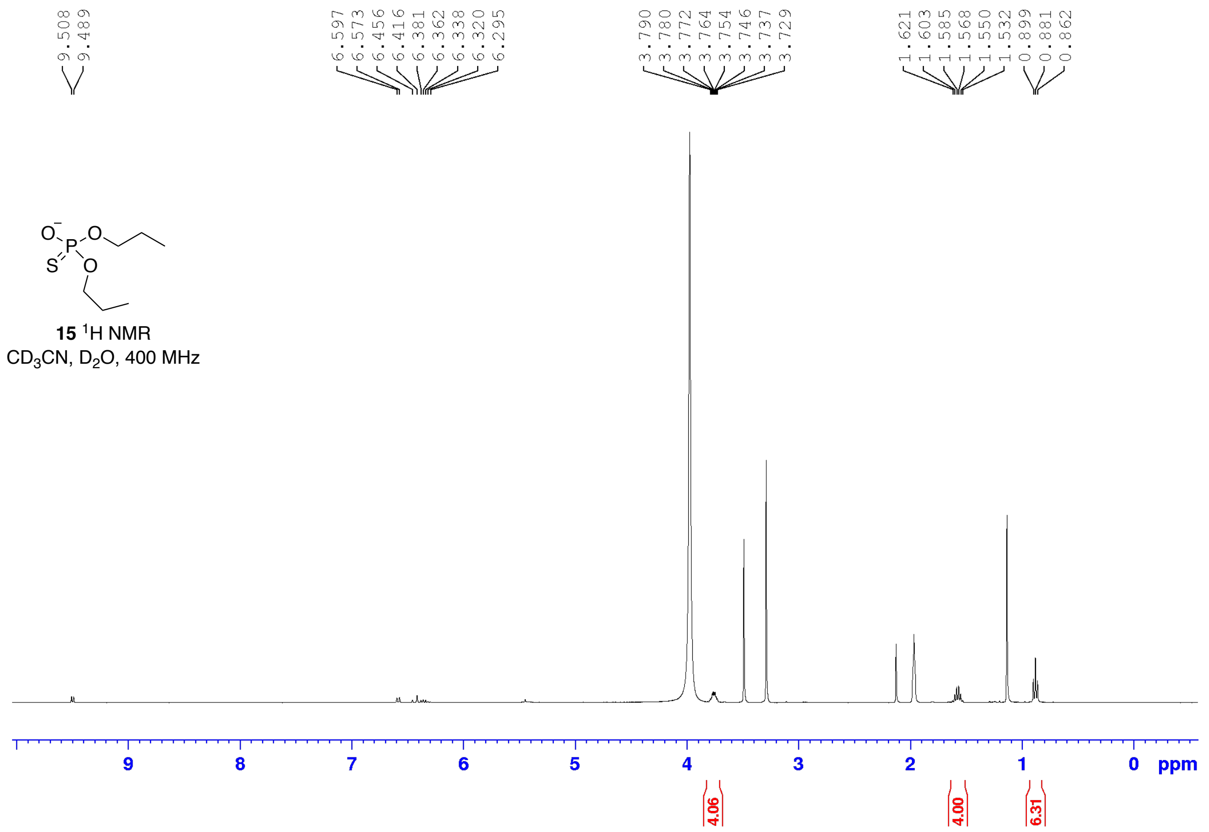

9.508
9.489
6.597
6.573
6.456
6.416
6.381
6.362
6.338
6.320
6.295
3.790
3.780
3.772
3.764
3.754
3.746
3.737
3.729
1.621
1.603
1.585
1.568
1.550
1.532
0.899
0.881
0.862
15 1H NMR
CD3CN, D2O, 400 MHz
9 8 7 6 5 4 3 2 1 0 ppm
4.06
4.00
6.31

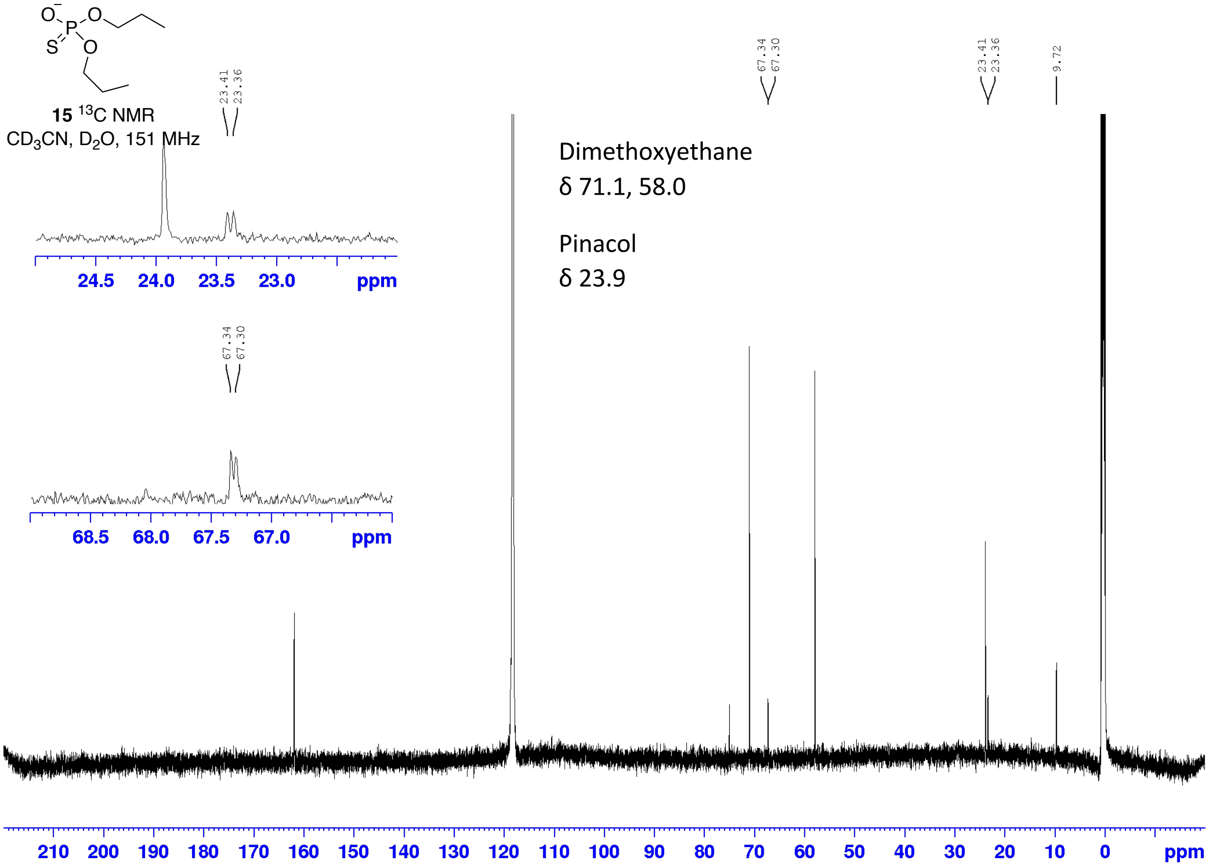

15 13C NMR
CD3CN, D2O, 151 MHz
23.41
23.36
24.5 24.0 23.5 23.0 ppm
67.34
67.30
68.5 68.0 67.5 67.0 ppm
9.72
Dimethoxyethane
δ 71.1, 58.0
Pinacol
δ 23.9
210 200 190 180 170 160 150 140 130 120 110 100 90 80 70 60 50 40 30 20 10 0 ppm

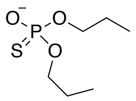

15 ^{31}P NMR
CD_3CN, D_2O, 162 MHz

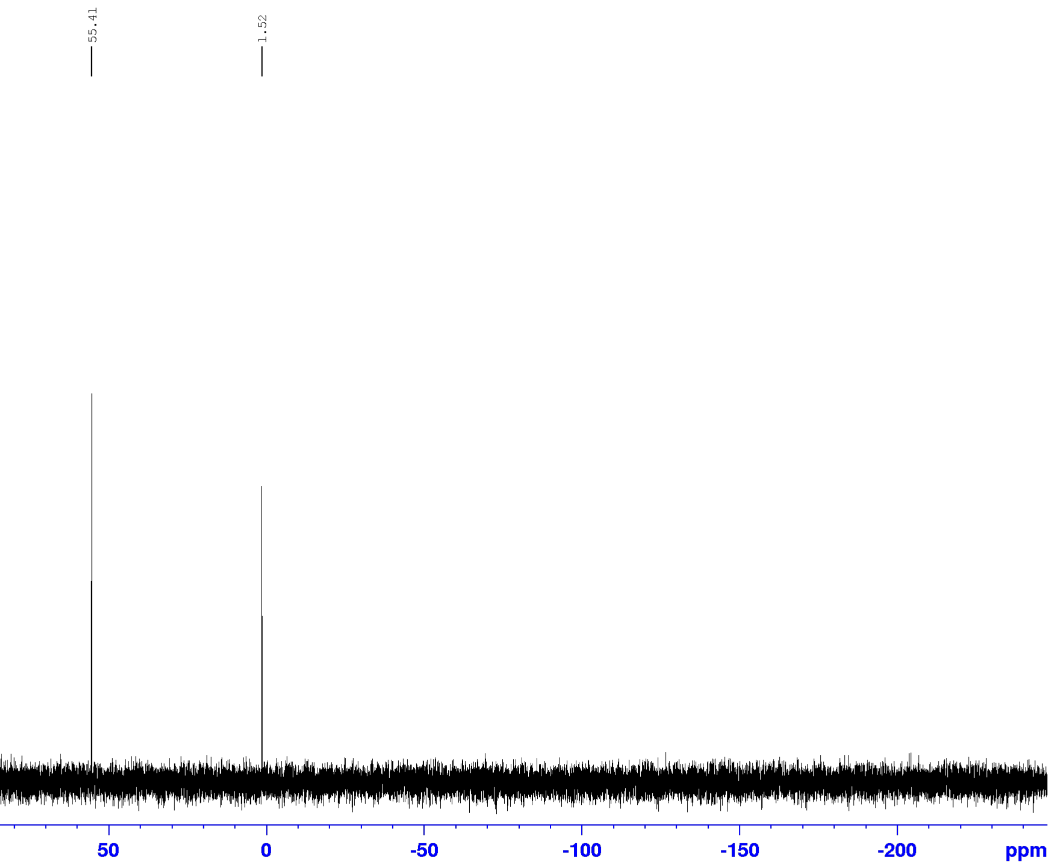

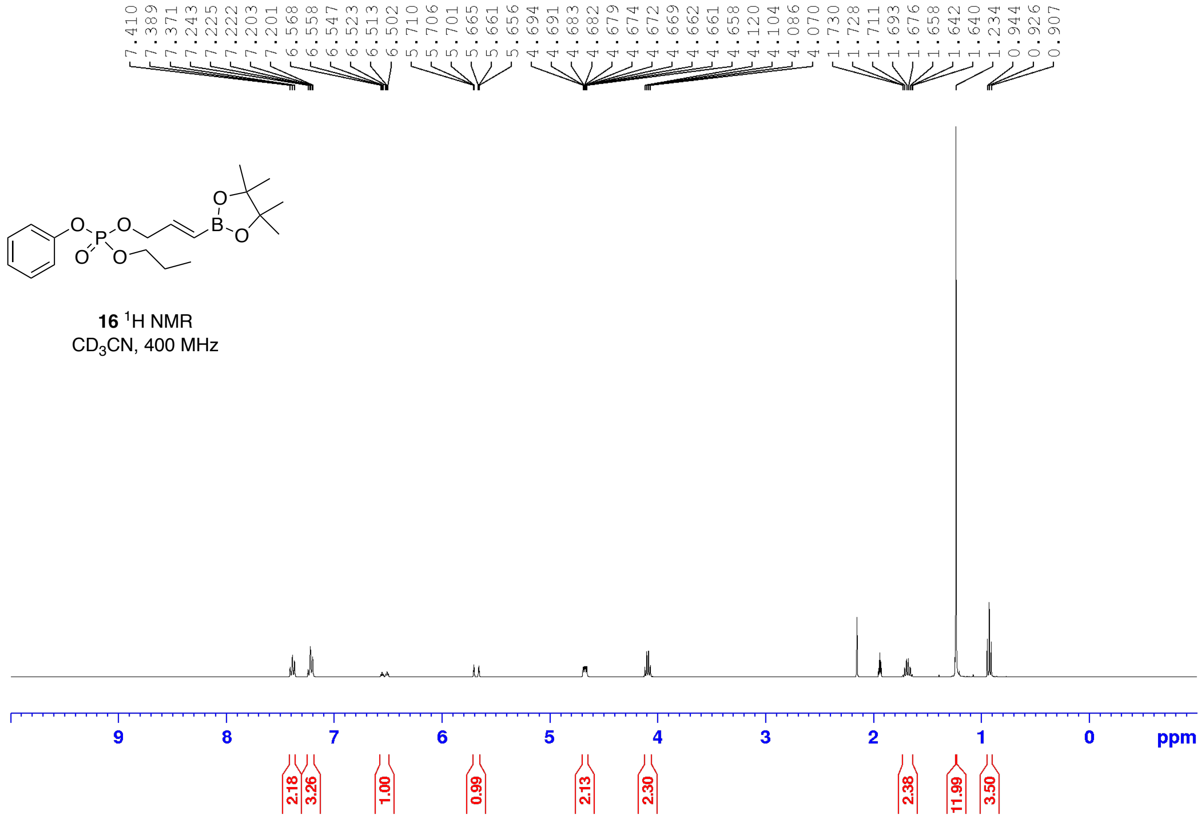
16 ^{1}H NMR
CD_3CN, 400 MHz
7.410 7.389 7.371 7.243 7.225 7.222 7.203 7.201 6.568 6.558 6.547 6.523 6.513 6.502 5.710 5.706 5.701 5.665 5.661 5.656 4.694 4.691 4.683 4.682 4.679 4.674 4.672 4.669 4.662 4.661 4.658 4.120 4.104 4.086 4.070 1.730 1.728 1.711 1.693 1.676 1.658 1.642 1.640 1.234 0.944 0.926 0.907
2.18 3.26 1.00 0.99 2.13 2.30 2.38 11.99 3.50
9 8 7 6 5 4 3 2 1 0 ppm

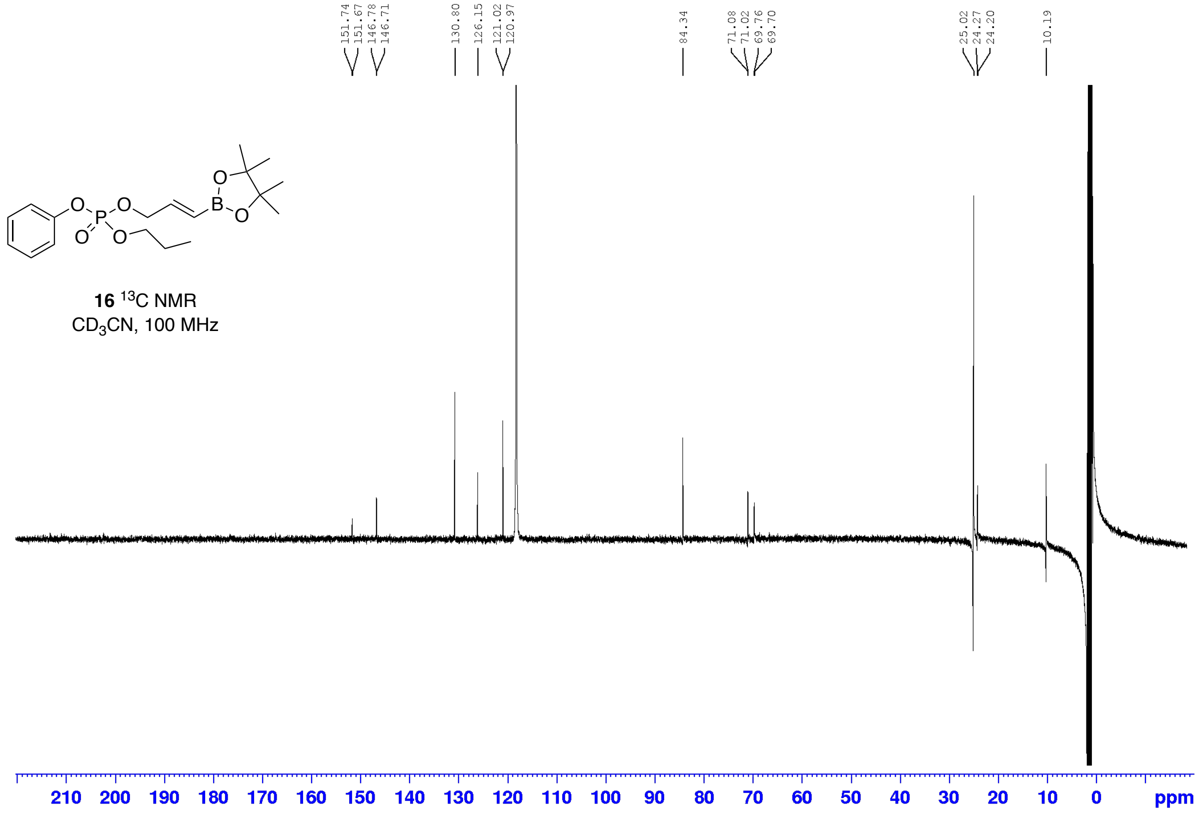
16 ^{13}C NMR
CD_3CN, 100 MHz
151.74 151.67 146.78 146.71 130.80 126.15 121.02 120.97 84.34 71.08 71.02 69.76 69.70 25.02 24.27 24.20 10.19
210 200 190 180 170 160 150 140 130 120 110 100 90 80 70 60 50 40 30 20 10 0 ppm

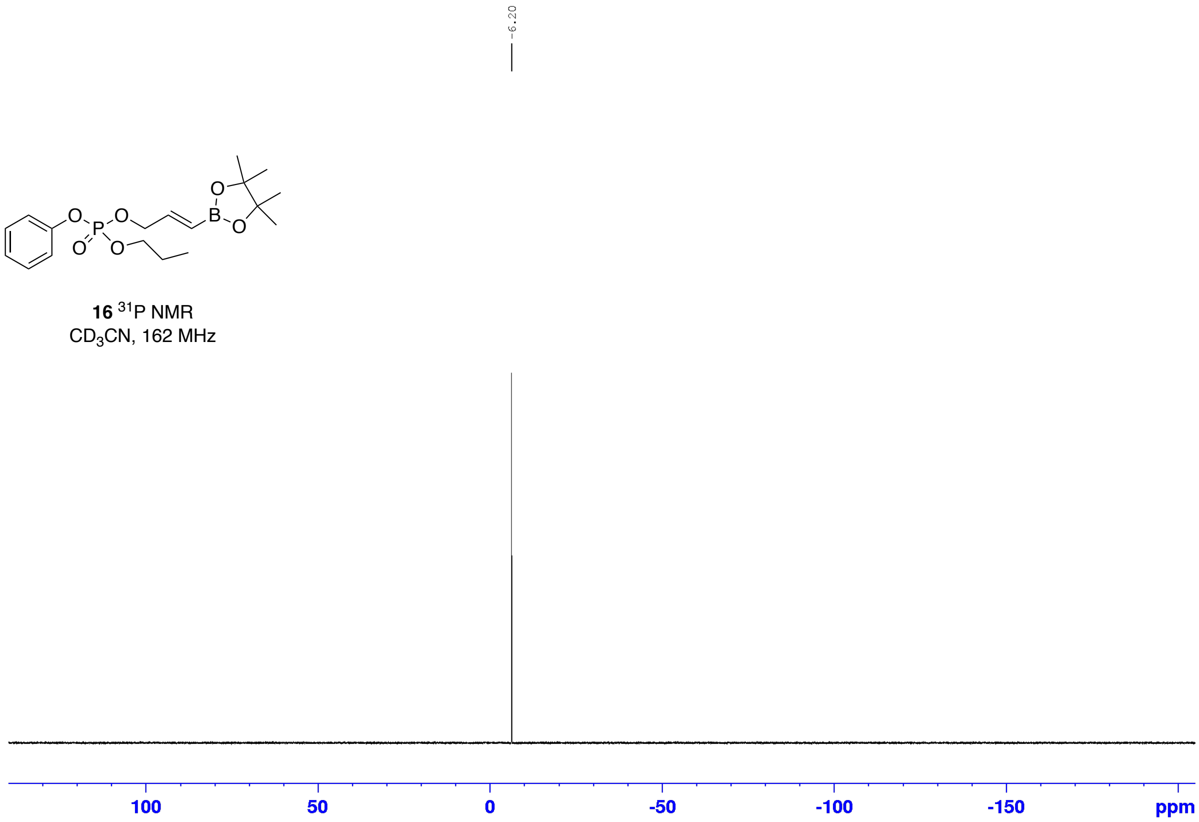
-6.20
16 31P NMR
CD3CN, 162 MHz
100
50
0
-50
-100
-150
ppm

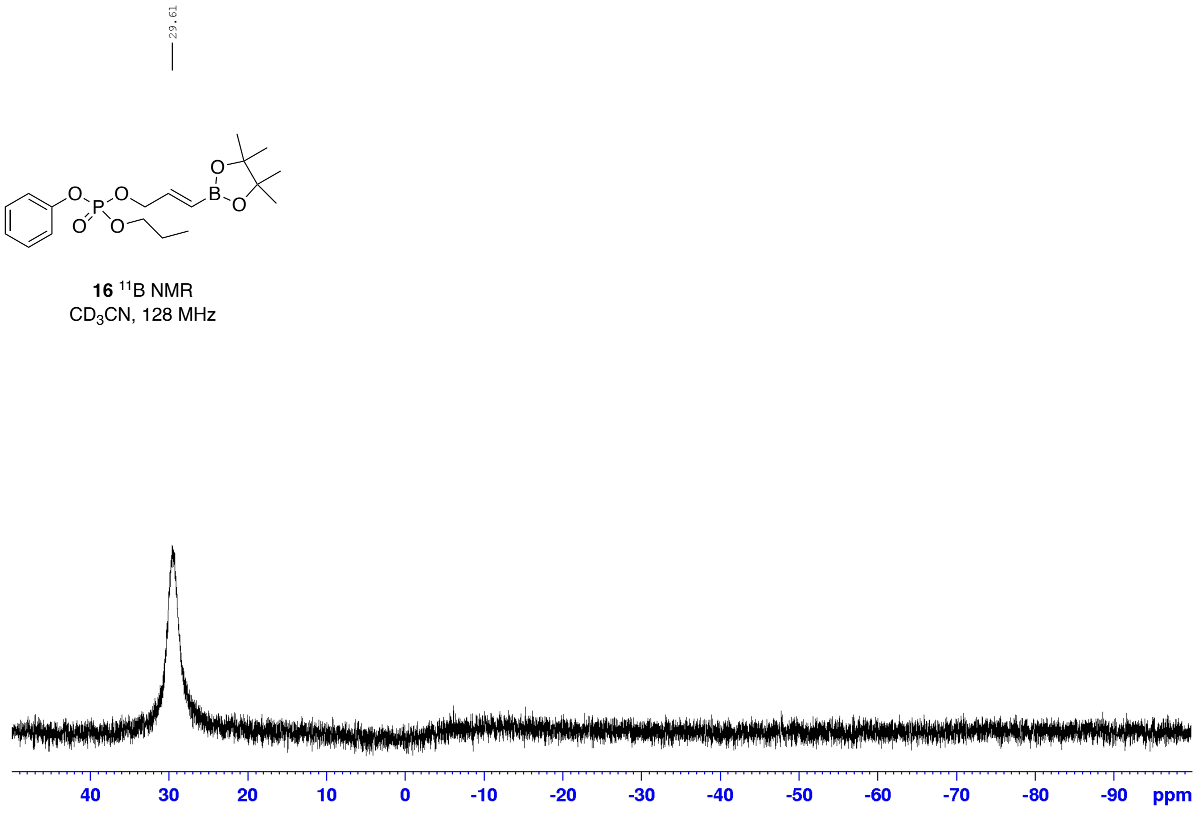
29.61
16 11B NMR
CD3CN, 128 MHz
40
30
20
10
0
-10
-20
-30
-40
-50
-60
-70
-80
-90
ppm

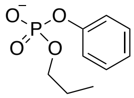

17 ^{1}H NMR
CD_3CN, D_2O, 500 MHz

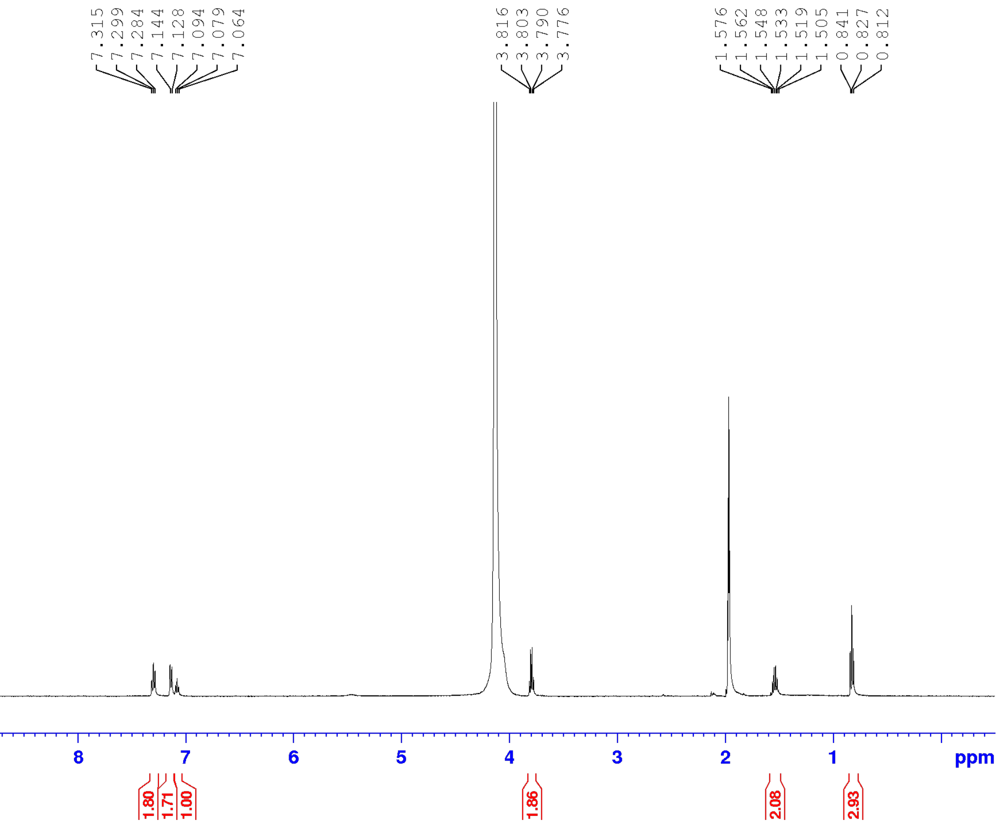

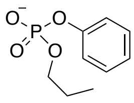

17 ^{13}C NMR
CD_3CN, D_2O, 101 MHz

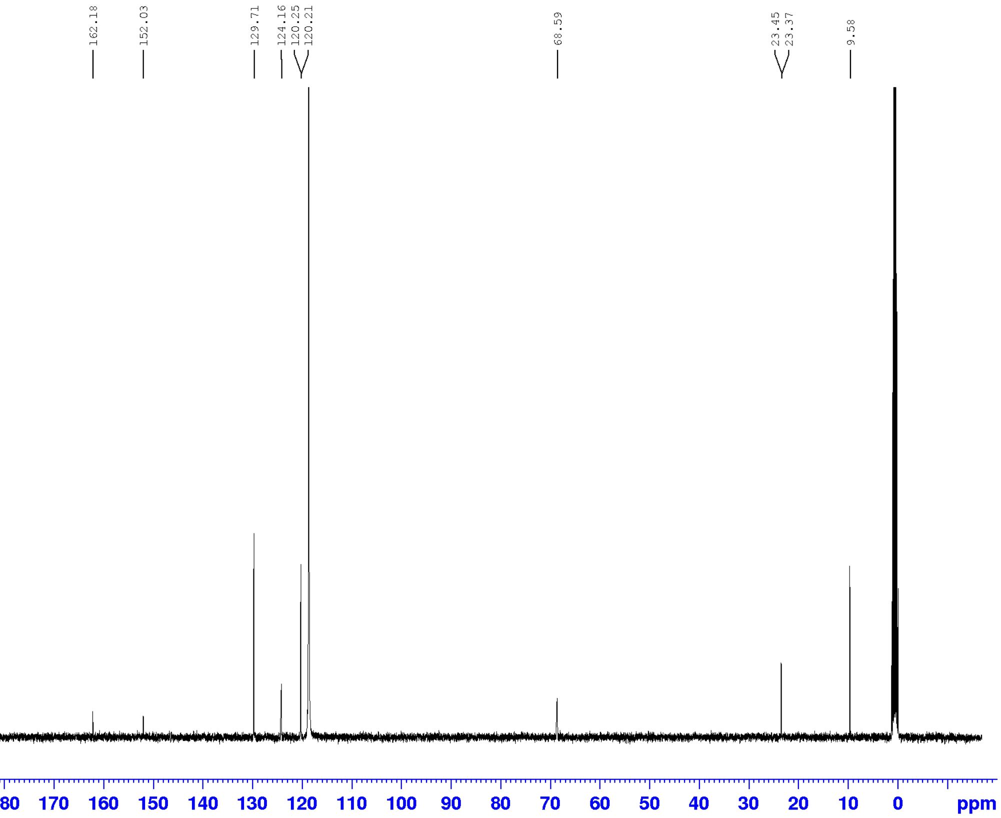

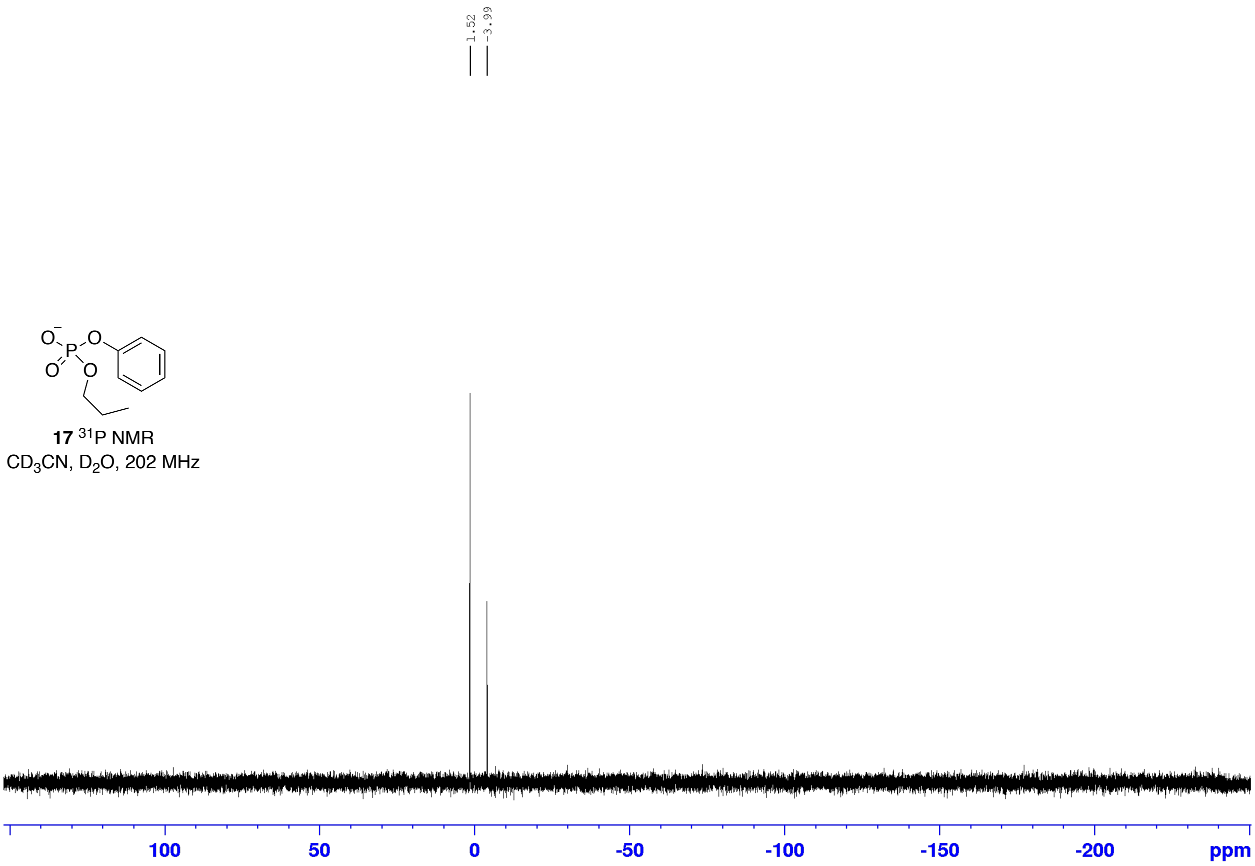

17 ^{31}P NMR
CD_3CN, D_2O, 202 MHz

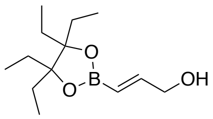

S5 ^{1}H NMR
$CDCl_3$, 500 MHz

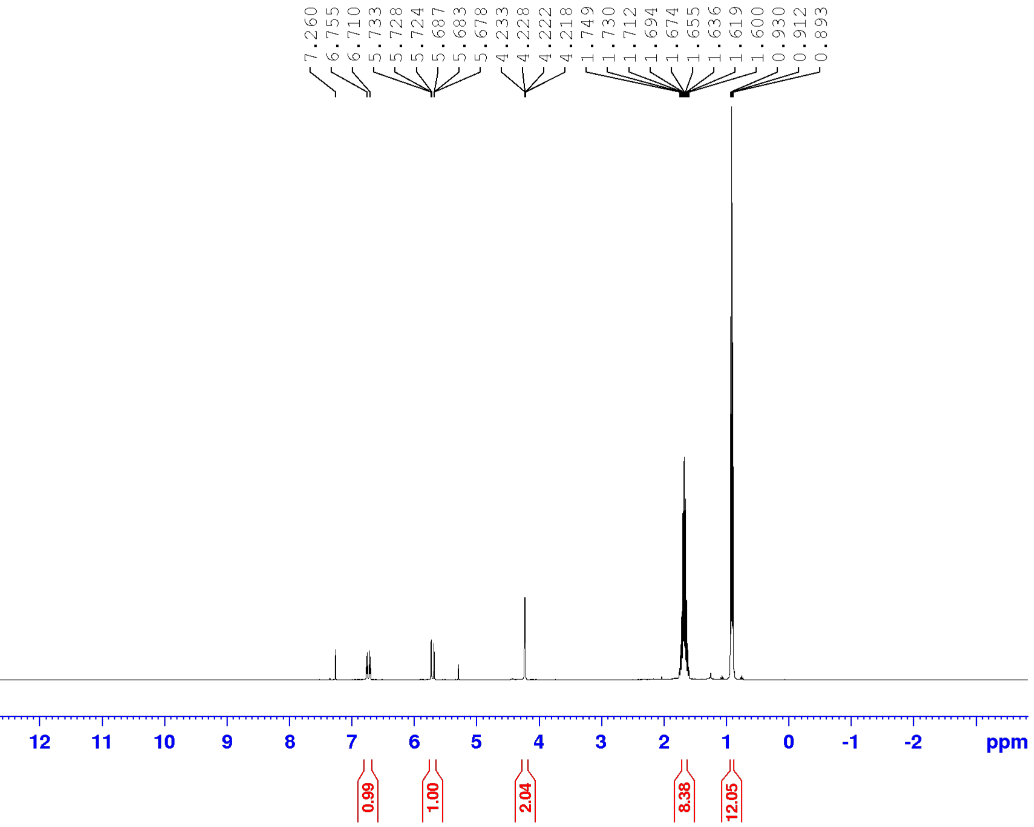

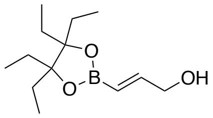

S5 ^{13}C NMR
$CDCl_3$, 125 MHz

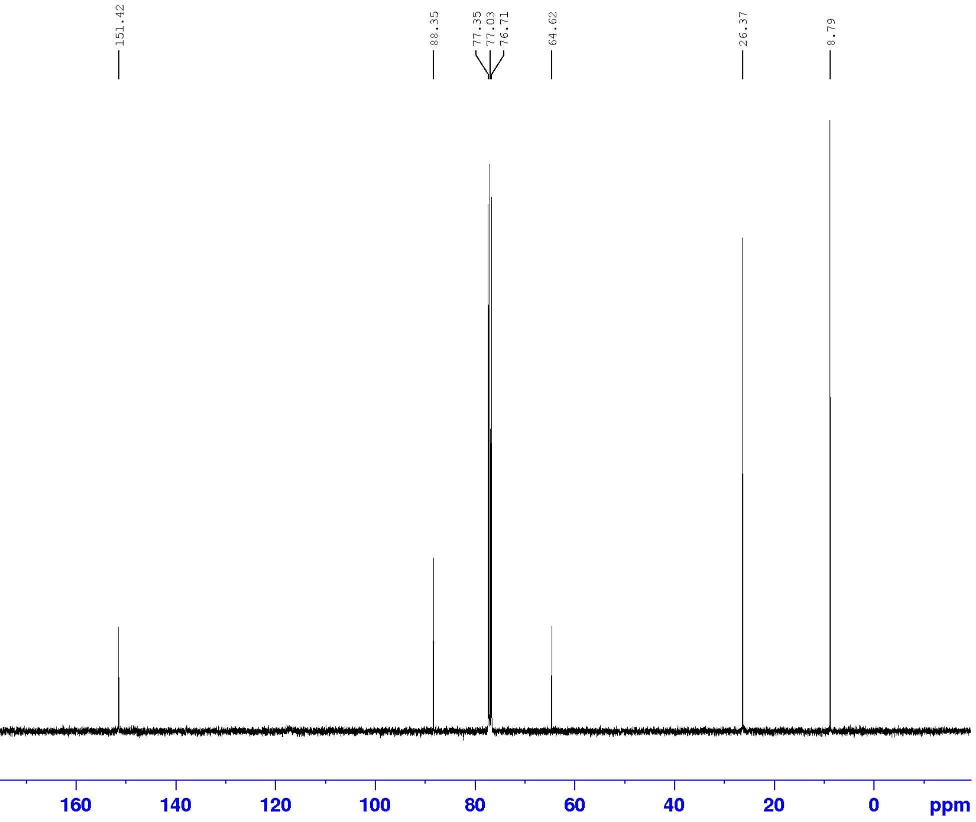

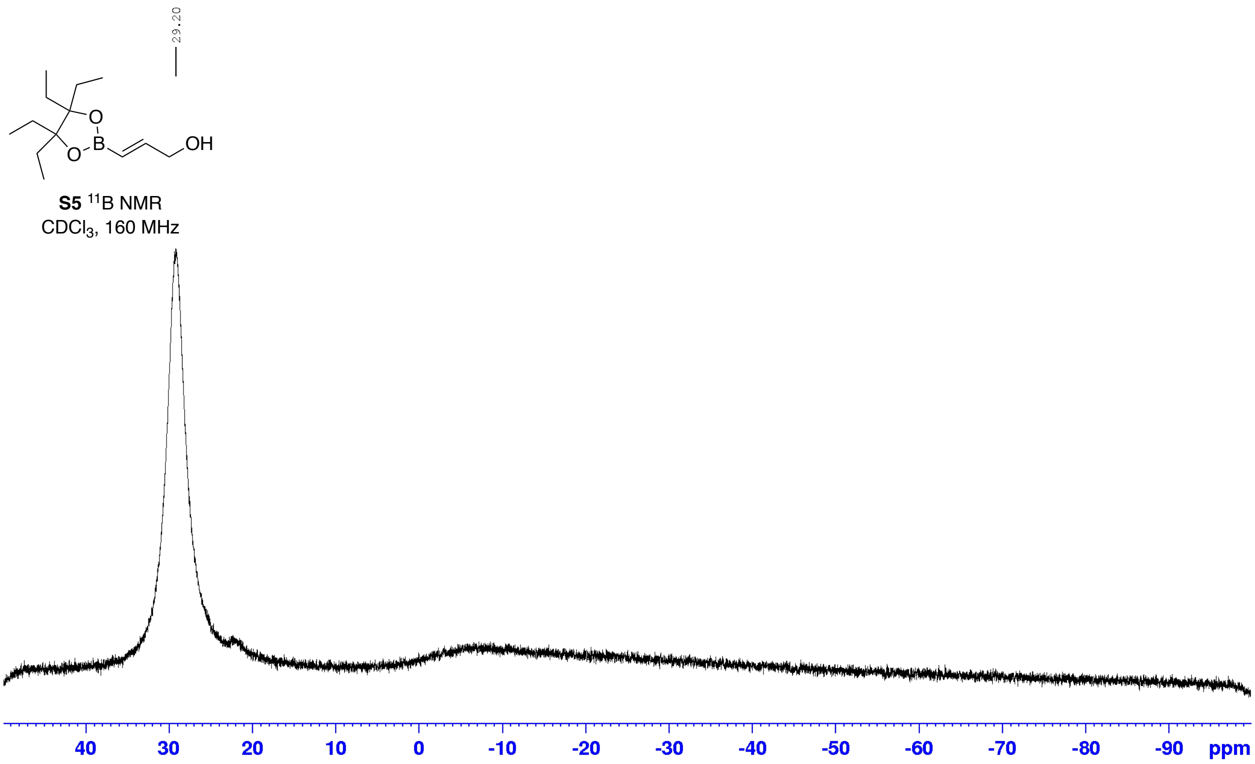

S5 ^{11}B NMR
$CDCl_3$, 160 MHz

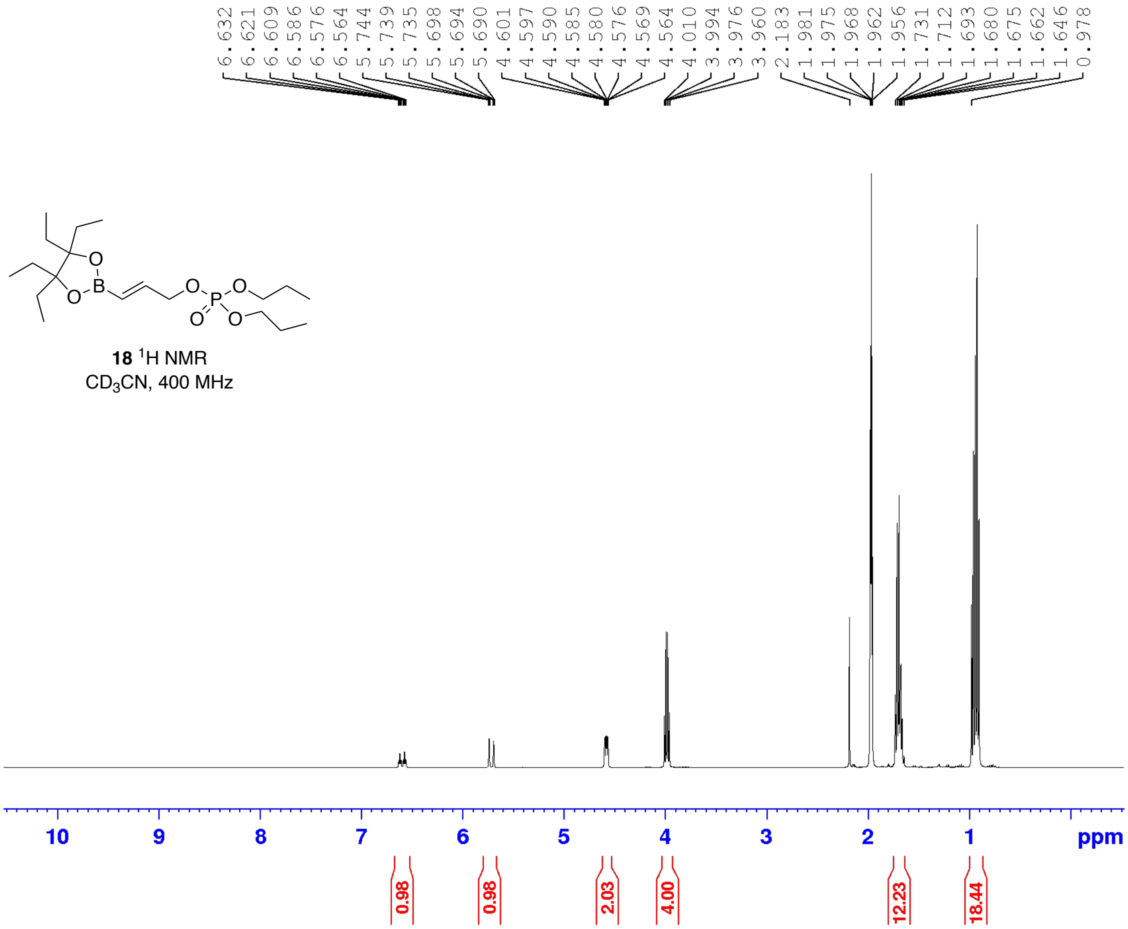

18 ^{1}H NMR
CD_3CN, 400 MHz

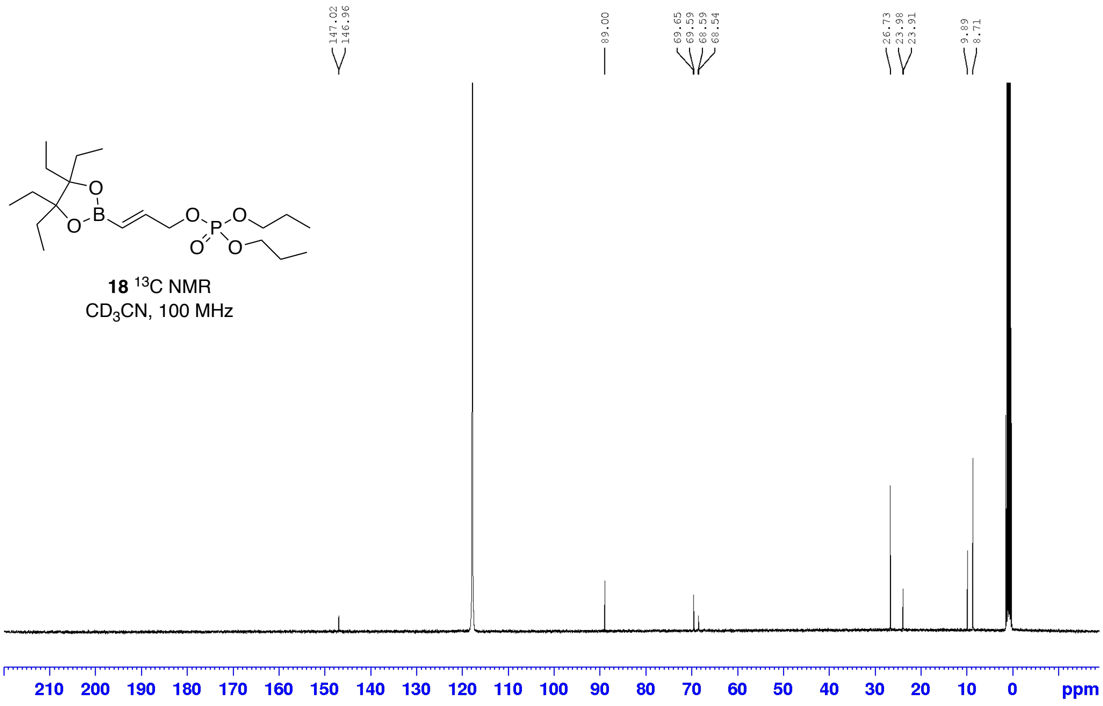

18 ^{13}C NMR
CD_3CN, 100 MHz

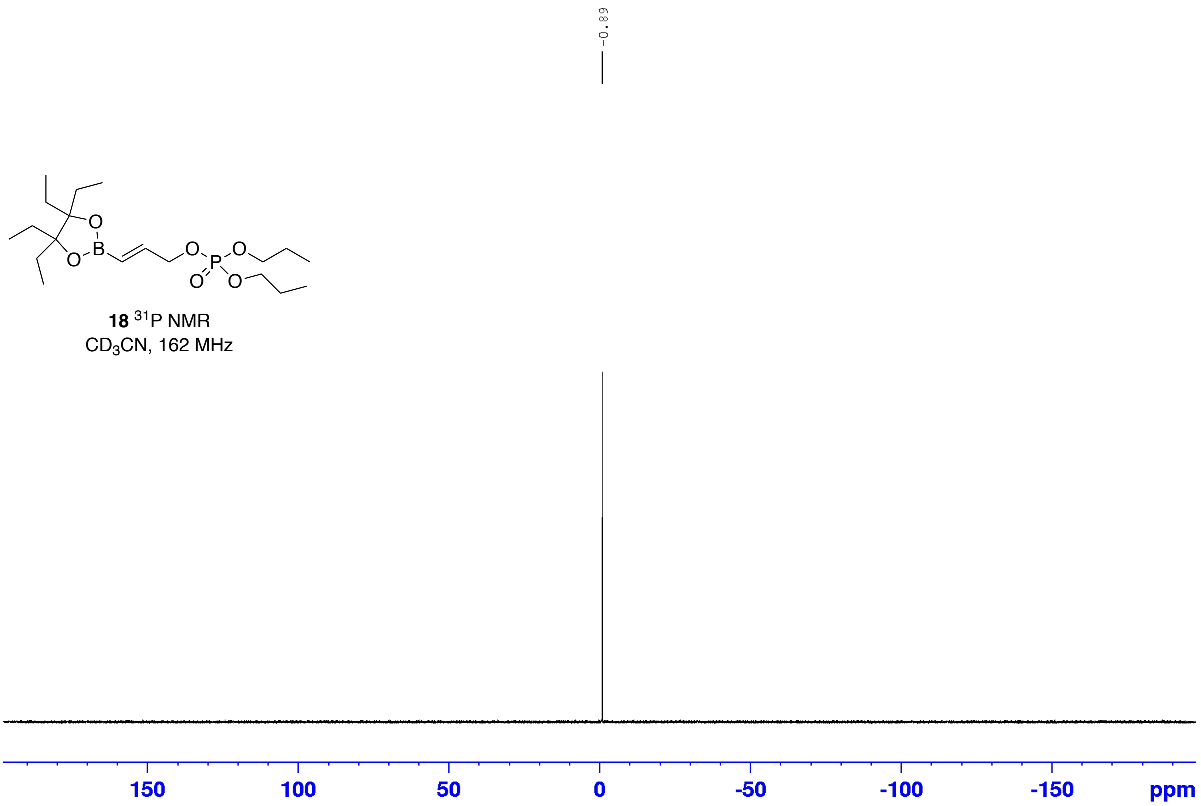
-0.89
18 ^{31}P NMR
CD_3CN, 162 MHz
150
100
50
0
-50
-100
-150
ppm

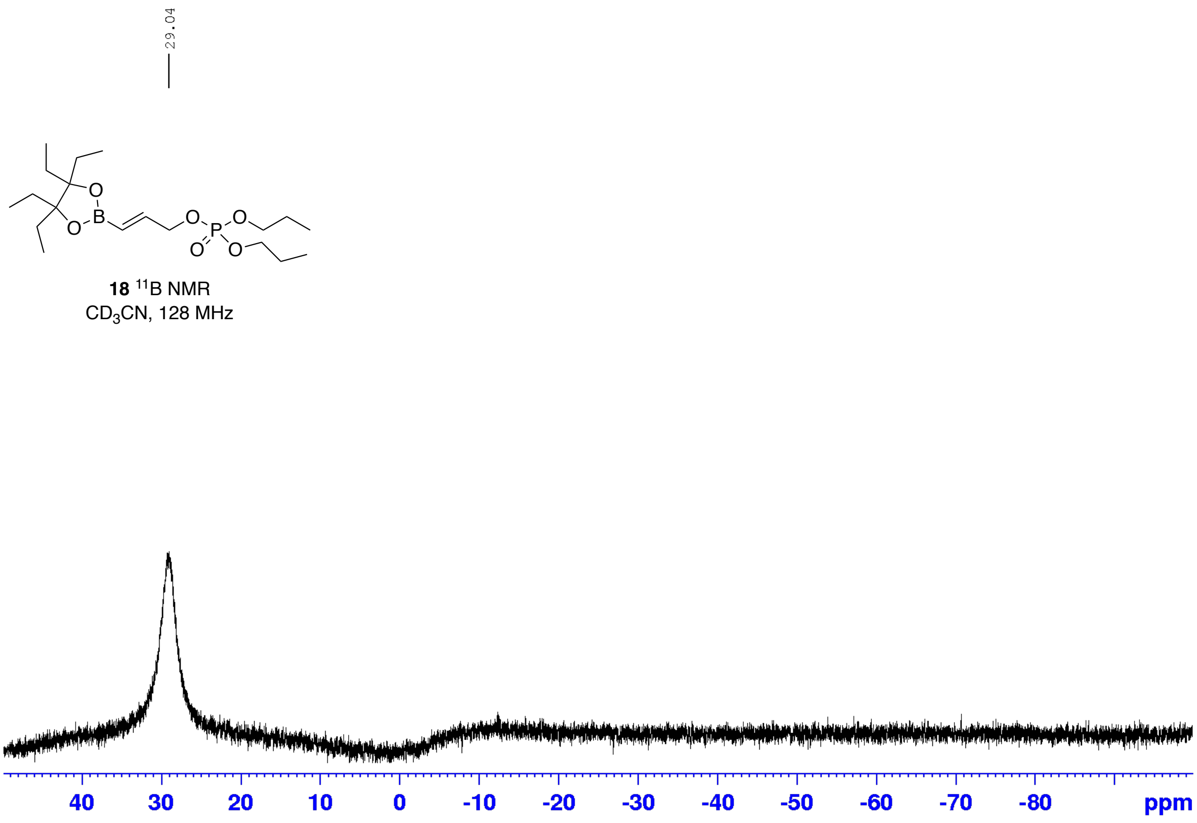
29.04
18 ^{11}B NMR
CD_3CN, 128 MHz
40
30
20
10
0
-10
-20
-30
-40
-50
-60
-70
-80
ppm

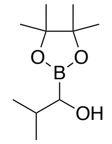

S6 ^{1}H NMR
$CDCl_3$, 400 MHz

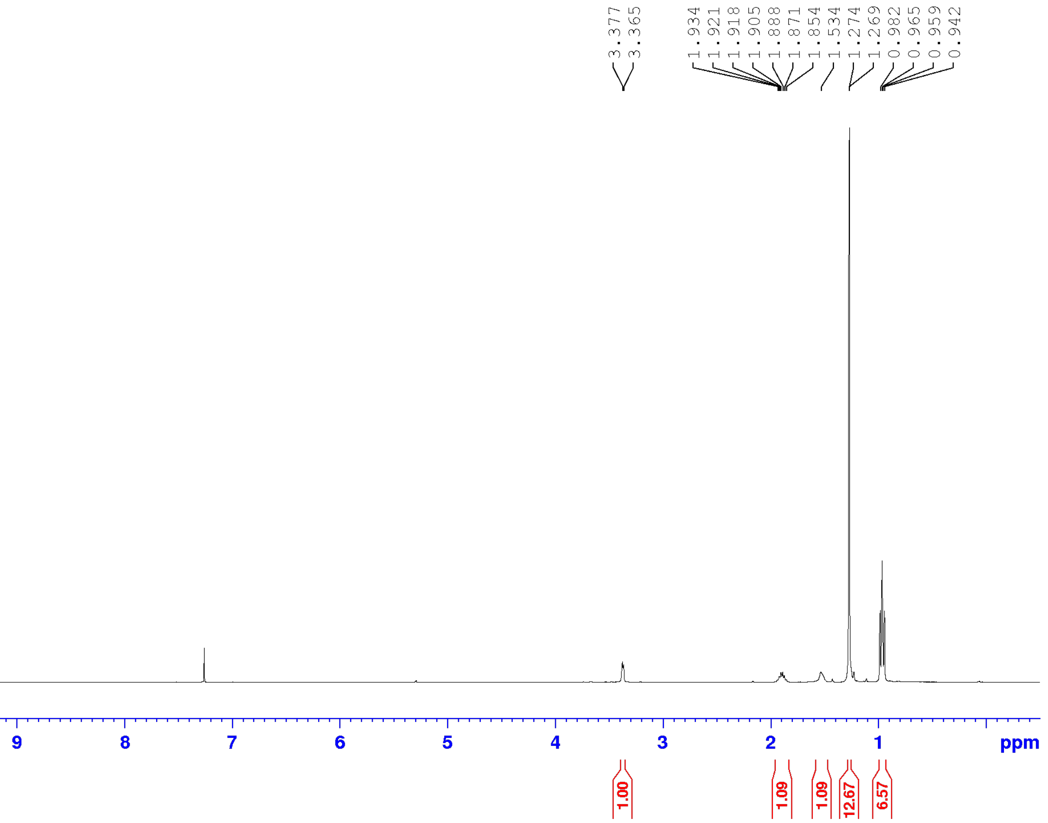

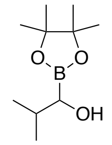

S6 ^{13}C NMR
$CDCl_3$, 100 MHz

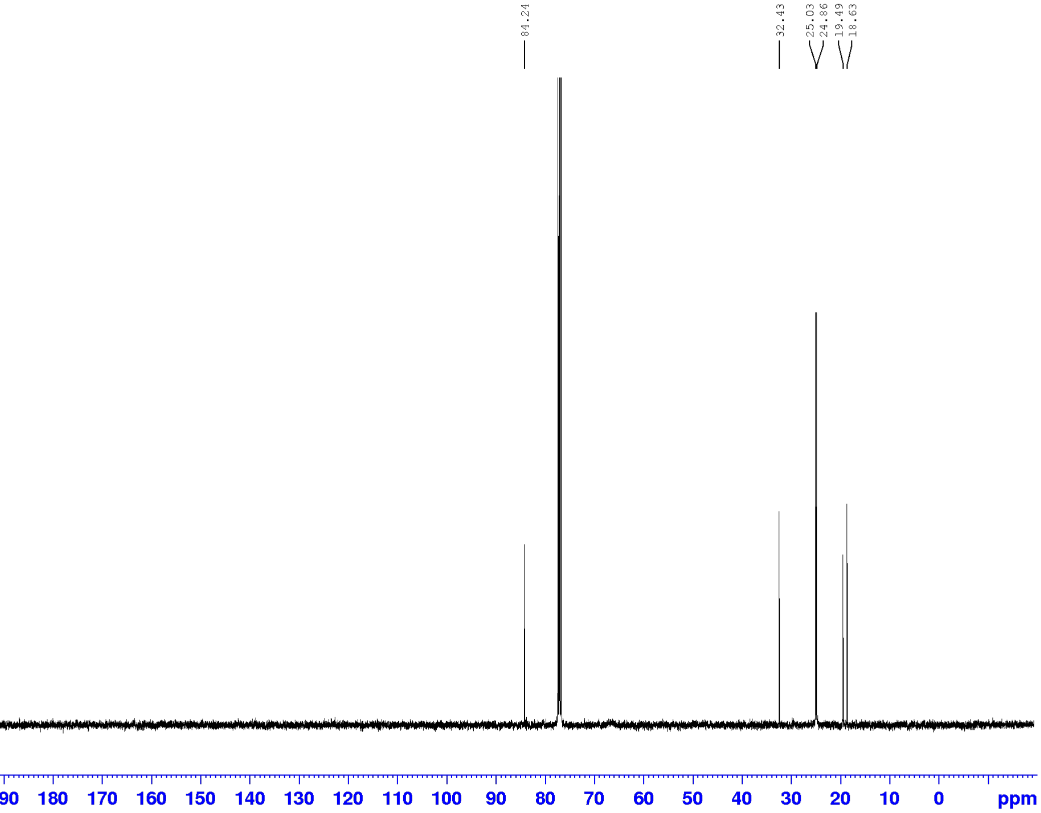

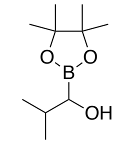

S6 ^{11}B NMR
$CDCl_3$, 128 MHz

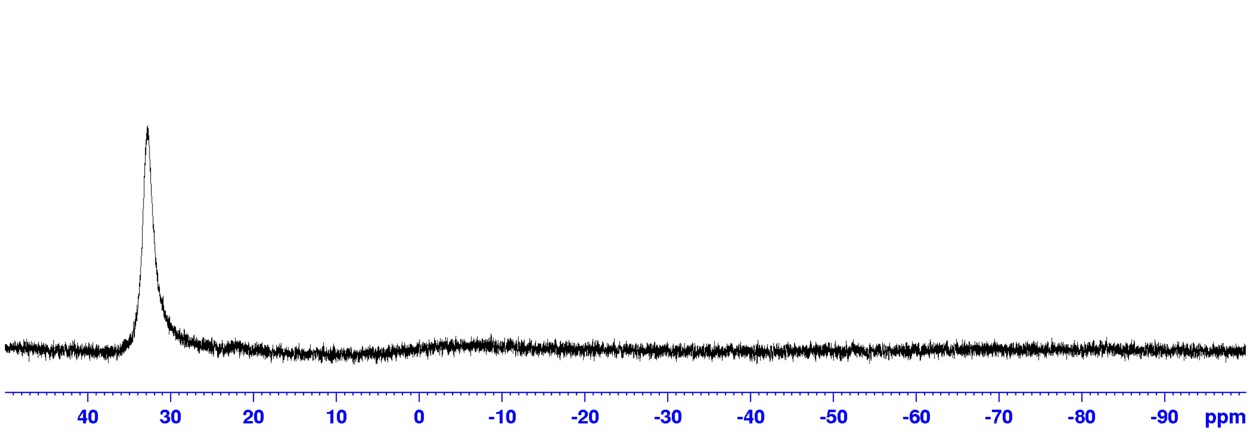

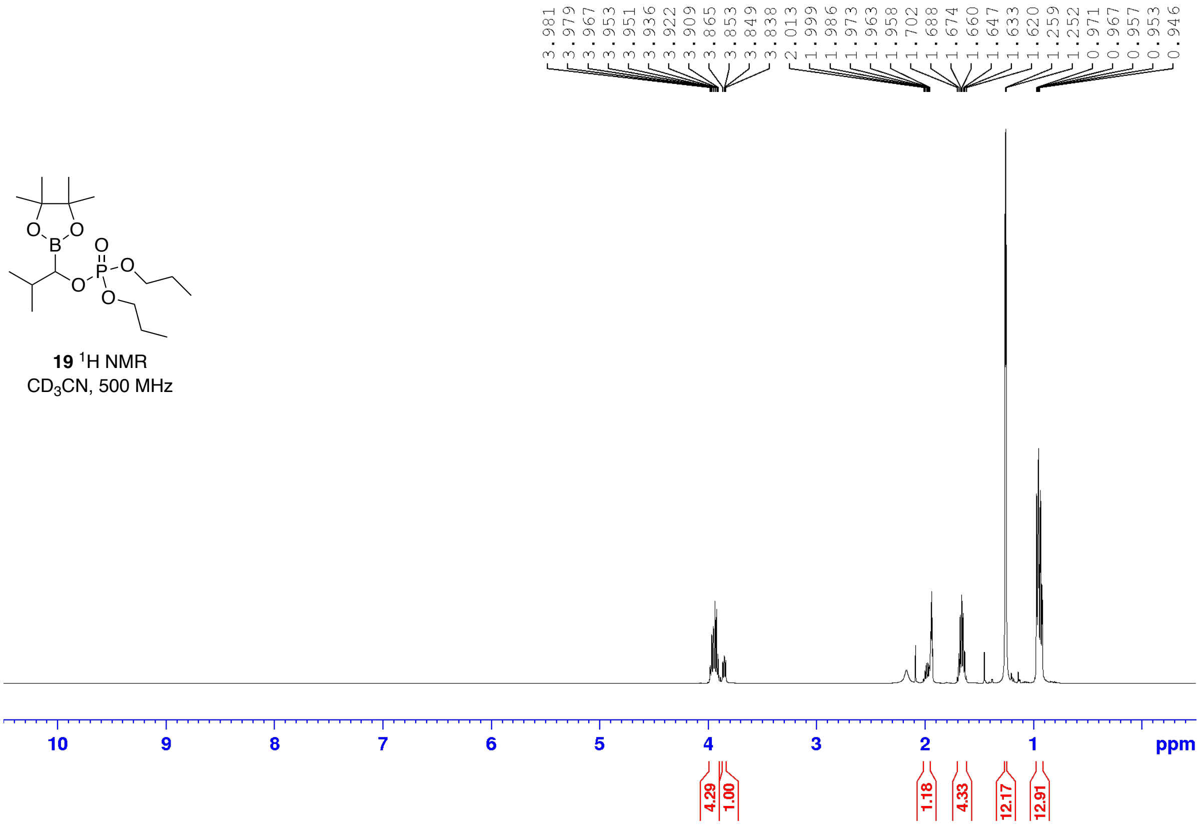
3.981
3.979
3.967
3.953
3.951
3.936
3.922
3.909
3.865
3.853
3.849
3.838
2.013
1.999
1.986
1.973
1.963
1.958
1.702
1.688
1.674
1.660
1.647
1.633
1.620
1.259
1.252
0.971
0.967
0.957
0.953
0.946
19 1H NMR
CD3CN, 500 MHz
10
9
8
7
6
5
4
3
2
1
ppm
4.29
1.00
1.18
4.33
12.17
12.91

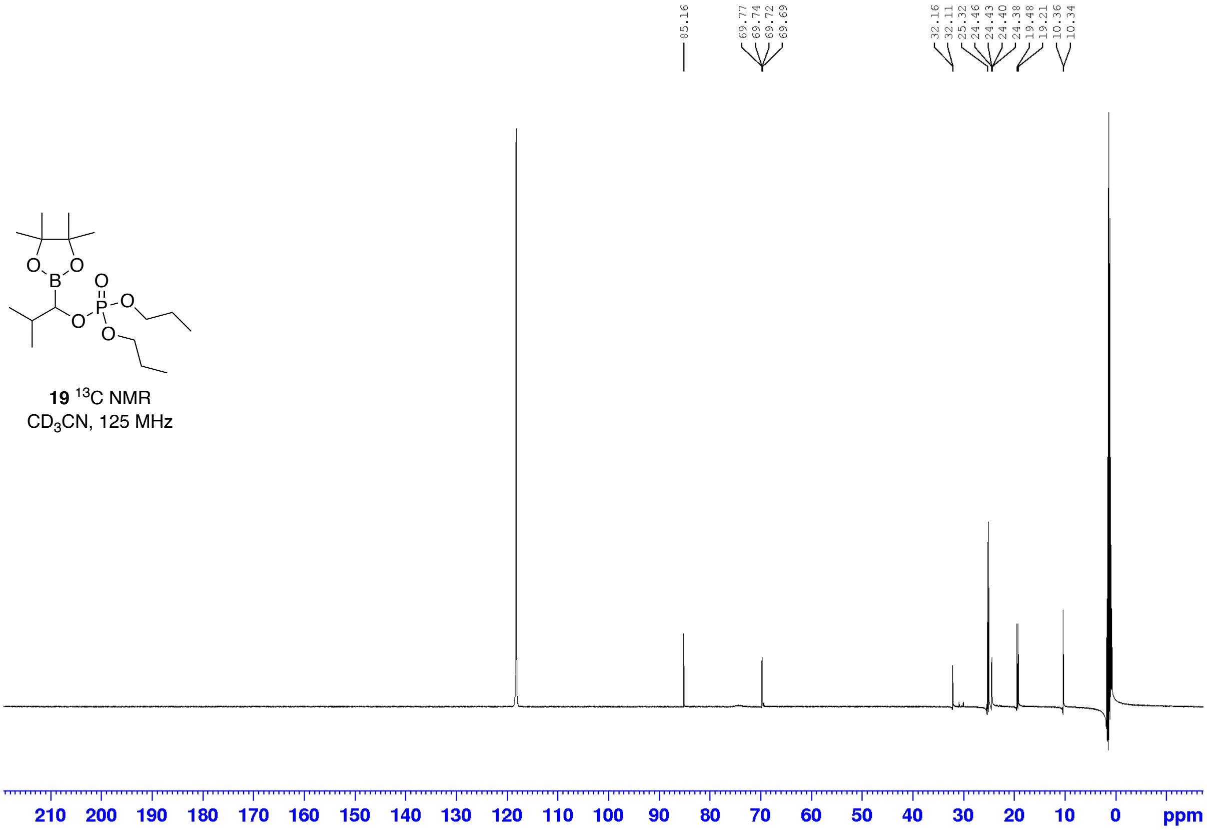
85.16
69.77
69.74
69.72
69.69
32.16
32.11
25.32
24.46
24.43
24.40
24.38
19.48
19.21
10.36
10.34
19 13C NMR
CD3CN, 125 MHz
210
200
190
180
170
160
150
140
130
120
110
100
90
80
70
60
50
40
30
20
10
0
ppm

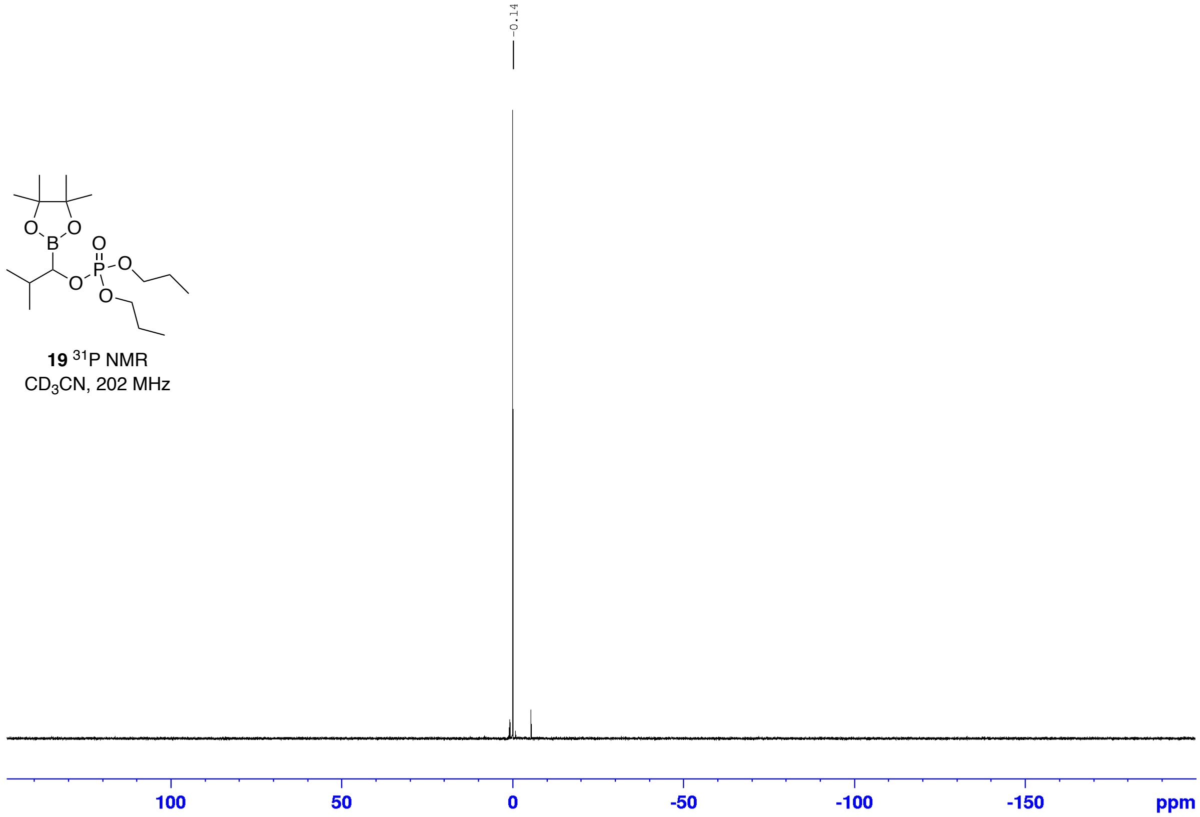
-0.14
19 31P NMR
CD3CN, 202 MHz
100
50
0
-50
-100
-150
ppm

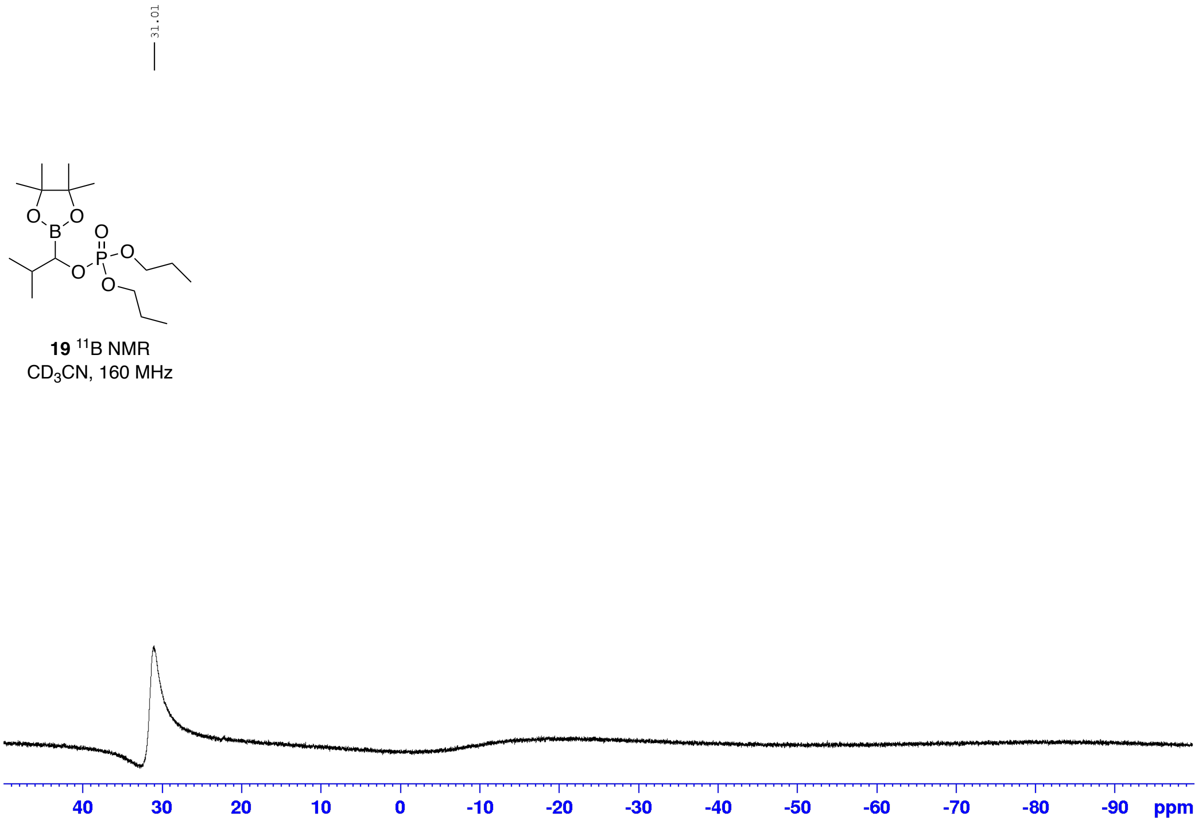
31.01
19 11B NMR
CD3CN, 160 MHz
40
30
20
10
0
-10
-20
-30
-40
-50
-60
-70
-80
-90
ppm

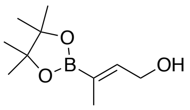

S7 ^{1}H NMR
$CDCl_3$, 400 MHz

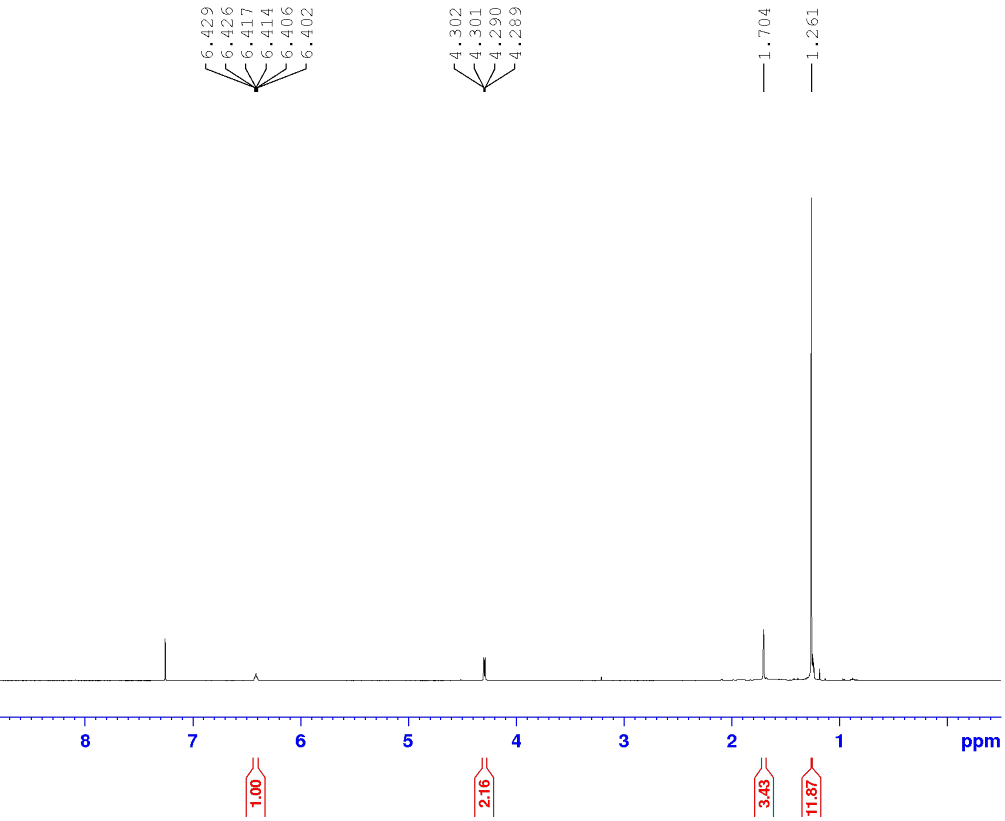

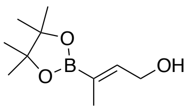

S7 ^{13}C NMR
$CDCl_3$, 125 MHz

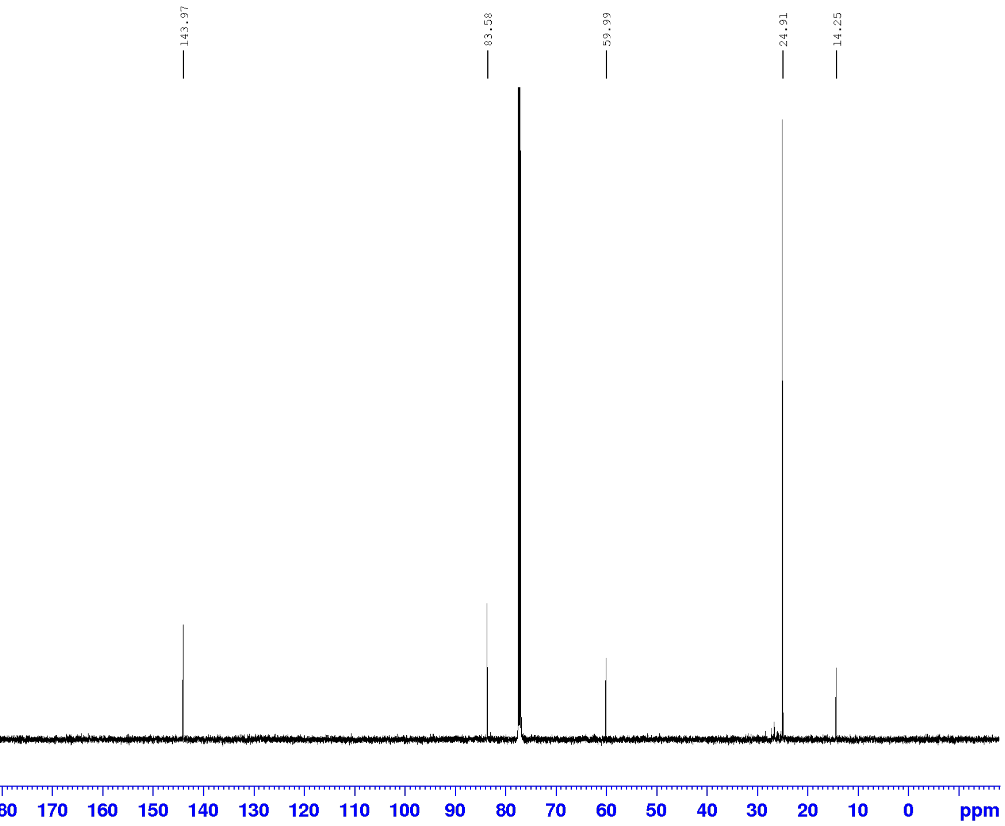

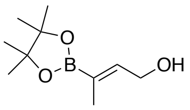

S7 ^{11}B NMR
$CDCl_3$, 160 MHz

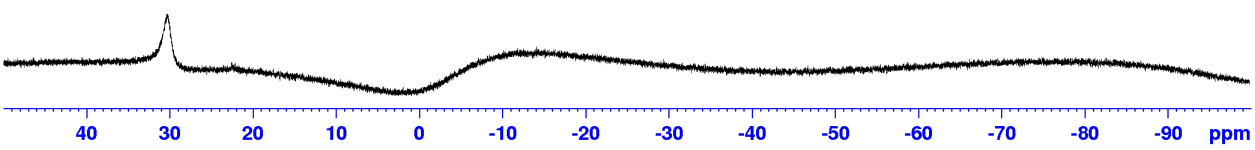

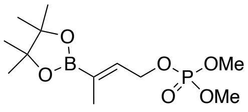

20 ^{1}H NMR
CD_3CN, 400 MHz

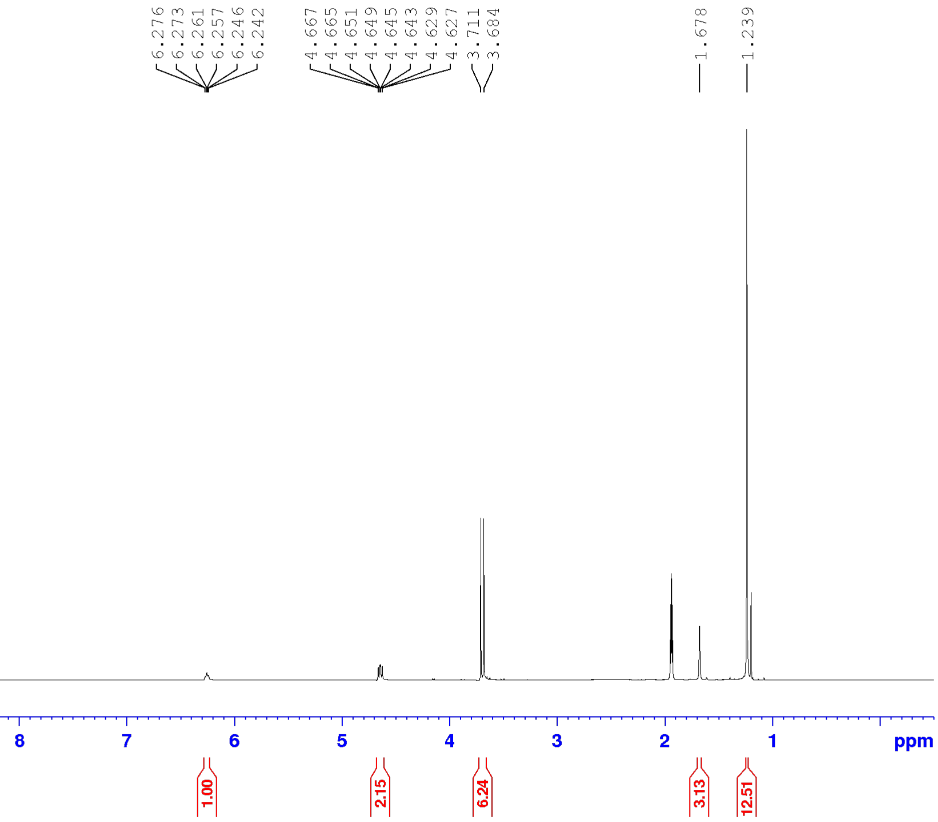

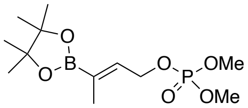

20 ^{13}C NMR
CD_3CN, 125 MHz

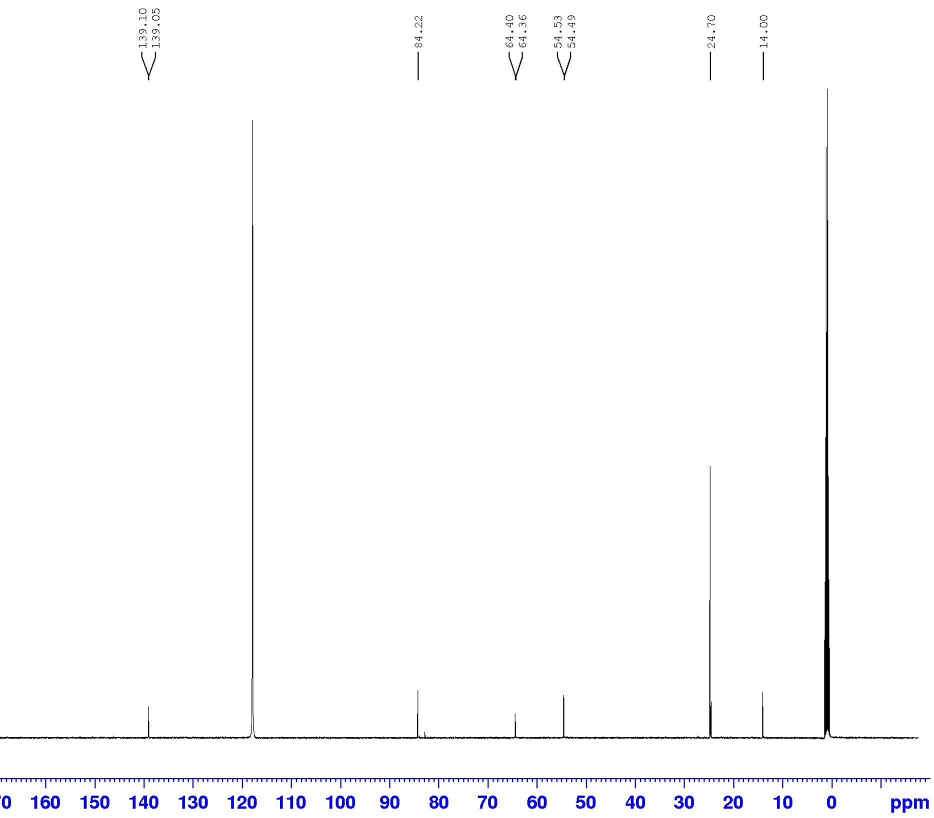

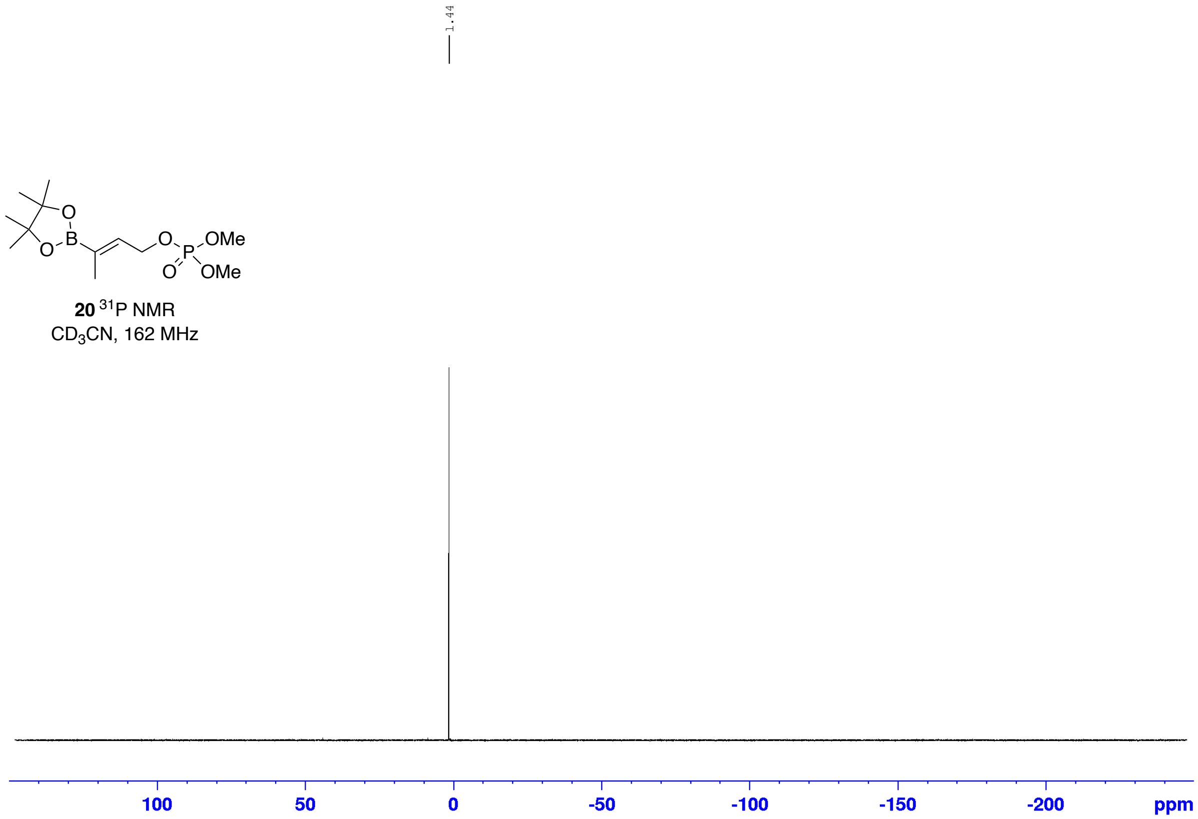

20 ^{31}P NMR
CD_3CN, 162 MHz

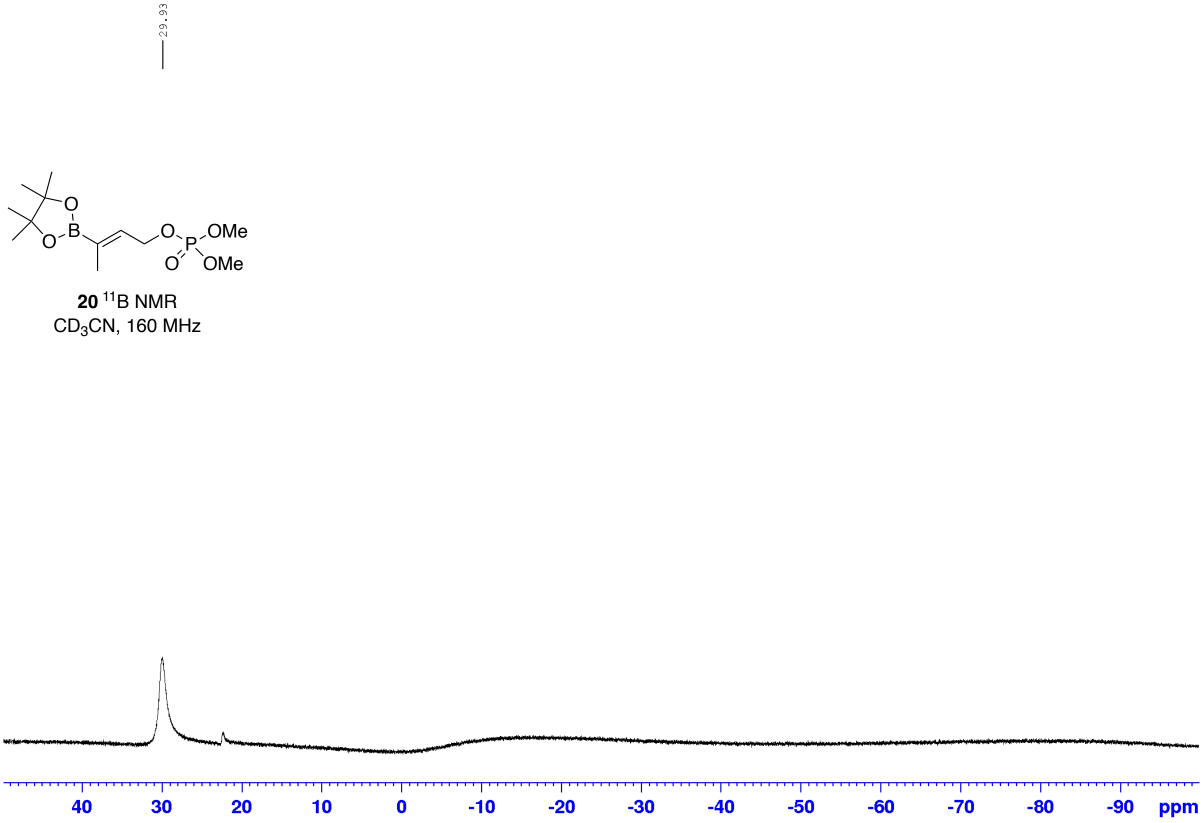

20 ^{11}B NMR
CD_3CN, 160 MHz

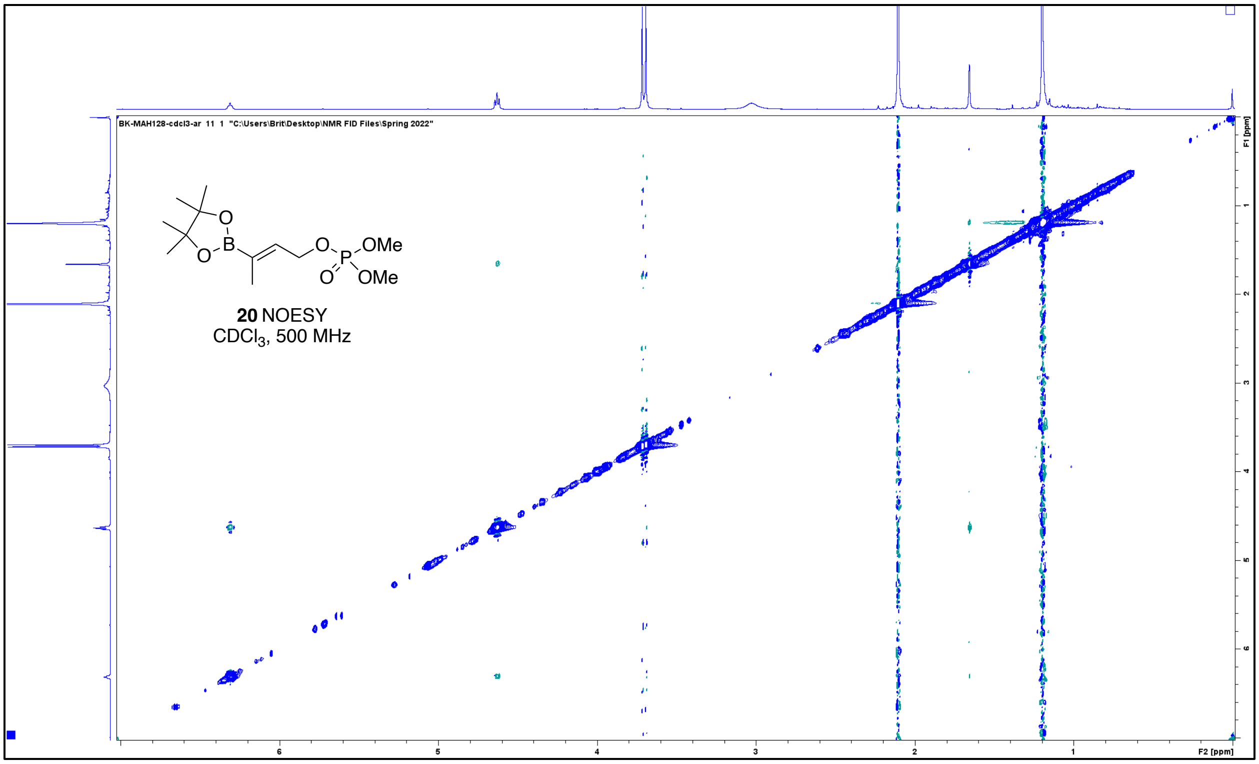
BK-MAH128-cdcl3-ar 11 1 "C:\Users\Brit\Desktop\NMR FID Files\Spring 2022"
OMe
OMe
20 NOESY
$CDCl_3$, 500 MHz
F1 [ppm]
F2 [ppm]

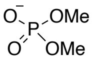

21 ^{1}H NMR
CD_3CN, D_2O, 300 MHz

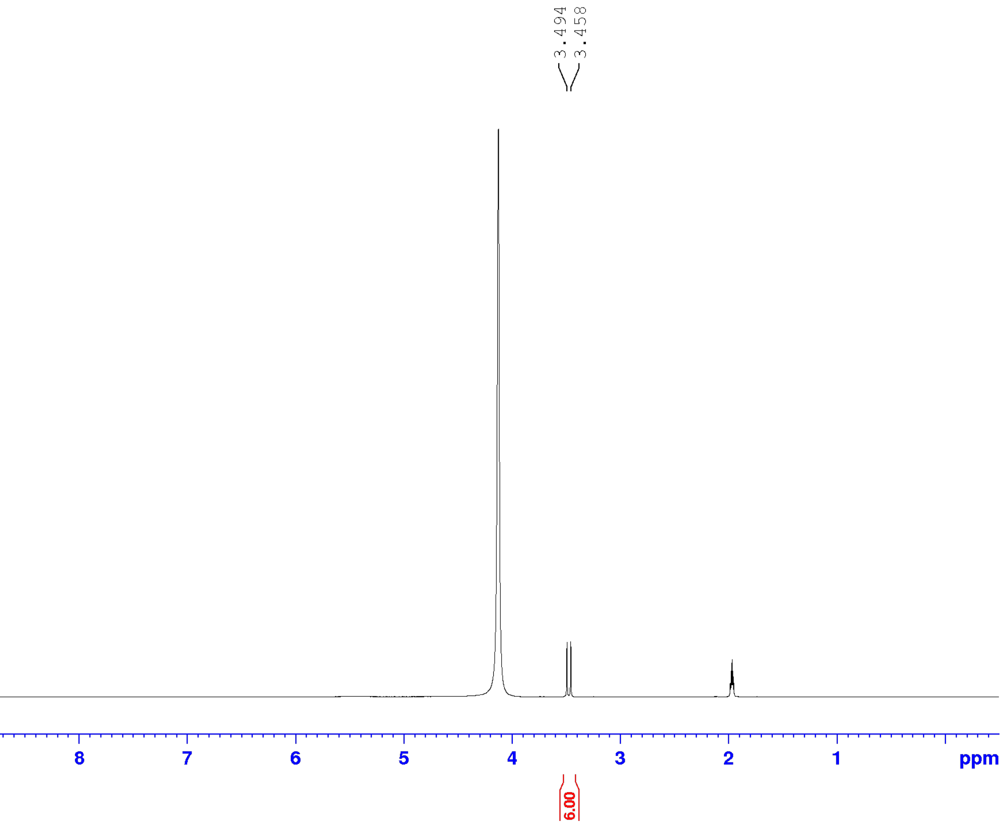

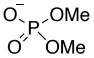

21 ^{13}C NMR
CD_3CN, D_2O, 76 MHz

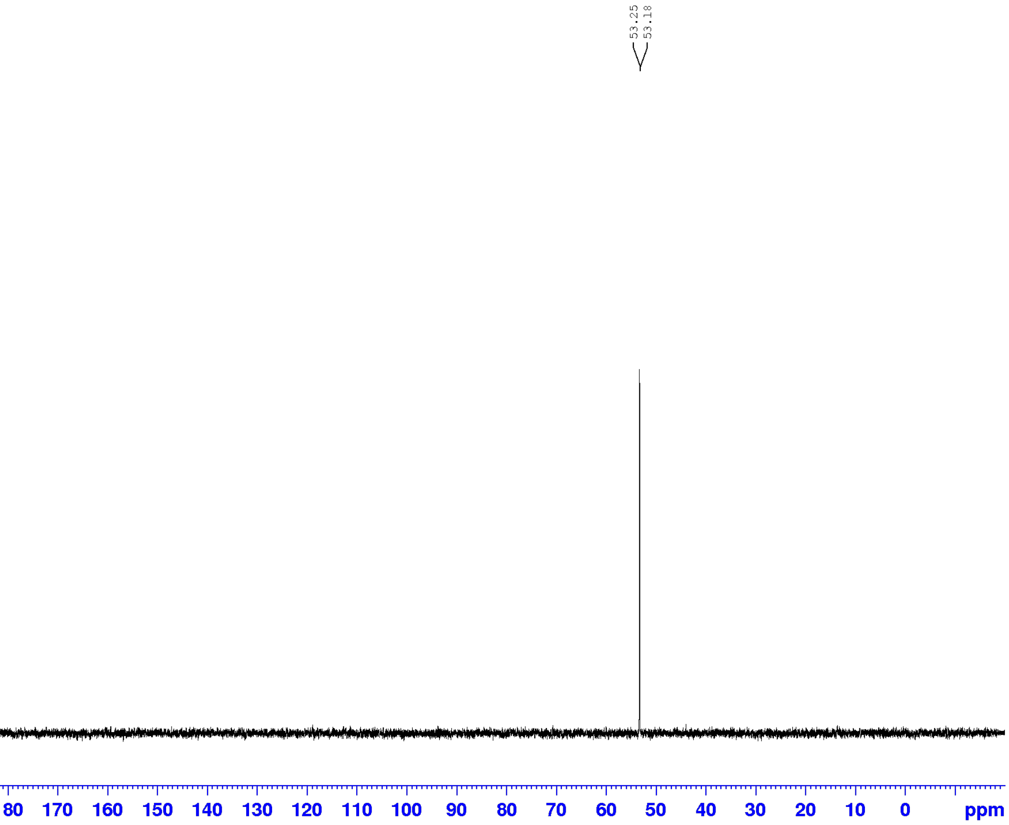

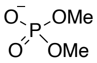

21 ^{31}P NMR
CD_3CN, D_2O, 122 MHz

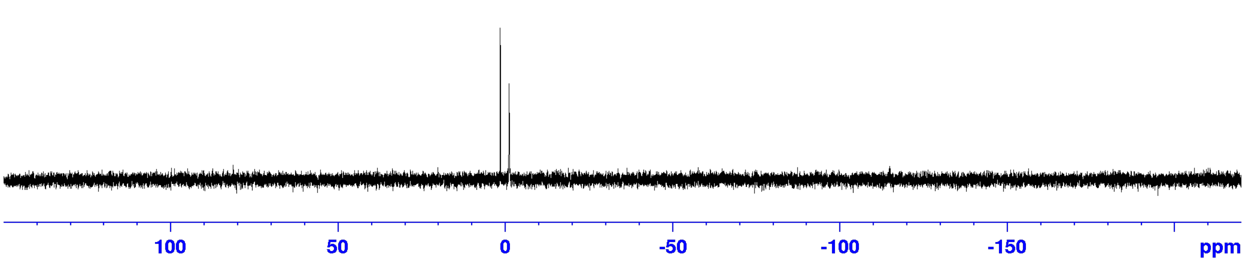

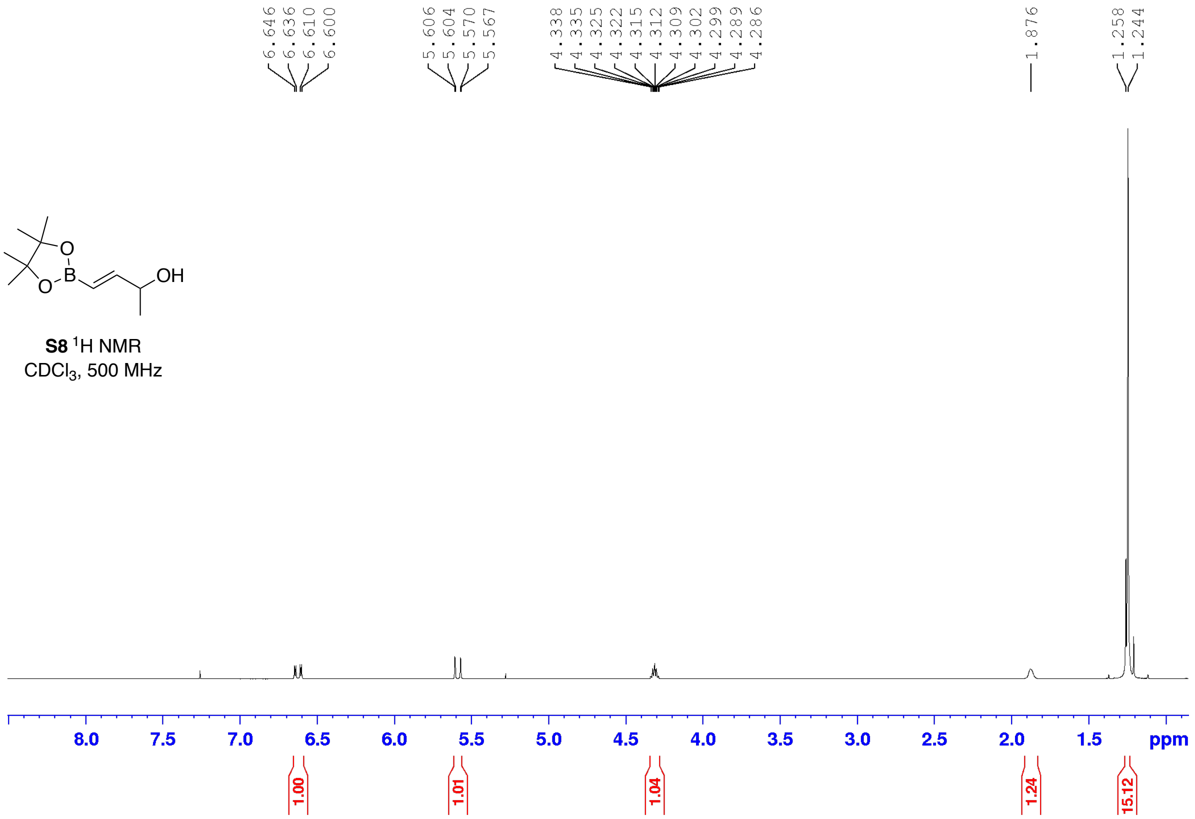
6.646
6.636
6.610
6.600
5.606
5.604
5.570
5.567
4.338
4.335
4.325
4.322
4.315
4.312
4.309
4.302
4.299
4.289
4.286
1.876
1.258
1.244
O
B
O
OH
S8 1H NMR
CDCl3, 500 MHz
8.0 7.5 7.0 6.5 6.0 5.5 5.0 4.5 4.0 3.5 3.0 2.5 2.0 1.5 ppm
1.00
1.01
1.04
1.24
15.12

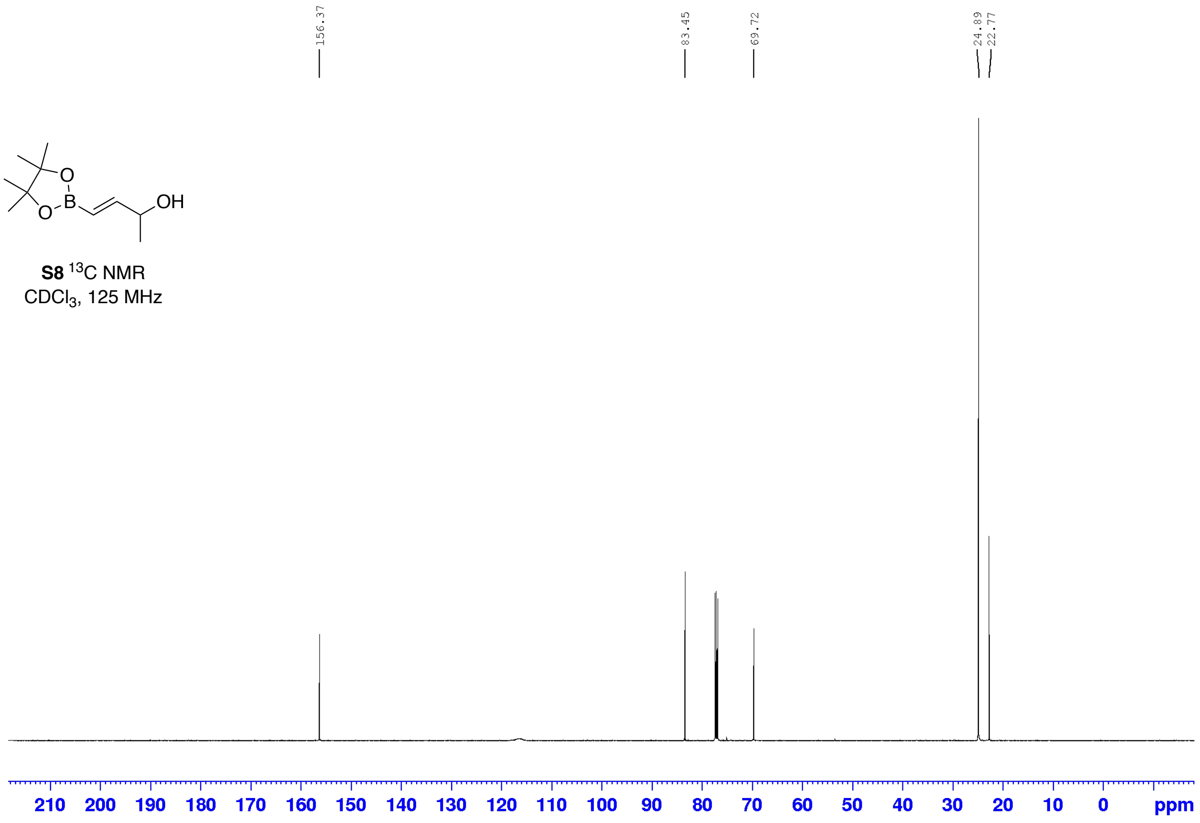
156.37
83.45
69.72
24.89
22.77
O
B
O
OH
S8 13C NMR
CDCl3, 125 MHz
210 200 190 180 170 160 150 140 130 120 110 100 90 80 70 60 50 40 30 20 10 0 ppm

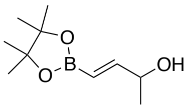

S8 ^{11}B NMR
$CDCl_3$, 160 MHz

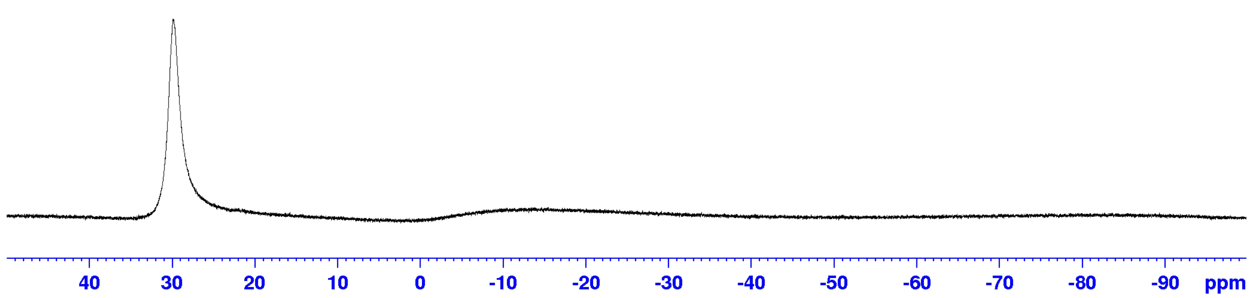

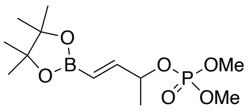

22 ^{1}H NMR
$CDCl_3$, 500 MHz

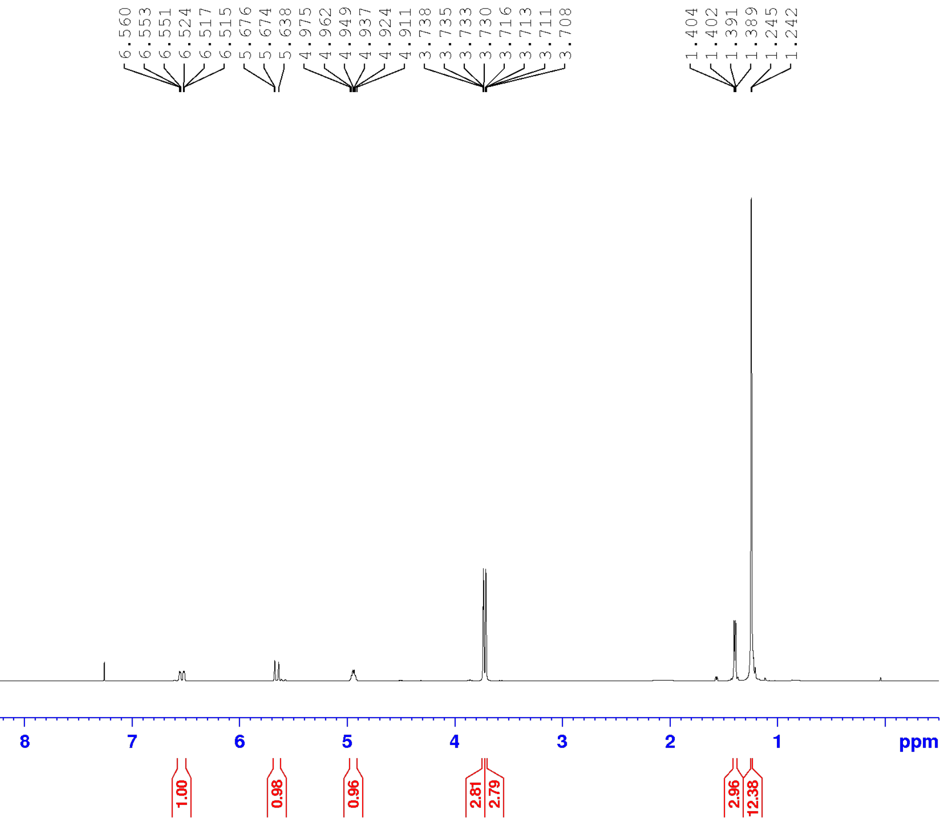

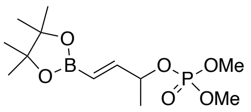

22 ^{13}C NMR
$CDCl_3$, 125 MHz

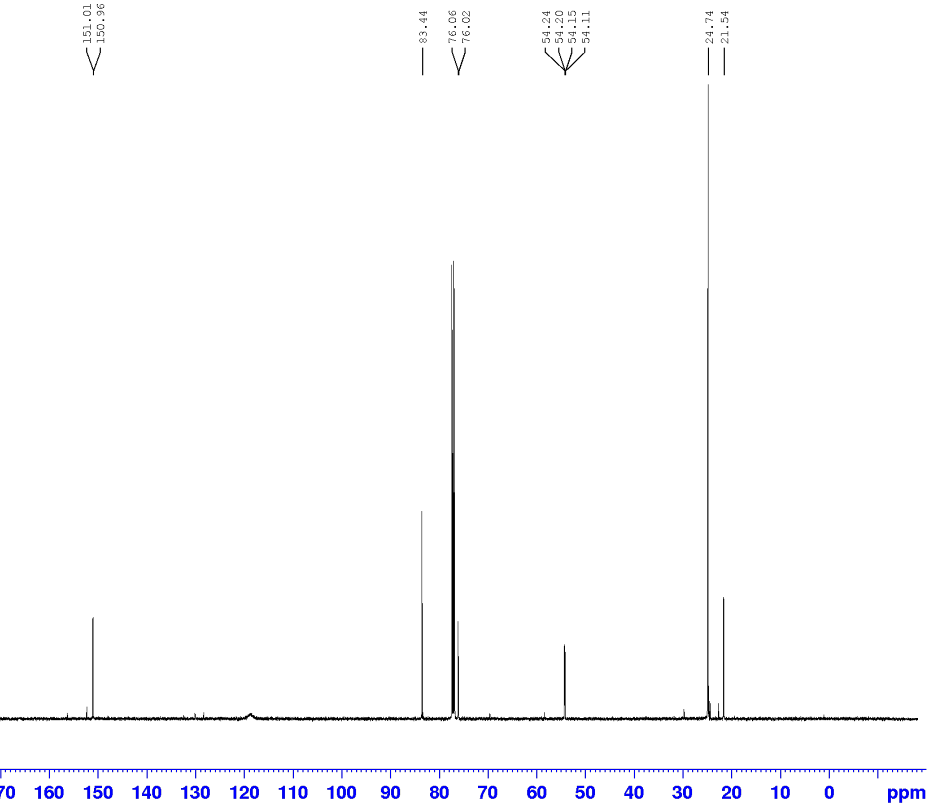

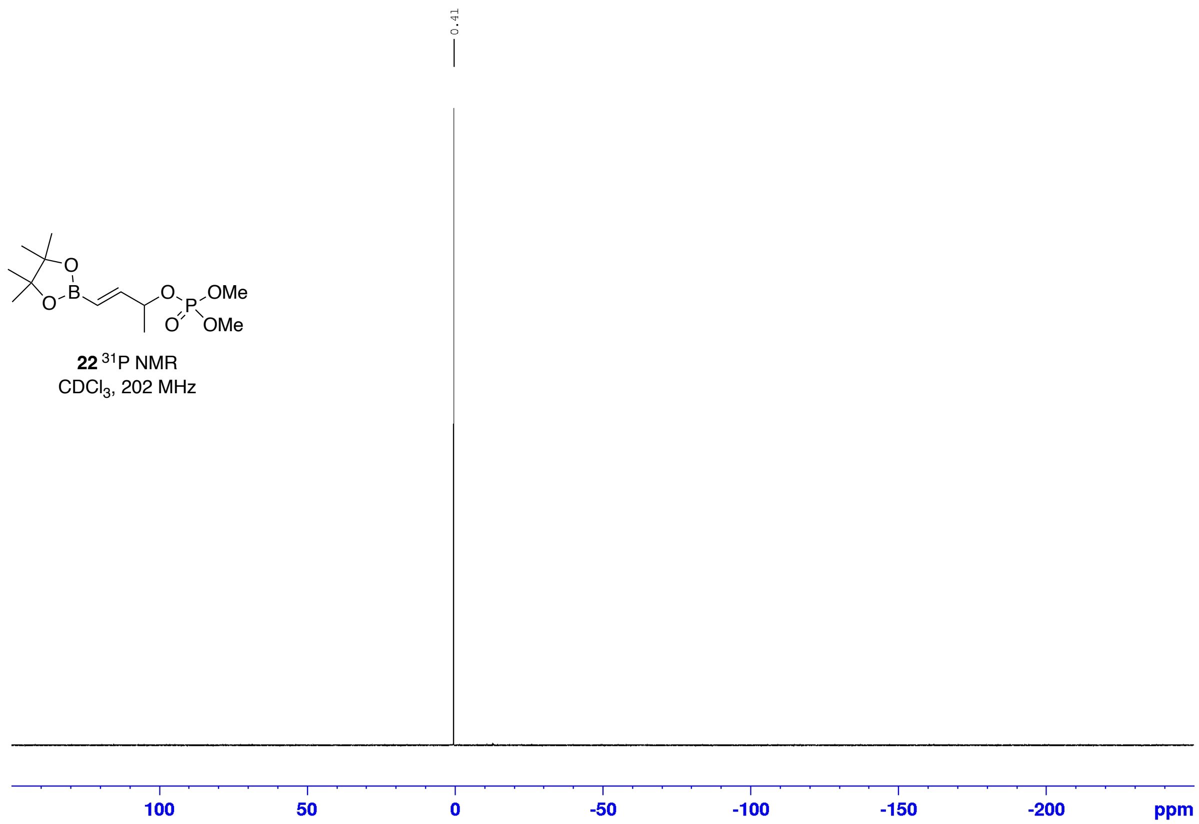
0.41
OMe
OMe
22 31P NMR
CDCl3, 202 MHz
100 50 0 -50 -100 -150 -200 ppm

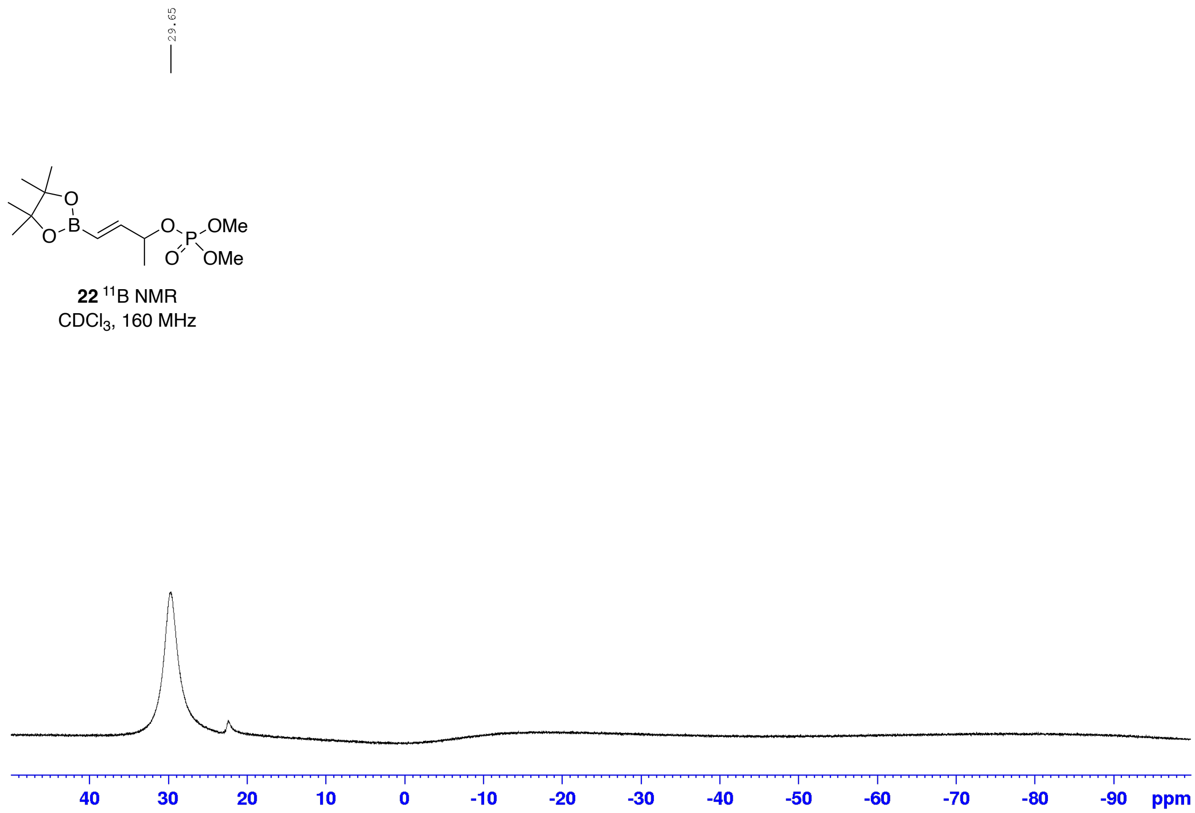
29.65
OMe
OMe
22 11B NMR
CDCl3, 160 MHz
40 30 20 10 0 -10 -20 -30 -40 -50 -60 -70 -80 -90 ppm

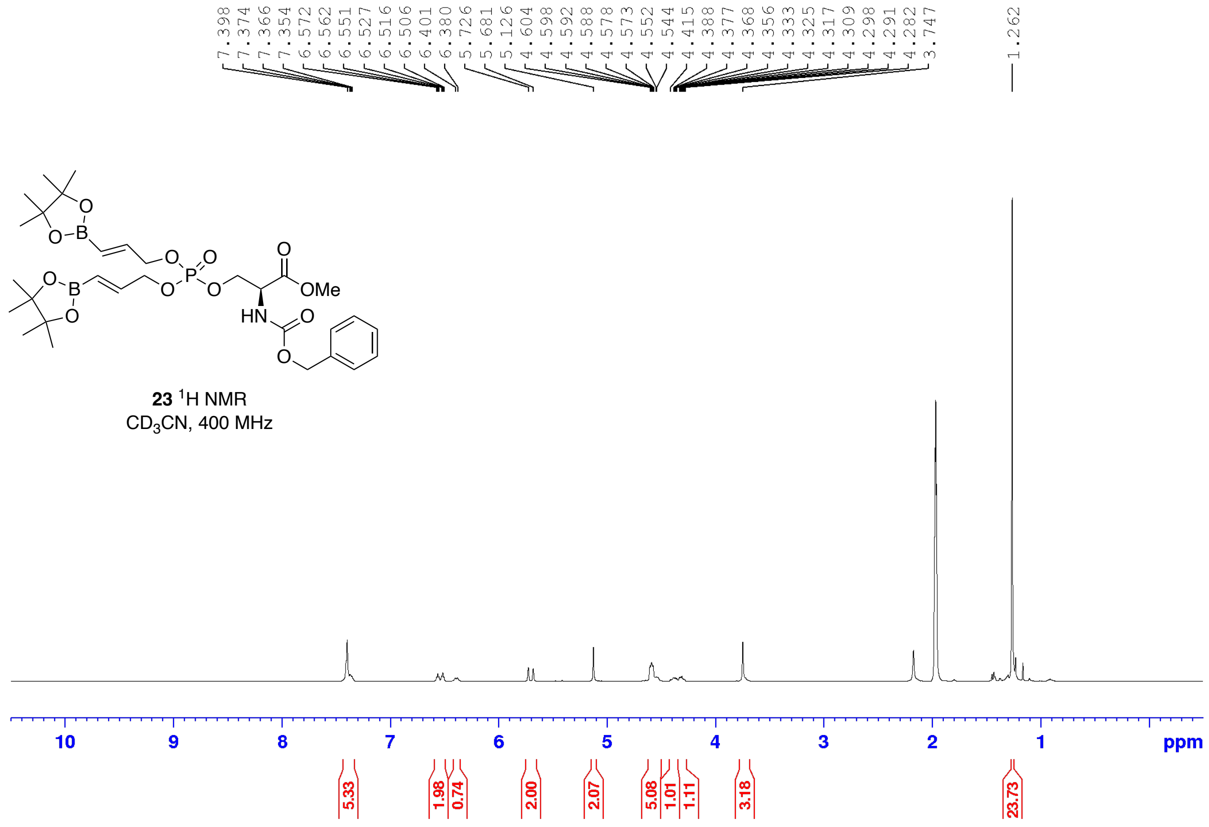
7.398
7.374
7.366
7.354
6.572
6.562
6.551
6.527
6.516
6.506
6.401
6.380
5.726
5.681
5.126
4.604
4.598
4.592
4.588
4.578
4.573
4.552
4.544
4.415
4.388
4.377
4.368
4.356
4.333
4.325
4.317
4.309
4.298
4.291
4.282
3.747
1.262
OMe
HN
23 ^{1}H NMR
CD_3CN, 400 MHz
ppm
5.33
1.98
0.74
2.00
2.07
5.08
1.01
1.11
3.18
23.73

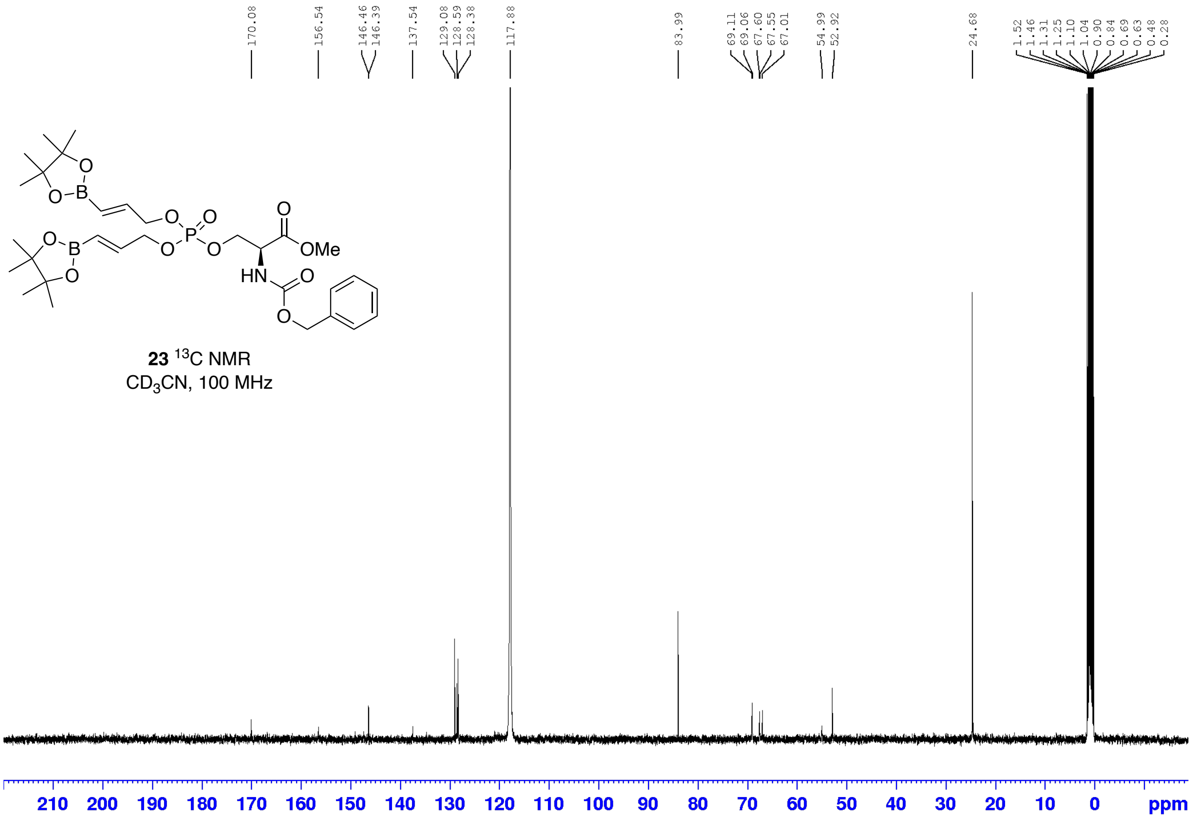
170.08
156.54
146.46
146.39
137.54
129.08
128.59
128.38
117.88
83.99
69.11
69.06
67.60
67.55
67.01
54.99
52.92
24.68
1.52
1.46
1.31
1.25
1.10
1.04
0.90
0.84
0.69
0.63
0.48
0.28
OMe
HN
23 ^{13}C NMR
CD_3CN, 100 MHz
ppm

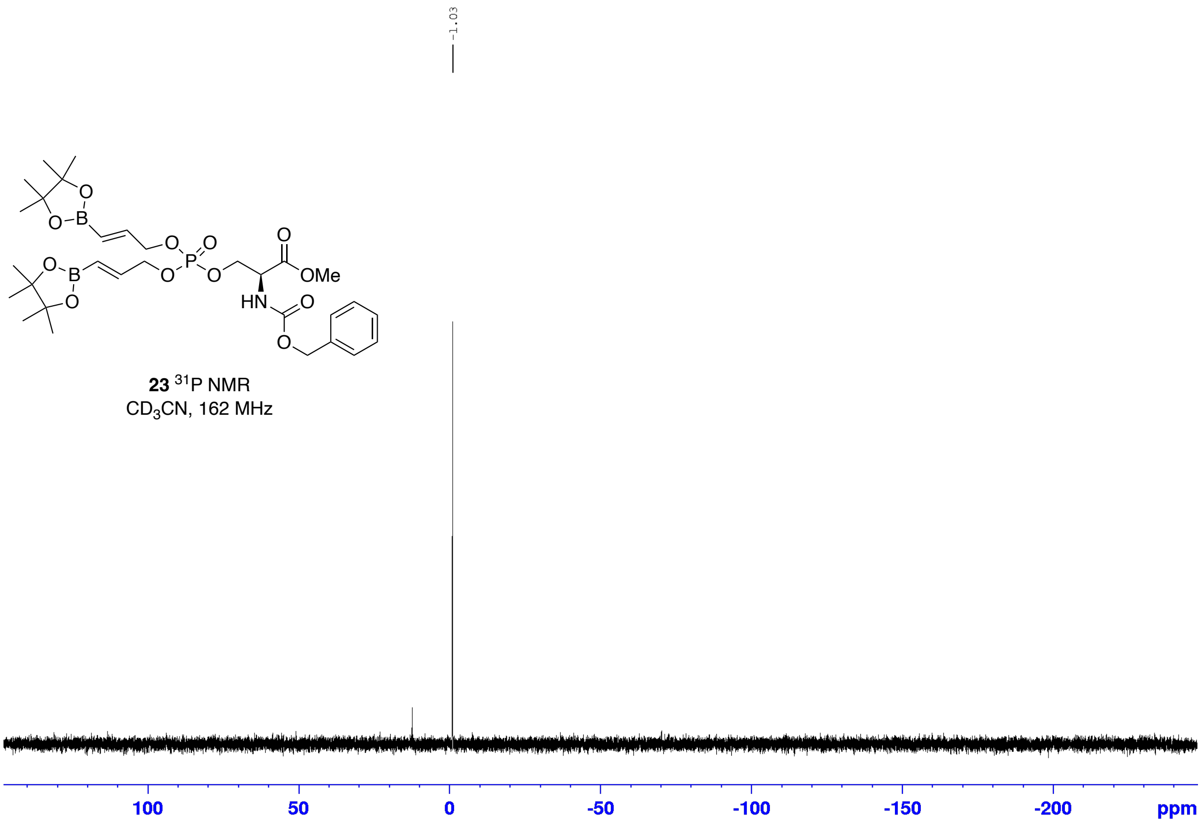
-1.03
O
O
B
O
O
P
O
O
O
O
OMe
O
O
B
HN
O
O
23 ³¹P NMR
CD3CN, 162 MHz
100
50
0
-50
-100
-150
-200
ppm

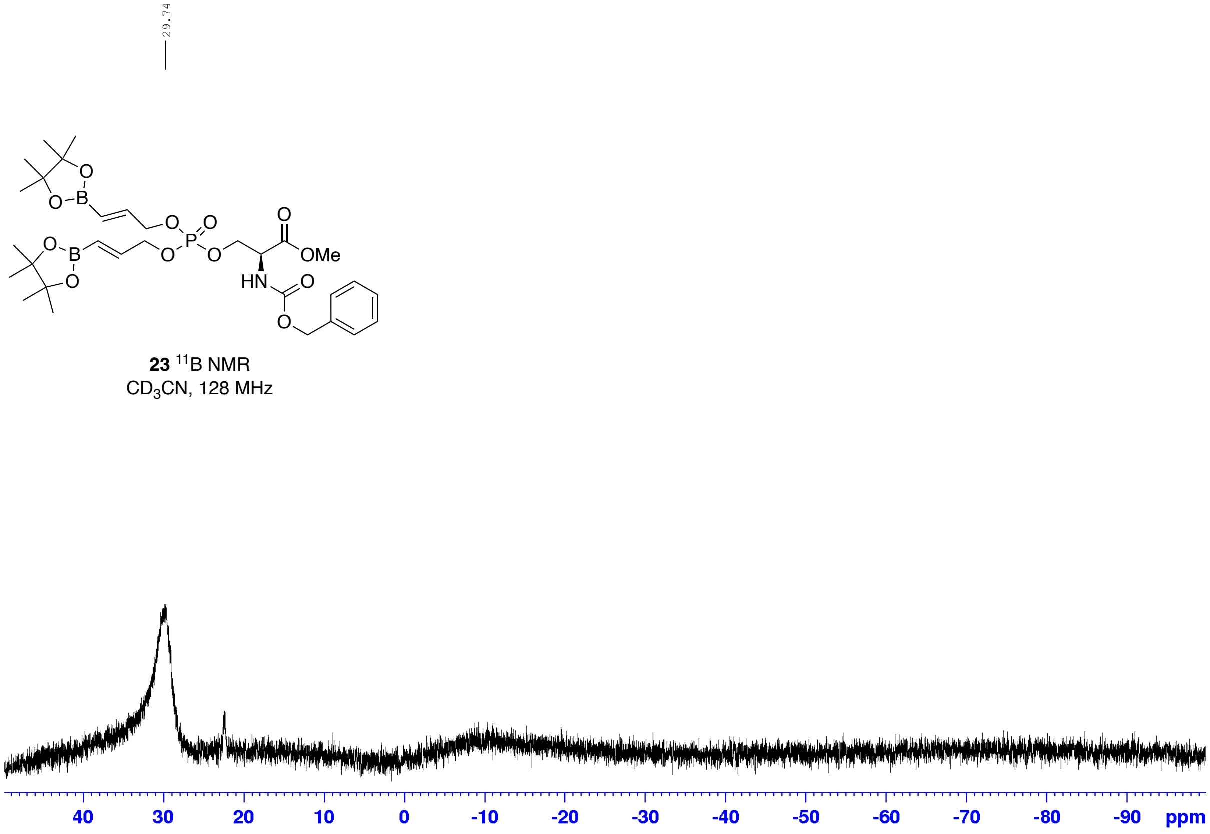
29.74
O
O
B
O
O
P
O
O
O
O
OMe
O
O
B
HN
O
O
23 ¹¹B NMR
CD3CN, 128 MHz
40
30
20
10
0
-10
-20
-30
-40
-50
-60
-70
-80
-90
ppm

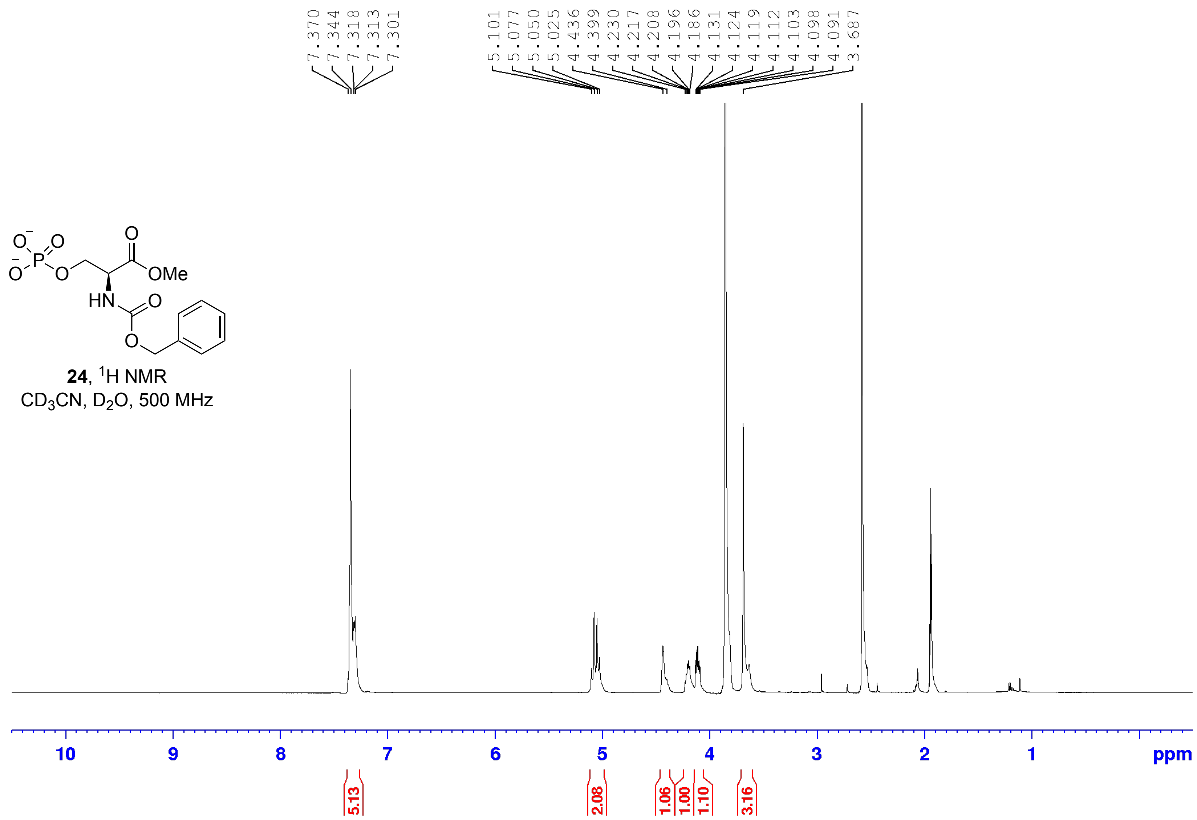
7.370
7.344
7.318
7.313
7.301
5.101
5.077
5.050
5.025
4.436
4.399
4.230
4.217
4.208
4.196
4.186
4.131
4.124
4.119
4.112
4.103
4.098
4.091
3.687
24, ¹H NMR
CD3CN, D2O, 500 MHz
5.13
2.08
1.06
1.00
1.10
3.16
ppm

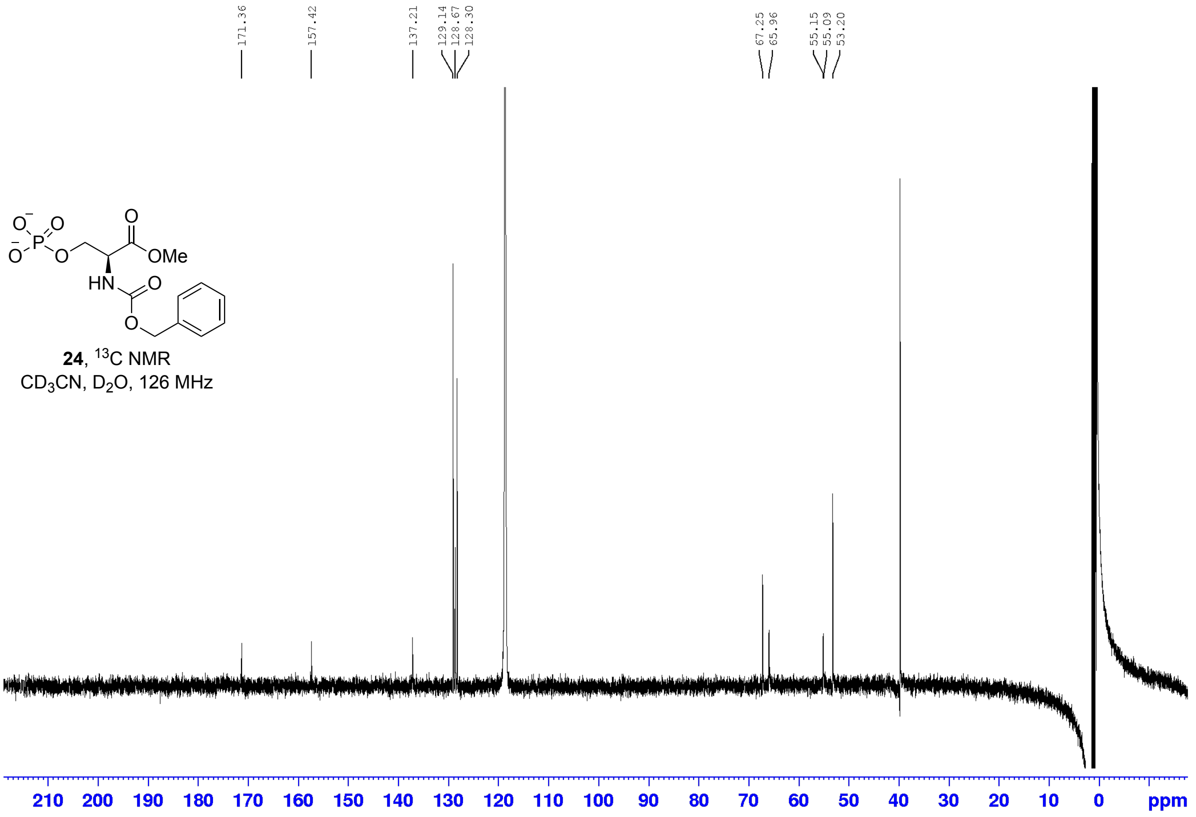
171.36
157.42
137.21
129.14
128.67
128.30
67.25
65.96
55.15
55.09
53.20
24, ¹³C NMR
CD3CN, D2O, 126 MHz
ppm

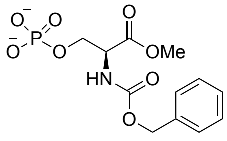

24, ^{31}P NMR
CD_3CN, D_2O, 202 MHz

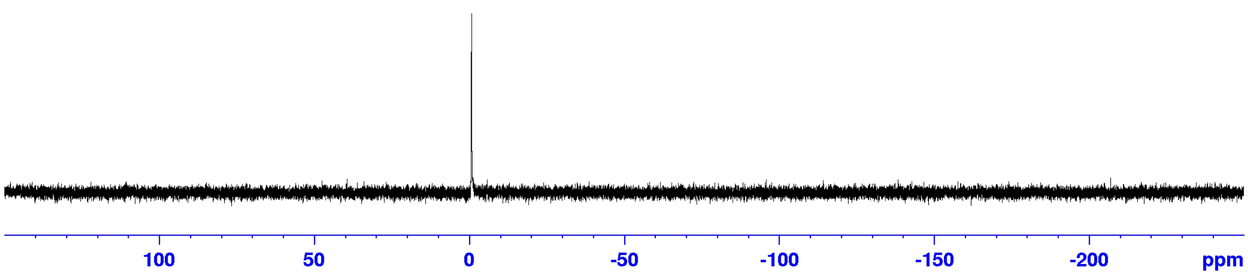

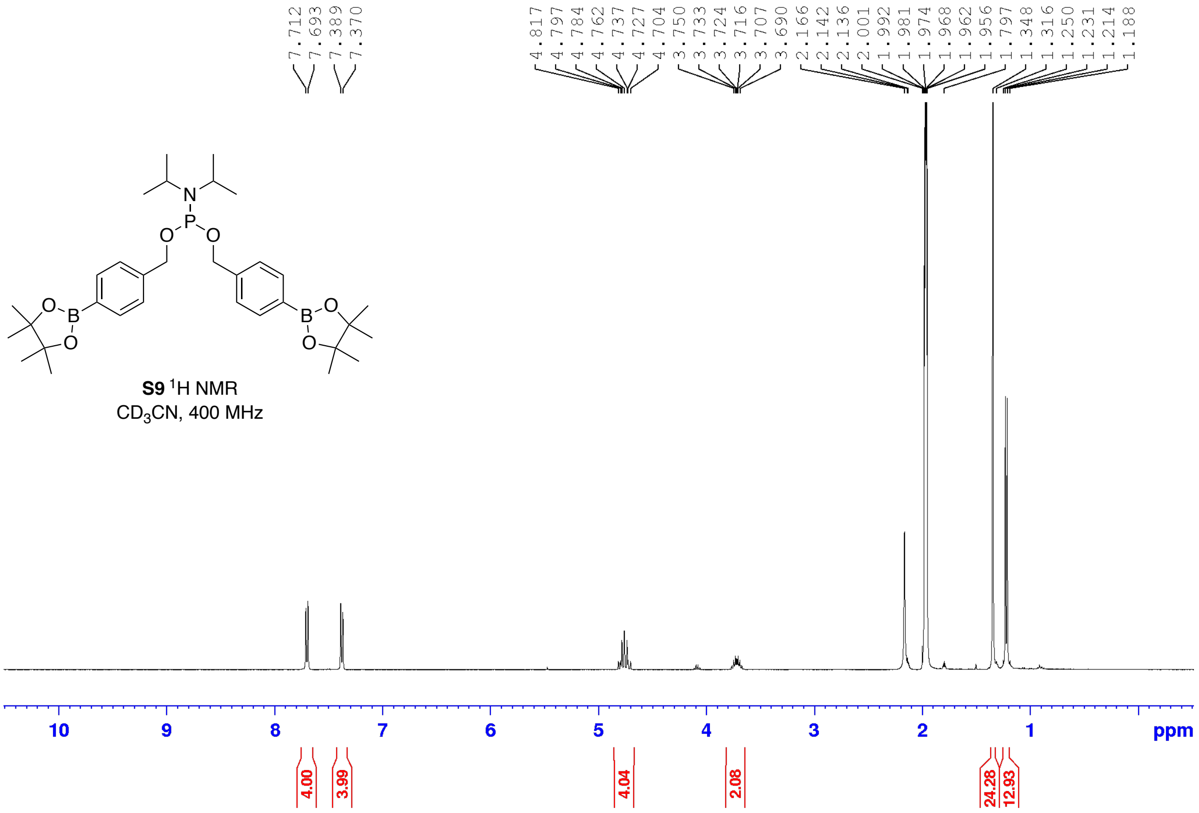

7.712
7.693
7.389
7.370
4.817
4.797
4.784
4.762
4.737
4.727
4.704
3.750
3.733
3.724
3.716
3.707
3.690
2.166
2.142
2.136
2.001
1.992
1.981
1.974
1.968
1.962
1.956
1.797
1.348
1.316
1.250
1.231
1.214
1.188
S9 ¹H NMR
CD3CN, 400 MHz
10
9
8
7
6
5
4
3
2
1
ppm
4.00
3.99
4.04
2.08
24.28
12.93

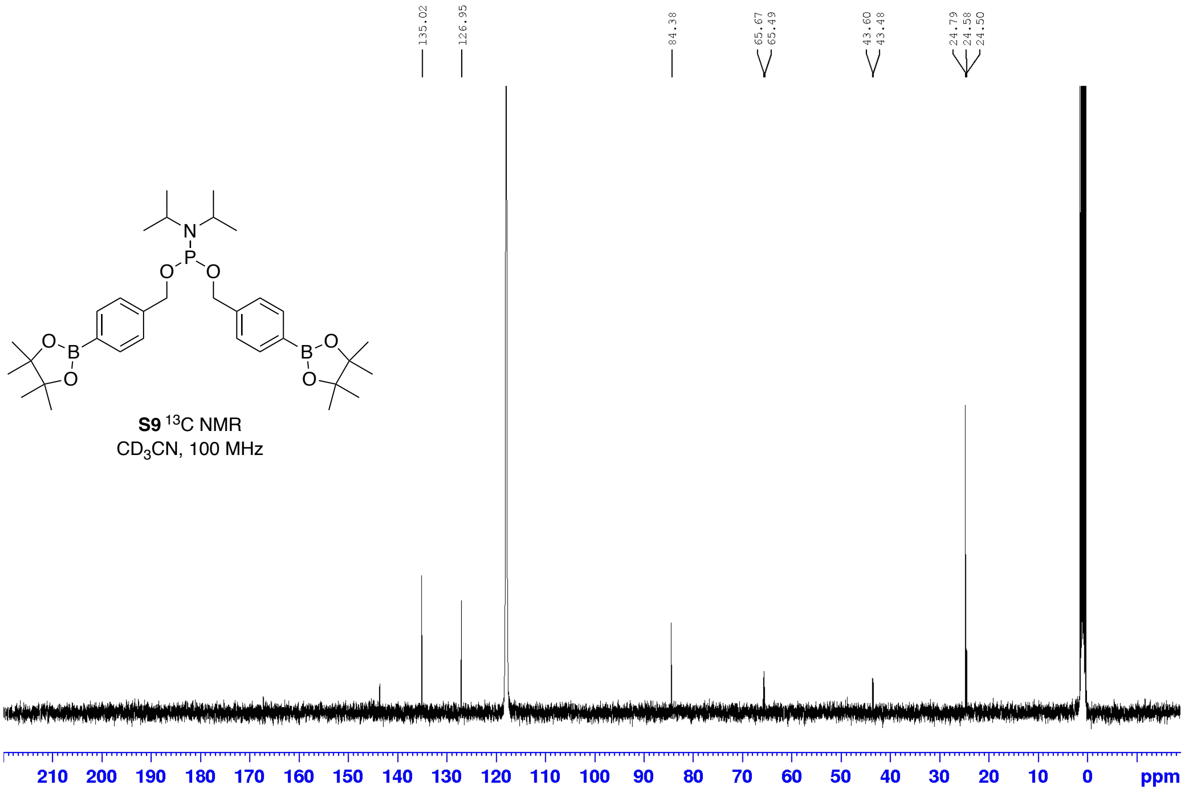

135.02
126.95
84.38
65.67
65.49
43.60
43.48
24.79
24.58
24.50
S9 ¹³C NMR
CD3CN, 100 MHz
210
200
190
180
170
160
150
140
130
120
110
100
90
80
70
60
50
40
30
20
10
0
ppm

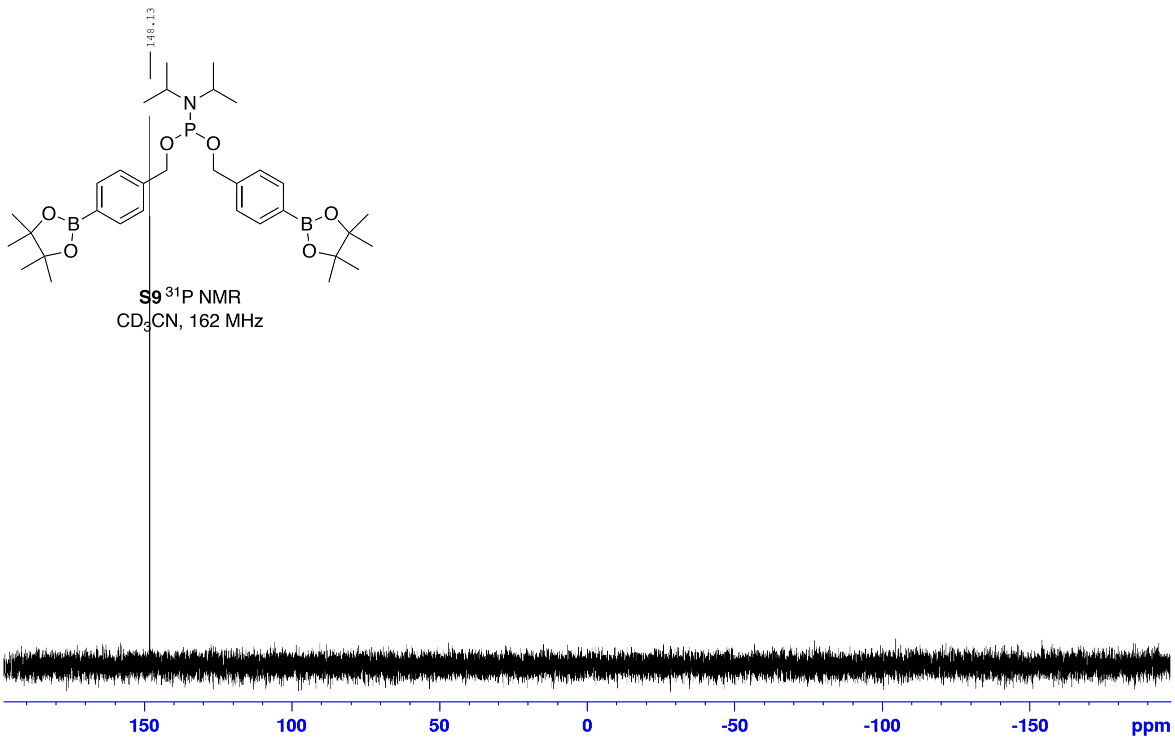
148.13
N
P
O
O
O
B
O
B
O
O
S9 ³¹P NMR
CD3CN, 162 MHz
150
100
50
0
-50
-100
-150
ppm

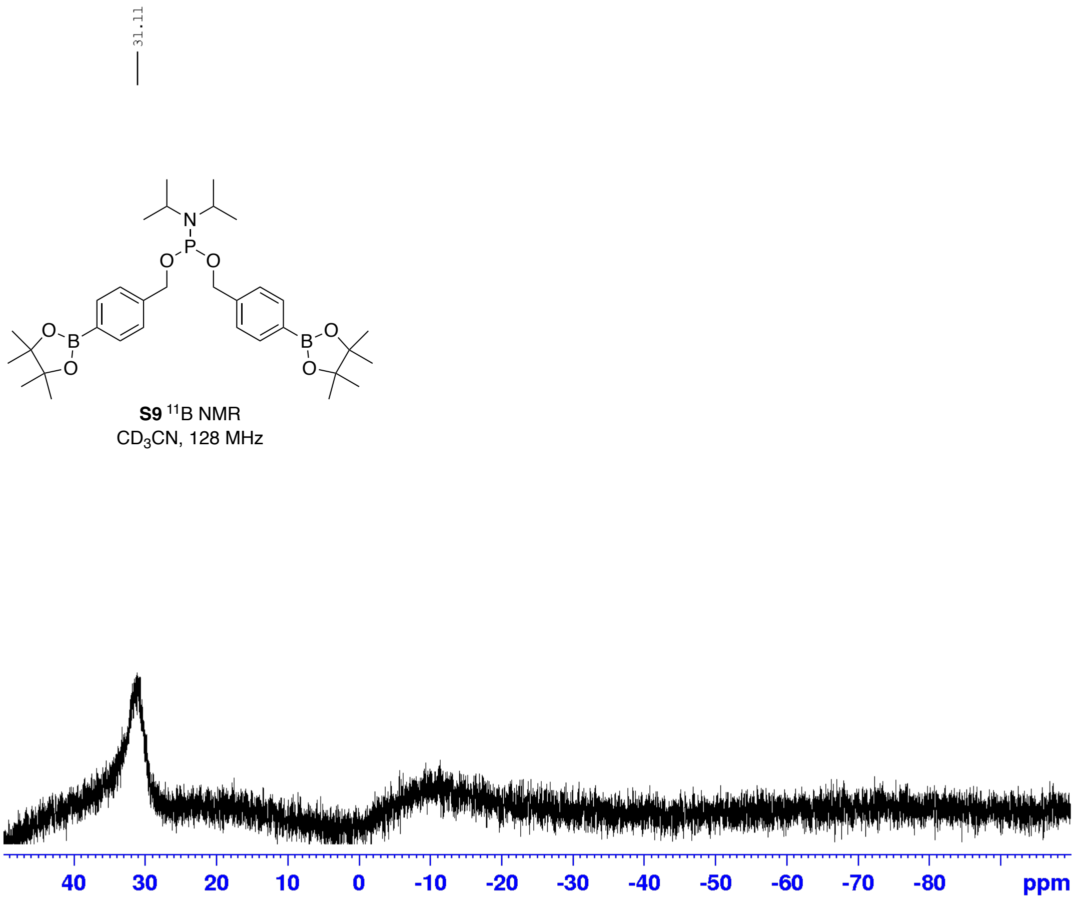
31.11
N
P
O
O
O
B
O
B
O
O
S9 ¹¹B NMR
CD3CN, 128 MHz
40
30
20
10
0
-10
-20
-30
-40
-50
-60
-70
-80
ppm

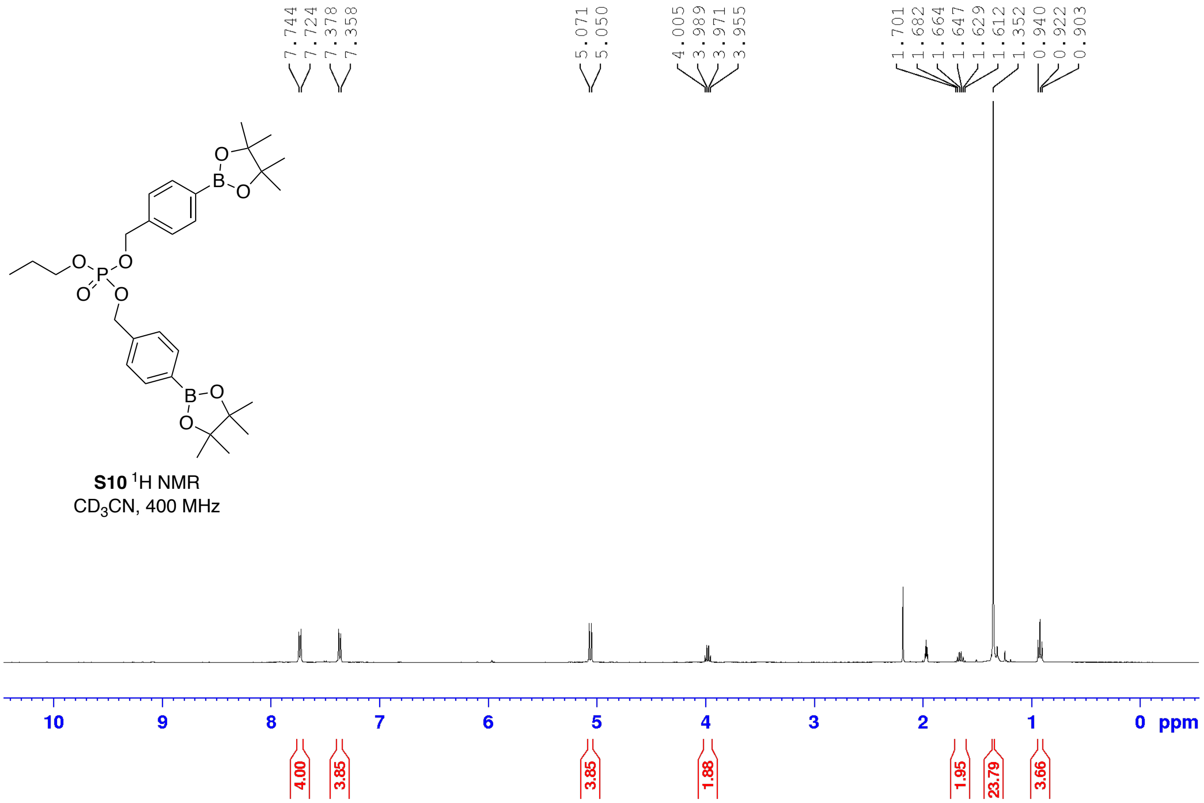

S10 ^{1}H NMR
CD_3CN, 400 MHz

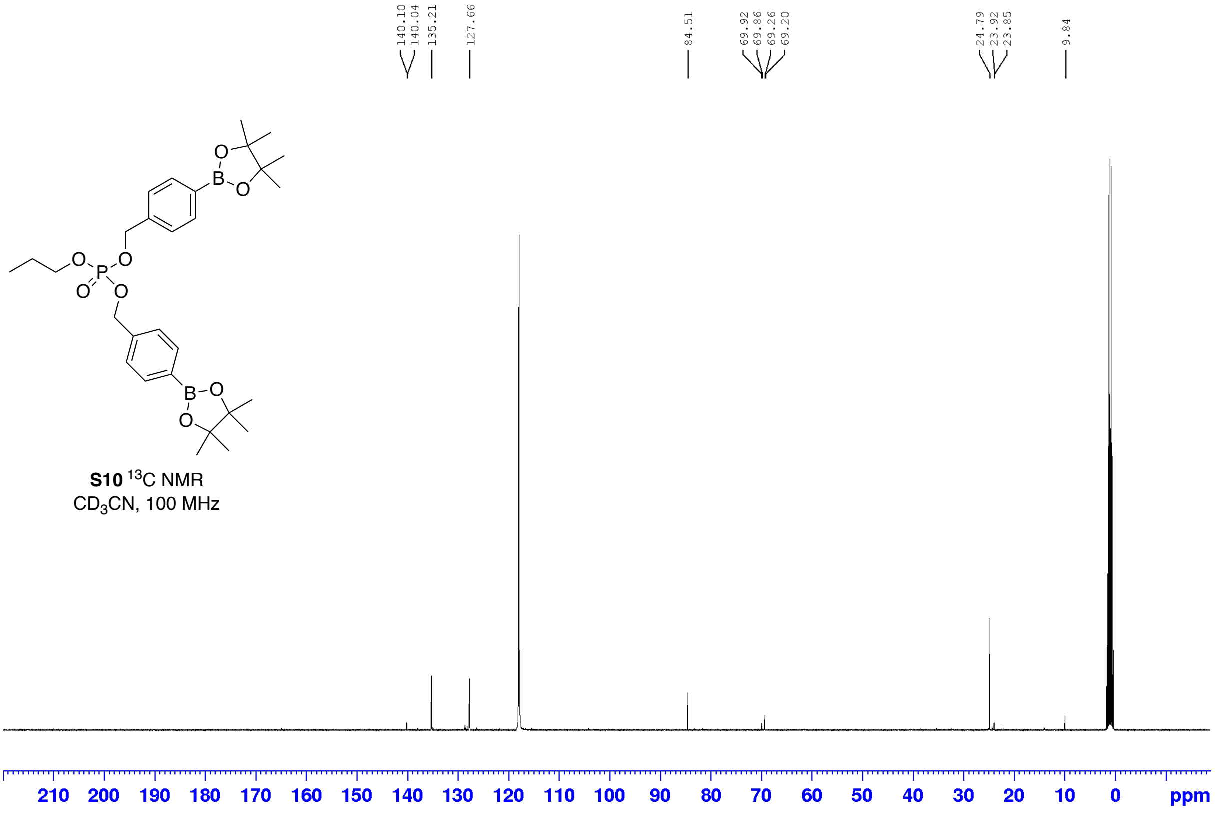

S10 ^{13}C NMR
CD_3CN, 100 MHz

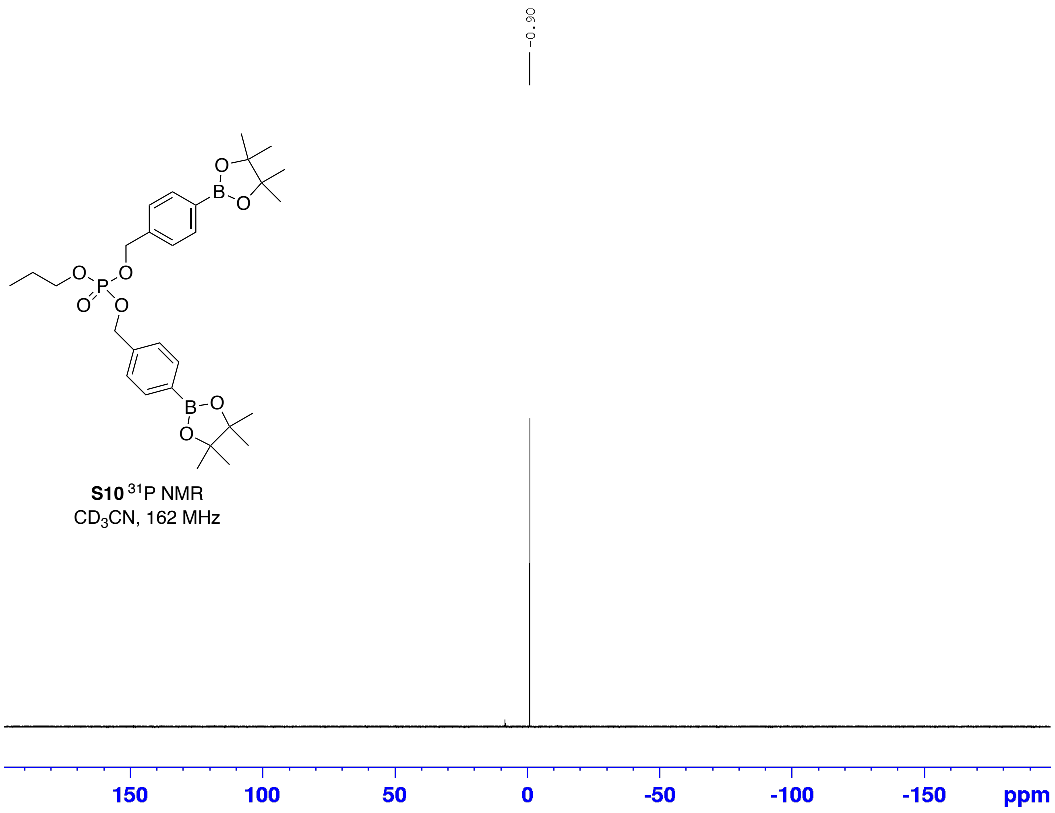
-0.90
S10 31P NMR
CD3CN, 162 MHz
150
100
50
0
-50
-100
-150
ppm

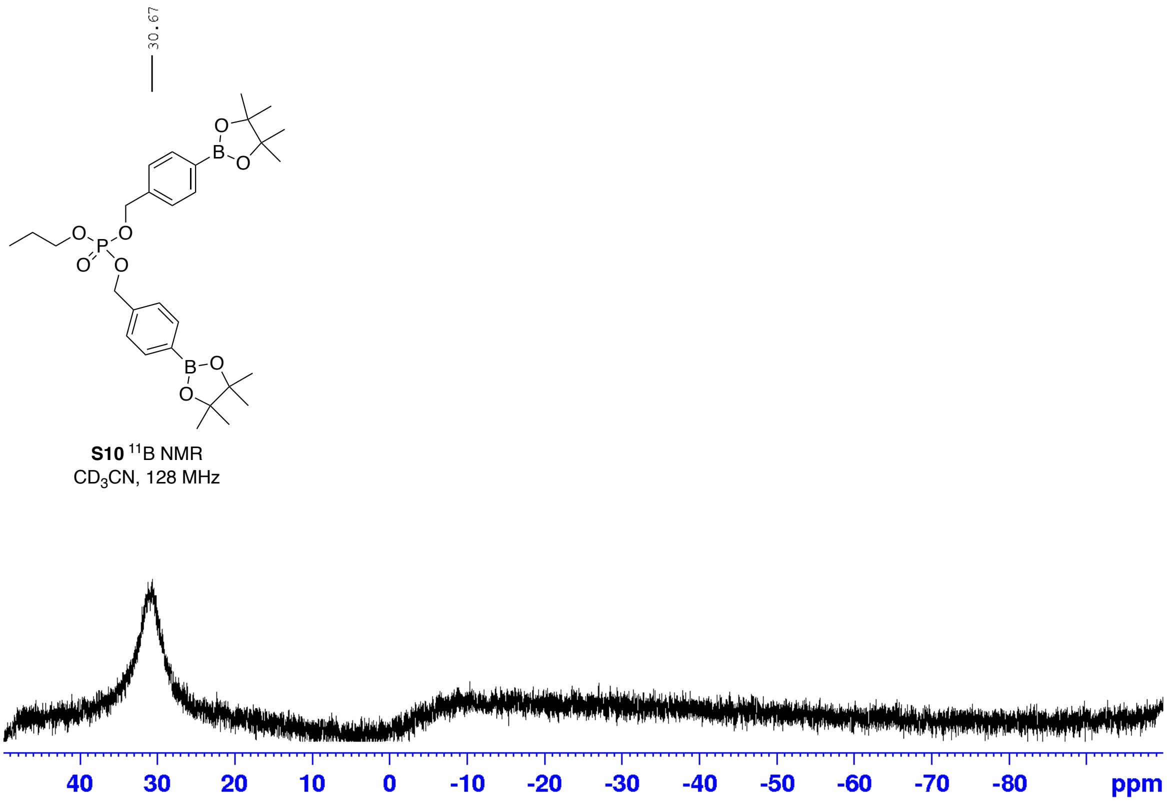
30.67
S10 11B NMR
CD3CN, 128 MHz
40
30
20
10
0
-10
-20
-30
-40
-50
-60
-70
-80
ppm

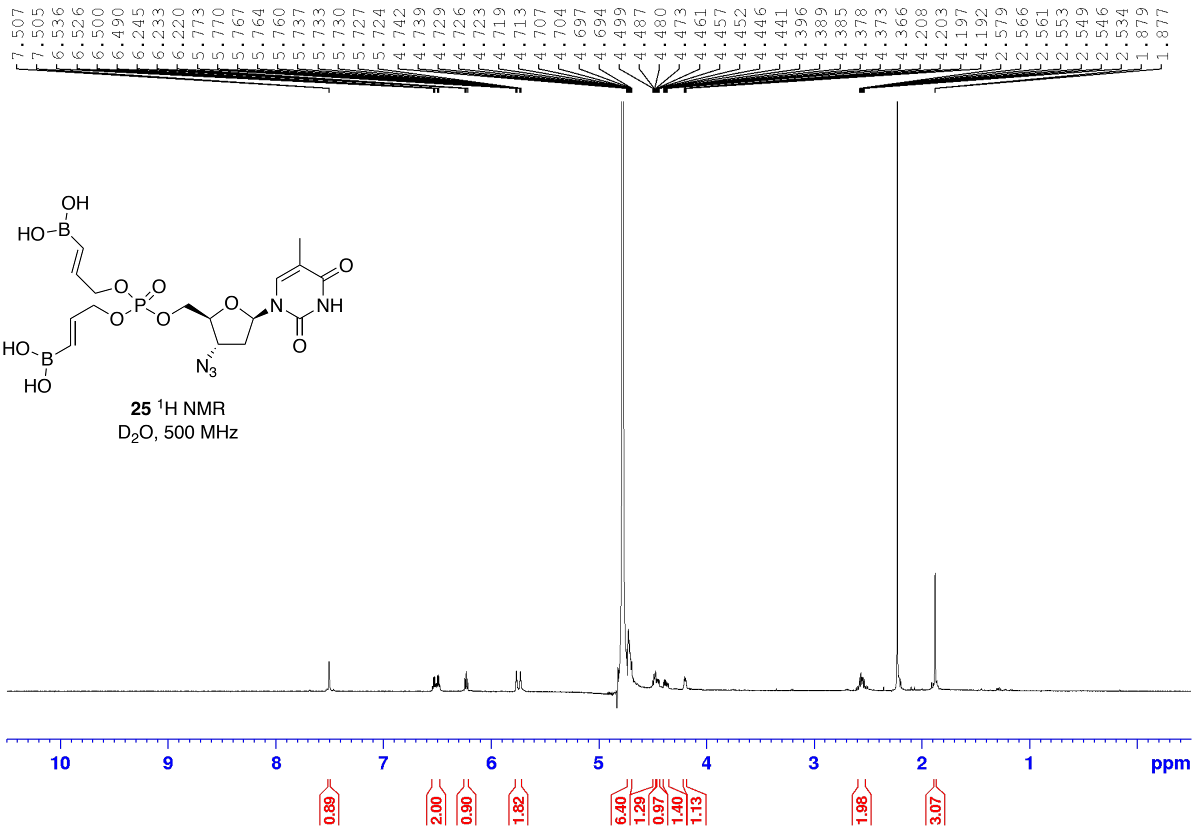

25 ^{1}H NMR
D_2O, 500 MHz

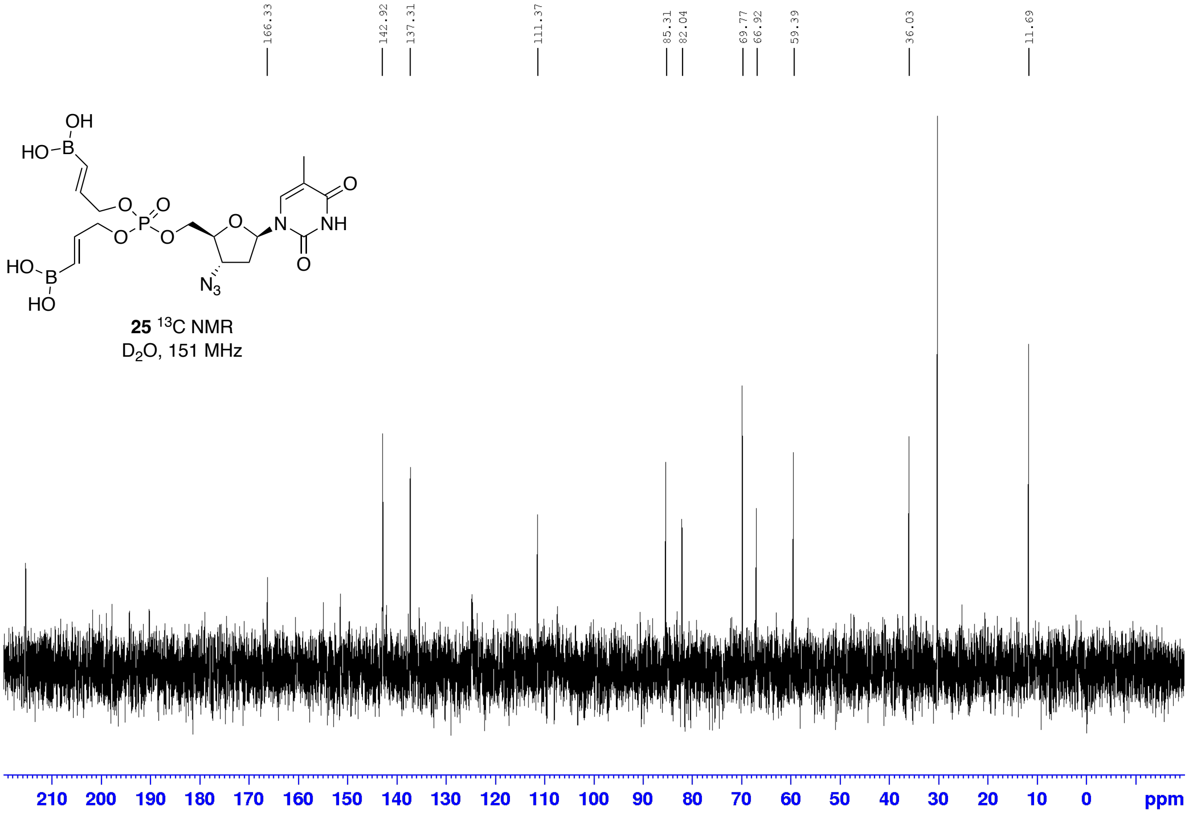

25 ^{13}C NMR
D_2O, 151 MHz

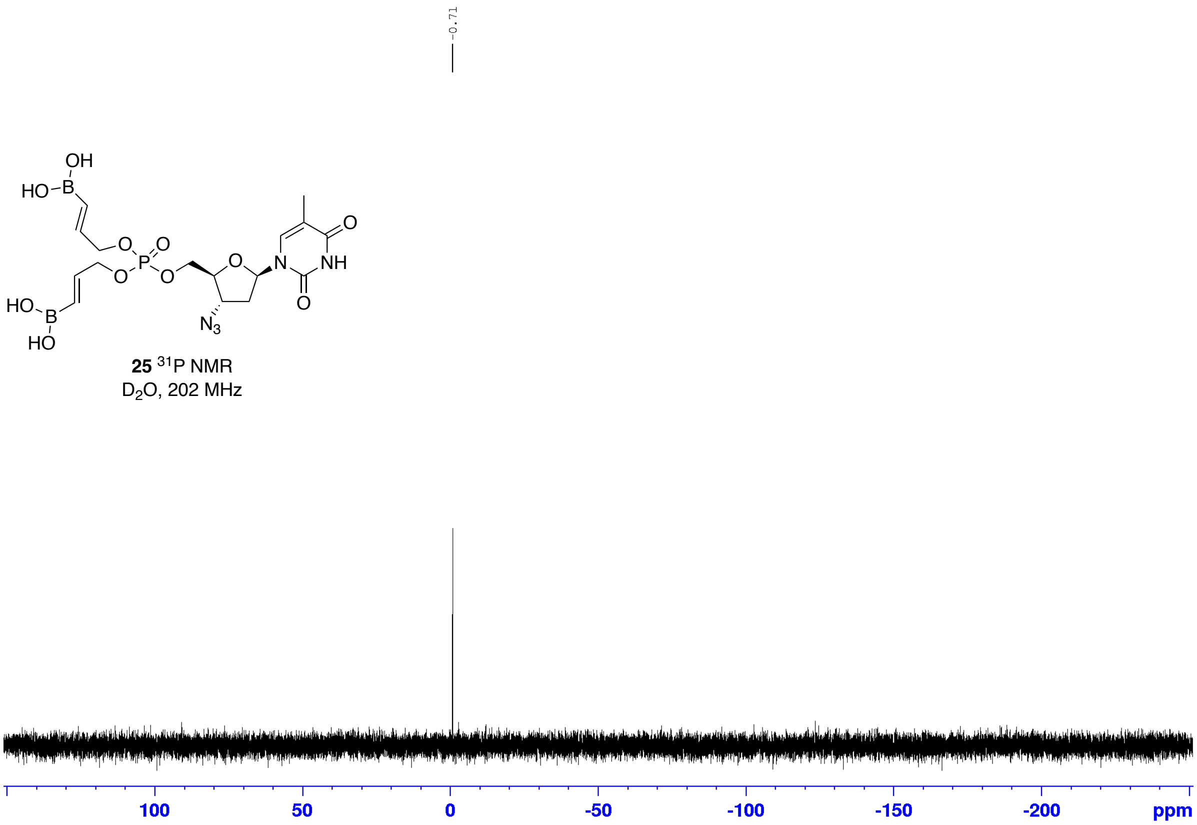
-0.71
OH
HO
B
O
P
O
O
O
O
N
O
NH
O
HO
B
HO
N3
25 31P NMR
D2O, 202 MHz
100
50
0
-50
-100
-150
-200
ppm

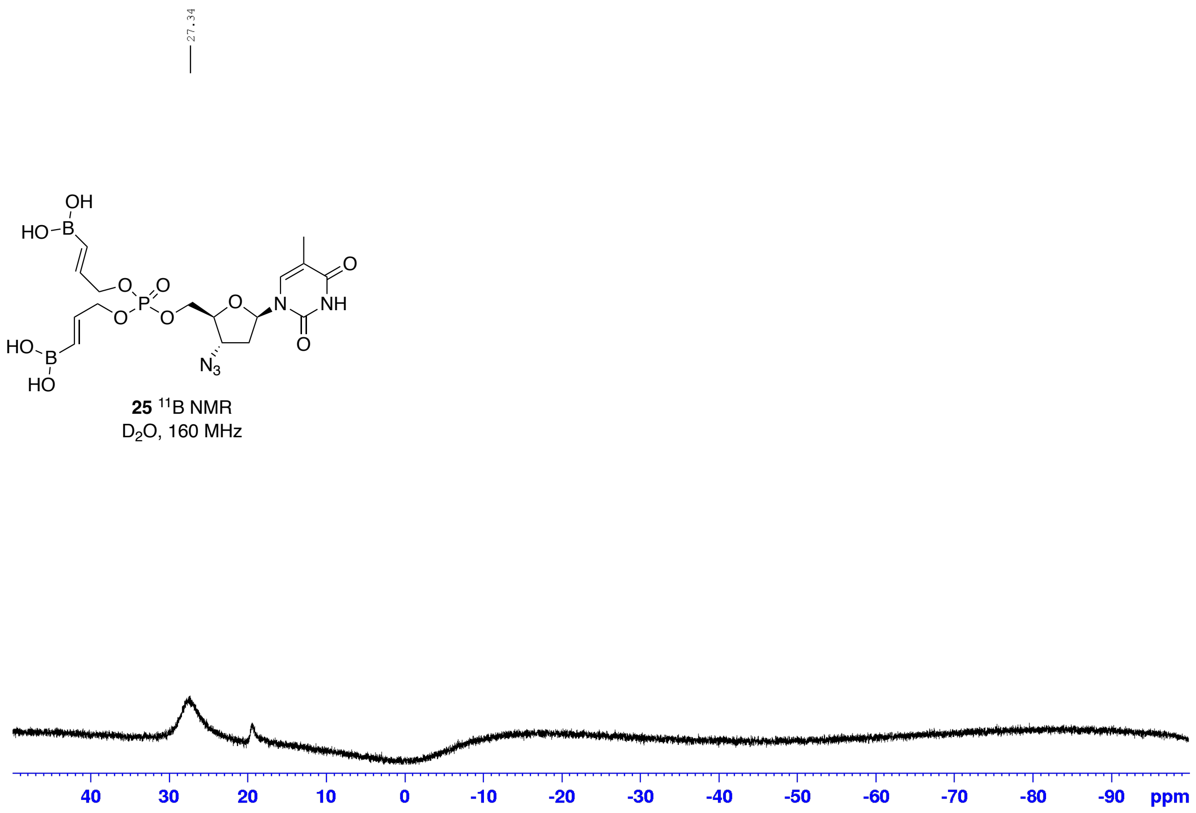
27.34
OH
HO
B
O
P
O
O
O
O
N
O
NH
O
HO
B
HO
N3
25 11B NMR
D2O, 160 MHz
40
30
20
10
0
-10
-20
-30
-40
-50
-60
-70
-80
-90
ppm

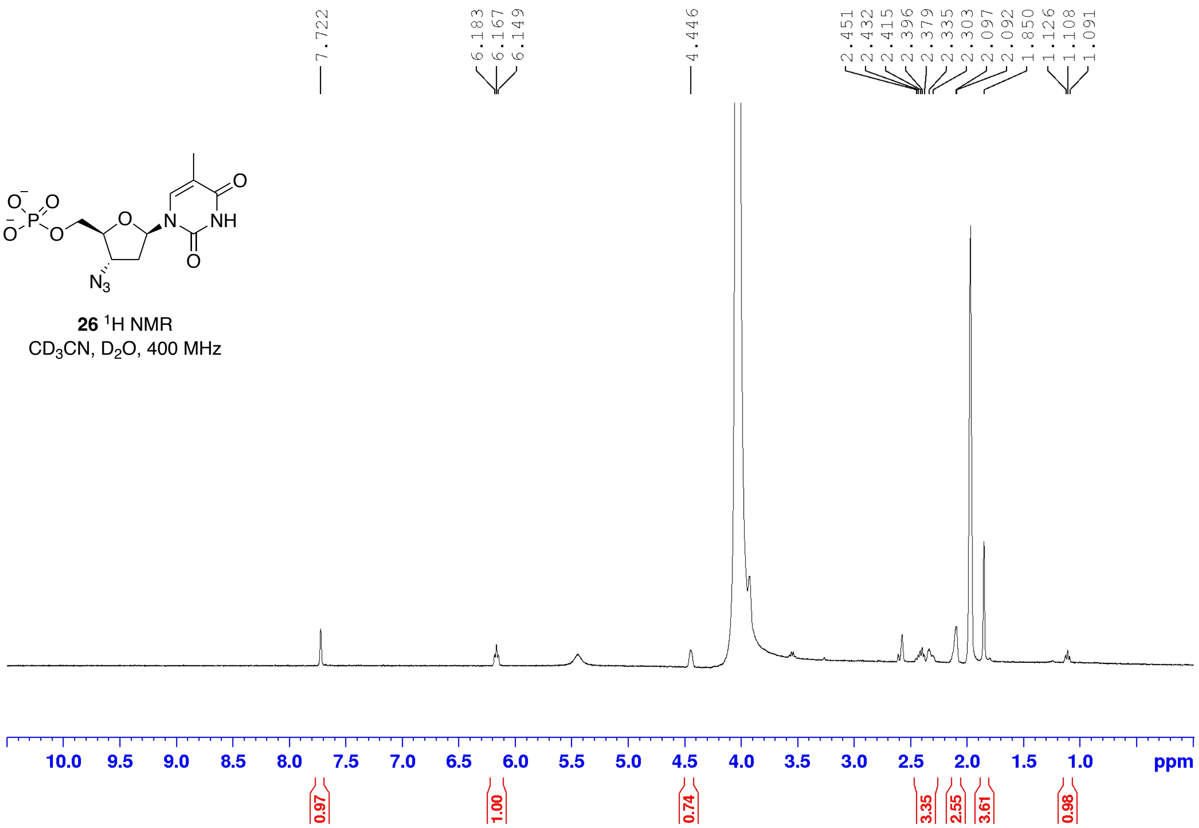
7.722
6.183
6.167
6.149
4.446
2.451
2.432
2.415
2.396
2.379
2.335
2.303
2.097
2.092
1.850
1.126
1.108
1.091
26 1H NMR
CD3CN, D2O, 400 MHz
10.0 9.5 9.0 8.5 8.0 7.5 7.0 6.5 6.0 5.5 5.0 4.5 4.0 3.5 3.0 2.5 2.0 1.5 1.0 ppm
0.97
1.00
0.74
3.35
2.55
3.61
0.98

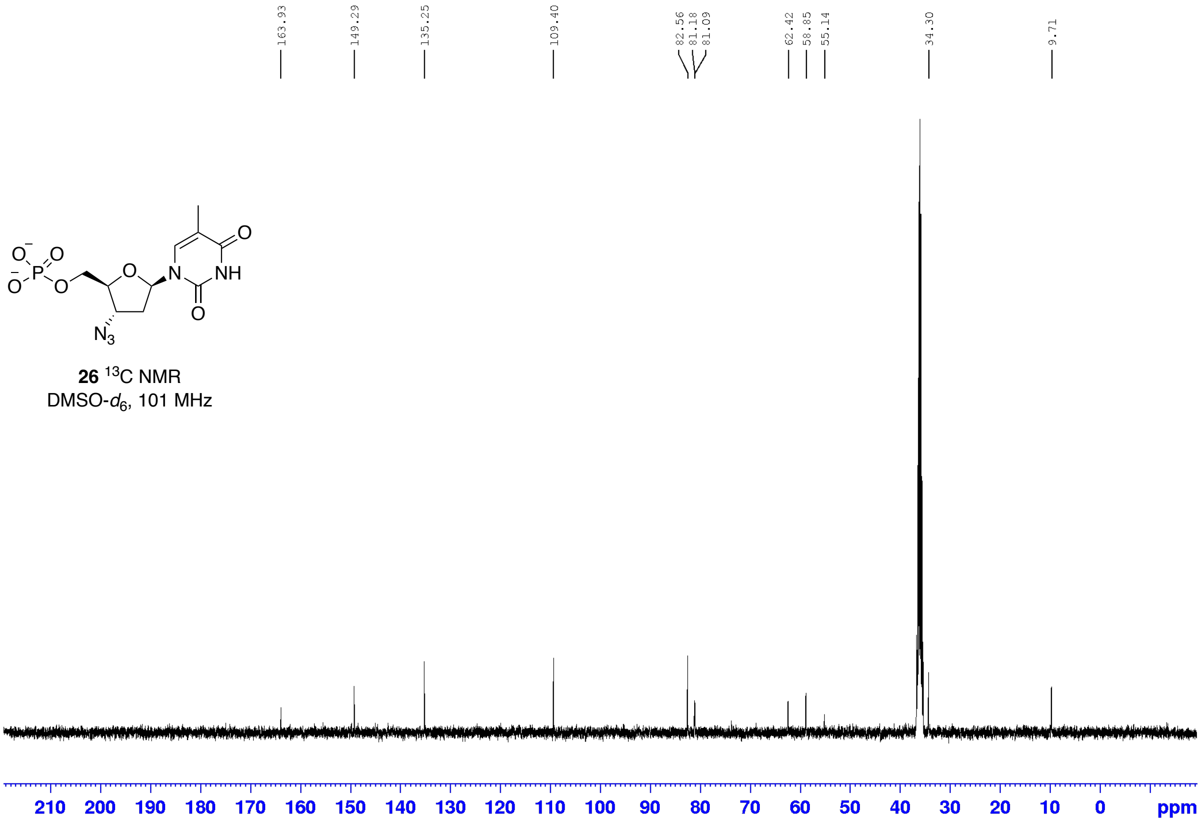
163.93
149.29
135.25
109.40
82.56
81.18
81.09
62.42
58.85
55.14
34.30
9.71
26 13C NMR
DMSO-d6, 101 MHz
210 200 190 180 170 160 150 140 130 120 110 100 90 80 70 60 50 40 30 20 10 0 ppm

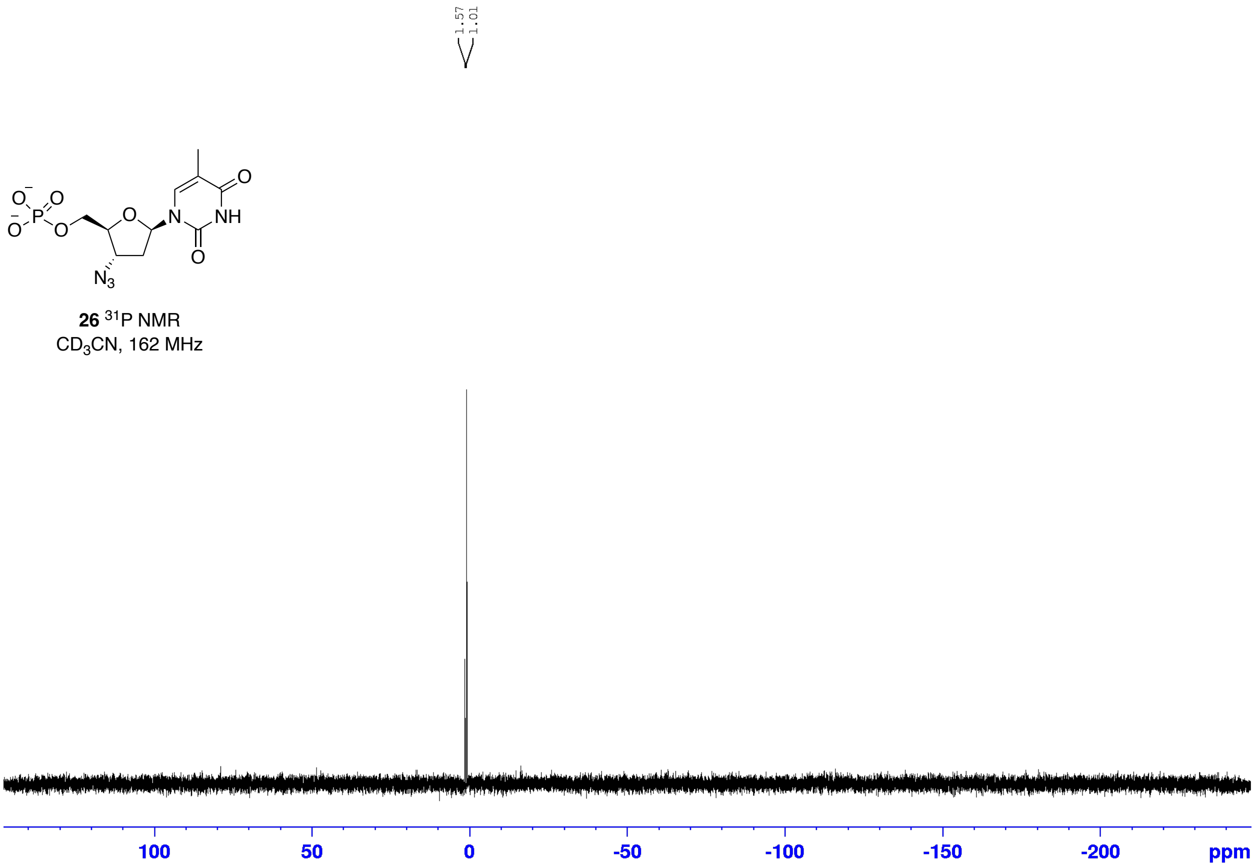

1.57
1.01
26 ^{31}P NMR
CD_3CN, 162 MHz
100
50
0
-50
-100
-150
-200
ppm

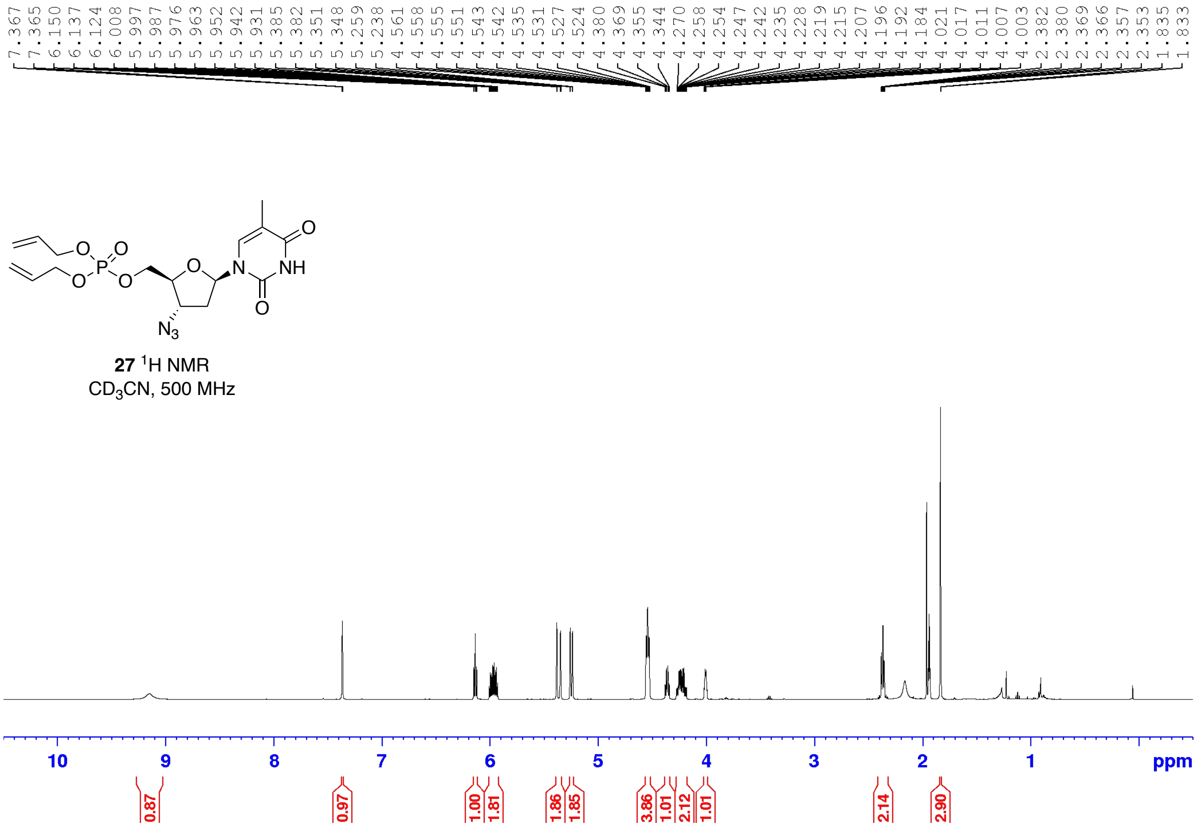

27 ^{1}H NMR
CD_3CN, 500 MHz

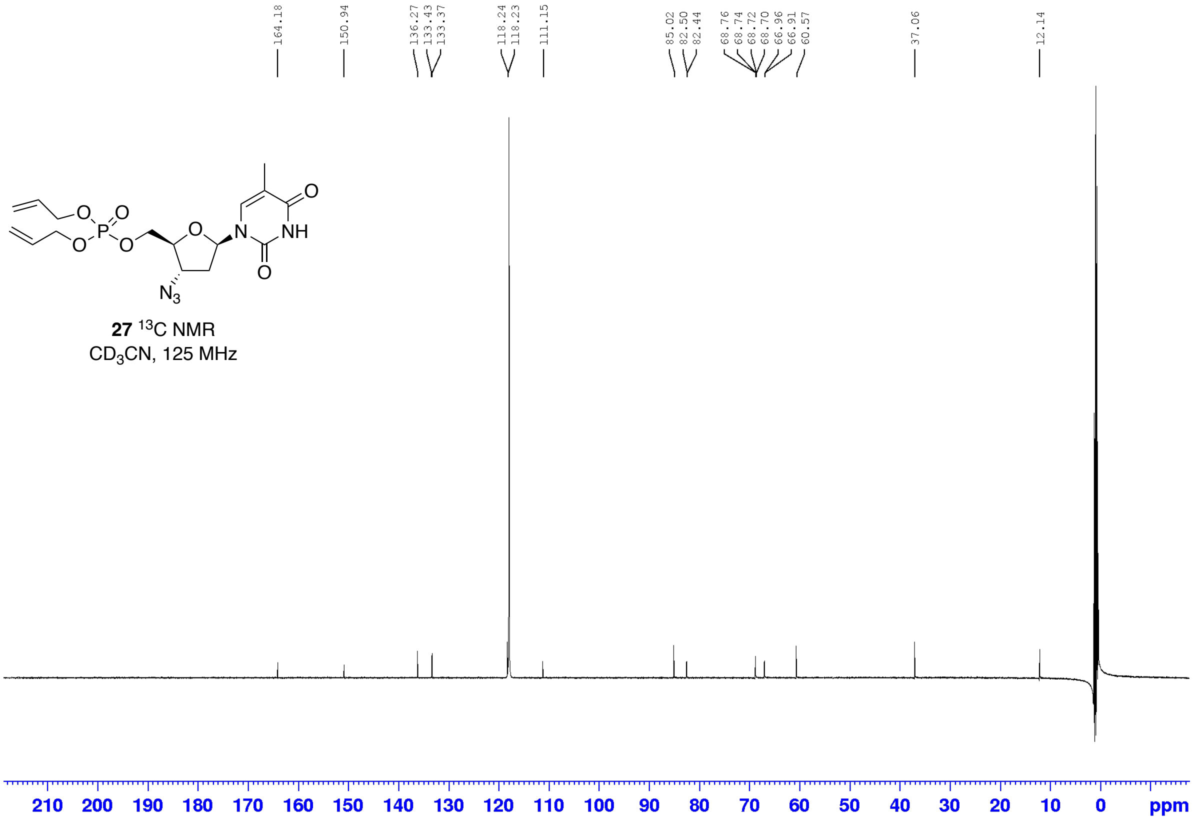

27 ^{13}C NMR
CD_3CN, 125 MHz

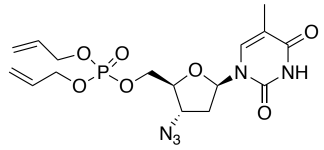

27 ^{31}P NMR
CD_3CN, 202 MHz

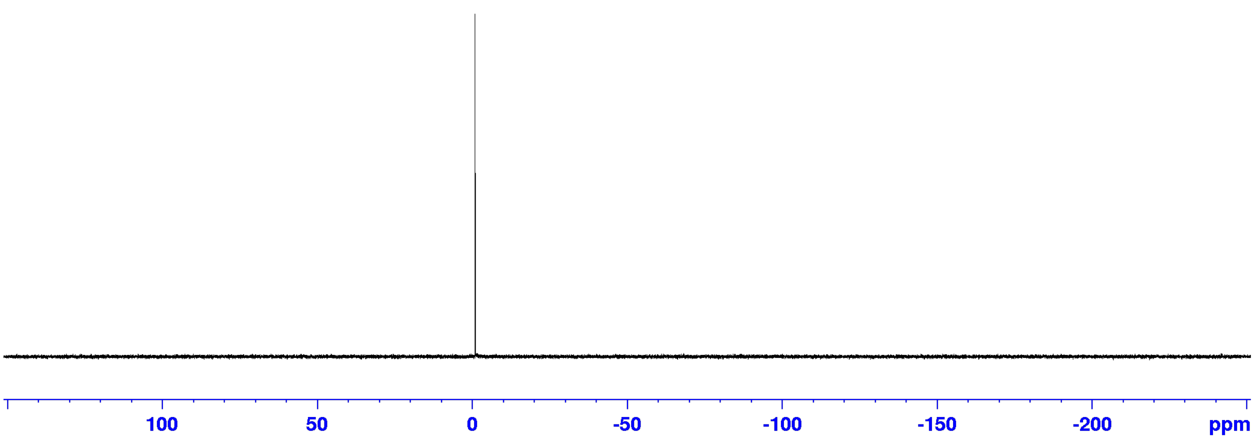

References

1. Wrackmeyer, B. Carbon-13 NMR spectroscopy of boron compounds. *Prog. Nucl. Magn. Reson. Spectrosc.* **1979**, *12*, 227-259.

2. Harris, R. K.; Becker, E. D.; Cabral De Menezes, S. M.; Granger, P.; Hoffman, R. E.; Zilm, K. W. Further conventions for NMR shielding and chemical shifts (IUPAC recommendations 2008). *Pure Appl. Chem.* **2008**, *80*, 59-84.

3. Reid, W. B.; Spillane, J. J.; Krause, S. B.; Watson, D. A. Direct Synthesis of Alkenyl Boronic Esters from Unfunctionalized Alkenes: A Boryl Heck Reaction. *J. Am. Chem. Soc.* **2016**, *138*, 5539-5542.

4. Hitosugi, S.; Tanimoto, D.; Nakanishi, W.; Isobe, H. A Facile Chromatographic Method for the Purification of Pinacol Boronic Esters. *Chem. Lett.* **2012**, *41*, 972-973.

5. Clarke, P. A.; Rolla, G. A.; Cridland, A. P.; Gill, A. A. An improved synthesis of (2E,4Z)-6-(benzyloxy)-4-bromohexa-2,4-dien-1-ol. *Tetrahedron* **2007**, *63*, 9124-9128.

6. Villar, L.; Orlov, N. V.; Kondratyev, N. S.; Uria, U.; Vicario, J. L.; Malkov, A. V. Kinetic Resolution of Secondary Allyl Boronates and Their Application in the Synthesis of Homoallylic Amines. *Chem. Eur. J.* **2018**, *24*, 16262-16265.

7. Yuan, W.; Ma, S. CuCl-K2CO3-catalyzed highly selective borylcupration os internal alkynes – ligand effect. *Org. Biomol. Chem.* **2012**, *10*, 7266-7268.

8. G. Gomori, in *Handbook of Biochemistry and Molecular Biology*, Fourth Edition, CRC Press: 2010; p 721.

9. Hanna, R. D.; Naro, Y.; Deiters, A.; Floreancig, P. E. Alcohol, Aldehyde, and Ketone Liberation and Intracellular Cargo Release through Peroxide-Mediated α-Boryl Ether Fragmentation. *J. Am. Chem. Soc.* **2016**, *138*, 13353-13360.

10. Orellana, E. A.; Kasinski, A. L. Sulforhodamine B (SRB) Assay in Cell Culture to Investigate Cell Proliferation. *Bio Protoc* **2016**, *6*, e1984.

11. Malinouski, M.; Zhou, Y.; Belousov, V. V.; Hatfield, D. L.; Gladyshev, V. N. Hydrogen peroxide probes directed to different cellular compartments. *PLoS One* **2011**, *6*, e14564.
