# Supplementary figures and images for: Peroxide-Mediated Release of Organophosphates from Boron-Containing Phosphotriesters: A New Class of Organophosphate Prodrugs
Source: Org Lett. 2023 Jul 18;25(29):5530–5. doi: 10.1021/acs.orglett.3c02036 (PMC10391626; doi:10.1021/acs.orglett.3c02036)

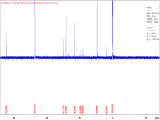

Supplement: Supplementary file 2 — ol3c02036_si_002.zip [file ol3c02036_si_002.zip › FID for Publication/Compound 11/13C/10/pdata/1/thumb.png]
